# Comprehensive RNA dataset of tissue and plasma from patients with esophageal cancer or precursor lesions

**DOI:** 10.1038/s41597-022-01176-x

**Published:** 2022-03-14

**Authors:** Kathleen Schoofs, Annouck Philippron, Francisco Avila Cobos, Jan Koster, Steve Lefever, Jasper Anckaert, Danny De Looze, Jo Vandesompele, Piet Pattyn, Katleen De Preter

**Affiliations:** 1 Translational Oncogenomics and Bioinformatics Lab, Cancer Research Institute Ghent (CRIG), Ghent, Belgium; 2 OncoRNALab, Cancer Research Institute Ghent (CRIG), Ghent, Belgium; 3 Department of Biomolecular Medicine, Ghent University, Ghent, Belgium; 4 Department of Gastro-Intestinal Surgery, University Hospital Ghent, Belgium; 5 Department of Oncogenomics, Academic Medical Center (AMC), Amsterdam, The Netherlands; 6 Department of Gastroenterology and Hepatology, University Hospital Ghent, Ghent, Belgium; 7 Department of Human Structure and Repair, Ghent University, Ghent, Belgium

**Keywords:** Gene expression, Biomarkers, Oesophageal cancer, Data mining, Cancer genomics

## Abstract

In the past decades, the incidence of esophageal adenocarcinoma has increased dramatically in Western populations. Better understanding of disease etiology along with the identification of novel prognostic and predictive biomarkers are urgently needed to improve the dismal survival probabilities. Here, we performed comprehensive RNA (coding and non-coding) profiling in various samples from 17 patients diagnosed with esophageal adenocarcinoma, high-grade dysplastic or non-dysplastic Barrett’s esophagus. Per patient, a blood plasma sample, and a healthy and disease esophageal tissue sample were included. In total, this comprehensive dataset consists of 102 sequenced libraries from 51 samples. Based on this data, 119 expression profiles are available for three biotypes, including miRNA (51), mRNA (51) and circRNA (17). This unique resource allows for discovery of novel biomarkers and disease mechanisms, comparison of tissue and liquid biopsy profiles, integration of coding and non-coding RNA patterns, and can serve as a validation dataset in other RNA landscaping studies. Moreover, structural RNA differences can be identified in this dataset, including protein coding mutations, fusion genes, and circular RNAs.

## Abstract (summary)

**Table:** 

Measurement(s)	mRNA Sequencing • MicroRNA Sequencing
Technology Type(s)	sequencer
Sample Characteristic - Organism	Homo sapiens
Sample Characteristic - Location	Belgium

## Background & Summary

Esophageal cancer is the sixth most common cause of cancer-related death worldwide^1^. The incidence of esophageal adenocarcinoma (EAC), a histological subtype of esophageal cancer, has rapidly increased in the Western world in the last decades^2^. Despite improved treatment strategies, the five-year survival rate remains unacceptably low (10–25%)^3,4^. The main risk factors to develop EAC are gastro-esophageal reflux disease (GERD), Barrett’s esophagus, smoking and age above 50 years^5^. Barrett’s esophagus is a known precursor lesion for EAC where the normal squamous mucosa of the esophagus is replaced by columnar intestinal epithelium triggered by chronical acid stress due to GERD. Specifically, GERD can cause progression from non-dysplastic Barrett’s esophagus (NDB) through the stages of low-grade dysplasia (LGD) to high-grade dysplasia (HGD), and eventually to invasive EAC^6^.

Upper endoscopy is not the ideal screening method due to its invasiveness, relatively high cost and above all large incidence of aforementioned risk factors in the general population. Despite high resolution endoscopy and virtual imaging techniques, detecting dysplasia in a long segment of Barrett’s esophagus remains challenging. Additionally, there is a low inter-observer agreement among pathologists in grading both low- and high-grade dysplasia, leading to over- and under-diagnosis^7,8^.

Mechanisms that drive EAC development remain poorly understood. The analysis of the transcriptomic landscape of EAC, HGD and NDB can provide further insights into molecular mechanisms involved in the development and progression of EAC. The study of RNA abundance profiles has proven its value to aid in the identification of new biomarkers to improve disease detection, therapeutic decision making, therapy response monitoring, and early relapse detection^9^.

Over the last decade, numerous studies have explored various types of RNA species in tissue biopsies from esophageal cancer patients. For instance, microRNAs (miRNAs) have been identified in tissue biopsies as potential biomarkers for EAC, HGD and NDB^10^. These miRNAs seem to have great potential as a diagnostic marker for Barrett’s esophagus in a population at risk (patients with GERD), but further research is required to identify miRNAs for risk stratification. To a lesser extent, messenger RNA (mRNA) expression has been studied in EAC, HGD and NDB as well^11,12^.

EAC is characterized by high mutation rates (including TP53 as a driver mutation that is most often found in tumor tissue^13^). Moreover, EAC as well as Barrett’s esophagus tissues are characterized by a large heterogeneity^14,15^. By gaining a deeper understanding in the different molecular subtypes, a more targeted treatment approach can be explored.

Besides gene dysregulation, chromosomal rearrangements can result in fusion proteins. Fusion genes have been reported to be involved in cancer^16^, including EAC^17–19^. Identification of fusion genes provides valuable insights in the development of EAC and can potentially be used as biomarkers for detection or therapeutic targeting.

Classically, these molecular profiling studies require the availability of (tumor) tissue that is not always readily available. The past decade, profiling of nucleic acids isolated from liquid biopsies (e.g. blood) for cancer biomarkers has gained increased interest, because this procedure is minimally invasive compared to tissue biopsies. For EAC, a number of studies have identified several miRNAs as putative biomarkers in serum or plasma^20,21^, but further clinical validation studies are needed prior to assessment of clinical utility. Circular RNA (circRNA) is an emerging new type of RNA that has gained interest in the field of cancer biomarker research. Due to their circular covalent structure, circRNAs are more resistant to degradation by exonucleases in the blood. Although the potential as cancer biomarker has been shown in several studies^22,23^, this has not yet been reported in either plasma or tissue from EAC patients.

Quantification of circulating mRNAs as a biomarker are much more challenging, due to their low concentration and fragmentation status in the blood. However, with the refinement of RNA sequencing methods, the detection of circulating mRNA is improving as well. In EAC these circulating mRNAs have not been identified yet, but have shown great potential in other cancer studies^24^.

In this study, we generated a comprehensive dataset that allows exploration of the complex transcriptome landscape of EAC and precursor lesions (HGD, NDB) in 17 patients. It includes polyA+ RNA (tissue samples), mRNA capture-based (plasma) and miRNA expression profiling (tissue and plasma). Exploratory data analysis was done to study protein coding gene mutations, fusion genes, and circRNAs.

## Methods

### Patient sample collection

**Fig. 1:**
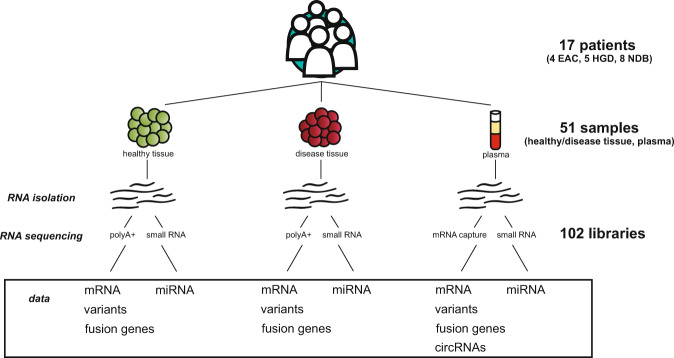
Experimental set-up and overview of the data. This comprehensive dataset includes 17 patients with EAC, HGD or NDB. From each patient disease tissue, healthy esophageal tissue and blood plasma was collected. From all 51 samples, RNA was isolated that was used for mRNA (polyA+ and capture-based) and small RNA sequencing. Data reported in this study includes data for mRNA and miRNA expression, variant analysis, fusion gene detection and circRNAs (the latter only in plasma samples).

Matching tissue and blood samples were obtained from four patients with esophageal adenocarcinoma (EAC), five patients with high-grade dysplasia (HGD) and eight patients with non-dysplastic Barrett’s esophagus (NDB) (Table 1). All samples were collected before treatment with informed consent (EC/2016–0495 and EC/2016–0496, Ghent University Hospital Ethics Committee). Tissue samples were obtained during endoscopy (NDB and HGD) or after surgical resection of the tumor (EAC). At least one of the tissue samples that was collected from the diseased tissue zone was sent for pathological investigation. The other disease tissue samples and healthy esopgahus tissue samples (collected from each patient) were preserved in RNAlater (Qiagen) at 4 °C and transferred to −80 °C the following day for long-term storage. Blood samples were collected in a 6 ml EDTA waste tube followed by a 9 ml sodium citrate (3.2%) VACUETTE blood tube (Greiner Bio-One). Plasma was prepared by centrifugation at 1,800 g for 10 min (full break and acceleration). The clear toplayer (leaving 0.5 cm above the buffy coat) was transferred to cryovials and stored at −80 °C. Time between blood collection and plasma preparation was less than 4 h, except for sample ID2 (6 h) and ID20 (7 h). Hemolysis was determined spectrophotometrically (absorbance at 414 nm) for all plasma samples using Nanodrop (ND1000, Thermo Scientific) (see Supplementary Table 1). RNA extraction, library preparation and sequencing of all samples was performed by Biogazelle (Zwijnaarde, Belgium) as discussed in the next sections. An experimental overview is shown in Fig. 1.

**Table 1:** Metadata of 17 patients included in this dataset.

clinical diagnosis	sample ID	age	gender	TNM^a^	Barrett’s segment^b^	location	follow-up time
EAC	ID20	74	M	pT2N1M0	C0M2	distal esophagus	44
EAC	ID29	77	M	pT1bN1M0	yes, CM not reported	GEJ	34
EAC	ID30	73	M	ypT1bN0M0	—	GEJ	36
EAC	ID43	63	M	pT1aN0M0	C4M5	NA (no resection)	10^D^
HGD	ID2	45	M	—	C10M12	—	29 (EAC)
HGD	ID5	78	M	—	C5M7	—	49
HGD	ID25	73	M	—	C10M10	—	23 (EAC)
HGD	ID26	54	M	—	C5M7	—	36
HGD	ID39	83	F	—	C0M3	—	37
NDB	ID1	59	M	—	C0M7	—	40^D^
NDB	ID18	59	F	—	C10M12	—	39 (LGD)
NDB	ID19	71	M	—	C11M12	—	43 (C11M11)
NDB	ID22	73	M	—	C6M6	—	20
NDB	ID33	51	M	—	C10M12	—	37 (C11M12)
NDB	ID35	78	F	—	C9M9	—	16 (C7M8)
NDB	ID37	45	M	—	C5M5	—	23 (C3M6)
NDB	ID40	76	M	—	C8M8	—	6

^a^Classification that describes the size of the primary tumor and invasion in surrounding tissue (T), lymph node involvement (N) and metastasis (M). The prefix p indicates histopathological staging of the resected tumor and y indicates that the patient received neoadjuvant therapy.

^b^The Prague C and M classification is used for reporting the Barrett’s segment: C = circumferential Barrett’s segment; M = maximal length of the Barrett’s tongue-like extent^62^.

EAC = esophageal adenocarcinoma, HGD = high-grade dysplasia, NDB = non-dysplastic Barrett’s esophagus, M = male, F = female, LGD = low-grade dysplasia, GEJ = gastro-esophageal junction. Follow-up time indicates time in months with the last known disease progression in brackets. D indicates the patient has died.

### RNA isolation from tissue and plasma samples

For all tissue samples, total RNA was isolated using the miRNeasy mini kit (Qiagen) with on-column DNase digestion, according to the manufacturer’s protocol. RNA concentration was measured with the Qubit 2.0 fluorometer (Thermo Fisher Scientific). The concentration ranged from 16.3 to 2,210 ng/µl, with sample ID43_EAC (disease tissue) having the lowest concentration (Supplementary Table 2). RNA integrity was determined using the Fragment Analyzer (Advanced Analytical Technologies). Most samples (70.6%) had quality scores above 7, the lowest score was 3.4 (disease tissue of sample ID43_EAC) (Supplementary Table 2). RNA was used for polyA+ RNA sequencing and small RNA sequencing.

For all plasma samples, RNA was isolated from 200 µl plasma using the miRNeasy Serum/Plasma Kit (Qiagen) according to the manufacturer’s instructions. For RNA used for mRNA capture sequencing, RNA isolation was followed by gDNA removal using the Heat&Run gDNA removal kit (ArcticZymes). Since extra-cellular RNA from plasma is highly fragmented and typically below the detection limit, the RNA concentration or integrity was not estimated. Instead, a volume based RNA input was used for library preparation.

### PolyA+ RNA sequencing for tissue samples

Libraries were prepared with the TruSeq Stranded mRNA Library Prep kit (Illumina), using 100 ng of RNA as input material. The quality and the size distribution of the libraries was validated on the Fragment Analyzer (Advanced Analytical Technologies) and quantification was done using the Qubit fluorometer (Life Technologies). Libraries were normalized and samples were pooled accordingly. Samples were paired-end sequenced with a read length of 2 × 75 base pairs (bp) on a NextSeq 500 (Illumina) instrument according to the manufacturer’s instructions.

### mRNA capture sequencing for plasma samples

Libraries were prepared with an optimized protocol of the TruSeq RNA Access Library Prep Kit (Illumina), using 8.5 µl of the RNA eluate. The Fragment Analyzer (Advanced Analytical Technologies) was used to validate size distribution and quality of the libraries and quantification was done using Qubit fluorometer (Life Technologies). Libraries were normalized and samples were pooled accordingly. Samples were paired-end sequenced with a read length of 2 × 75 bp on a NextSeq 500 (Illumina) instrument according to the manufacturer’s instructions. Sequencing was done in two runs for all samples to obtain sufficient sequencing depth. For sample ID37_NDB, reads from only one run have been included, since the first run contained an insufficient number of reads (less than 2,000) for this sample.

### Small RNA sequencing for tissue and plasma samples

Libraries were prepared using the NEBNext small RNA library prep kit (New England Biolabs) for both tissue and plasma samples. For tissue and for plasma, 100 ng and 6 µl of total RNA was used as input, respectively. Library size selection was done with the Pippin Prep system (Sage Science) to select the ~147–157 nt fragments containing mature miRNAs. Libraries were normalized based on qPCR quantification and pooled accordingly. Pools were concentrated with ethanol precipitation and quantification with the Qubit 2.0 fluorometer (Thermo Fisher Scientific). Tissue and plasma samples were single-end sequenced with a 75 bp read length on a NextSeq 500 (Illumina) instrument according to the manufacturer’s instructions.

### Data processing of mRNA sequencing data

Pre-processing of mRNA sequencing data of plasma and tissue samples included 3′-end trimming, adapter removal and filtering (discard reads smaller than 20 nt) using Cutadapt (v1.18). Low quality read pairs were removed using Biopython (v1.72) by keeping pairs with minimal 80% of their length having a Phred score greater or equal than 19. Clumpify (BBMap v38.26) was used for read duplicate removal for plasma samples only, due to the low RNA input. STAR (v2.6.0) was used for mapping (GRCh38 v91) and quantification was done with HTSeq (v0.11.0). Individual QC reports were generated with FastQC (v0.11.8) and multiQC (v1.8) was used to combine these reports for tissue and plasma samples. Annotation was based on GRCh38, UCSC Genome Browser (reference genome) and GENCODE v20, Ensembl 84 (reference transcriptome). The number of mapped reads remaining after the different pre-processing steps in tissue and plasma samples is shown in Table 2. The R packages edgeR (v3.28.1) and limma (v3.42.2) were used for normalization (Trimmed Mean of M-values) differential gene expression (tissue)/ abundance (plasma) analysis, respectively. Prior to these analyses, genes were filtered based on more than four counts in at least half of the samples per group (EAC, HGD, NDB). The Gene Set Enrichment Analysis (GSEA) tool (v4.1.0) was used to identify sets of genes that are significantly different between two groups^25^. As input for the analysis, a ranked list based on log2 fold change of all genes was used. For the purpose of this study, two collections of the Molecular Signatures Database (MSigDB) were used: the hallmark^26^ and the C2 chemical and genetic perturbations gene sets.

**Table 2:** Range and mean (±standard deviation) of the number of reads per sample during the different pre-processing steps for all mRNA (tissue and plasma) and miRNA (tissue and samples) samples.

	mRNA (incl. circRNA for plasma)			miRNA	
	*samples*	*range*	*mean ± s.d*.	*range*	*mean ± s.d*.
raw reads (million)	tissue healthy	25.7–30.5	27.7 ± 1.5	14.7–28.8	21.7 ± 3.7
	tissue disease	24.2–31.2	27.1 ± 1.8	19.1–26.2	22.5 ± 2.0
reads after trimming (million)	tissue healthy	20.8–25.6	23.1 ± 1.5	—	—
	tissue disease	16.7–25.7	21.9 ± 2.1	—	—
mapped reads (million)	tissue healthy	20.5–25.4	22.9 ± 1.5	2.0–11.7	6.0 ± 2.7
	tissue disease	14.5–25.4	21.5 ± 2.4	3.5–10.5	7.0 ± 1.9
raw reads (million)	plasma	22.9–34.1	29.1 ± 3.2	15.2–20.6	18.0 ± 1.3
reads after trimming (million)	plasma	13.3–29.7	23.5 ± 4.5	—	—
reads after deduplication (million)	plasma	1.0–6.0	3.3 ± 1.4	—	—
mapped reads (million)	plasma	0.9–5.8	3.2 ± 1.4	0.4–1.5	0.8 ± 0.3

### Data processing of small RNA sequencing data

Adapter trimming was applied to all small RNA sequencing reads of tissue and plasma samples, followed by mapping to the GRCh38 reference genome with Bowtie (v1.2.2). No mismatches were allowed for mapping reads smaller than 25 nucleotides, while for the longer reads a maximum of two mismatches were allowed. Annotation was based on Ensembl (v84), UCSC (hg38) and miRBase (v21). Mapped reads were annotated to mature miRNAs as well as other small RNAs, including tRNA, rRNA, sn(o)RNAs and piRNAs. Here, we only present the miRNA results. The number of mapped reads remaining after the different pre-processing steps in tissue and plasma samples is shown in Table 2. The R packages edgeR (v3.28.1) and limma (v3.42.2) were used for normalization (Trimmed Mean of M-values) and differential miRNA expression (tissue)/abundance (plasma) analysis, respectively. Prior to these analyses, genes were filtered based on more than four counts in at least half of the samples per group (EAC, HGD, NDB).

### Analysis of circRNAs in mRNA capture sequencing data

For plasma samples, raw mRNA capture sequencing reads were used to identify circRNAs based on back-splice junctions using CIRCexplorer2 (v2.3.3). Reads were trimmed with Cutadapt (v.1.18), low quality reads were removed with a custom script, retaining only reads where 80% of the read has a Phred quality score of at least 20 and duplicates were removed with Clumpify BBMap (v38.26). Mapping was done in 2 steps with TopHat2/TopHat-Fusion (v2.1.0) using indices of both Bowtie2 (v2.3.4.1) and Bowtie (v1.1.2) respectively. First, reads are aligned onto the genome and transcriptome using TopHat2 in order to reduce false positive reads aligned in the TopHat-Fusion alignment. BEDTools (v2.26.0) was used to convert BAM files to fastq files. The “parse”, “annotate”, “assemble” and “denovo” modules in CIRCexplorer2 were used according to the user’s manual^27^.

### Variant analysis of mRNA capture sequencing data

RNA sequencing data can be used for variant analysis, as previously demonstrated^28^. Using the RNA sequencing data from tissue and plasma samples, variants were identified using the following pipeline (based on Piskol *et al*.^29^): the first ten bases of all paired-end reads of each sample were trimmed due to possible false positives that can occur here as a result of random priming. The remaining sequence was aligned against the human reference genome build GRCh38 using STAR (v2.6.0c, two-step mode). Next, Mutect2 was used to call variants using default settings following the GATK (v3.8.0) best practices workflow, which included base-recalibration and duplicate removal with Picard (v.2.21.6)^30^. Variants located within four nucleotides of splice-junctions, in homopolymeric regions or regions overlapping other repeat types were removed. For each of the remaining variants, a BLAT (v3.5) analysis was performed to assess the quality of the reads contributing to the variant call^31^. This helped identify and filter out variants introduced by misaligned reads. Afterwards, variants were filtered differently depending on the tissue of origin. For healthy and tumor tissue samples, variants supported by at least 20 reads in total (DP > 20) and four reads for the alternative allele (AD > 4) were retained. In addition, variants found in more than one gnomAD^32^ (v3.1) sample or having allele frequencies below 20 or above 80 percent were removed in the tissue data. Next, variants identified in the healthy tissue were subtracted from the tumor variant list to obtain a list of tumor-specific variants. In a last phase, the disease-specific variant list was intersected with a list of variants in plasma. These results were filtered to only keep variants that have a coverage of at least two reads.

### Fusion gene analysis in polyA+ and mRNA capture sequencing data

Fusion gene analysis was done on all tissue (polyA+ sequencing data) and plasma samples (mRNA capture sequencing data). Adapter clipping and quality trimming from all sequencing reads was done using Trimmomatic (v0.35). After 3′ quality trimming, fusion genes were detected using a pipeline based on the FusionCatcher methodology (v0.99.7c). Mapping to the reference genome (Ensembl release 84) was performed with STAR (v2.5.1b) using the 2-pass mode and duplicates were removed with Picard tools (v2.7). This analysis results in a list of candidate fusion genes with the presumed breakpoint (“fusion junction”).

## Data Records

This dataset includes mRNA and small RNA sequencing data from four patients with EAC, five patients with HGD and eight patients with NDB. For each patient, RNA from matching tissue (healthy esophagus and disease) and plasma was sequenced, resulting in 102 sequenced libraries from 51 samples. Clinical information of the 17 patients is available in Table 1, including age at diagnosis, tumor stage and/or Barrett’s segment and follow-up information (if known). An overview of all available data and access information is provided in Table 3. For this publication, raw data was pre-processed using in-house optimized pipelines (Biogazelle and Ghent University), resulting in 119 expression profiles: 34 mRNA and 34 miRNA expression profiles from healthy and disease tissue samples, 17 mRNA and 17 miRNA expression profiles from plasma, and 17 circRNA expression profiles (based on mRNA sequencing data) from plasma. Count tables have been deposited in the ArrayExpress^33^ database at EMBL-EBI. In addition, results from variant- and fusion gene analysis are available as supplementary tables (Supplementary Tables 4, 5).

**Table 3:** Overview of available data and sources.

data	data type	samples	source	accession number or name
pre-processed data (count tables)	mRNA	tissue (healthy and disease, 34 samples)	ArrayExpress	*E-MTAB-10005*^63^
pre-processed data (count tables)	mRNA	plasma (17 samples)	ArrayExpress	*E-MTAB-10002*^64^
pre-processed data (count tables)	small RNA	tissue (healthy and disease, 34 samples)	ArrayExpress	*E-MTAB-10003*^65^
pre-processed data (count tables)	small RNA	plasma (17 samples)	ArrayExpress	*E-MTAB-10004*^66^
pre-processed data (count tables)	circRNA	plasma (17 samples)	ArrayExpress	*E-MTAB-10002*^64^
pre-processed data (count tables)	mRNA	tissue (healthy and disease, 34 samples)	R2	*Mixed Barretts Tissue de Preter - 34 - deseq. 2_rlog - hsens91*
pre-processed data (count tables)	mRNA	plasma (17 samples)	R2	*Mixed Barretts Plasma de Preter - 17 - deseq. 2_rlog - hsens91*
pre-processed data (count tables)	small RNA	tissue (healthy and disease, 34 samples)	R2	*Mixed Barretts Tissue de Preter - 34 - deseq. 2_rlog - kdpmir001*
pre-processed data (count tables)	small RNA	plasma (17 samples)	R2	*Mixed Barretts Plasma de Preter - 17 - deseq. 2_rlog - kdpmir001*
pre-processed data (count tables)	circRNA	plasma (17 samples)	R2	*Mixed Barretts Plasma (circRNA) de Preter - 17 - deseq. 2_rlog - circpret1*
results variant analysis	based on mRNA data	plasma	Supplementary Table 4	—
results fusion gene analysis	based on mRNA data	tissue	Supplementary Table 5	—
results fusion gene analysis	based on mRNA data	plasma	Supplementary Table 5	—

All pre-processed mRNA, miRNA and circRNA expression data for tissue and plasma samples was also uploaded to the R2 Genomics Analysis and Visualization Platform (http://r2.amc.nl), an online genomics data visualization tool. The user-friendly web application allows rapid and easy visualization of the data, including gene expression analysis, gene correlation analysis and visualization of one or multiple genes.

All raw sequencing data (polyA+, mRNA capture, small RNA) is available through the European genome-phenome archive (EGA) under accession number EGAS00001004939^34^. Data requests can be made by contacting the Data Access Committee, as stated on the EGA information page of the study (https://ega-archive.org/studies/EGAS00001004939). A Data Transfer Agreement (DTA) and Data Access Agreement (DAA) will have to be signed in order for the data to be transferred (a template can be found in Supplementary File 1). The raw sequencing data available at EGA were not part of the peer-reviewed content of this manuscript.

## Technical Validation

### Assessment of RNA sequencing quality

#### mRNA sequencing quality

**Fig. 2:**
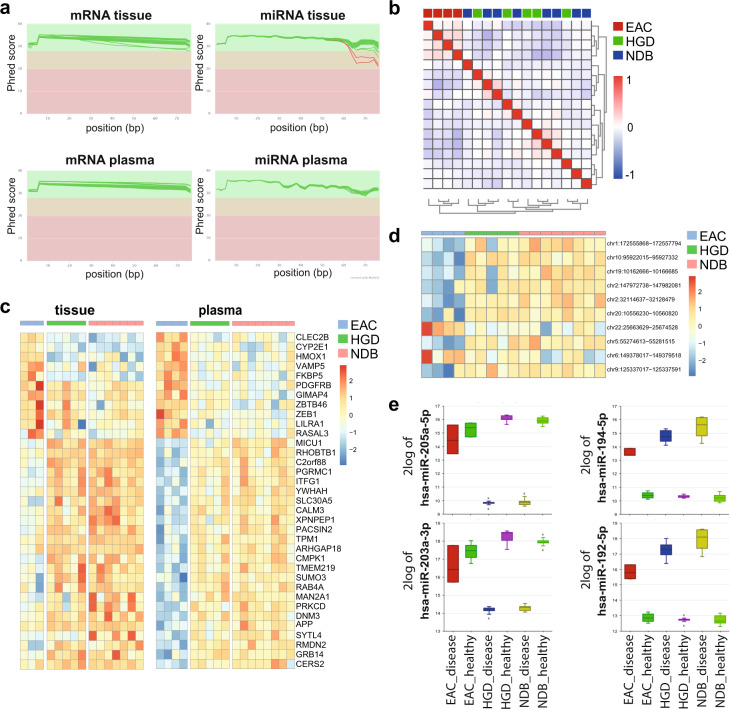
Technical validation of the data. (**a**) quality plots of the RNA raw reads sequencing data: per base mean quality of mRNA tissue and plasma data (top row), and miRNA tissue and plasma data (bottom row); (**b**) hierarchical clustering of the mRNA plasma samples based on Pearson’s correlation coefficient, generated in R2 (Euclidian distances, average linkage), where the R-value ranging from −1 to 1 represents the negative (−1), positive (1) or no (0) relationship. It shows a clustering of EAC samples versus HGD and NDB samples; (**c**) heatmap showing the relative expression of 35 overlapping differentially expressed genes (up and down) for tissue (left) and plasma (right) samples (Benjamini-Hochberg adjusted p-value < 0.05); (**d**) the relative expression of top ten abundant circRNAs in plasma (EAC vs NDB) shown in a heatmap (p-value < 2.36 × 10^−3^); (**e**) boxplot representation of the relative expression of four of the most frequently reported up- and down regulated miRNAs (more than four times in literature) in EAC, HGD and/or NDB tissue samples compared to matched healthy esophageal tissue. Samples included in the boxplots are healthy and disease tissues from 3 patients with EAC, 5 with HGD and 7 with NDB.

The mean sequencing quality per base (raw data) for mRNA tissue and plasma is higher than 28 for all samples (Fig. 2a), reflecting the very good quality of the data. The average number of reads for mRNA tissue and plasma samples throughout the pre-processing steps is shown in Table 2. For all tissue samples, 19–25 million reads per sample remain after trimming and filtering, except for sample ID40_NDB (disease tissue) that has a slightly lower number of reads (14.5 million). For the plasma samples, on average 3.2 million reads remain after filtering, trimming and deduplication.

For further downstream analyses, sample ID40_NDB was excluded due to the lower library yield (measured as described above) of the disease tissue sample (data not shown) and the lower percentage (68%) of reads with a quality score higher than 30, compared to all other tissue samples (85% on average). Sample ID43_EAC was also excluded for downstream analyses, due to the lower library quality of the disease tissue sample. This was likely due to the low concentration (16.3 ng/µl) and low RNA quality score (3.4) (Supplementary Table 2).

#### small RNA sequencing quality

The mean sequencing quality per base (raw data) of the small RNA sequencing data (tissue and plasma) is higher than 28 for the first 60 bp in all samples (Fig. 2a), reflecting the very good quality of the data. The sequencing quality for samples ID26_HGD (healthy tissue) and ID19_NDB (disease tissue) decreases slightly towards the end of the reads (>60 bp). However, as most small RNAs are typically around 20–30 nucleotides in length, a good quality measure for the first 30 nucleotides of the 5′-end of the read is more relevant in the context of small RNA expression analysis. The number of remaining miRNA reads per sample after pre-processing is 5–10 million reads for tissue samples and 1–3 million for plasma samples (Table 2).

### Successful detection of thousands of RNA genes in tissue and plasma

Expressed mRNAs, miRNAs and circRNAs have been identified in all tissue and/or plasma samples (Table 4). As expected, fewer unique mRNAs and miRNAs were found in plasma compared to tissue samples. In EAC samples, fewer unique circRNAs were found (353-1,165) compared to HGD (858-3,624) and NDB (1,237-3,683).

**Table 4:** Range and mean (±standard deviation) of unique protein coding genes (mRNAs), miRNAs and circRNAs found in tissue or plasma samples.

RNA type	disease	sample type	range	mean ± s.d.
mRNA	EAC	healthy tissue	17,297–18,844	18,122 ± 552
		disease tissue	15,374–19,291	17,990 ± 1,534
		plasma	8,195–10,237	8,968 ± 763
	HGD	healthy tissue	17,578–18,119	17,834 ± 220
		disease tissue	18,055–19,817	18,893 ± 688
		plasma	8,974–11,468	10,707 ± 886
	NDB	healthy tissue	16,848–17,937	17,503 ± 338
		disease tissue	16,294–19,685	18,282 ± 909
		plasma	9,514–11,443	10,455 ± 633
miRNA	EAC	healthy tissue	483–639	529 ± 64
		disease tissue	629–682	657 ± 20
		plasma	375–438	417 ± 25
	HGD	healthy tissue	494–726	598 ± 81
		disease tissue	577–704	659 ± 44
		plasma	347–427	386 ± 28
	NDB	healthy tissue	531–682	626 ± 54
		disease tissue	621–714	663 ± 32
		plasma	332–432	391 ± 30
circRNA	EAC	plasma	353–1,165	745 ± 301
	HGD	plasma	858–3,624	2,286 ± 895
	NDB	plasma	1,237–3,683	2,000 ± 824

Counts were filtered by only keeping RNAs with more than four counts.

### Validation of mRNA abundance data

#### mRNA in tissue

Several studies have reported lists of differentially expressed genes in EAC, HGD and NDB compared to healthy tissue samples^11,12,35^. However, the overlap among these reported genes is limited. Tables 5 and 6 show the overlap of differentially expressed genes (adjusted p-value < 0.05) between EAC and healthy tissue from three large studies^11,12,35^ and our own dataset. A significant overlap (Fisher’s exact test; Benjamini-Hochberg adjusted p-value < 0.05) was observed between the differentially expressed genes reported in this study and the three published gene sets.

**Table 5:** Number of overlapping upregulated genes in EAC tissue compared to healthy tissue.

	Maag *et al*.^11^	Lv *et al*.^12^	Wang *et al*.^35^	tissue data from this study (including all 34 samples)
**Maag** ***et al****.*^11^	19			
**Lv** ***et al****.*^12^	0 (1)	63		
**Wang** ***et al****.*^35^	0 (1)	10 (9.54 × 10^−12^)	119	
**tissue data from this manuscript** (including all 34 samples)	19 (1.32 × 10^−15^)	12 (2.48 × 10^−08^)	20 (9.29 × 10^−12^)	446

On the diagonal line are the number of reported genes in each gene set. The number of overlapping genes between a given pair of datasets are shown, with Fisher’s exact test adjusted p-values (Benjamini-Hochberg).

**Table 6:** Number of overlapping downregulated genes in EAC tissue compared to healthy tissue.

	Lv *et al*.^12^	Wang *et al*.^35^	tissue data from this manuscript (including all 34 samples)
**Lv** ***et al****.*^12^	57		
**Wang** ***et al****.*^35^	5 (3.27 × 10^−05^)	100	
**tissue data from this manuscript** (including all 34 samples)	2 (0.01)	3 (4.70 × 10^−03^)	57

On the diagonal line are the number of reported genes in each gene set. The number of overlapping genes between a given pair of datasets are shown, with Fisher’s exact test adjusted p-values (Benjamini-Hochberg).

GSEA in tissue revealed several interesting gene sets that are enriched in disease tissue (EAC or NDB) compared to healthy tissue, and EAC compared to NDB tissue (Supplementary Table 3). For example, comparing EAC tissue with healthy tissue the following relevant gene sets were significantly (FDR < 1%) positively enriched in EAC: HALLMARK_EPITHELIAL_MESENCHYMAL_TRANSITION, HALLMARK_KRAS_SIGNALING_UP and WANG_ESOPHAGUS_CANCER_VS_NORMAL_UP^35^. Comparing EAC with NDB tissue samples, the WANG_BARRETTS_ESOPHAGUS_UP^35^ gene set was significantly negatively enriched in EAC (FDR < 1%). These GSEA results (FDR < 25%) are available in Supplementary Table 3.

#### mRNA in plasma

There are currently no studies reporting on mRNAs in plasma of patients with EAC, HGD or NDB. Using the sample clustering option in R2 for the plasma mRNA expression level data, a clear clustering of the samples according to sample identity, i.e. EAC samples versus HGD and NDB samples (Fig. 2b) is observed. If we look into more detail we observe that some of the differentially expressed mRNAs in tissue of patients with EAC compared to NDB are also differentially abundant in the plasma samples (in the same direction). More specifically, there is an overlap of 11 up- and 24 downregulated genes, as shown in the heatmap in Fig. 2c.

When comparing EAC with NDB plasma, several relevant gene sets showed positive enrichment in EAC, including HALLMARK_MYC_TARGETS V1 and V2 (FDR < 1%). Deregulation of MYC is known to play a key role in the development of EAC^36,37^, indicating that tumor signal may be present in plasma. These GSEA results (FDR < 25%) are available in Supplementary Table 3.

Markers for epithelial mesenchymal transition (EMT) are of clinical relevance for a more targeted treatment^38^. The process of EMT enables cancer cells to enter the blood stream and form local and distant metastasis^39^. Several EMT markers have been identified in EAC as well as in precursor lesions (NDB)^40,41^, suggesting that this process could be an early event for progression to EAC. Importantly, ZEB1 is a gene involved in EMT^42,43^ and in this data it was found to be significantly higher in EAC compared to NDB in both tissue and plasma (Benjamini-Hochberg adjusted p-values are 2.62 × 10^−2^ and 3.01 × 10^−2^, respectively).

#### circRNA in plasma

Like mRNA, circRNAs have also not yet been reported in plasma from patients with EAC, HGD or NDB. In our analyses, no significantly differentially expressed circRNAs were identified (Table 7). While the adjusted p-values (Benjamini-Hochberg) are not significant in this dataset, a heatmap of the top ten most abundant circRNAs (p-values are below 2.36 × 10^−3^) comparing EAC with NDB samples shows that plasma circRNAs may have biomarker potential, but needs further validation (Fig. 2d).

**Table 7:** Results of expression and abundance analyses of tissue samples (19,734 genes and 676 miRNAs included) and plasma samples (11,255 genes, 457 miRNAs and 2,275 circRNAs included).

contrasts	1. disease vs healthy tissue			2. disease tissue vs disease tissue			3. disease-healthy vs disease-healthy			4. plasma		
	EAC	HGD	NDB	EAC vs NDB	EAC vs HGD	HGD vs NDB	EAC vs NDB	EAC vs HGD	HGD vs NDB	EAC vs NDB	EAC vs HGD	HGD vs NDB
**mRNA (up/down)**	99/5	4,440/4,218	4,799/4,324	3,653/2,615	2,798/1,956	2/8	1,979/1,172	1,665/734	0/0	54/167	0/0	0/0
**miRNA (up/down)**	42/42	203/154	219/186	56/38	15/5	0/0	46/62	27/21	0/0	0/0	0/0	0/0
**circRNA (up/down)**	—	—	—	—	—	—	—	—	—	0/0	0/0	0/0

Prior to the analyses, count tables were filtered to include RNAs with more than four counts in at least half of the samples per group. Results shown in the table are filtered based on adjusted p-value < 0.05 (Benjamini-Hochberg) and LFC > log2(1.5). Different contrasts were analyzed: comparing disease with healthy tissue (contrast 1), comparing disease tissue between groups (contrast 2), comparing disease versus healthy tissue samples of one group with the disease versus healthy tissue samples of another group (contrast 3), and comparing the three groups for the plasma samples (contrast 4).

### Validation of miRNA abundance data

#### miRNA in tissue

Many miRNAs have been reported to be up- or downregulated in EAC, HGD and NDB tissue compared to healthy tissue samples^44^. Two of the most reported miRNAs to be upregulated in EAC, HGD, and/or NDB compared to healthy tissue are hsa-miR-192-5p^45–50^ and hsa-miR-194-5p^45–47,49–51^. Similarly, two of the most reported downregulated miRNAs in EAC, HGD, and/or NDB are hsa-miR-203a-3p^46–51^ and hsa-miR-205-5p^45–47,50–52^. The latter miRNA (hsa-miR-205-5p) is known for targeting ZEB1^38^. In our dataset, we confirm the differential expression patterns of these miRNAs in disease tissue compared to healthy tissue (Fig. 2e).

#### miRNA in plasma

While several differential miRNA abundance patterns in EAC or NDB plasma have been reported^21,53–58^, there is only one overlapping miRNA (miR-194-5p) among these studies^54,56^. Moreover, different blood fractions, including serum^21,55–59^, plasma^54^ and extracellular vesicles^53^ were studied. With our analysis pipeline, no differentially abundant miRNAs between the plasma samples of the different groups were identified (Table 7).

## Usage Notes

### Gene expression and abundance analysis

Differential gene expression and abundance analyses were performed for mRNAs, miRNAs and circRNAs in tissue and plasma. The number of differentially expressed genes are depicted in Table 7. The pre-processed data is also uploaded in R2, allowing further exploration and visualization of the dataset. In this study, we have identified several circRNAs in plasma of patients with EAC, HGD and NDB. This type of RNA has great potential as circulating biomarker because they are more resistant to RNA degradation by exonucleases due to their circular structure. While we focused on miRNA expression and abundance analyses using the small RNA sequencing data, other small RNAs such as tRNA (fragments), and piRNAs could be analyzed using our data as well.

### Expression of related miRNAs and mRNAs

**Fig. 3:**
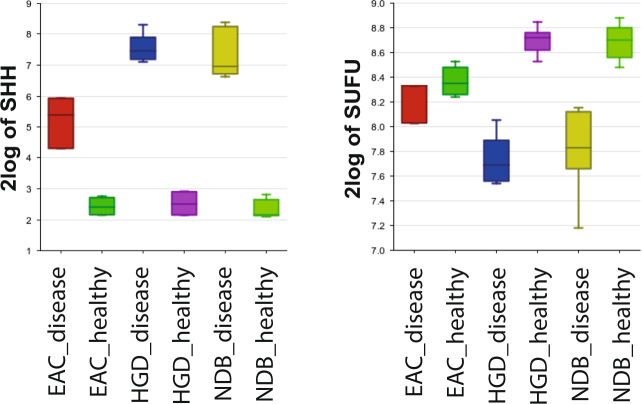
Usage notes. Boxplot per sample group of the hsa-miR-194, SHH and SUFU expression levels in the tissue samples (generated in R2). Samples included in the boxplots are healthy and disease tissues from 3 patients with EAC, 5 with HGD and 7 with NDB.

One of the unique features of our dataset is the inclusion of both miRNA and mRNA data of matching disease and healthy tissue samples. The relationship between miRNA and mRNA expression can thus be studied in our data. As an example, the hedgehog (HH) signaling pathway is known to play an important role in EAC and NDB^60^. In NDB, increased expression of hsa-miR-194 results in a loss of SUFU, which leads to an upregulation of the Sonic Hegdgehog (SHH) gene. The upregulation of hsa-miR-194 and SHH, and downregulation of SUFU compared to healthy tissue is also observed in our NDB tissue data as well as in the EAC and HGD tissue samples (Figs. 2e and 3). These unique matched disease and healthy fractions dataset allows further exploration of potentially relevant pathways, i.e. by using both miRNA and mRNA data, as demonstrated by this example.

### Mutation analysis

Based on the polyA+ sequencing data (tissue) and mRNA capture sequencing data (plasma), mutation analysis was performed. For each patient, disease specific variants were identified using strict filtering as described in the methods section. Subsequently, these variants were intersected with variants in plasma. In total, 24 variants were identified in the plasma of two EAC patients, five HGD patients and four NDB patients (Supplementary Table 4). Per patient, 1-7 variants were found, but no overlap was observed within a disease group or between groups. Three variants are known tumor mutations according to the COSMIC database in prostate cancer (COSM5564582), cervix or biliary tract cancer (COSM5493837), or large intestine cancer (COSM5756079). These results are a proof-of-concept to demonstrate the ability to identify likely somatic mutations or disease-specific RNA-editing events in plasma RNA sequencing data.

### Fusion gene analysis

Fusion gene analysis in EAC tissue has been reported in only a few studies^17–19^. Here, we demonstrate the potential of detecting fusion genes for EAC, HGD and NDB tissue and plasma samples. Results obtained from these analyses are provided in Supplementary Table 5. Results in this table are unfiltered, but in red are the fusion genes that have a high probability of being a false positive. In tissue samples, potential fusion genes were identified in all samples. By excluding (on a per sample basis) fusion genes also found in the healthy tissue, disease-specific fusion genes were identified. As a result, for all samples 2-14 fusion genes remain (excluding the potential false positives). For the plasma samples, potential fusion genes are identified in one HGD patient sample and in two NDB patient samples, with two overlapping fusion genes (ID5_HGD and ID19_NDB). No overlapping fusion gene between disease tissue and plasma samples was observed. Further validation of these potentially relevant fusion genes is required.

## Supplementary information

Supplementary Table 1

Supplementary Table 2

Supplementary Table 3

Supplementary Table 4

Supplementary Table 5

Supplementary File 1

## Data Availability

All code used for pre-processing mRNA and miRNA sequencing data is publicly available on GitHub (https://github.com/OncoRNALab/exRNAQC/blob/main/Preprocessing)^61^. For circRNA detection, the CircExplorer2 manual was followed as described in the Methods section. Further downstream analyses (differential expression, GSEA, fusion gene detection, and variant analysis) was done following the guidelines of the different R packages and software tools as described (with the used versions) in the Methods section.
