# Supplementary material for: Comprehensive RNA dataset of tissue and plasma from patients with esophageal cancer or precursor lesions
Source: Sci Data. 2022 Mar 14;9:86. doi: 10.1038/s41597-022-01176-x (PMC8921197; doi:10.1038/s41597-022-01176-x)
Supplement: Supplementary file 1 — Supplementary Table 1 [file 41597_2022_1176_MOESM1_ESM.pdf]

Supplementary Table 1: Hemolysis measurement results of all plasma samples

sample ID	disease	absorbance at 414 nm (ng/µl)
ID20	EAC	0.263
ID29	EAC	0.085
ID30	EAC	0.16
ID43	EAC	0.202
ID2	HGD	1.68
ID5	HGD	0.113
ID25	HGD	0.039
ID26	HGD	0.057
ID39	HGD	0.049
ID1	NDB	1,979
ID18	NDB	0.04
ID19	NDB	0.183
ID22	NDB	0.108
ID33	NDB	0.117
ID35	NDB	0.112
ID37	NDB	0.518
ID40	NDB	0.01
