# Supplementary material for: Comprehensive RNA dataset of tissue and plasma from patients with esophageal cancer or precursor lesions
Source: Sci Data. 2022 Mar 14;9:86. doi: 10.1038/s41597-022-01176-x (PMC8921197; doi:10.1038/s41597-022-01176-x)
Supplement: Supplementary file 2 — Supplementary Table 2 [file 41597_2022_1176_MOESM2_ESM.pdf]

Supplementary Table 2: RNA quality and concentration measurement results of all tissue samples

sample ID	tissue type	disease	RNA quality score	concentration (ng/µl)
ID20	disease	EAC	7.7	70.4
ID20	healthy	EAC	9.7	77.6
ID29	disease	EAC	7.9	438
ID29	healthy	EAC	9.6	1560
ID30	disease	EAC	7.9	110
ID30	healthy	EAC	9.1	27.6
ID43	disease	EAC	3.4	16.3
ID43	healthy	EAC	9.3	135.6
ID2	disease	HGD	7.4	200
ID2	healthy	HGD	9.9	172
ID5	disease	HGD	9.7	156
ID5	healthy	HGD	9.5	23
ID25	disease	HGD	8.7	212
ID25	healthy	HGD	9.7	228
ID26	disease	HGD	9.5	2210
ID26	healthy	HGD	9.6	94.4
ID39	disease	HGD	8.7	560
ID39	healthy	HGD	9.4	30
ID1	disease	NDB	6.1	166
ID1	healthy	NDB	9.5	183.6
ID18	disease	NDB	5.1	270.6
ID18	healthy	NDB	9.9	263.4
ID19	disease	NDB	8.7	317.6
ID19	healthy	NDB	9.8	125.6
ID22	disease	NDB	8.8	285
ID22	healthy	NDB	9.8	122
ID33	disease	NDB	5.7	138
ID33	healthy	NDB	9.4	100
ID35	disease	NDB	8.6	167.6
ID35	healthy	NDB	9.9	300
ID37	disease	NDB	6.8	124
ID37	healthy	NDB	7.6	236
ID40	disease	NDB	9.7	480
ID40	healthy	NDB	9.1	399.2
