# Supplementary material for: Comprehensive RNA dataset of tissue and plasma from patients with esophageal cancer or precursor lesions
Source: Sci Data. 2022 Mar 14;9:86. doi: 10.1038/s41597-022-01176-x (PMC8921197; doi:10.1038/s41597-022-01176-x)
Supplement: Supplementary file 3 — Supplementary Table 3 [file 41597_2022_1176_MOESM3_ESM.pdf]

Supplementary Table 3a: GSEA results of NDB vs healthy tissue samples. MSigDB Collection: hallmark gene sets.

na_pos (FDR <25%)

	GS<br>follow link to MSigDB	GS DETAILS	SIZE	ES	NES	NOM p-val	FDR q-val	FWER p-val	RANK AT MAX	LEADING EDGE
1	HALLMARK_PANCREAS_BETA_CELLS	Details ...	31	0.85	2.16	0	0	0	1053	tags=58%, list=6%, signal=62%
2	HALLMARK_EPITHELIAL_MESENCHYMAL_TRANSITION	Details ...	196	0.57	1.9	0	0	0	2890	tags=52%, list=17%, signal=62%
3	HALLMARK_COAGULATION	Details ...	113	0.53	1.69	0	0.006	0.017	2560	tags=42%, list=15%, signal=50%
4	HALLMARK_KRAS_SIGNALING_UP	Details ...	191	0.5	1.66	0	0.005	0.022	3304	tags=48%, list=20%, signal=59%
5	HALLMARK_INTERFERON_ALPHA_RESPONSE	Details ...	97	0.48	1.5	0.006	0.041	0.198	5040	tags=56%, list=30%, signal=79%
6	HALLMARK_ANGIOGENESIS	Details ...	32	0.58	1.48	0.032	0.046	0.25	3424	tags=63%, list=20%, signal=78%
7	HALLMARK_XENOBIOTIC_METABOLISM	Details ...	178	0.44	1.45	0.004	0.055	0.331	3025	tags=32%, list=18%, signal=39%
8	HALLMARK_UV_RESPONSE_DN	Details ...	143	0.44	1.42	0.014	0.061	0.4	2584	tags=30%, list=15%, signal=35%
9	HALLMARK_COMPLEMENT	Details ...	189	0.4	1.33	0.014	0.141	0.74	3062	tags=31%, list=18%, signal=38%
#	HALLMARK_HEDGEHOG_SIGNALING	Details ...	34	0.51	1.33	0.104	0.135	0.766	2194	tags=32%, list=13%, signal=37%
#	HALLMARK_MYOGENESIS	Details ...	170	0.39	1.3	0.039	0.167	0.861	2861	tags=36%, list=17%, signal=43%
#	HALLMARK_BILE_ACID_METABOLISM	Details ...	99	0.41	1.26	0.08	0.217	0.937	4277	tags=46%, list=26%, signal=62%
#	HALLMARK_OXIDATIVE_PHOSPHORYLATION	Details ...	198	0.37	1.23	0.067	0.243	0.965	6848	tags=61%, list=41%, signal=101%
#	HALLMARK_IL6_JAK_STAT3_SIGNALING	Details ...	83	0.41	1.22	0.131	0.25	0.977	3232	tags=28%, list=19%, signal=34%

na_neg (FDR <25%)

	GS<br>follow link to MSigDB	GS DETAILS	SIZE	ES	NES	NOM p-val	FDR q-val	FWER p-val	RANK AT MAX	LEADING EDGE
1	HALLMARK_KRAS_SIGNALING_DN	Details ...	157	-0.45	-1.59	0	0.04	0.036	1404	tags=26%, list=8%, signal=28%
2	HALLMARK_P53_PATHWAY	Details ...	198	-0.43	-1.56	0	0.032	0.058	2464	tags=25%, list=15%, signal=29%
3	HALLMARK_APICAL_SURFACE	Details ...	42	-0.48	-1.4	0.059	0.11	0.256	1889	tags=31%, list=11%, signal=35%
4	HALLMARK_E2F_TARGETS	Details ...	200	-0.37	-1.38	0.011	0.104	0.311	7043	tags=61%, list=42%, signal=104%
5	HALLMARK_WNT_BETA_CATENIN_SIGNALING	Details ...	41	-0.44	-1.25	0.141	0.244	0.659	3029	tags=37%, list=18%, signal=45%
6	HALLMARK_G2M_CHECKPOINT	Details ...	199	-0.33	-1.23	0.038	0.242	0.724	6364	tags=56%, list=38%, signal=89%
7	HALLMARK_PI3K_AKT_MTOR_SIGNALING	Details ...	100	-0.36	-1.2	0.098	0.275	0.831	2885	tags=19%, list=17%, signal=23%
8	HALLMARK_TNFA_SIGNALING_VIA_NFKB	Details ...	199	-0.32	-1.17	0.083	0.299	0.897	3264	tags=33%, list=20%, signal=41%
9	HALLMARK_ESTROGEN_RESPONSE_EARLY	Details ...	198	-0.31	-1.15	0.106	0.317	0.929	1362	tags=20%, list=8%, signal=22%
#	HALLMARK_MYC_TARGETS_V1	Details ...	200	-0.3	-1.1	0.189	0.406	0.978	7088	tags=51%, list=42%, signal=88%
#	HALLMARK_MTORC1_SIGNALING	Details ...	200	-0.28	-1.02	0.389	0.638	0.999	5177	tags=41%, list=31%, signal=59%
#	HALLMARK_MITOTIC_SPINDLE	Details ...	198	-0.28	-1.01	0.448	0.642	1	4074	tags=28%, list=24%, signal=37%

#	HALLMARK_HYPOXIA	Details ...	189	-0.25	-0.91	0.738	0.97	1	1906	tags=19%, list=11%, signal=21%
#	HALLMARK_APOPTOSIS	Details ...	159	-0.24	-0.86	0.881	1	1	2904	tags=20%, list=17%, signal=24%
#	HALLMARK_NOTCH_SIGNALING	Details ...	31	-0.31	-0.84	0.741	1	1	2360	tags=29%, list=14%, signal=34%
#	HALLMARK_MYC_TARGETS_V2	Details ...	58	-0.22	-0.67	0.972	1	1	5816	tags=50%, list=35%, signal=76%
#	HALLMARK_UNFOLDED_PROTEIN_RESPONSE	Details ...	112	-0.17	-0.58	1	1	1	3438	tags=14%, list=21%, signal=18%
#	HALLMARK_REACTIVE_OXYGEN_SPECIES_PATHWAY	Details ...	48	-0.19	-0.57	0.997	0.998	1	4200	tags=31%, list=25%, signal=42%

Supplementary Table 3b: GSEA results of NDB vs healthy tissue samples. MSigDB Collection: C2 chemical and genetic perturbations (cgp) gene sets (showing max. 50 results).

na_pos										
	GS follow link to MSigDB	GS DETAILS	SIZE	ES	NES	NOM p-val	FDR q-val	FWER p-val	RANK AT MAX	LEADING EDGE
1	VECCHI_GASTRIC_CANCER_ADVANCED_VS_EARLY_DN	Details ...	139	0.79	2.56	0	0	0	1787	tags=52%, list=11%, signal=58%
2	SABATES_COLORECTAL_ADENOMA_DN	Details ...	263	0.67	2.29	0	0	0	2201	tags=56%, list=13%, signal=63%
3	SERVITJA_ISLET_HNF1A_TARGETS_DN	Details ...	91	0.71	2.19	0	0	0	1670	tags=48%, list=10%, signal=53%
4	HSIAO_LIVER_SPECIFIC_GENES	Details ...	185	0.66	2.18	0	0	0	2414	tags=46%, list=14%, signal=53%
5	WANG_ESOPHAGUS_CANCER_VS_NORMAL_UP	Details ...	119	0.69	2.16	0	0	0	3264	tags=61%, list=20%, signal=76%
6	WANG_BARRETTS_ESOPHAGUS_UP	Details ...	49	0.78	2.15	0	0	0	1868	tags=63%, list=11%, signal=71%
7	SMID_BREAST_CANCER_RELAPSE_IN_BONE_UP	Details ...	88	0.7	2.11	0	0	0	2069	tags=51%, list=12%, signal=58%
8	MEBARKI_HCC_PROGENITOR_FZD8CRD_DN	Details ...	358	0.6	2.1	0	0	0	2776	tags=46%, list=17%, signal=54%
9	KANG_GLIS3_TARGETS	Details ...	27	0.84	2.1	0	0	0	1901	tags=70%, list=11%, signal=79%
10	WANG_BARRETTS_ESOPHAGUS_AND_ESOPHAGUS_CANCER_UP	Details ...	26	0.83	2.08	0	0	0	1939	tags=69%, list=12%, signal=78%
11	ANASTASSIOU_MULTICANCER_INVASIVENESS_SIGNATURE	Details ...	61	0.73	2.07	0	0	0	2694	tags=69%, list=16%, signal=82%
12	RICKMAN_HEAD_AND_NECK_CANCER_D	Details ...	26	0.85	2.05	0	0	0	1010	tags=62%, list=6%, signal=65%
13	LIU_CDX2_TARGETS_UP	Details ...	33	0.8	2.04	0	0	0	300	tags=42%, list=2%, signal=43%
14	BOQUEST_STEM_CELL_UP	Details ...	252	0.6	2.04	0	0	0	2574	tags=52%, list=15%, signal=61%
15	ONDER_CDH1_TARGETS_2_UP	Details ...	249	0.59	2.03	0	0	0.001	2740	tags=49%, list=16%, signal=58%
16	RODWELL_AGING_KIDNEY_NO_BLOOD_UP	Details ...	211	0.59	2.01	0	0	0.002	2884	tags=45%, list=17%, signal=54%
17	BLANCO_MELO_RESPIRATORY_SYNCYTIAL_VIRUS_INFECTION_A594_CELLS_DN	Details ...	109	0.63	2	0	0	0.004	1684	tags=37%, list=10%, signal=41%
18	BLANCO_MELO_COVID19_SARS_COV_2_INFECTION_CALU3_CELLS_DN	Details ...	22	0.83	2	0	0	0.004	546	tags=36%, list=3%, signal=38%
19	VECCHI_GASTRIC_CANCER_EARLY_DN	Details ...	327	0.57	1.99	0	0	0.006	2350	tags=45%, list=14%, signal=52%
20	WU_SILENCED_BY_METHYLATION_IN_BLADDER_CANCER	Details ...	50	0.72	1.99	0	0	0.006	2609	tags=54%, list=16%, signal=64%
21	OISHI_CHOLANGIOMA_STEM_CELL_LIKE_DN		271	0.57	1.97	0	0	0.009	2526	tags=44%, list=15%, signal=50%
22	PICCALUGA_ANGIOIMMUNOBLASTIC_LYMPHOMA_UP		208	0.58	1.96	0	0.001	0.011	2949	tags=54%, list=18%, signal=65%
23	WAMUNYOKOLI_OVARIAN_CANCER_GRADES_1_2_UP		139	0.61	1.96	0	0.001	0.013	3739	tags=49%, list=22%, signal=62%
24	NAKAYAMA_SOFT_TISSUE_TUMORS_PCA2_DN		75	0.65	1.95	0	0.001	0.015	2359	tags=59%, list=14%, signal=68%
25	ANDERSEN_LIVER_CANCER_KRT19_DN		60	0.68	1.94	0	0.001	0.019	1511	tags=32%, list=9%, signal=35%
26	WONG_ENDMETRIUM_CANCER_DN		69	0.66	1.94	0	0.001	0.02	2168	tags=55%, list=13%, signal=63%
27	BLANCO_MELO_COVID19_SARS_COV_2_INFECTION_A594_CELLS_DN		69	0.65	1.92	0	0.001	0.023	1468	tags=39%, list=9%, signal=43%
28	NAKAMURA_ADIPOGENESIS_EARLY_DN		35	0.73	1.91	0	0.001	0.033	2761	tags=54%, list=17%, signal=65%

29	KIM_GLIS2_TARGETS_UP		85	0.63	1.9	0	0.002	0.042	3413	tags=55%, list=20%, signal=69%
30	SMID_BREAST_CANCER_RELAPSE_IN_LUNG_DN		34	0.73	1.89	0	0.002	0.056	1988	tags=65%, list=12%, signal=73%
31	SMID_BREAST_CANCER_LUMINAL_A_UP		80	0.63	1.88	0	0.002	0.062	2465	tags=49%, list=15%, signal=57%
32	ONDER_CDH1_SIGNALING_VIA_CTNNB1		82	0.62	1.88	0	0.002	0.075	1702	tags=43%, list=10%, signal=47%
33	SMID_BREAST_CANCER_LUMINAL_B_UP		151	0.57	1.87	0	0.003	0.081	1819	tags=33%, list=11%, signal=37%
34	SERVITJA_LIVER_HNF1A_TARGETS_DN		119	0.59	1.86	0	0.003	0.095	1670	tags=31%, list=10%, signal=34%
35	SANSOM_APC_TARGETS_DN		329	0.53	1.85	0	0.003	0.109	3578	tags=40%, list=21%, signal=50%
36	WESTON_VEGFA_TARGETS_6HR		43	0.68	1.84	0	0.004	0.139	2173	tags=53%, list=13%, signal=61%
37	RODRIGUES_NTN1_TARGETS_DN		156	0.57	1.84	0	0.005	0.15	2174	tags=34%, list=13%, signal=39%
38	WATANABE_COLON_CANCER_MSI_VS_MSS_DN		62	0.62	1.84	0	0.005	0.153	843	tags=34%, list=5%, signal=36%
39	TURASHVILI_BREAST_LOBULAR_CARCINOMA_VS_LOBULAR_NORMAL_DN		71	0.61	1.83	0	0.005	0.184	3993	tags=55%, list=24%, signal=72%
40	VILLANUEVA_LIVER_CANCER_KRT19_DN		53	0.65	1.82	0.002	0.006	0.198	2127	tags=32%, list=13%, signal=37%
41	KIM_LIVER_CANCER_POOR_SURVIVAL_DN		34	0.69	1.82	0.002	0.006	0.211	1290	tags=44%, list=8%, signal=48%
42	RICKMAN_HEAD_AND_NECK_CANCER_F		39	0.68	1.82	0	0.006	0.232	2121	tags=59%, list=13%, signal=67%
43	DELYS_THYROID_CANCER_DN		219	0.54	1.81	0	0.007	0.253	2855	tags=48%, list=17%, signal=58%
44	ZHU_CMV_ALL_DN		113	0.57	1.79	0	0.011	0.362	2441	tags=38%, list=15%, signal=44%
45	CHIANG_LIVER_CANCER_SUBCLASS_PROLIFERATION_DN		140	0.55	1.79	0	0.011	0.362	2539	tags=40%, list=15%, signal=47%
46	ABE_INNER_EAR		39	0.66	1.78	0	0.012	0.417	1755	tags=33%, list=11%, signal=37%
47	BARRIER_CANCER_RELAPSE_NORMAL_SAMPLE_UP		31	0.69	1.78	0.003	0.013	0.436	3071	tags=48%, list=18%, signal=59%
48	CHARAFE_BREAST_CANCER_BASAL_VS_MESENCHYMAL_DN		50	0.64	1.78	0	0.013	0.45	2858	tags=48%, list=17%, signal=58%
49	BROWNE_HCMV_INFECTION_2HR_UP		36	0.67	1.78	0	0.014	0.467	1866	tags=36%, list=11%, signal=41%
50	WATANABE_COLON_CANCER_MSI_VS_MSS_UP		28	0.7	1.77	0	0.014	0.488	2394	tags=43%, list=14%, signal=50%

na_neg

	GS follow link to MSigDB	GS DETAILS	SIZE	ES	NES	NOM p-val	FDR q-val	FWER p-val	RANK AT MAX	LEADING EDGE
1	BLANCO_MELO_BETA_INTERFERON_TREATED_BRONCHIAL_EPITHELIAL_CELLS_DN	Details ...	208	-0.76	-2.79	0	0	0	1164	tags=43%, list=7%, signal=45%
2	JAEGER_METASTASIS_DN	Details ...	252	-0.72	-2.7	0	0	0	1586	tags=52%, list=9%, signal=56%
3	BOSCO_EPITHELIAL_DIFFERENTIATION_MODULE	Details ...	64	-0.85	-2.65	0	0	0	870	tags=56%, list=5%, signal=59%
4	HOLLERN_SQUAMOUS_BREAST_TUMOR	Details ...	136	-0.76	-2.63	0	0	0	1234	tags=57%, list=7%, signal=61%
5	RICKMAN_TUMOR_DIFFERENTIATED_WELL_VS_POORLY_DN	Details ...	375	-0.65	-2.51	0	0	0	2032	tags=36%, list=12%, signal=40%
6	RICKMAN_HEAD_AND_NECK_CANCER_C	Details ...	97	-0.75	-2.48	0	0	0	1312	tags=57%, list=8%, signal=61%

7	RICKMAN_TUMOR_DIFFERENTIATED_WELL_VS_MODERATELY_DN	Details ...	106	-0.73	-2.48	0	0	0	1320	tags=42%, list=8%, signal=45%
8	WANG_BARRETTS_ESOPHAGUS_AND_ESOPHAGUS_CANCER_DN	Details ...	36	-0.89	-2.46	0	0	0	1083	tags=81%, list=6%, signal=86%
9	HUPER_BREAST_BASAL_VS_LUMINAL_UP	Details ...	53	-0.8	-2.44	0	0	0	1229	tags=58%, list=7%, signal=63%
10	RICKMAN_HEAD_AND_NECK_CANCER_E	Details ...	86	-0.74	-2.41	0	0	0	1016	tags=57%, list=6%, signal=60%
11	WANG_ESOPHAGUS_CANCER_VS_NORMAL_DN	Details ...	101	-0.72	-2.39	0	0	0	2631	tags=56%, list=16%, signal=67%
12	RICKMAN_METASTASIS_DN	Details ...	253	-0.61	-2.28	0	0	0	2465	tags=33%, list=15%, signal=38%
13	ONDER_CDH1_TARGETS_2_DN	Details ...	456	-0.57	-2.25	0	0	0	2120	tags=39%, list=13%, signal=44%
14	SESTO_RESPONSE_TO_UV_C1	Details ...	72	-0.68	-2.18	0	0	0	2050	tags=31%, list=12%, signal=35%
15	LIN_SILENCED_BY_TUMOR_MICROENVIRONMENT	Details ...	105	-0.64	-2.17	0	0	0	1304	tags=40%, list=8%, signal=43%
16	CHARAFE_BREAST_CANCER_BASAL_VS_MESENCHYMAL_UP	Details ...	122	-0.62	-2.17	0	0	0	2808	tags=42%, list=17%, signal=50%
17	WANG_BARRETTS_ESOPHAGUS_DN	Details ...	25	-0.82	-2.16	0	0	0	2344	tags=84%, list=14%, signal=98%
18	ONDER_CDH1_TARGETS_3_DN	Details ...	56	-0.7	-2.14	0	0	0.001	1427	tags=52%, list=9%, signal=56%
19	CROMER_METASTASIS_DN	Details ...	76	-0.65	-2.13	0	0	0.001	2205	tags=38%, list=13%, signal=44%
20	ONDER_CDH1_TARGETS_1_DN	Details ...	166	-0.56	-2.01	0	0.001	0.012	1427	tags=20%, list=9%, signal=22%
21	CHANG_IMMORTALIZED_BY_HPV31_DN		55	-0.64	-1.97	0	0.002	0.036	1197	tags=33%, list=7%, signal=35%
22	HUMMERICH_BENIGN_SKIN_TUMOR_UP		15	-0.86	-1.96	0	0.002	0.055	533	tags=47%, list=3%, signal=48%
23	BLANCO_MELO_COVID19_BRONCHIAL_EPITHELIAL_CELLS_SARS_COV_2_INFECTION_UP		140	-0.54	-1.91	0	0.005	0.121	747	tags=17%, list=4%, signal=18%
24	DOANE_BREAST_CANCER_ESR1_DN		37	-0.67	-1.91	0.003	0.005	0.126	1603	tags=62%, list=10%, signal=69%
25	CROMER_TUMORIGENESIS_DN		44	-0.65	-1.89	0	0.007	0.172	809	tags=45%, list=5%, signal=48%
26	DARWICHE_PAPILLOMA_RISK_HIGH_UP		129	-0.54	-1.86	0	0.011	0.267	613	tags=14%, list=4%, signal=14%
27	SESTO_RESPONSE_TO_UV_C4		20	-0.74	-1.86	0	0.011	0.282	486	tags=20%, list=3%, signal=21%
28	SMID_BREAST_CANCER_LUMINAL_A_DN		18	-0.77	-1.85	0	0.011	0.297	761	tags=22%, list=5%, signal=23%
29	CHARAFE_BREAST_CANCER_LUMINAL_VS_BASAL_DN		449	-0.46	-1.84	0	0.014	0.359	2217	tags=31%, list=13%, signal=35%
30	ODONNELL_METASTASIS_DN		23	-0.71	-1.83	0	0.014	0.376	620	tags=26%, list=4%, signal=27%
31	HINATA_NFKB_TARGETS_KERATINOCYTE_DN		22	-0.71	-1.83	0.002	0.015	0.394	705	tags=45%, list=4%, signal=47%
32	AMIT_SERUM_RESPONSE_240_MCF10A		55	-0.6	-1.82	0	0.018	0.453	2595	tags=38%, list=16%, signal=45%
33	FARMER_BREAST_CANCER_CLUSTER_3		16	-0.77	-1.8	0.005	0.023	0.568	2434	tags=69%, list=15%, signal=80%
34	NIKOLSKY_BREAST_CANCER_19Q13.1_AMPLICON		22	-0.71	-1.79	0.005	0.023	0.579	1453	tags=36%, list=9%, signal=40%
35	DARWICHE_PAPILLOMA_RISK_LOW_UP		139	-0.5	-1.78	0	0.026	0.624	613	tags=12%, list=4%, signal=13%
36	MURAKAMI_UV_RESPONSE_1HR_UP		15	-0.76	-1.75	0.002	0.038	0.779	219	tags=27%, list=1%, signal=27%

37	HUMMERICH_MALIGNANT_SKIN_TUMOR_UP		17	-0.73	-1.74	0	0.041	0.807	164	tags=35%, list=1%, signal=36%
38	ENK_UV_RESPONSE_EPIDERMIS_UP		289	-0.46	-1.74	0	0.043	0.831	2007	tags=20%, list=12%, signal=23%
39	PYEON_CANCER_HEAD_AND_NECK_VS_CERVICAL_DN		27	-0.65	-1.74	0.005	0.043	0.836	692	tags=37%, list=4%, signal=39%
40	GARGALOVIC_RESPONSE_TO_OXIDIZED_PHOSPHOLIPIDS_RED_UP		16	-0.72	-1.73	0.01	0.043	0.845	961	tags=31%, list=6%, signal=33%
41	DAZARD_UV_RESPONSE_CLUSTER_G24		22	-0.69	-1.73	0	0.042	0.847	2282	tags=50%, list=14%, signal=58%
42	NAKAMURA_METASTASIS_MODEL_UP		43	-0.59	-1.73	0	0.043	0.861	1801	tags=30%, list=11%, signal=34%
43	SIMBULAN_UV_RESPONSE_NORMAL_DN		33	-0.62	-1.73	0.003	0.042	0.863	2388	tags=39%, list=14%, signal=46%
44	SMID_BREAST_CANCER_RELAPSE_IN_BONE_DN		268	-0.45	-1.71	0	0.05	0.909	1943	tags=30%, list=12%, signal=34%
45	SCHLINGEMANN_SKIN_CARCINOGENESIS_TPA_UP		35	-0.6	-1.69	0.007	0.067	0.959	1157	tags=20%, list=7%, signal=21%
46	HOLLERN_EMT_BREAST_TUMOR_DN		122	-0.48	-1.68	0	0.072	0.975	2526	tags=39%, list=15%, signal=45%
47	QUINTENS_EMBRYONIC_BRAIN_RESPONSE_TO_IR		80	-0.51	-1.64	0.003	0.106	0.995	3088	tags=43%, list=18%, signal=52%
48	WINTER_HYPOXIA_UP		93	-0.49	-1.61	0	0.139	0.999	1999	tags=26%, list=12%, signal=29%
49	BLANCO_MELO_COVID19_BRONCHIAL_EPITHELIAL_CELLS_SARS_COV_2_INFECTION_DN		56	-0.52	-1.59	0	0.166	1	1905	tags=41%, list=11%, signal=46%
50	ROYLANCE_BREAST_CANCER_16Q_COPY_NUMBER_UP		53	-0.52	-1.59	0.003	0.164	1	2661	tags=30%, list=16%, signal=36%

Supplementary Table 3c: GSEA results of EAC vs healthy tissue samples. MSigDB Collection: hallmark gene sets.

na_pos

	GS follow link to MSigDB	GS DETAILS	SIZE	ES	NES	NOM p-val	FDR q-val	FWER p-val	RANK AT MAX	LEADING EDGE
1	HALLMARK_EPITHELIAL_MESENCHYMAL_TRANSITION	Details ...	196	0.73	1.97	0	0	0	2889	tags=62%, list=17%, signal=74%
2	HALLMARK_INFLAMMATORY_RESPONSE	Details ...	191	0.73	1.96	0	0	0	2086	tags=50%, list=12%, signal=56%
3	HALLMARK_COAGULATION	Details ...	113	0.72	1.88	0	0	0	1946	tags=45%, list=12%, signal=51%
4	HALLMARK_ANGIOGENESIS	Details ...	32	0.79	1.86	0	0	0	2270	tags=66%, list=14%, signal=76%
5	HALLMARK_TNFA_SIGNALING_VIA_NFKB	Details ...	199	0.69	1.85	0	0	0.001	3192	tags=57%, list=19%, signal=70%
6	HALLMARK_INTERFERON_ALPHA_RESPONSE	Details ...	97	0.7	1.84	0	0	0.001	3149	tags=61%, list=19%, signal=75%
7	HALLMARK_INTERFERON_GAMMA_RESPONSE	Details ...	198	0.68	1.83	0	0	0.001	3232	tags=57%, list=19%, signal=70%
8	HALLMARK_PANCREAS_BETA_CELLS	Details ...	31	0.79	1.83	0	0	0.001	1524	tags=45%, list=9%, signal=50%
9	HALLMARK_KRAS_SIGNALING_UP	Details ...	191	0.68	1.81	0	0	0.001	2929	tags=53%, list=18%, signal=63%
10	HALLMARK_IL6_JAK_STAT3_SIGNALING	Details ...	83	0.69	1.77	0	0	0.001	3515	tags=59%, list=21%, signal=74%
11	HALLMARK_ALLOGRAFT_REJECTION	Details ...	188	0.65	1.73	0	0	0.001	3270	tags=49%, list=20%, signal=61%
12	HALLMARK_COMPLEMENT	Details ...	189	0.64	1.71	0	0	0.002	3051	tags=42%, list=18%, signal=51%
13	HALLMARK_TGF_BETA_SIGNALING	Details ...	53	0.62	1.55	0.006	0.004	0.057	4084	tags=49%, list=24%, signal=65%
14	HALLMARK_IL2_STAT5_SIGNALING	Details ...	192	0.57	1.54	0	0.005	0.07	4225	tags=50%, list=25%, signal=66%
15	HALLMARK_UV_RESPONSE_DN	Details ...	143	0.56	1.51	0.001	0.008	0.125	3615	tags=37%, list=22%, signal=47%
16	HALLMARK_HEDGEHOG_SIGNALING	Details ...	34	0.63	1.5	0.013	0.007	0.126	3070	tags=53%, list=18%, signal=65%
17	HALLMARK_APOPTOSIS	Details ...	159	0.53	1.39	0.004	0.032	0.477	3581	tags=37%, list=21%, signal=47%
18	HALLMARK_HYPOXIA	Details ...	189	0.51	1.36	0.006	0.05	0.651	3169	tags=34%, list=19%, signal=42%
19	HALLMARK_APICAL_JUNCTION	Details ...	184	0.5	1.35	0.007	0.053	0.693	4162	tags=45%, list=25%, signal=59%
20	HALLMARK_UV_RESPONSE_UP	Details ...	150	0.47	1.25	0.049	0.156	0.975	4133	tags=37%, list=25%, signal=48%

na_neg

	GS follow link to MSigDB	GS DETAILS	SIZE	ES	NES	NOM p-val	FDR q-val	FWER p-val	RANK AT MAX	LEADING EDGE
1	HALLMARK_MYC_TARGETS_V1	Details ...	200	-0.42	-2.11	0	0	0	6057	tags=57%, list=36%, signal=88%
2	HALLMARK_OXIDATIVE_PHOSPHORYLATION	Details ...	198	-0.44	-1.79	0	0.007	0.002	4697	tags=54%, list=28%, signal=74%

Supplementary Table 3d: GSEA results of EAC vs healthy tissue samples. MSigDB Collection: C2 chemical and genetic perturbations (cgp) gene sets (showing max. 50 results).

na_pos										
	GS follow link to MSigDB	GS DETAILS	SIZE	ES	NES	NOM p-val	FDR q-val	FWER p-val	RANK AT MAX	LEADING EDGE
1	CROMER_TUMORIGENESIS_UP	Details ...	55	0.84	2.1	0	0	0	1204	tags=65%, list=7%, signal=70%
2	ANASTASSIOU_MULTICANCER_INVASIVENESS_SIGNATURE	Details ...	61	0.82	2.08	0	0	0	1533	tags=70%, list=9%, signal=77%
3	TURASHVILI_BREAST_LOBULAR_CARCINOMA_VS_DUCTAL_NORMAL_UP	Details ...	66	0.8	2.04	0	0	0	2041	tags=64%, list=12%, signal=72%
4	TURASHVILI_BREAST_LOBULAR_CARCINOMA_VS_LOBULAR_NORMAL_DN	Details ...	71	0.77	2	0	0	0	1387	tags=52%, list=8%, signal=57%
5	SANA_TNF_SIGNALING_UP	Details ...	82	0.76	1.98	0	0	0	2838	tags=68%, list=17%, signal=82%
6	LIANG_SILENCED_BY_METHYLATION_2	Details ...	49	0.79	1.97	0	0	0	2637	tags=67%, list=16%, signal=80%
7	WANG_BARRETTS_ESOPHAGUS_AND_ESOPHAGUS_CANCER_UP	Details ...	26	0.86	1.97	0	0	0	1953	tags=77%, list=12%, signal=87%
8	MCLACHLAN_DENTAL_CARIES_UP	Details ...	233	0.73	1.97	0	0	0	3045	tags=67%, list=18%, signal=81%
9	POOLA_INVASIVE_BREAST_CANCER_UP	Details ...	259	0.73	1.96	0	0	0	2352	tags=51%, list=14%, signal=58%
10	BLANCO_MELO_COVID19_SARS_COV_2_POS_PATIENT_LUNG_TISSUE_UP	Details ...	141	0.73	1.96	0	0	0	1999	tags=57%, list=12%, signal=64%
11	FARMER_BREAST_CANCER_CLUSTER_4	Details ...	19	0.89	1.95	0	0	0	1560	tags=89%, list=9%, signal=99%
12	WANG_ESOPHAGUS_CANCER_VS_NORMAL_UP	Details ...	119	0.73	1.95	0	0	0	2383	tags=61%, list=14%, signal=70%
13	ROY_WOUND_BLOOD_VESSEL_UP	Details ...	49	0.79	1.94	0	0	0	2503	tags=69%, list=15%, signal=81%
14	LINDSTEDT_DENDRITIC_CELL_MATURATION_A	Details ...	59	0.77	1.94	0	0	0	2486	tags=64%, list=15%, signal=75%
15	VERHAAK_AML_WITH_NPM1_MUTATED_UP	Details ...	178	0.72	1.93	0	0	0.001	3016	tags=61%, list=18%, signal=73%
16	ALTEMEIER_RESPONSE_TO_LPS_WITH_MECHANICAL_VENTILATION	Details ...	118	0.73	1.91	0	0	0.002	1878	tags=53%, list=11%, signal=60%
17	SCHUETZ_BREAST_CANCER_DUCTAL_INVASIVE_UP	Details ...	344	0.71	1.91	0	0	0.002	3544	tags=68%, list=21%, signal=84%
18	CLASPER_LYMPHATIC_VESSELS_DURING_METASTASIS_DN	Details ...	33	0.81	1.91	0	0	0.002	1637	tags=64%, list=10%, signal=70%
19	SCHOEN_NFKB_SIGNALING	Details ...	32	0.8	1.9	0	0	0.002	2063	tags=66%, list=12%, signal=75%
20	LIU_CDX2_TARGETS_UP	Details ...	33	0.79	1.9	0	0	0.002	1850	tags=52%, list=11%, signal=58%
21	CROONQUIST_STROMAL_STIMULATION_UP		51	0.76	1.9	0	0	0.002	1964	tags=61%, list=12%, signal=69%
22	NAKAYAMA_SOFT_TISSUE_TUMORS_PCA1_UP		75	0.74	1.89	0	0	0.003	2034	tags=60%, list=12%, signal=68%
23	LIAN_LIPA_TARGETS_3M		55	0.75	1.89	0	0	0.003	1431	tags=49%, list=9%, signal=54%
24	RICKMAN_HEAD_AND_NECK_CANCER_D		26	0.83	1.88	0	0	0.005	988	tags=46%, list=6%, signal=49%
25	HINATA_NFKB_TARGETS_FIBROBLAST_UP		75	0.72	1.88	0	0	0.007	1898	tags=37%, list=11%, signal=42%
26	TSAI_RESPONSE_TO_RADIATION_THERAPY		32	0.79	1.87	0	0	0.007	2466	tags=75%, list=15%, signal=88%
27	WORSCHECH_TUMOR_REJECTION_UP		54	0.74	1.87	0	0	0.007	3326	tags=67%, list=20%, signal=83%

28	VECCHI_GASTRIC_CANCER_ADVANCED_VS_EARLY_DN		139	0.71	1.87	0	0	0.007	2034	tags=35%, list=12%, signal=40%
29	UROSEVIC_RESPONSE_TO_IMIQUIMOD		19	0.84	1.87	0	0	0.007	2594	tags=84%, list=16%, signal=100%
30	SANA_RESPONSE_TO_IFNG_UP		74	0.72	1.86	0	0	0.012	3796	tags=77%, list=23%, signal=99%
31	HAHTOLA_MYCOSIS_FUNGOIDES_CD4_UP		59	0.73	1.86	0	0	0.012	2826	tags=63%, list=17%, signal=75%
32	BLANCO_MELO_COVID19_BRONCHIAL_EPITHELIAL_CELLS_SARS_COV_2_INFECTION_UP		140	0.7	1.86	0	0	0.012	2908	tags=60%, list=17%, signal=72%
33	VECCHI_GASTRIC_CANCER_ADVANCED_VS_EARLY_UP		165	0.69	1.85	0	0	0.012	2623	tags=52%, list=16%, signal=61%
34	WANG_BARRETTS_ESOPHAGUS_UP		49	0.74	1.85	0	0	0.012	2274	tags=49%, list=14%, signal=57%
35	BLANCO_MELO_COVID19_SARS_COV_2_INFECTION_CALU3_CELLS_UP		304	0.69	1.85	0	0	0.013	3240	tags=62%, list=19%, signal=75%
36	GHANDHI_BYSTANDER_IRRADIATION_UP		78	0.71	1.84	0	0	0.016	1795	tags=44%, list=11%, signal=49%
37	GAURNIER_PSMD4_TARGETS		64	0.73	1.84	0	0	0.016	3623	tags=78%, list=22%, signal=99%
38	SERVITJA_ISLET_HNF1A_TARGETS_DN		91	0.71	1.84	0	0	0.016	1451	tags=37%, list=9%, signal=41%
39	PICCALUGA_ANGIOIMMUNOBLASTIC_LYMPHOMA_UP		208	0.69	1.84	0	0	0.016	3322	tags=59%, list=20%, signal=73%
40	MOSERLE_IFNA_RESPONSE		31	0.78	1.84	0	0	0.019	2796	tags=74%, list=17%, signal=89%
41	GALINDO_IMMUNE_RESPONSE_TO_ENTEROTOXIN		79	0.71	1.84	0	0.001	0.021	3192	tags=53%, list=19%, signal=65%
42	KIM_GLIS2_TARGETS_UP		85	0.71	1.84	0	0.001	0.021	3169	tags=72%, list=19%, signal=88%
43	BLANCO_MELO_RESPIRATORY_SYNCYTIAL_VIRUS_INFECTION_A594_CELLS_UP		273	0.68	1.84	0	0	0.021	3123	tags=62%, list=19%, signal=74%
44	WINZEN_DEGRADED_VIA_KHSRP		96	0.7	1.83	0	0.001	0.022	3018	tags=52%, list=18%, signal=63%
45	LIEN_BREAST_CARCINOMA_METAPLASTIC		32	0.78	1.83	0	0.001	0.023	2323	tags=63%, list=14%, signal=72%
46	DASU_IL6_SIGNALING_UP		60	0.72	1.83	0	0.001	0.023	2973	tags=57%, list=18%, signal=69%
47	HECKER_IFNB1_TARGETS		85	0.71	1.83	0	0.001	0.025	3221	tags=71%, list=19%, signal=87%
48	CORRE_MULTIPLE_MYELOMA_UP		68	0.71	1.82	0	0.001	0.035	2349	tags=46%, list=14%, signal=53%
49	HINATA_NFKB_TARGETS_KERATINOCYTE_UP		82	0.7	1.82	0	0.001	0.036	3192	tags=57%, list=19%, signal=71%
50	VALK_AML_CLUSTER_5		31	0.77	1.82	0	0.001	0.038	3129	tags=77%, list=19%, signal=95%

na_neg

	GS follow link to MSigDB	GS DETAILS	SIZE	ES	NES	NOM p-val	FDR q-val	FWER p-val	RANK AT MAX	LEADING EDGE
1	GARY_CD5_TARGETS_DN	Details ...	434	-0.12			1	0	4927	tags=32%, list=29%, signal=45%
2	JAEGER_METASTASIS_DN	Details ...	252	-0.56			1	0	2326	tags=58%, list=14%, signal=66%
3	ENK_UV_RESPONSE_KERATINOCYTE_DN	Details ...	478	-0.13			1	0	5121	tags=34%, list=31%, signal=47%
4	RICKMAN_METASTASIS_DN	Details ...	253	-0.22			1	0	2374	tags=24%, list=14%, signal=28%
5	SHEN_SMARCA2_TARGETS_UP	Details ...	426	-0.29			1	0	6668	tags=53%, list=40%, signal=86%

6	KAUFFMANN_DNA_REPAIR_GENES	Details ...	221	-0.21			1	0	6116	tags=41%, list=37%, signal=64%
7	MANALO_HYPOXIA_DN	Details ...	288	-0.2			1	0	5743	tags=40%, list=34%, signal=60%
8	WONG_MITOCHONDRIA_GENE_MODULE	Details ...	216	-0.28			1	0	4775	tags=49%, list=29%, signal=67%
9	ZHANG_BREAST_CANCER_PROGENITORS_UP	Details ...	423	-0.14			1	0	6092	tags=45%, list=36%, signal=69%
10	MOOTHA_HUMAN_MITODB_6_2002	Details ...	412	-0.23			1	0	4811	tags=46%, list=29%, signal=62%
11	MOOTHA_MITOCHONDRIA	Details ...	430	-0.29			1	0	4890	tags=47%, list=29%, signal=65%
12	HSIAO_HOUSEKEEPING_GENES	Details ...	391	-0.21			1	0	3661	tags=33%, list=22%, signal=41%
13	WONG_EMBRYONIC_STEM_CELL_CORE	Details ...	331	-0.19			1	0	5554	tags=40%, list=33%, signal=58%
14	YAO_TEMPORAL_RESPONSE_TO_PROGESTERONE_CLUSTER_17	Details ...	180	-0.13			1	0	4852	tags=33%, list=29%, signal=46%
15	WANG_BARRETTS_ESOPHAGUS_AND_ESOPHAGUS_CANCER_DN	Details ...	36	-0.77	-2.67	0	0	0	1758	tags=75%, list=11%, signal=84%
16	BLANCO_MELO_BETA_INTERFERON_TREATED_BRONCHIAL_EPITHELIAL_CELLS_DN	Details ...	208	-0.52	-2.6	0	0	0	2422	tags=49%, list=14%, signal=56%
17	BILANGES_SERUM_AND_RAPAMYCIN_SENSITIVE_GENES	Details ...	68	-0.66	-2.56	0	0	0	3661	tags=78%, list=22%, signal=99%
18	CHNG_MULTIPLE_MYELOMA_HYPERPLOID_UP	Details ...	51	-0.62	-2.34	0	0	0	3415	tags=75%, list=20%, signal=93%
19	MOOTHA_VOXPHOS	Details ...	86	-0.54	-2.25	0	0	0.001	5514	tags=74%, list=33%, signal=110%
20	WANG_BARRETTS_ESOPHAGUS_DN	Details ...	25	-0.68	-2.17	0	0	0.01	2638	tags=64%, list=16%, signal=76%
21	HOLLERN_SQUAMOUS_BREAST_TUMOR		136	-0.49	-2.09	0	0.001	0.03	2239	tags=53%, list=13%, signal=61%
22	WANG_ESOPHAGUS_CANCER_VS_NORMAL_DN		101	-0.49	-2.05	0	0.002	0.054	3456	tags=57%, list=21%, signal=72%
23	CROMER_METASTASIS_DN		76	-0.48	-1.99	0	0.004	0.108	3514	tags=54%, list=21%, signal=68%
24	RICKMAN_HEAD_AND_NECK_CANCER_E		86	-0.49	-1.96	0	0.006	0.156	1565	tags=57%, list=9%, signal=63%
25	NIKOLSKY_BREAST_CANCER_19Q13.1_AMPLICON		22	-0.64	-1.96	0	0.005	0.158	4107	tags=68%, list=25%, signal=90%
26	CHARAFE_BREAST_CANCER_BASAL_VS_MESENCHYMAL_UP		122	-0.43	-1.95	0	0.006	0.169	2597	tags=39%, list=16%, signal=46%
27	CROMER_TUMORIGENESIS_DN		44	-0.53	-1.94	0	0.006	0.179	1182	tags=45%, list=7%, signal=49%
28	HORTON_SREBF_TARGETS		24	-0.55	-1.87	0	0.012	0.342	1911	tags=38%, list=11%, signal=42%
29	SCHMIDT_POR_TARGETS_IN_LIMB_BUD_UP		23	-0.56	-1.78	0.006	0.025	0.586	1773	tags=43%, list=11%, signal=49%
30	BOSCO_EPITHELIAL_DIFFERENTIATION_MODULE		64	-0.46	-1.78	0	0.024	0.59	2365	tags=53%, list=14%, signal=62%
31	HUMMERICH_BENIGN_SKIN_TUMOR_UP		15	-0.64	-1.76	0.023	0.028	0.656	2033	tags=40%, list=12%, signal=45%
32	BILANGES_SERUM_RESPONSE_TRANSLATION		28	-0.53	-1.72	0	0.036	0.765	2767	tags=68%, list=17%, signal=81%
33	ABRAMSON_INTERACT_WITH_AIRE		42	-0.47	-1.71	0	0.038	0.787	7310	tags=79%, list=44%, signal=139%
34	STAMBOLSKY_BOUND_BY_MUTATED_TP53		15	-0.58	-1.61	0.023	0.075	0.952	255	tags=13%, list=2%, signal=14%
35	MELLMAN_TUT1_TARGETS_UP		19	-0.54	-1.61	0.025	0.075	0.957	3803	tags=42%, list=23%, signal=54%

36	ZHANG_RESPONSE_TO_CANTHARIDIN_DN		65	-0.4	-1.6	0	0.078	0.967	5279	tags=54%, list=32%, signal=78%
37	PECE_MAMMARY_STEM_CELL_UP		135	-0.36	-1.6	0	0.078	0.968	4037	tags=53%, list=24%, signal=70%
38	LUI_THYROID_CANCER_CLUSTER_3		28	-0.47	-1.59	0.023	0.077	0.968	3299	tags=57%, list=20%, signal=71%
39	RAHMAN_TP53_TARGETS_PHOSPHORYLATED		20	-0.52	-1.59	0	0.077	0.971	4679	tags=50%, list=28%, signal=69%
40	MOOTHA_TCA		15	-0.55	-1.54	0.049	0.106	0.994	4697	tags=53%, list=28%, signal=74%
41	TIEN_INTESTINE_PROBIOTICS_6HR_UP		50	-0.41	-1.53	0	0.11	0.997	4034	tags=56%, list=24%, signal=74%
42	MURAKAMI_UV_RESPONSE_1HR_UP		15	-0.55	-1.51	0.081	0.122	0.999	2237	tags=47%, list=13%, signal=54%
43	ODONNELL_METASTASIS_DN		23	-0.48	-1.5	0.031	0.12	0.999	3936	tags=65%, list=24%, signal=85%
44	WINNEPENNINCKX_MELANOMA_METASTASIS_DN		41	-0.4	-1.47	0.022	0.144	0.999	1283	tags=29%, list=8%, signal=32%
45	HUPER_BREAST_BASAL_VS_LUMINAL_UP		53	-0.41	-1.46	0.03	0.152	0.999	1206	tags=45%, list=7%, signal=49%
46	HOLLERN_EMT_BREAST_TUMOR_DN		122	-0.33	-1.45	0	0.157	1	2374	tags=39%, list=14%, signal=46%
47	NIKOLSKY_BREAST_CANCER_15Q26_AMPLICON		19	-0.48	-1.42	0.039	0.177	1	2039	tags=26%, list=12%, signal=30%
48	ZHONG_RESPONSE_TO_AZACITIDINE_AND_TSA_DN		69	-0.36	-1.42	0	0.178	1	6399	tags=62%, list=38%, signal=101%
49	DEN_INTERACT_WITH_LCA5		26	-0.44	-1.4	0.051	0.195	1	6184	tags=62%, list=37%, signal=98%
50	YAO_TEMPORAL_RESPONSE_TO_PROGESTERONE_CLUSTER_13		175	-0.3	-1.4	0	0.193	1	5529	tags=50%, list=33%, signal=74%

Supplementary Table 3e: GSEA results of EAC disease vs NDB disease tissue samples. MSigDB Collection: hallmark gene sets.

na_pos

	GS follow link to MSigDB	GS DETAILS	SIZE	ES	NES	NOM p-val	FDR q-val	FWER p-val	RANK AT MAX	LEADING EDGE
1	HALLMARK_TNFA_SIGNALING_VIA_NFKB	Details ...	199	0.63	2.08	0	0	0	3564	tags=54%, list=21%, signal=68%
2	HALLMARK_INFLAMMATORY_RESPONSE	Details ...	191	0.6	2	0	0	0	3239	tags=56%, list=19%, signal=69%
3	HALLMARK_ALLOGRAFT_REJECTION	Details ...	188	0.56	1.88	0	0	0	3564	tags=47%, list=21%, signal=59%
4	HALLMARK_EPITHELIAL_MESENCHYMAL_TRANSITION	Details ...	196	0.57	1.88	0	0	0	3252	tags=48%, list=19%, signal=59%
5	HALLMARK_KRAS_SIGNALING_DN	Details ...	157	0.56	1.81	0	0.001	0.004	1535	tags=31%, list=9%, signal=34%
6	HALLMARK_IL6_JAK_STAT3_SIGNALING	Details ...	83	0.55	1.67	0	0.003	0.02	3961	tags=52%, list=24%, signal=68%
7	HALLMARK_INTERFERON_GAMMA_RESPONSE	Details ...	198	0.49	1.62	0	0.006	0.047	4288	tags=44%, list=26%, signal=58%
8	HALLMARK_P53_PATHWAY	Details ...	198	0.47	1.56	0	0.015	0.12	3384	tags=29%, list=20%, signal=36%
9	HALLMARK_APICAL_JUNCTION	Details ...	184	0.47	1.55	0	0.015	0.138	4071	tags=39%, list=24%, signal=50%
#	HALLMARK_IL2_STAT5_SIGNALING	Details ...	192	0.46	1.52	0.002	0.018	0.183	3798	tags=39%, list=23%, signal=49%
#	HALLMARK_APOPTOSIS	Details ...	159	0.46	1.49	0.004	0.026	0.268	3001	tags=26%, list=18%, signal=32%
#	HALLMARK_APICAL_SURFACE	Details ...	42	0.54	1.46	0.033	0.037	0.386	2781	tags=36%, list=17%, signal=43%
#	HALLMARK_HYPOXIA	Details ...	189	0.43	1.42	0.01	0.052	0.53	3608	tags=39%, list=22%, signal=49%
#	HALLMARK_MYOGENESIS	Details ...	170	0.42	1.37	0.022	0.087	0.749	2549	tags=26%, list=15%, signal=31%
#	HALLMARK_WNT_BETA_CATENIN_SIGNALING	Details ...	41	0.5	1.37	0.065	0.082	0.755	3086	tags=37%, list=18%, signal=45%
#	HALLMARK_COMPLEMENT	Details ...	189	0.41	1.36	0.024	0.084	0.781	2201	tags=22%, list=13%, signal=25%
#	HALLMARK_ANGIOGENESIS	Details ...	32	0.52	1.35	0.1	0.084	0.8	2916	tags=34%, list=17%, signal=42%
#	HALLMARK_ESTROGEN_RESPONSE_EARLY	Details ...	198	0.39	1.31	0.032	0.124	0.926	1449	tags=22%, list=9%, signal=24%
#	HALLMARK_KRAS_SIGNALING_UP	Details ...	191	0.37	1.22	0.114	0.253	0.995	3523	tags=42%, list=21%, signal=52%

na_neg

	GS follow link to MSigDB	GS DETAILS	SIZE	ES	NES	NOM p-val	FDR q-val	FWER p-val	RANK AT MAX	LEADING EDGE
1	HALLMARK_OXIDATIVE_PHOSPHORYLATION	Details ...	198	-0.58	-2.36	0	0	0	4731	tags=66%, list=28%, signal=90%
2	HALLMARK_PANCREAS_BETA_CELLS	Details ...	31	-0.75	-2.2	0	0	0	1119	tags=52%, list=7%, signal=55%
3	HALLMARK_FATTY_ACID_METABOLISM	Details ...	151	-0.45	-1.72	0	0.003	0.008	2637	tags=33%, list=16%, signal=39%
4	HALLMARK_BILE_ACID_METABOLISM	Details ...	99	-0.47	-1.72	0	0.003	0.009	1920	tags=37%, list=11%, signal=42%
5	HALLMARK_XENOBIOTIC_METABOLISM	Details ...	178	-0.4	-1.58	0	0.009	0.031	2445	tags=38%, list=15%, signal=44%
6	HALLMARK_PEROXISOME	Details ...	97	-0.41	-1.53	0	0.012	0.049	2278	tags=30%, list=14%, signal=34%
7	HALLMARK_ADIPOGENESIS	Details ...	192	-0.35	-1.39	0	0.038	0.177	4143	tags=48%, list=25%, signal=64%

Supplementary Table 3f: GSEA results of EAC disease vs NDB disease tissue samples. MSigDB Collection: C2 chemical and genetic perturbations (cgp) gene sets (showing max. 50 results).

na_pos										
	GS follow link to MSigDB	GS DETAILS	SIZE	ES	NES	NOM p-val	FDR q-val	FWER p-val	RANK AT MAX	LEADING EDGE
1	HOLLERN_SQUAMOUS_BREAST_TUMOR	Details ...	136	0.8	2.54	0	0	0	1069	tags=52%, list=6%, signal=55%
2	BLANCO_MELO_BETA_INTERFERON_TREATED_BRONCHIAL_EPITHELIAL_CELLS_DN	Details ...	208	0.75	2.51	0	0	0	1418	tags=41%, list=8%, signal=45%
3	BOSCO_EPITHELIAL_DIFFERENTIATION_MODULE	Details ...	64	0.84	2.43	0	0	0	1089	tags=55%, list=7%, signal=58%
4	JAEGER_METASTASIS_DN	Details ...	252	0.72	2.43	0	0	0	1353	tags=42%, list=8%, signal=46%
5	RICKMAN_HEAD_AND_NECK_CANCER_C	Details ...	97	0.78	2.43	0	0	0	912	tags=55%, list=5%, signal=57%
6	VECCHI_GASTRIC_CANCER_ADVANCED_VS_EARLY_UP	Details ...	165	0.74	2.42	0	0	0	3204	tags=72%, list=19%, signal=88%
7	RICKMAN_TUMOR_DIFFERENTIATED_WELL_VS_MODERATELY_DN	Details ...	106	0.76	2.37	0	0	0	2293	tags=55%, list=14%, signal=63%
8	WANG_BARRETTS_ESOPHAGUS_AND_ESOPHAGUS_CANCER_DN	Details ...	36	0.88	2.34	0	0	0	698	tags=67%, list=4%, signal=69%
9	HUPER_BREAST_BASAL_VS_LUMINAL_UP	Details ...	53	0.83	2.32	0	0	0	1438	tags=68%, list=9%, signal=74%
10	RICKMAN_TUMOR_DIFFERENTIATED_WELL_VS_POORLY_DN	Details ...	375	0.67	2.31	0	0	0	2628	tags=39%, list=16%, signal=45%
11	ONDER_CDH1_TARGETS_3_DN	Details ...	56	0.81	2.3	0	0	0	1264	tags=54%, list=8%, signal=58%
12	BLANCO_MELO_COVID19_BRONCHIAL_EPITHELIAL_CELLS_SARS_COV_2_INFECTION_UP	Details ...	140	0.71	2.29	0	0	0	2657	tags=51%, list=16%, signal=61%
13	ONDER_CDH1_TARGETS_2_DN	Details ...	456	0.63	2.21	0	0	0	2109	tags=38%, list=13%, signal=42%
14	ONDER_CDH1_TARGETS_1_DN	Details ...	166	0.66	2.18	0	0	0	1089	tags=20%, list=7%, signal=22%
15	RICKMAN_HEAD_AND_NECK_CANCER_E	Details ...	86	0.72	2.17	0	0	0	485	tags=44%, list=3%, signal=45%
16	LIN_SILENCED_BY_TUMOR_MICROENVIRONMENT	Details ...	105	0.68	2.14	0	0	0	1616	tags=41%, list=10%, signal=45%
17	SESTO_RESPONSE_TO_UV_C1	Details ...	72	0.71	2.12	0	0	0	1850	tags=29%, list=11%, signal=33%
18	HINATA_NFKB_TARGETS_KERATINOCYTE_UP	Details ...	82	0.69	2.11	0	0	0	2839	tags=49%, list=17%, signal=58%
19	BLANCO_MELO_COVID19_SARS_COV_2_INFECTION_A594_CELLS_UP	Details ...	79	0.7	2.1	0	0	0	1511	tags=42%, list=9%, signal=46%
20	CHARAFE_BREAST_CANCER_LUMINAL_VS_BASAL_DN	Details ...	449	0.6	2.1	0	0	0	3538	tags=48%, list=21%, signal=59%
21	SMIRNOV_CIRCULATING_ENDOTHELIOCYTES_IN_CANCER_UP		156	0.64	2.07	0	0	0	2768	tags=53%, list=17%, signal=62%
22	MCLACHLAN_DENTAL_CARIES_UP		233	0.61	2.07	0	0	0	3386	tags=60%, list=20%, signal=74%
23	LIM_MAMMARY_STEM_CELL_UP		461	0.59	2.06	0	0	0	3551	tags=50%, list=21%, signal=62%
24	BLANCO_MELO_RESPIRATORY_SYNCYTIAL_VIRUS_INFECTION_A594_CELLS_UP		273	0.6	2.06	0	0	0	3017	tags=48%, list=18%, signal=57%
25	ALTEMEIER_RESPONSE_TO_LPS_WITH_MECHANICAL_VENTILATION		118	0.65	2.06	0	0	0	3190	tags=56%, list=19%, signal=69%
26	CROMER_TUMORIGENESIS_UP		55	0.72	2.05	0	0	0	2639	tags=62%, list=16%, signal=73%
27	DOANE_BREAST_CANCER_ESR1_DN		37	0.77	2.05	0	0	0	1954	tags=68%, list=12%, signal=76%
28	SMID_BREAST_CANCER_RELAPSE_IN_BONE_DN		268	0.6	2.03	0	0	0.001	2045	tags=35%, list=12%, signal=39%

29	PYEON_CANCER_HEAD_AND_NECK_VS_CERVICAL_DN		27	0.8	2.03	0	0	0.001	1367	tags=67%, list=8%, signal=72%
30	WANG_ESOPHAGUS_CANCER_VS_NORMAL_DN		101	0.63	1.99	0	0	0.003	3111	tags=38%, list=19%, signal=46%
31	PLASARI_TGFB1_TARGETS_10HR_UP		180	0.6	1.99	0	0	0.003	2703	tags=44%, list=16%, signal=52%
32	SCHUETZ_BREAST_CANCER_DUCTAL_INVASIVE_UP		344	0.58	1.99	0	0	0.003	4248	tags=60%, list=25%, signal=79%
33	FLECHNER_BIOPSY_KIDNEY_TRANSPLANT_REJECTED_VS_OK_UP		87	0.65	1.99	0	0	0.003	3344	tags=61%, list=20%, signal=76%
34	CROMER_TUMORIGENESIS_DN		44	0.72	1.98	0	0	0.004	641	tags=41%, list=4%, signal=42%
35	LINDSTEDT_DENDRITIC_CELL_MATURATION_A		59	0.69	1.98	0	0	0.007	3017	tags=58%, list=18%, signal=70%
36	ZHANG_RESPONSE_TO_IKK_INHIBITOR_AND_TNF_UP		219	0.58	1.96	0	0	0.008	3728	tags=44%, list=22%, signal=56%
37	AZARE_NEOPLASTIC_TRANSFORMATION_BY_STAT3_UP		120	0.61	1.96	0	0	0.008	3210	tags=47%, list=19%, signal=57%
38	LIANG_SILENCED_BY_METHYLATION_2		49	0.69	1.95	0	0	0.017	1839	tags=43%, list=11%, signal=48%
39	KUROZUMI_RESPONSE_TO_ONCOCYTIC_VIRUS		40	0.72	1.95	0	0.001	0.018	2612	tags=55%, list=16%, signal=65%
40	RICKMAN_METASTASIS_DN		253	0.57	1.93	0	0.001	0.025	3785	tags=41%, list=23%, signal=52%
41	DAZARD_UV_RESPONSE_CLUSTER_G24		22	0.79	1.92	0	0.001	0.031	1776	tags=45%, list=11%, signal=51%
42	BLANCO_MELO_COVID19_SARS_COV_2_INFECTION_CALU3_CELLS_UP		304	0.56	1.92	0	0.001	0.032	3603	tags=53%, list=22%, signal=66%
43	CHANG_IMMORTALIZED_BY_HPV31_DN		55	0.67	1.92	0	0.001	0.043	2149	tags=38%, list=13%, signal=44%
44	POOLA_INVASIVE_BREAST_CANCER_UP		259	0.56	1.91	0	0.001	0.044	3215	tags=49%, list=19%, signal=60%
45	AMIT_SERUM_RESPONSE_240_MCF10A		55	0.67	1.91	0	0.001	0.054	1879	tags=33%, list=11%, signal=37%
46	GRAHAM_CML_QUIESCENT_VS_CML_DIVIDING_UP		24	0.76	1.9	0	0.001	0.057	3190	tags=67%, list=19%, signal=82%
47	DARWICHE_PAPILLOMA_RISK_HIGH_UP		129	0.6	1.9	0	0.001	0.058	2976	tags=31%, list=18%, signal=37%
48	GALINDO_IMMUNE_RESPONSE_TO_ENTEROTOXIN		79	0.63	1.9	0	0.001	0.063	2947	tags=51%, list=18%, signal=61%
49	BILD_HRAS_ONCOGENIC_SIGNATURE		243	0.56	1.9	0	0.001	0.066	3229	tags=39%, list=19%, signal=47%
50	LU_TUMOR_ENDOTHELIAL_MARKERS_UP		22	0.78	1.88	0	0.002	0.08	1875	tags=64%, list=11%, signal=72%

na_neg

	GS<br>follow link to MSigDB	GS DETAILS	SIZE	ES	NES	NOM p-val	FDR q-val	FWER p-val	RANK AT MAX	LEADING EDGE
1	VECCHI_GASTRIC_CANCER_ADVANCED_VS_EARLY_DN	Details ...	139	-0.81	-3.1	0	0	0	1435	tags=61%, list=9%, signal=66%
2	ANDERSEN_LIVER_CANCER_KRT19_DN	Details ...	60	-0.74	-2.55	0	0	0	2378	tags=55%, list=14%, signal=64%
3	WANG_BARRETTS_ESOPHAGUS_UP	Details ...	49	-0.78	-2.49	0	0	0	1894	tags=65%, list=11%, signal=73%
4	WAMUNYOKOLI_OVARIAN_CANCER_LMP_UP	Details ...	267	-0.61	-2.48	0	0	0	3784	tags=54%, list=23%, signal=69%
5	WAMUNYOKOLI_OVARIAN_CANCER_GRADES_1_2_UP	Details ...	139	-0.65	-2.46	0	0	0	3705	tags=63%, list=22%, signal=80%
6	HSIAO_LIVER_SPECIFIC_GENES	Details ...	185	-0.6	-2.39	0	0	0	1300	tags=36%, list=8%, signal=38%

7	KANG_GLIS3_TARGETS	Details ...	27	-0.82	-2.34	0	0	0	1119	tags=56%, list=7%, signal=59%
8	SMID_BREAST_CANCER_RELAPSE_IN_BONE_UP	Details ...	88	-0.62	-2.3	0	0	0	1246	tags=43%, list=7%, signal=46%
9	SABATES_COLORECTAL_ADENOMA_DN	Details ...	263	-0.55	-2.25	0	0	0	1091	tags=35%, list=7%, signal=37%
10	RICKMAN_HEAD_AND_NECK_CANCER_D	Details ...	26	-0.79	-2.25	0	0	0	1091	tags=62%, list=7%, signal=66%
11	SERVITJA_ISLET_HNF1A_TARGETS_DN	Details ...	91	-0.61	-2.24	0	0	0	1611	tags=52%, list=10%, signal=57%
12	VILLANUEVA_LIVER_CANCER_KRT19_DN	Details ...	53	-0.68	-2.22	0	0	0	1808	tags=43%, list=11%, signal=49%
13	LIU_CDX2_TARGETS_UP	Details ...	33	-0.74	-2.19	0	0	0	945	tags=48%, list=6%, signal=51%
14	BLANCO_MELO_RESPIRATORY_SYNCYTIAL_VIRUS_INFECTION_A594_CELLS_DN	Details ...	109	-0.59	-2.18	0	0	0	2049	tags=49%, list=12%, signal=55%
15	MEBARKI_HCC_PROGENITOR_FZD8CRD_DN	Details ...	358	-0.51	-2.17	0	0	0.003	1532	tags=32%, list=9%, signal=35%
16	WOO_LIVER_CANCER_RECURRENCE_DN	Details ...	66	-0.63	-2.15	0	0	0.003	2168	tags=48%, list=13%, signal=55%
17	WANG_BARRETTS_ESOPHAGUS_AND_ESOPHAGUS_CANCER_UP	Details ...	26	-0.76	-2.15	0	0	0.003	1086	tags=42%, list=6%, signal=45%
18	VARELA_ZMPSTE24_TARGETS_DN	Details ...	41	-0.68	-2.13	0	0	0.004	1762	tags=51%, list=11%, signal=57%
19	BLANCO_MELO_COVID19_SARS_COV_2_INFECTION_CALU3_CELLS_DN	Details ...	22	-0.8	-2.11	0	0	0.006	1055	tags=50%, list=6%, signal=53%
20	CHIANG_LIVER_CANCER_SUBCLASS_PROLIFERATION_DN	Details ...	140	-0.54	-2.1	0	0	0.008	2065	tags=39%, list=12%, signal=44%
21	VECCHI_GASTRIC_CANCER_EARLY_DN		327	-0.49	-2.09	0	0	0.008	1911	tags=37%, list=11%, signal=41%
22	OHGUCHI_LIVER_HNF4A_TARGETS_DN		118	-0.55	-2.08	0	0	0.01	1666	tags=39%, list=10%, signal=43%
23	SERVITJA_LIVER_HNF1A_TARGETS_DN		119	-0.53	-2.04	0	0.001	0.027	1465	tags=34%, list=9%, signal=37%
24	LIEN_BREAST_CARCINOMA_METAPLASTIC_VS_DUCTAL_DN		102	-0.54	-2.02	0	0.002	0.042	2551	tags=54%, list=15%, signal=63%
25	BLANCO_MELO_COVID19_SARS_COV_2_INFECTION_A594_CELLS_DN		69	-0.6	-2.01	0	0.002	0.044	1179	tags=38%, list=7%, signal=40%
26	BARRIER_CANCER_RELAPSE_NORMAL_SAMPLE_UP		31	-0.69	-2.01	0	0.002	0.046	1728	tags=35%, list=10%, signal=40%
27	HOSHIDA_LIVER_CANCER_SURVIVAL_DN		101	-0.55	-2.01	0	0.002	0.047	2286	tags=30%, list=14%, signal=34%
28	WONG_MITOCHONDRIA_GENE_MODULE		216	-0.49	-2.01	0	0.002	0.05	4859	tags=59%, list=29%, signal=82%
29	HOLLERN_MICROACINAR_BREAST_TUMOR_UP		41	-0.63	-2.01	0	0.002	0.051	1583	tags=44%, list=9%, signal=48%
30	DESERT_PERIPORTAL_HEPATOCELLULAR_CARCINOMA_SUBCLASS_UP		119	-0.53	-1.96	0	0.003	0.106	1484	tags=34%, list=9%, signal=38%
31	MOOTHA_GLUCONEOGENESIS		29	-0.66	-1.96	0	0.003	0.115	362	tags=21%, list=2%, signal=21%
32	NAKAYAMA_SOFT_TISSUE_TUMORS_PCA2_DN		75	-0.55	-1.93	0	0.005	0.171	1199	tags=35%, list=7%, signal=37%
33	HOSHIDA_LIVER_CANCER_SUBCLASS_S3		233	-0.47	-1.92	0	0.006	0.196	2499	tags=37%, list=15%, signal=43%
34	KIM_LIVER_CANCER_POOR_SURVIVAL_DN		34	-0.62	-1.89	0.003	0.009	0.311	1762	tags=59%, list=11%, signal=66%
35	LUI_THYROID_CANCER_CLUSTER_5		15	-0.76	-1.87	0.003	0.012	0.389	837	tags=40%, list=5%, signal=42%
36	LIU_PROSTATE_CANCER_UP		93	-0.53	-1.87	0	0.012	0.397	1714	tags=35%, list=10%, signal=39%

37	CHNG_MULTIPLE_MYELOMA_HYPERPLOID_UP		51	-0.58	-1.86	0	0.013	0.442	5515	tags=73%, list=33%, signal=108%
38	MOOTHA_VOXPHOS		86	-0.5	-1.81	0	0.021	0.615	3991	tags=70%, list=24%, signal=91%
39	SANSOM_APC_TARGETS_DN		329	-0.42	-1.8	0	0.024	0.666	1697	tags=27%, list=10%, signal=29%
40	SU_LIVER		30	-0.61	-1.79	0	0.025	0.691	1768	tags=37%, list=11%, signal=41%
41	RUAN_RESPONSE_TO_TNF_DN		72	-0.52	-1.79	0	0.024	0.693	2505	tags=36%, list=15%, signal=42%
42	MOOTHA_TCA		15	-0.7	-1.78	0.003	0.026	0.727	3933	tags=67%, list=24%, signal=87%
43	WATANABE_COLON_CANCER_MSI_VS_MSS_DN		62	-0.52	-1.77	0	0.029	0.771	1001	tags=31%, list=6%, signal=32%
44	ICHIBA_GRAFT_VERSUS_HOST_DISEASE_D7_DN		31	-0.61	-1.77	0	0.029	0.782	756	tags=29%, list=5%, signal=30%
45	BLANCO_MELO_HUMAN_PARAINFLUENZA_VIRUS_3_INFECTION_A594_CELLS_DN		107	-0.47	-1.76	0	0.032	0.815	1238	tags=30%, list=7%, signal=32%
46	SMID_BREAST_CANCER_RELAPSE_IN_LUNG_DN		34	-0.58	-1.76	0.003	0.031	0.821	1163	tags=38%, list=7%, signal=41%
47	LEE_LIVER_CANCER_SURVIVAL_UP		147	-0.45	-1.75	0	0.034	0.852	2522	tags=39%, list=15%, signal=46%
48	SHETH_LIVER_CANCER_VS_TXNIP_LOSS_PAM4		246	-0.42	-1.74	0	0.036	0.873	1491	tags=22%, list=9%, signal=23%
49	BOYAULT_LIVER_CANCER_SUBCLASS_G123_DN		42	-0.55	-1.73	0.01	0.04	0.908	2445	tags=45%, list=15%, signal=53%
50	ICHIBA_GRAFT_VERSUS_HOST_DISEASE_35D_DN		44	-0.54	-1.71	0.003	0.048	0.949	1444	tags=30%, list=9%, signal=32%

Supplementary Table 3g: GSEA results of EAC vs NDB plasma samples. MSigDB Collection: hallmark gene sets.

na_pos										
	GS follow link to MSigDB	GS DETAILS	SIZE	ES	NES	NOM p-val	FDR q-val	FWER p-val	RANK AT MAX	LEADING EDGE
1	HALLMARK_MYC_TARGETS_V2	Details ...	54	0.56	2.18	0	0	0	3508	tags=67%, list=32%, signal=98%
2	HALLMARK_INTERFERON_GAMMA_RESPONSE	Details ...	178	0.46	2.17	0	0	0	2920	tags=53%, list=27%, signal=71%
3	HALLMARK_INTERFERON_ALPHA_RESPONSE	Details ...	87	0.51	2.15	0	0	0	3226	tags=61%, list=30%, signal=86%
4	HALLMARK_MYC_TARGETS_V1	Details ...	192	0.41	1.98	0	0.001	0.004	3599	tags=56%, list=33%, signal=82%
5	HALLMARK_OXIDATIVE_PHOSPHORYLATION	Details ...	191	0.36	1.72	0	0.011	0.039	3258	tags=49%, list=30%, signal=68%
6	HALLMARK_XENOBIOTIC_METABOLISM	Details ...	122	0.32	1.45	0.007	0.122	0.41	2695	tags=40%, list=25%, signal=53%

na_neg										
	GS follow link to MSigDB	GS DETAILS	SIZE	ES	NES	NOM p-val	FDR q-val	FWER p-val	RANK AT MAX	LEADING EDGE
1	HALLMARK_KRAS_SIGNALING_DN	Details ...	64	-0.39	-1.58	0.004	0.126	0.182	1370	tags=30%, list=13%, signal=34%
2	HALLMARK_PROTEIN_SECRETION	Details ...	88	-0.35	-1.47	0.007	0.158	0.404	3772	tags=48%, list=35%, signal=72%
3	HALLMARK_APICAL_SURFACE	Details ...	22	-0.45	-1.4	0.088	0.192	0.616	1208	tags=32%, list=11%, signal=36%
4	HALLMARK_EPITHELIAL_MESENCHYMAL_TRANSITION	Details ...	112	-0.3	-1.34	0.06	0.231	0.782	1703	tags=34%, list=16%, signal=40%

Supplementary Table 3h: GSEA results of EAC vs NDB plasma samples. MSigDB Collection: C2 chemical and genetic perturbations (cgp) gene sets (showing max. 50 results).

na_pos										
	GS<br>follow link to MSigDB	GS DETAILS	SIZE	ES	NES	NOM p-val	FDR q-val	FWER p-val	RANK AT MAX	LEADING EDGE
1	MOSERLE_IFNA_RESPONSE	Details ...	29	0.74	2.53	0	0	0	2080	tags=76%, list=19%, signal=94%
2	BILANGES_SERUM_AND_RAPAMYCIN_SENSITIVE_GENES	Details ...	64	0.61	2.44	0	0	0	3279	tags=86%, list=30%, signal=122%
3	PICCALUGA_ANGIOIMMUNOBLASTIC_LYMPHOMA_UP	Details ...	135	0.51	2.34	0	0	0	1620	tags=43%, list=15%, signal=50%
4	CHNG_MULTIPLE_MYELOMA_HYPERPLOID_UP	Details ...	50	0.59	2.29	0	0.001	0.002	3485	tags=80%, list=32%, signal=117%
5	PYEON_CANCER_HEAD_AND_NECK_VS_CERVICAL_DN	Details ...	13	0.84	2.28	0	0	0.002	1773	tags=100%, list=16%, signal=119%
6	RUNNE_GENDER_EFFECT_UP	Details ...	9	0.93	2.26	0	0.001	0.003	250	tags=89%, list=2%, signal=91%
7	BROWNE_INTERFERON_RESPONSIVE_GENES	Details ...	61	0.55	2.26	0	0.001	0.003	2601	tags=66%, list=24%, signal=86%
8	FARMER_BREAST_CANCER_CLUSTER_1	Details ...	33	0.65	2.25	0	0.001	0.004	3008	tags=79%, list=28%, signal=109%
9	BAELDE_DIABETIC_NEPHROPATHY_UP	Details ...	57	0.57	2.24	0	0.001	0.007	1446	tags=47%, list=13%, signal=54%
10	BENNETT_SYSTEMIC_LUPUS_ERYTHEMATOSUS	Details ...	27	0.67	2.21	0	0.002	0.013	2160	tags=67%, list=20%, signal=83%
11	BOSCO_INTERFERON_INDUCED_ANTIVIRAL_MODULE	Details ...	64	0.54	2.2	0	0.002	0.015	3076	tags=63%, list=28%, signal=87%
12	ICHIBA_GRAFT_VERSUS_HOST_DISEASE_D7_UP	Details ...	91	0.5	2.18	0	0.003	0.026	1654	tags=37%, list=15%, signal=44%
13	ZHANG_ANTIVIRAL_RESPONSE_TO_RIBAVIRIN_UP	Details ...	21	0.68	2.14	0	0.004	0.043	1749	tags=57%, list=16%, signal=68%
14	UROSEVIC_RESPONSE_TO_IMIQUIMOD	Details ...	17	0.72	2.13	0	0.004	0.045	1674	tags=65%, list=15%, signal=76%
15	KIM_GLIS2_TARGETS_UP	Details ...	57	0.54	2.12	0	0.005	0.055	930	tags=32%, list=9%, signal=34%
16	BOWIE_RESPONSE_TO_TAMOXIFEN	Details ...	17	0.71	2.1	0.002	0.005	0.063	1920	tags=71%, list=18%, signal=86%
17	ABRAHAM_ALPC_VS_MULTIPLE_MYELOMA_UP	Details ...	23	0.65	2.1	0	0.005	0.067	1437	tags=48%, list=13%, signal=55%
18	DAUER_STAT3_TARGETS_DN	Details ...	44	0.56	2.09	0	0.005	0.068	2097	tags=59%, list=19%, signal=73%
19	ZHANG_INTERFERON_RESPONSE	Details ...	20	0.67	2.08	0	0.006	0.083	1920	tags=65%, list=18%, signal=79%
20	ICHIBA_GRAFT_VERSUS_HOST_DISEASE_35D_UP	Details ...	111	0.47	2.08	0	0.005	0.085	2424	tags=42%, list=22%, signal=54%
21	MARTENS_TRETINOIN_RESPONSE_UP		193	0.43	2.08	0	0.005	0.088	1664	tags=33%, list=15%, signal=38%
22	HUMMERICH_BENIGN_SKIN_TUMOR_DN		8	0.87	2.07	0	0.006	0.098	392	tags=50%, list=4%, signal=52%
23	BOWIE_RESPONSE_TO_EXTRACELLULAR_MATRIX		17	0.68	2.05	0	0.007	0.133	1920	tags=65%, list=18%, signal=78%
24	HECKER_IFNB1_TARGETS		83	0.48	2.04	0	0.008	0.143	1941	tags=53%, list=18%, signal=64%
25	CHEN_LVAD_SUPPORT_OF_FAILING_HEART_DN		25	0.6	1.98	0	0.017	0.306	1569	tags=44%, list=14%, signal=51%
26	EINAV_INTERFERON_SIGNATURE_IN_CANCER		25	0.61	1.98	0	0.017	0.31	1920	tags=56%, list=18%, signal=68%
27	RADAEVA_RESPONSE_TO_IFNA1_UP		43	0.54	1.98	0	0.017	0.326	1904	tags=44%, list=17%, signal=53%

28	IRITANI_MAD1_TARGETS_DN		42	0.52	1.97	0	0.019	0.366	2804	tags=57%, list=26%, signal=77%
29	PECE_MAMMARY_STEM_CELL_UP		107	0.44	1.96	0	0.023	0.427	3279	tags=61%, list=30%, signal=86%
30	ROETH_TERT_TARGETS_UP		12	0.73	1.95	0	0.024	0.455	2080	tags=75%, list=19%, signal=93%
31	NAKAYAMA_SOFT_TISSUE_TUMORS_PCA2_DN		39	0.52	1.95	0	0.025	0.477	923	tags=41%, list=8%, signal=45%
32	HOSHIDA_LIVER_CANCER_SUBCLASS_S3		160	0.41	1.95	0	0.025	0.489	1930	tags=36%, list=18%, signal=43%
33	LIANG_SILENCED_BY_METHYLATION_2		31	0.56	1.94	0	0.024	0.498	1920	tags=58%, list=18%, signal=70%
34	LI_DCP2_BOUND_MRNA		78	0.46	1.94	0	0.025	0.519	3060	tags=53%, list=28%, signal=73%
35	BOYAULT_LIVER_CANCER_SUBCLASS_G3_DN		31	0.55	1.93	0	0.028	0.569	1620	tags=42%, list=15%, signal=49%
36	MOOTHA_VOXPHOS		78	0.46	1.91	0	0.034	0.651	3207	tags=55%, list=29%, signal=78%
37	SWEET_LUNG_CANCER_KRAS_DN		271	0.38	1.91	0	0.034	0.653	1291	tags=27%, list=12%, signal=29%
38	BILANGES_SERUM_RESPONSE_TRANSLATION		27	0.58	1.91	0	0.034	0.666	3241	tags=81%, list=30%, signal=116%
39	WU_CELL_MIGRATION		115	0.43	1.91	0	0.034	0.682	1741	tags=38%, list=16%, signal=45%
40	WENG_POR_TARGETS_GLOBAL_UP		11	0.74	1.91	0	0.033	0.682	147	tags=27%, list=1%, signal=28%
41	KRASNOSELSKAYA_ILF3_TARGETS_UP		31	0.55	1.9	0	0.035	0.704	1962	tags=58%, list=18%, signal=71%
42	SHEPARD_CRUSH_AND_BURN_MUTANT_UP		152	0.4	1.88	0	0.044	0.802	1988	tags=36%, list=18%, signal=43%
43	LIU_VAV3_PROSTATE_CARCINOGENESIS_UP		54	0.47	1.87	0	0.05	0.84	1899	tags=39%, list=17%, signal=47%
44	HAEGERSTRAND_RESPONSE_TO_IMATINIB		6	0.87	1.87	0	0.049	0.84	422	tags=50%, list=4%, signal=52%
45	FURUKAWA_DUSP6_TARGETS_PCI35_UP		39	0.52	1.87	0.002	0.049	0.842	2238	tags=49%, list=21%, signal=61%
46	MCDOWELL_ACUTE_LUNG_INJURY_DN		31	0.54	1.86	0.002	0.049	0.851	892	tags=26%, list=8%, signal=28%
47	HUMMERICH_MALIGNANT_SKIN_TUMOR_DN		9	0.75	1.86	0.004	0.05	0.859	392	tags=33%, list=4%, signal=35%
48	WANG_LSD1_TARGETS_UP		14	0.67	1.86	0.004	0.049	0.859	1048	tags=64%, list=10%, signal=71%
49	MCLACHLAN_DENTAL_CARIES_UP		192	0.38	1.85	0	0.052	0.879	2679	tags=38%, list=25%, signal=50%
50	DANG_MYC_TARGETS_UP		115	0.42	1.85	0	0.052	0.879	3139	tags=56%, list=29%, signal=77%

na_neg

	GS follow link to MSigDB	GS DETAILS	SIZE	ES	NES	NOM p-val	FDR q-val	FWER p-val	RANK AT MAX	LEADING EDGE
1	RAGHAVACHARI_PLATELET_SPECIFIC_GENES	Details ...	69	-0.69	-2.79	0	0	0	2495	tags=84%, list=23%, signal=108%
2	WIERENGA_STAT5A_TARGETS_DN	Details ...	179	-0.52	-2.44	0	0	0	2430	tags=54%, list=22%, signal=69%
3	ROSS_AML_OF_FAB_M7_TYPE	Details ...	58	-0.52	-2.02	0	0.048	0.156	2315	tags=59%, list=21%, signal=74%
4	VALK_AML_CLUSTER_7	Details ...	21	-0.63	-1.92	0	0.16	0.537	1280	tags=48%, list=12%, signal=54%
5	MOLENAAR_TARGETS_OF_CCND1_AND_CDK4_DN	Details ...	48	-0.5	-1.9	0	0.165	0.632	1251	tags=33%, list=11%, signal=37%

6	GAVIN_FOXP3_TARGETS_CLUSTER_P6	Details ...	67	-0.47	-1.9	0	0.143	0.65	1549	tags=34%, list=14%, signal=40%
7	KUNINGER_IGF1_VS_PDGFB_TARGETS_DN	Details ...	32	-0.55	-1.89	0.002	0.133	0.685	947	tags=41%, list=9%, signal=44%
8	GNATENKO_PLATELET_SIGNATURE	Details ...	43	-0.52	-1.89	0.002	0.117	0.686	2552	tags=60%, list=23%, signal=79%
9	TENEDINI_MEGAKARYOCYTE_MARKERS	Details ...	57	-0.48	-1.89	0	0.106	0.687	2497	tags=51%, list=23%, signal=66%
10	CHUNG_BLISTER_CYTOTOXICITY_DN	Details ...	43	-0.51	-1.87	0	0.123	0.762	3253	tags=63%, list=30%, signal=89%
11	KYNG_DNA_DAMAGE_BY_4NQO_OR_GAMMA_RADIATION	Details ...	13	-0.67	-1.82	0	0.194	0.929	1175	tags=46%, list=11%, signal=52%
12	HOLLEMAN_VINCRISTINE_RESISTANCE_ALL_UP	Details ...	25	-0.56	-1.81	0.004	0.21	0.967	2336	tags=44%, list=21%, signal=56%
13	LIU_VAV3_PROSTATE_CARCINOGENESIS_DN	Details ...	9	-0.73	-1.8	0.004	0.225	0.981	1069	tags=56%, list=10%, signal=62%
