# Supplementary material for: Comprehensive RNA dataset of tissue and plasma from patients with esophageal cancer or precursor lesions
Source: Sci Data. 2022 Mar 14;9:86. doi: 10.1038/s41597-022-01176-x (PMC8921197; doi:10.1038/s41597-022-01176-x)
Supplement: Supplementary file 4 — Supplementary Table 4 [file 41597_2022_1176_MOESM4_ESM.pdf]

Supplementary Table 4: Variant analysis results of all plasma samples.

sample ID	chromosome	location	mutation	genotype	read depth (wildtype, alternative allel)	total read depth	Cosmic database
**ID20_EAC**	chr12	120212502	G>A	0/1	1,3	4	NA
**ID29_EAC**	chr4	153705181	C>T	1/1	0,2	2	NA
**ID2_HGD**	chr4	57110068	T>C	1/1	0,2	2	NA
	chr8	17967103	C>T	0/1	3,2	5	NA
**ID5_HGD**	chr1	171670604	G>A	0/1	1,6	7	NA
	chr4	38125007	T>C	0/1	2,2	4	NA
	chr8	27605281	G>A	0/1	216,210	426	COSM5564582 (prostate)
**ID25_HGD**	chr22	31104425	C>T	1/1	0,2	2	NA
**ID26_HGD**	chr2	233125868	C>T	1/1	0,3	3	NA
	chrM	16126	T>C	1/1	0,5	5	NA
**ID39_HGD**	chr6	109453742	C>T	0/1	2,4	6	NA
**ID1_NDB**	chr1	147178275	C>T	0/1	1,7	8	NA
	chr1	227072270	A>C	0/1	2,5	7	NA
	chr4	87372750	C>T	0/1	7,11	18	NA
	chr9	120415109	C>T	0/1	3,4	7	NA
	chr17	80351781	A>G	0/1	3,4	7	NA
	chr19	12663438	G>A	0/1	2,4	6	COSM5493837 (cervix,biliary tract)
	chr20	48985538	G>A	0/1	1,3	4	NA
**ID19_NDB**	chr2	33347385	T>C	0/1	48,55	103	NA
	chr10	22591708	T>C	0/1	203,161	364	NA
**ID35_NDB**	chr10	102812743	C>T	0/1	3,2	5	NA
	chr13	49482808	A>G	0/1	10,8	18	NA
**ID37_NDB**	chr2	55603992	C>T	0/1	8,3	11	COSM5756079 (large intestine)
	chr3	148991619	C>A	0/1	2,3	5	NA
