# Supplementary material for: Comprehensive RNA dataset of tissue and plasma from patients with esophageal cancer or precursor lesions
Source: Sci Data. 2022 Mar 14;9:86. doi: 10.1038/s41597-022-01176-x (PMC8921197; doi:10.1038/s41597-022-01176-x)
Supplement: Supplementary file 5 — Supplementary Table 5 [file 41597_2022_1176_MOESM5_ESM.pdf]

Supplementary Table 5a: column descriptions for Supplementary Tables 5b-5e.

Columns description	
**Column**	**Description**
**Gene 1 symbol (5' end fusion partner)**	Gene symbol of the 5' end fusion partner
**Gene 2 symbol (3' end fusion partner)**	Gene symbol of the 3' end fusion partner
**Gene 1 ID (5' end fusion partner)**	Ensembl gene id of the 5' end fusion partner
**Gene 2 ID (3' end fusion partner)**	Ensembl gene id of the 3' end fusion partner
**Exon 1 ID (5' end fusion partner)**	Ensembl exon id of the 5' end fusion exon-exon junction
**Exon 2 ID (3' end fusion partner)**	Ensembl exon id of the 3' end fusion exon-exon junction
**Fusion point for gene 1 (5' end fusion partner)**	Chromosomal position of the 5' end of fusion junction (chromosome:position:strand); 1-based coordinate
**Fusion point for gene 2 (3' end fusion partner)**	Chromosomal position of the 3' end of fusion junction (chromosome:position:strand); 1-based coordinate
**Spanning_pairs**	Count of pair-end reads supporting the fusion
**Spanning_unique_reads**	Count of unique reads (i.e. unique mapping positions) mapping on the fusion junction (also known as split reads). Shortly, here are counted all the reads which map on fusion junction minus the PCR duplicated reads.
**Longest_anchor_found**	Longest anchor (hangover) found among the unique reads mapping on the fusion junction
**Fusion_finding_method**	Aligning method used for mapping the reads and finding the fusion genes. Here are two methods used which are: (i) **BOWTIE** = only Bowtie aligner is used for mapping the reads on the genome and exon-exon fusion junctions, (ii) **BOWTIE+BLAT** = Bowtie aligner is used for mapping reads on the genome and BLAT is used for mapping reads for finding the fusion junction, (iii) **BOWTIE+STAR** = Bowtie aligner is used for mapping reads on the genome and STAR is used for mapping reads for finding the fusion junction, (iv) **BOWTIE+BOWTIE2** = Bowtie aligner is used for mapping reads on the genome and Bowtie2 is used for mapping reads for finding the fusion junction, and (v) **BOWTIE+BWA** = Bowtie aligner is used for mapping reads on the genome and Bowtie2 is used for mapping reads for finding the fusion junction.
**Fusion_sequence**	The inferred fusion junction (the asterisk sign marks the junction point)
**Fusion_description**	Type of the fusion gene (see the Table 2)
**Counts_of_common_mapping_reads**	Count of reads mapping simultaneously on both genes which form the fusion gene. This is an indication how similar are the DNA/RNA sequences of the genes forming the fusion gene (i.e. what is their homology because highly homologous genes tend to appear show as candidate fusion genes). In case of completely different sequences of the genes involved in forming a fusion gene then here it is expected to have the value zero.
**Predicted effect**	Predicted effect of the candidate fusion gene using the annotation from Ensembl database. This is shown in format effect_gene_1/effect_gene_2, where the possible values for effect_gene_1 or effect_gene_2 are: intergenic, intronic, exonic(no-known-CDS), UTR, CDS(not-reliable-start-or-end), CDS(truncated), or CDS(complete). In case that the fusion junction for both genes is within their CDS (coding sequence) then only the values in-frame or out-of-frame will be shown.

Fusion description labels		
Labels in red have a high probability of being a false positive. Labels in orange have a low/medium probability of being false positives.		
**Label**	**Description**	**Source**
**1000genomes**	fusion gene has been seen in a healthy sample. It has been found in RNA-seq data from some samples from 1000 genomes project *A candidate fusion gene having this label has a very high probability of being a false positive.*	http://dx.doi.org/10.1371/journal.pone.0104567
**18cancers**	fusion gene found in a RNA-seq dataset of 18 types of cancers from 600 tumor samples	http://dx.doi.org/10.1073/pnas.1606220113
**adjacent**	both genes forming the fusion are adjacent on the genome (i.e. same strand and there is no other genes situated between them on the same strand)	
**antisense**	one or both genes is a gene coding for antisense RNA	
**banned**	fusion gene is on a list of known false positive fusion genes. These were found with very strong supporting data in healthy samples. *A candidate fusion gene having this label has a very high probability of being a false positive.*	
**bodymap2**	fusion gene is on a list of known false positive fusion genes. It has been found in healthy human samples collected from 16 organs from Illumina BodyMap2 RNA-seq database *A candidate fusion gene having this label has a very high probability of being a false positive.*	http://www.ebi.ac.uk/arrayexpress/experiments/E-MTAB-513/
**cacg**	known conjoined genes (that is fusion genes found in samples from healthy patients) from the CACG database (please see CACG database for more information). *A candidate fusion gene having this label has a very high probability of being a false positive in case that one looks for fusion genes specific to a disease.*	http://cgc.kribb.re.kr/map/
**cell_lines**	known fusion gene from paper: C. Klijn et al., A comprehensive transcriptional portrait of human cancer cell lines, Nature Biotechnology, Dec. 2014,	http://dx.doi.org/10.1038/nbt.3080
**cgp**	known fusion gene from the CGP database	http://www.sanger.ac.uk/genetics/CGP/Census/
**chimerdb2**	known fusion gene from the ChimerDB 2 database	http://ercsb.ewha.ac.kr/FusionGene/

**chimerdb3kb**	known fusion gene from the ChimerDB 3 KB (literature curration) database	http://ercsb.ewha.ac.kr/FusionGene/
**chimerdb3pub**	known fusion gene from the ChimerDB 3 PUB (PubMed articles) database	http://ercsb.ewha.ac.kr/FusionGene/
**chimerdb3seq**	known fusion gene from the ChimerDB 3 SEQ (TCGA) database	http://ercsb.ewha.ac.kr/FusionGene/
**conjoing**	known conjoined genes (that is fusion genes found in samples from healthy patients) from the [ConjoinG database (please use ConjoinG database for more information regarding the fusion gene). *A candidate fusion gene having this label has a very high probability of being a false positive in case that one looks for fusion genes specific to a disease.*	http://metasystems.riken.jp/conjoing/
**cosmic**	known fusion gene from the COSMIC database (please use COSMIC database for more information regarding the fusion gene)	http://cancer.sanger.ac.uk/cancergenome/projects/cosmic/
**cta**	one gene or both genes is CTA gene (that is that the gene name starts with CTA-). *A candidate fusion gene having this label has a very high probability of being a false positive.*	
**ctb**	one gene or both genes is CTB gene (that is that the gene name starts with CTB-). *A candidate fusion gene having this label has a very high probability of being a false positive.*	
**ctc**	one gene or both genes is CTC gene (that is that the gene name starts with CTC-). *A candidate fusion gene having this label has a very high probability of being a false positive.*	
**ctd**	one gene or both genes is CTD gene (that is that the gene name starts with CTD-). *A candidate fusion gene having this label has a very high probability of being a false positive.*	
**distance1000bp**	both genes are on the same strand and they are less than 1,000 bp apart. *A candidate fusion gene having this label has a very high probability of being a false positive.*	
**distance100kbp**	both genes are on the same strand and they are less than 100,000 bp apart. *A candidate fusion gene having this label has a higher probability than expected of being a false positive.*	
**distance10kbp**	both genes are on the same strand and they are less than 10,000 bp apart. *A candidate fusion gene having this label has a higher probability than expected of being a false positive.*	
**duplicates**	both genes involved in the fusion gene are paralog for each other. For more see Duplicated Genes Database (DGD) database . *A candidate fusion gene having this label has a higher probability than expected of being a false positive.*	http://dgd.genouest.org/
**exon-exon**	the fusion junction point is exactly at the known exon's borders of both genes forming the candidate fusion	
**ensembl_fully_overlapping**	the genes forming the fusion gene are fully overlapping according to Ensembl database. *A candidate fusion gene having this label has a very high probability of being a false positive.*	
**ensembl_partially_overlapping**	the genes forming the fusion gene are partially overlapping (on same strand or on different strands) according the Ensembl database. *A candidate fusion gene having this label has a good probability of being a false positive.*	
**ensembl_same_strand_overlapping**	the genes forming the fusion gene are fully/partially overlapping and are both on the same strand according to Ensembl database. *A candidate fusion gene having this label has a very high probability of being a false positive (this is most likely and alternative splicing event).*	
**fragments**	the genes forming the fusion are supported by only and only one fragment of RNA. *A candidate fusion gene having this label has a medium probability of being a false positive.*	
**gliomas**	fusion gene found in a RNA-seq dataset of 272 glioblastoms	http://dx.doi.org/10.1101/gr.165126.113
**gtex**	fusion gene has been seen in a healthy sample. It has been found in GTEx database of healthy tissues thru FusionAnnotator). *A candidate fusion gene having this label has a very high probability of being a false positive.*	http://www.gtexportal.org/home/; https://github.com/FusionAnnotator/FusionAnnotator
**healthy**	fusion gene has been seen in a healthy sample. These have been found in healthy samples but the support for them is less strong (i.e. paired reads were found to map on both genes but no fusion junction was found) than in the case of **banned** label (i.e. it showed up in file preliminary list of candidate fusion genes). Also genes which have some degree of sequence similarity may show up marked like this.*A candidate fusion gene having this label has a small probability of being a false positive in case that one looks for fusion genes specific to a disease.*	
**hpa**	fusion gene has been seen in a healthy sample. It has been found in RNA-seq database of 27 healthy tissues. *A candidate fusion gene having this label has a very high probability of being a false positive.*	http://dx.doi.org/10.1074/mcp.M113.035600
**known**	fusion gene which has been previously reported or published in scientific articles/reports/books/abstracts/databases indexed by Google, Google Scholar, PubMed, etc. This label has only the role to answer with YES or NO the question "has ever before a given (candidate) fusion gene been published or reported?". This label does not have in anyway the role to provide the original references to the original scientific articles/reports/books/abstracts/databases for a given fusion gene.	http://www.google.com/; http://scholar.google.com/; http://www.ncbi.nlm.nih.gov/pubmed
**lincrna**	one or both genes is a lincRNA	
**matched-normal**	candidate fusion gene (which is supported by paired reads mapping on both genes and also by reads mapping on the junction point) was found also in the matched normal sample given as input to the command line option '--normal'	
**metazoa**	one or both genes is a metazoa_srp gene	http://www.genecards.org/index.php?path=/Search/keyword/metazoa_srp
**mirna**	one or both genes is a miRNA	
**mt**	one or both genes are situated on mitochondrion. *A candidate fusion gene having this label has a very high probability of being a false positive.*	

**non_cancer_tissues**	fusion gene which has been previously reported/found in non-cancer tissues and cell lines in Babiceanu et al, Recurrent chimeric fusion RNAs in non-cancer tissues and cells, Nucl. Acids Res. 2016. These are considered as non-somatic mutation and therefore they may be skipped and not reported.	http://nar.oxfordjournals.org/content/early/2016/02/01/nar.gkw032.abstract
**non_tumor_cells**	fusion gene which has been previously reported/found in non-tumor cell lines, like for example HEK293. These are considered as non-somatic mutation and therefore may be skipped and not reported.	
**no_protein**	one or both genes have no known protein product	
**oesophagus**	fusion gene found in a oesophageal tumors from TCGA samples	http://dx.doi.org/10.1038/nature20805
**oncogene**	one gene or both genes are a known oncogene according to ONGENE database	https://doi.org/10.1016/j.jgg.2016.12.004
**cancer**	one gene or both genes are cancer associated according to Cancer Gene database	http://www.bushmanlab.org/links/genelists
**tumor**	one gene or both genes are proto-oncogene or tumor suppresor gene according to UniProt database	http://www.uniprot.org
**pair_pseudo_genes**	one gene is the other's pseudogene. *A candidate fusion gene having this label has a very high probability of being a false positive.*	
**pancreases**	known fusion gene found in pancreatic tumors from article: P. Bailey et al., Genomic analyses identify molecular subtypes of pancreatic cancer, Nature, Feb. 2016,	http://dx.doi.org/110.1038/nature16965
**paralogs**	both genes involved in the fusion gene are paralog for each other (most likely this is a false positive fusion gene). *A candidate fusion gene having this label has a very high probability of being a false positive.*	
**multi**	one of the genes of both have multi-mapping reads mapping (which map simultaneously also on other gene/genes	
**partial-matched-normal**	candidate fusion gene (which is supported by paired reads mapping on both genes but no reads were found which map on the junction point) was found also in the matched normal sample given as input to the command line option '--normal'. This is much weaker than *matched-normal* .	
**prostates**	known fusion gene found in 150 prostate tumors RNAs from paper: D. Robison et al, Integrative Clinical Genomics of Advanced Prostate Cancer, Cell, Vol. 161, May 2015	http://dx.doi.org/10.1016/j.cell.2015.05.001
**pseudogene**	one or both of the genes is a pseudogene	
**readthrough**	the fusion gene is a readthrough event (that is both genes forming the fusion are on the same strand and there is no known gene situated in between); Please notice, that many of readthrough fusion genes might be false positive fusion genes due to errors in Ensembl database annotation (for example, one gene is annotated in Ensembl database as two separate genes). *A candidate fusion gene having this label has a high probability of being a false positive.*	
**refseq_fully_overlapping**	the genes forming the fusion gene are fully overlapping according to RefSeq NCBI database. *A candidate fusion gene having this label has a very high probability of being a false positive.*	
**refseq_partially_overlapping**	the genes forming the fusion gene are partially overlapping (on same strand or on different strands) according the RefSeq NCBI. *A candidate fusion gene having this label has a good probability of being a false positive.*	
**refseq_same_strand_overlapping**	the genes forming the fusion gene are fully/partially overlapping and are both on the same strand according to RefSeq NCBI database. *A candidate fusion gene having this label has a very high probability of being a false positive (this is most likely and alternative splicing event).*	
**ribosomal**	one or both gene is a gene encoding for ribosomal protein	
**rp11**	one gene or both genes is RP11 gene (that is that the gene name starts with **RP11-**). *A candidate fusion gene having this label has a very high probability of being a false positive.*	
**rp**	one gene or both genes is RP?? gene (that is that the gene name starts with **RP??-**) where ? is a digit. *A candidate fusion gene having this label has a very high probability of being a false positive.*	
**rrna**	one or both genes is a rRNA. *A candidate fusion gene having this label has a very high probability of being a false positive.*	
**short_distance**	both genes are on the same strand and they are less than X bp apart, where X is set using the option '--dist-fusion' and by default it is 200,000 bp. *A candidate fusion gene having this label has a higher probability than expected of being a false positive.*	
**similar_reads**	both genes have the same reads which map simultaneously on both of them (this is an indicator of how similar are the sequences of both genes; ideally this should be zero or as close to zero as possible for a real fusion). *A candidate fusion gene having this label has a very high probability of being a false positive.*	
**similar_symbols**	both genes have the same or very similar gene names (for example: RP11ADF.1 and RP11ADF.2). *A candidate fusion gene having this label has a very high probability of being a false positive.*	
**snorna**	one or both genes is a snoRNA	
**snrna**	one or both genes is a snRNA	
**tcga**	known fusion gene from the TCGA database	https://tcga-data.nci.nih.gov/tcga/
**ticdb**	known fusion gene from the TICdb] database	http://www.unav.es/genetica/TICdb/
**trna**	one or both genes is a tRNA	
**ucsc_fully_overlapping**	the genes forming the fusion gene are fully overlapping according to UCSC database. *A candidate fusion gene having this label has a very high probability of being a false positive.*	

**ucsc_partially_overlapping**	the genes forming the fusion gene are partially overlapping (on same strand or on different strands) according the UCSC database. *A candidate fusion gene having this label has a good probability of being a false positive.</i>
**ucsc_same_strand_overlapping**	the genes forming the fusion gene are fully/partially overlapping and are both on the same strand according to UCSC database. *A candidate fusion gene having this label has a very high probability of being a false positive (this is most likely and alternative splicing event).</i>
**yrna**	one or both genes is a Y RNA

Supplementary Table 5b: all fusion genes identified in the healthy tissue samples (EAC, HGD and NDB). Fusion gene pairs indicated in red are likely false positives, based on fusion description (see Supplementary Table 5a) or high number of common mapping reads.

sample ID	gene 1 symbol (5' end fusion partner)	gene 2 symbol (3' end fusion partner)	fusion_description	counts_of_common_mapping_reads	spanning_pairs	spanning_unique_reads	longest_anchor_found	fusion_finding_method	fusion_point_for_gene_1(5' end_fusion_partner)	fusion_point_for_gene_2(3' end_fusion_partner)	gene_1_id(5' end_fusion_partner)
ID20_EAC	KRT13	ANXA1	tcga,gtex,non_cancer_tissues,cancer,m44,exon-exon	0	46	3	21	BOWTIE	17:41500982:-	9:73157654:+	ENSG00000171401
	KRT13	ANXA1	tcga,gtex,non_cancer_tissues,cancer,m44,exon-exon	0	46	2	20	BOWTIE	17:41500981:-	9:73157654:+	ENSG00000171401
	SPRR3	ANXA1	gtex,cancer,exon-exon	0	23	3	21	BOWTIE	1:153003855:+	9:73157654:+	ENSG00000163209
	SPRR3	ANXA1	gtex,cancer,exon-exon	0	23	2	20	BOWTIE	1:153003856:+	9:73157654:+	ENSG00000163209
	RMND5A	ANAPC1	banned,known,bodymap2,hpa,m0,multi,exon-exon	0	8	7	30	BOWTIE	2:86741069:+	2:111822600:-	ENSG00000153561
	KRT4	PPL	gtex,non_cancer_tissues	0	7	2	24	BOWTIE+STAR	12:52806552:-	16:4950251:-	ENSG00000170477
	ANXA2	S100A9	cancer,exon-exon	0	4	2	23	BOWTIE	15:60394574:-	1:153360644:+	ENSG00000182718
	KRT13	PKM	gtex,cancer	0	4	4	18	BOWTIE+STAR	17:41500991:-	15:72205126:-	ENSG00000171401
	AC006210.2	RDX	no_protein,pseudogene	25	3	2	23	BOWTIE+STAR	X:27517981:+	11:110264810:-	ENSG00000238247
	NPEPPS	TBC1D3	banned,known,oncogene,bodymap2,hpa,gtex,18cancers,tumor,m0,multi,exon-exon	0	3	12	29	BOWTIE	17:47592545:+	17:38191030:-	ENSG00000141279
	SARNP	AC073063.1	no_protein,pseudogene,cancer	44	3	3	33	BOWTIE+STAR	12:55800593:-	7:99443293:-	ENSG00000205323
	DUS4L	BCAP29	banned,known,adjacent,healthy,bodymap2,hpa,chimerdb3pub,1K<gap<10K,readthrough,exon-exon	0	2	2	27	BOWTIE	7:107576592:+	7:107580759:+	ENSG00000105865
	BPTF	LRRC37A2	banned,known,hpa,m0,multi,exon-exon	0	1	2	21	BOWTIE	17:67826337:+	17:46517362:+	ENSG00000171634
ID29_EAC	KRT13	ANXA1	tcga,gtex,non_cancer_tissues,cancer,exon-exon	0	27	2	20	BOWTIE	17:41500982:-	9:73157654:+	ENSG00000171401
	KRT13	ANXA1	tcga,gtex,non_cancer_tissues,cancer,exon-exon	0	27	2	19	BOWTIE	17:41500981:-	9:73157654:+	ENSG00000171401
	NPEPPS	TBC1D3	banned,known,oncogene,bodymap2,hpa,gtex,18cancers,tumor,m0,multi,exon-exon	0	12	15	30	BOWTIE	17:47592545:+	17:38191030:-	ENSG00000141279
	RMND5A	ANAPC1	banned,known,bodymap2,hpa,m0,multi,exon-exon	0	12	7	29	BOWTIE	2:86741069:+	2:111822600:-	ENSG00000153561
	CSTB	AHNAK	cancer	0	7	2	23	BOWTIE+STAR	21:43772872:-	11:62481115:-	ENSG00000160213
	PRIM1	NACA	banned,known,adjacent,conjoing,healthy,bodymap2,hpa,1000genomes,cancer,m4,gap<1K,readthrough,exon-exon	0	5	2	27	BOWTIE	12:56734147:-	12:56714687:-	ENSG00000198056
	SCNN1A	TNFRSF1A	banned,known,adjacent,conjoing,healthy,bodymap2,non_tumor_cells,hpa,cancer,1K<gap<10K,readthrough	0	4	2	28	BOWTIE+STAR	12:6348727:-	12:6342025:-	ENSG00000111319
	AC087386.1	IGK@	no_protein,lincrna	0	3	2	37	BOWTIE+STAR	15:20139242:-	2:90254788:+	ENSG00000258410
	CRLF2	GBP6	oncogene,cancer,m2	3	3	6	38	BOWTIE+STAR	X:1224767:-	1:89385616:+	ENSG00000205755
	GBP6	CRLF2	oncogene,cancer,m2	3	3	5	32	BOWTIE+STAR	1:89385768:+	X:1141025:-	ENSG00000183347
	GBP6	CRLF2	oncogene,cancer,m2	3	3	5	32	BOWTIE+STAR	1:89385768:+	X:1240145:-	ENSG00000183347
	GBP6	CRLF2	oncogene,cancer,m2	3	3	5	32	BOWTIE+STAR	1:89385768:+	X:1145567:-	ENSG00000183347
	GBP6	CRLF2	oncogene,cancer,m2	3	3	5	32	BOWTIE+STAR	1:89385768:+	X:1131262:-	ENSG00000183347
	IGK@	TMEM128		0	3	3	38	BOWTIE+BOWTIE2;BOWTIE+STAR	2:90241463:-	4:4237935:-	ENSG09000000012
	IGK@	AC087386.1	no_protein,lincrna	0	3	3	26	BOWTIE+STAR	2:90255789:+	15:20138256:-	ENSG09000001012
	NAIP	OCLN	banned,known,bodymap2,hpa,1000genomes,m0,multi,exon-exon	0	3	3	25	BOWTIE	5:70979869:-	5:69534694:+	ENSG00000249437
	KANSL1	ARL17A	banned,known,healthy,bodymap2,hpa,gtex,18cancers,chimerdb3seq,m0,multi,exon-exon	0	2	3	26	BOWTIE	17:46094560:-	17:46570869:-	ENSG00000120071
	KANSL1	ARL17B	banned,known,healthy,bodymap2,hpa,chimerdb3seq,m0,multi,10K<gap<100K,exon-exon	0	2	3	26	BOWTIE	17:46094560:-	17:46352930:-	ENSG00000120071
	KRT13	CLTA	exon-exon	0	2	2	20	BOWTIE	17:41500982:-	9:36304067:+	ENSG00000171401
	KRT13	CLTA	exon-exon	0	2	2	19	BOWTIE	17:41500981:-	9:36304067:+	ENSG00000171401
	RASAL2	ANXA1	cancer,exon-exon	0	2	19	30	BOWTIE	1:178123815:+	9:73158522:+	ENSG00000075391
	BPTF	LRRC37A2	banned,known,hpa,m0,multi,exon-exon	0	1	3	28	BOWTIE	17:67826337:+	17:46517362:+	ENSG00000171634

ID30_EAC	KRT13	ANXA1	tcga,gtex,non_cancer_tissues,cancer,m57,exon-exon	0	58	2	19	BOWTIE	17:41500982:-	9:73157654:+	ENSG00000171401
	SPRR3	ANXA1	gtex,cancer,exon-exon	0	16	3	20	BOWTIE	1:153003855:+	9:73157654:+	ENSG00000163209
	SPRR3	ANXA1	gtex,cancer,exon-exon	0	16	2	19	BOWTIE	1:153003856:+	9:73157654:+	ENSG00000163209
	NPEPPS	TBC1D3	banned,known,oncogene,bodymap2,hpa,gtex,18cancers,tumor,m0,multi,exon-exon	0	10	14	29	BOWTIE	17:47592545:+	17:38191030:-	ENSG00000141279
	RMND5A	ANAPC1	banned,known,bodymap2,hpa,m0,multi,exon-exon	0	8	6	29	BOWTIE	2:86741069:+	2:111822600:-	ENSG00000153561
	SMG1	NPIPB5	banned,known,bodymap2,hpa,m0,multi	0	6	3	22	BOWTIE+STAR	16:18858211:-	16:22513522:+	ENSG00000157106
	SMG1	NPIPB5	banned,known,bodymap2,hpa,m0,multi,exon-exon	0	6	2	28	BOWTIE	16:18858170:-	16:22513522:+	ENSG00000157106
	KRT13	GTF2I	exon-exon	0	4	2	19	BOWTIE	17:41500982:-	7:74657667:+	ENSG00000171401
	COQ8B	NUMBL	banned,known,adjacent,healthy,bodymap2,hpa,gap<1K,readthrough	0	3	2	34	BOWTIE+STAR	19:40691592:-	19:40686995:-	ENSG00000123815
	KRT13	PDIA3	exon-exon	0	3	2	19	BOWTIE	17:41500982:-	15:43746392:+	ENSG00000171401
	KRT4	GABRP		0	3	2	22	BOWTIE+STAR	12:52813845:-	5:170785243:+	ENSG00000170477
	KRT6A	AHNAK	gtex,m2	0	3	4	21	BOWTIE+STAR	12:52487186:-	11:62461017:-	ENSG00000205420
	PITX1	ANXA1	cancer,exon-exon	0	3	3	22	BOWTIE	5:135028553:-	9:73157654:+	ENSG00000069011
	ANXA1	RPL30	ribosomal,cancer,exon-exon	0	2	4	21	BOWTIE	9:73151924:+	8:98025738:-	ENSG00000135046
	FBXO25	FAM157B	banned,known,no_protein,pseudogene,bodymap2,hpa,m0,multi,exon-exon	0	2	2	21	BOWTIE	8:435707:+	9:138243620:+	ENSG00000147364
	KRT13	HECTD1	exon-exon	0	2	2	19	BOWTIE	17:41500982:-	14:31136838:-	ENSG00000171401
ID43_EAC	*excluded from fusion gene analysis (see 'Methods' section)*										
ID2_HGD	ARPC2	ANXA1	cancer,exon-exon	0	3	4	23	BOWTIE	2:218254108:+	9:73157654:+	ENSG00000163466
	ARPC2	ANXA1	cancer,exon-exon	0	3	4	21	BOWTIE	2:218254110:+	9:73157654:+	ENSG00000163466
	FAM157A	S100A9	no_protein,pseudogene,cancer,m2,exon-exon	0	3	2	25	BOWTIE	3:198210368:+	1:153360644:+	ENSG00000236438
	SARNP	AC073063.1	no_protein,pseudogene,cancer	30	3	2	32	BOWTIE+STAR	12:55800593:-	7:99443293:-	ENSG00000205323
	NRBP1	ANXA1	cancer,exon-exon	0	2	19	30	BOWTIE	2:27431991:+	9:73158522:+	ENSG00000115216
	PITX1	ANXA1	cancer,exon-exon	0	2	3	20	BOWTIE	5:135028553:-	9:73157654:+	ENSG00000069011
	SPRR3	BNIPL	exon-exon	0	2	3	22	BOWTIE	1:153003855:+	1:151045559:+	ENSG00000163209
	SPRR3	BNIPL	exon-exon	0	2	2	21	BOWTIE	1:153003856:+	1:151045559:+	ENSG00000163209
	SPRR3	GTF2I	exon-exon	0	2	2	21	BOWTIE	1:153003856:+	7:74657667:+	ENSG00000163209
	SPRR3	GTF2I	exon-exon	0	2	2	18	BOWTIE	1:153003855:+	7:74657667:+	ENSG00000163209
	SPRR3	PILRB	exon-exon	0	2	2	18	BOWTIE	1:153003855:+	7:100358015:+	ENSG00000163209
	SPRR3	STAG3L5P-PVRIG2P-PILRB	no_protein,exon-exon	0	2	3	22	BOWTIE	1:153003855:+	7:100336114:+	ENSG00000163209
	SPRR3	STAG3L5P-PVRIG2P-PILRB	no_protein,exon-exon	0	2	2	21	BOWTIE	1:153003856:+	7:100336114:+	ENSG00000163209
	TMPRSS11B	CNOT1	exon-exon	0	2	9	29	BOWTIE	4:68234463:-	16:58624794:-	ENSG00000185873
	DUS4L	BCAP29	banned,known,adjacent,healthy,bodymap2,hpa,chimerdb3pub,1K<gap<10K,readthrough,exon-exon	0	1	3	28	BOWTIE	7:107576592:+	7:107580759:+	ENSG00000105865
	KANSL1	ARL17A	banned,known,healthy,bodymap2,hpa,gtex,18cancers,chimerdb3seq,m0,multi,exon-exon	0	1	3	28	BOWTIE	17:46094560:-	17:46570869:-	ENSG00000120071
	KANSL1	ARL17B	banned,known,healthy,bodymap2,hpa,chimerdb3seq,m0,multi,10K<gap<100K,exon-exon	0	1	3	28	BOWTIE	17:46094560:-	17:46352930:-	ENSG00000120071
	SLC22A20	HORMAD1	banned,known,no_protein,pseudogene,hpa,m0,multi,exon-exon	0	1	2	24	BOWTIE	11:65239480:+	1:150719538:-	ENSG00000197847
	KRT13	ANXA1	tcga,gtex,non_cancer_tissues,cancer,exon-exon	0	33	3	19	BOWTIE	17:41500982:-	9:73157654:+	ENSG00000171401
	KRT13	ANXA1	tcga,gtex,non_cancer_tissues,cancer,exon-exon	0	33	2	18	BOWTIE	17:41500981:-	9:73157654:+	ENSG00000171401
	SPRR3	ANXA1	gtex,cancer,exon-exon	0	32	3	19	BOWTIE	1:153003855:+	9:73157654:+	ENSG00000163209
	SPRR3	ANXA1	gtex,cancer,exon-exon	0	32	2	18	BOWTIE	1:153003856:+	9:73157654:+	ENSG00000163209
	NPEPPS	TBC1D3	banned,known,oncogene,bodymap2,hpa,gtex,18cancers,tumor,m0,multi,exon-exon	0	19	15	30	BOWTIE;BOWTIE+STAR	17:47592545:+	17:38191030:-	ENSG00000141279
	AC087386.1	IGK@	no_protein,lincrna	0	10	2	37	BOWTIE+STAR	15:20137892:-	2:90256400:+	ENSG00000258410
	IGK@	AC087386.1	no_protein,lincrna	0	10	5	37	BOWTIE+STAR	2:90255789:+	15:20138256:-	ENSG09000001012
	IGK@	AC087386.1	no_protein,lincrna	0	10	2	35	BOWTIE+STAR	2:90257295:+	15:20136698:-	ENSG09000001012
	SMG1	NPIPB5	banned,known,bodymap2,hpa,m0,multi	0	10	4	36	BOWTIE+STAR	16:18858211:-	16:22513522:+	ENSG00000157106

	SMG1	NPIPB5	banned,known,bodymap2,hpa,m0,multi,exon-exon	0	10	2	28	BOWTIE	16:18858170:-	16:22513522:+	ENSG00000157106
	IGH@	MIR100HG	banned,known,oncogene,hpa,m0,multi	0	8	2	28	BOWTIE+STAR	14:106292748:+	11:121821868:-	ENSG09000000016
	RMND5A	ANAPC1	banned,known,bodymap2,hpa,m0,multi,exon-exon	0	6	9	28	BOWTIE;BOWTIE+STAR	2:86741069:+	2:111822600:-	ENSG00000153561
	DDX17	ERO1A		0	5	2	27	BOWTIE+STAR	22:38500048:-	14:52643627:-	ENSG00000100201
	SPRR2D	CRNN	m3	0	4	2	24	BOWTIE+STAR	1:153039675:-	1:152412246:-	ENSG00000163216
ID5_HGD	AHNAK	ERO1A		0	3	3	26	BOWTIE+STAR	11:62510616:-	14:52643627:-	ENSG00000124942
	C1GALT1	AC005532.1	no_protein,adjacent,1K<gap<10K,readthrough	0	3	2	34	BOWTIE+BOWTIE2;BOWTIE+STAR	7:7243750:+	7:7277585:+	ENSG00000106392
	DSC3	AC245884.4	pseudogene,m2	1	3	7	38	BOWTIE+STAR	18:30992181:-	19:54386454:+	ENSG00000134762
	SLC22A20	HORMAD1	banned,known,no_protein,pseudogene,hpa,m0,multi	0	3	2	28	BOWTIE+BOWTIE2	11:65239477:+	1:150719537:-	ENSG00000197847
	ACTB	ERO1A	cancer,exon-exon	0	2	4	25	BOWTIE	7:5540676:-	14:52661304:-	ENSG00000075624
	ACTB	ERO1A	cancer,exon-exon	0	2	3	24	BOWTIE	7:5540676:-	14:52658150:-	ENSG00000075624
	CD55	ANXA1	cancer,exon-exon	0	2	2	18	BOWTIE	1:207367487:+	9:73158522:+	ENSG00000196352
	KRT13	CLTA	exon-exon	0	2	4	25	BOWTIE	17:41500982:-	9:36304067:+	ENSG00000171401
	KRT13	CLTA	exon-exon	0	2	3	24	BOWTIE	17:41500981:-	9:36304067:+	ENSG00000171401
	KRT13	ECHDC2	exon-exon	0	2	2	18	BOWTIE	17:41500981:-	1:52912225:-	ENSG00000171401
	KRT13	HEBP2	exon-exon	0	2	4	25	BOWTIE	17:41500982:-	6:138403531:+	ENSG00000171401
	KRT13	HEBP2	exon-exon	0	2	3	24	BOWTIE	17:41500981:-	6:138403531:+	ENSG00000171401
	KRT13	HOOK2	exon-exon	0	2	4	25	BOWTIE	17:41500982:-	19:12773253:-	ENSG00000171401
	KRT13	HOOK2	exon-exon	0	2	3	24	BOWTIE	17:41500981:-	19:12773253:-	ENSG00000171401
	KRT13	PDIA3	exon-exon	0	2	4	25	BOWTIE	17:41500982:-	15:43746392:+	ENSG00000171401
	KRT13	PDIA3	exon-exon	0	2	3	24	BOWTIE	17:41500981:-	15:43746392:+	ENSG00000171401
	PITX1	ANXA1	cancer,exon-exon	0	2	4	22	BOWTIE	5:135028553:-	9:73157654:+	ENSG00000069011
	SH3PXD2A	ANXA1	cancer,exon-exon	0	2	2	18	BOWTIE	10:103673438:-	9:73158522:+	ENSG00000107957
	WSB1	ANXA2	exon-exon	0	2	2	25	BOWTIE	17:27297308:+	15:60393440:-	ENSG00000109046
	ZNF431	ANXA1	cancer,exon-exon	0	2	3	20	BOWTIE	19:21151198:+	9:73158522:+	ENSG00000196705
	KANSL1	LRRC37A3	banned,known,hpa,non_cancer_tissues,m0,multi,exon-exon	0	1	2	26	BOWTIE	17:46152904:-	17:64869166:-	ENSG00000120071
	KRT13	ANXA1	tcga,gtex,non_cancer_tissues,cancer,m34,exon-exon	0	35	4	23	BOWTIE	17:41500981:-	9:73157654:+	ENSG00000171401
	KRT13	ANXA1	tcga,gtex,non_cancer_tissues,cancer,m34,exon-exon	0	35	3	19	BOWTIE	17:41500982:-	9:73157654:+	ENSG00000171401
	KRT4	AHNAK	gtex	0	12	2	22	BOWTIE+STAR	12:52806552:-	11:62454948:-	ENSG00000170477
ID25_HGD	NPEPPS	TBC1D3	banned,known,oncogene,bodymap2,hpa,gtex,18cancers,tumor,m0,multi,exon-exon	0	11	10	29	BOWTIE	17:47592545:+	17:38191030:-	ENSG00000141279
	ANXA2	S100A9	cancer,exon-exon	0	3	2	23	BOWTIE	15:60394574:-	1:153360644:+	ENSG00000182718
	ANXA2	KRT78	exon-exon	0	2	13	30	BOWTIE	15:60394574:-	12:52839984:-	ENSG00000182718
	CPNE3	S100A9	cancer,exon-exon	0	2	4	27	BOWTIE	8:86524997:+	1:153360644:+	ENSG00000085719
	KRT13	GTF2I	exon-exon	0	2	4	23	BOWTIE	17:41500982:-	7:74657667:+	ENSG00000171401
	KRT13	GTF2I	exon-exon	0	2	2	18	BOWTIE	17:41500981:-	7:74657667:+	ENSG00000171401
	LSM14A	S100A9	cancer,exon-exon	0	2	4	27	BOWTIE	19:34195320:+	1:153360644:+	ENSG00000257103
	SPRR3	ANXA1	gtex,cancer,exon-exon	0	15	2	19	BOWTIE	1:153003855:+	9:73157654:+	ENSG00000163209
	RMND5A	ANAPC1	banned,known,bodymap2,hpa,m0,multi,exon-exon	0	10	8	29	BOWTIE	2:86741069:+	2:111822600:-	ENSG00000153561
	KRT13	LMO7	gtex,cancer	0	4	2	21	BOWTIE+STAR	17:41500995:-	13:75801231:+	ENSG00000171401
	NPEPPS	TBC1D3	banned,known,oncogene,bodymap2,hpa,gtex,18cancers,tumor,m0,multi,exon-exon	0	4	8	29	BOWTIE	17:47592545:+	17:38191030:-	ENSG00000141279
	KRT13	CRYBG1		0	3	3	21	BOWTIE+STAR	17:41500989:-	6:106472903:+	ENSG00000171401
	SMG1	NPIPB5	banned,known,bodymap2,hpa,m0,multi	0	3	2	27	BOWTIE+STAR	16:18858211:-	16:22513522:+	ENSG00000157106
	SMG1	NPIPB5	banned,known,bodymap2,hpa,m0,multi,exon-exon	0	3	2	19	BOWTIE	16:18858170:-	16:22513522:+	ENSG00000157106
	ANXA2	ERO1A	exon-exon	0	2	6	29	BOWTIE	15:60394574:-	14:52643630:-	ENSG00000182718
ID26_HGD	CNOT1	ANXA1	cancer,exon-exon	0	2	2	18	BOWTIE	16:58624673:-	9:73158522:+	ENSG00000125107
	CRABP2	ANXA1	cancer,exon-exon	0	2	4	20	BOWTIE	1:156699618:-	9:73157654:+	ENSG00000143320
	ERO1A	ANXA2	exon-exon	0	2	6	24	BOWTIE	14:52663801:-	15:60394695:-	ENSG00000197930
	KRT13	AC026461.1	no_protein,exon-exon	0	2	2	27	BOWTIE	17:41500982:-	16:56687578:+	ENSG00000171401
	KRT13	PDIA3	exon-exon	0	2	2	27	BOWTIE	17:41500982:-	15:43746392:+	ENSG00000171401
	NAIP	OCLN	banned,known,bodymap2,hpa,1000genomes,m0,multi,exon-exon	0	2	2	30	BOWTIE	5:70979869:-	5:69534694:+	ENSG00000249437

	PPIP5K1	CATSPER2	banned,known,bodymap2,hpa,1000genomes,m0,multi,10K<gap<100K,exon-exon	8	1	2	29	BOWTIE	15:43564867:-	15:43640496:-	ENSG00000168781
	PRKCH	FLJ22447	banned,known,no_protein,adjacent,lincrna,non_tumor_cells,hpa,18cancers,chimerdb3seq,10K<gap<100K,readthrough,exon-exon	0	1	2	20	BOWTIE	14:61530595:+	14:61650962:+	ENSG00000027075
	AC087386.1	IGK@	no_protein,lincrna,m14	0	16	2	31	BOWTIE+STAR	15:20137124:-	2:90256870:+	ENSG00000258410
	IGK@	AC087386.1	no_protein,lincrna,m14	0	16	3	38	BOWTIE+STAR	2:90255789:+	15:20138256:-	ENSG09000001012
	IGK@	AC087386.1	no_protein,lincrna,m14	0	16	3	29	BOWTIE+STAR	2:90257295:+	15:20136698:-	ENSG09000001012
	ATXN1L	AL136982.5	no_protein,pseudogene,m4	18	12	10	37	BOWTIE+STAR	16:71849747:+	10:87027189:+	ENSG00000224470
	NPEPPS	TBC1D3	banned,known,oncogene,bodymap2,hpa,gtex,18cancers,tumor,m0,multi,exon-exon	0	7	10	30	BOWTIE	17:47592545:+	17:38191030:-	ENSG00000141279
	RMND5A	ANAPC1	banned,known,bodymap2,hpa,m0,multi,exon-exon	0	6	11	30	BOWTIE;BOWTIE+STAR	2:86741069:+	2:111822600:-	ENSG00000153561
	ACER3	TMPRSS11B	m2	0	5	3	36	BOWTIE+STAR	11:77009811:+	4:68227424:-	ENSG00000078124
	ACER3	TMPRSS11B	m2	0	5	3	35	BOWTIE+STAR	11:77024274:+	4:68227424:-	ENSG00000078124
	S100A8	ANXA1	oncogene,gtex,non_cancer_tissues,cancer,exon-exon	0	5	2	19	BOWTIE	1:153390032:-	9:73157654:+	ENSG00000143546
	AC009094.1	NOP58	no_protein,pseudogene	16	4	2	36	BOWTIE+STAR	16:60024605:-	2:202303410:+	ENSG00000261278
	AC006210.2	RDX	no_protein,pseudogene,m2	37	3	2	33	BOWTIE+STAR	X:27517981:+	11:110264810:-	ENSG00000238247
	AC006210.2	RDX	no_protein,pseudogene,m2	37	3	2	30	BOWTIE+STAR	X:27518911:+	11:110255327:-	ENSG00000238247
	DSG1	CRNN		0	3	2	21	BOWTIE+STAR	18:31357079:+	1:152412246:-	ENSG00000134760
ID39_HGD	PPIP5K1	CATSPER2	banned,known,bodymap2,hpa,1000genomes,m0,multi,10K<gap<100K,exon-exon	12	3	2	28	BOWTIE	15:43568463:-	15:43649879:-	ENSG00000168781
	SLC22A20	HORMAD1	banned,known,no_protein,pseudogene,hpa,m0,multi,exon-exon	0	3	5	28	BOWTIE	11:65239480:+	1:150719538:-	ENSG00000197847
	SMG1	NPIPB5	banned,known,bodymap2,hpa,m0,multi,exon-exon	0	3	2	29	BOWTIE	16:18858170:-	16:22513522:+	ENSG00000157106
	ANKRD20A5P	CR381670.1	no_protein,pseudogene,exon-exon	21	2	8	29	BOWTIE	18:14201201:+	21:8864001:+	ENSG00000186481
	KRT13	DTX2	cancer,exon-exon	0	2	2	26	BOWTIE	17:41500982:-	7:76470300:+	ENSG00000171401
	KRT13	DTX2	cancer,exon-exon	0	2	2	25	BOWTIE	17:41500981:-	7:76470300:+	ENSG00000171401
	S100A9	TAB2	cancer,exon-exon	0	2	3	24	BOWTIE	1:153358433:+	6:149346478:+	ENSG00000163220
	SH3PXD2A	ANXA1	cancer,exon-exon	0	2	2	18	BOWTIE	10:103673438:-	9:73158522:+	ENSG00000107957
	HACL1	COLQ	banned,known,adjacent,healthy,cacg,hpa,1000genomes,chimerdb3seq,10K<gap<100K,readthrough,exon-exon	0	1	2	28	BOWTIE	3:15563358:-	3:15489637:-	ENSG00000131373
	NUTM2A-AS1	MINPP1	banned,known,no_protein,antisense,hpa,non_cancer_tissues,m0,multi,exon-exon	0	1	4	26	BOWTIE	10:87246115:-	10:87508336:+	ENSG00000223482
	KRT13	ANXA1	tcga,gtex,non_cancer_tissues,cancer,m33,exon-exon	0	35	3	22	BOWTIE	17:41500982:-	9:73157654:+	ENSG00000171401
	KRT13	ANXA1	tcga,gtex,non_cancer_tissues,cancer,m33,exon-exon	0	35	2	21	BOWTIE	17:41500981:-	9:73157654:+	ENSG00000171401
	SPRR3	ANXA1	gtex,cancer,exon-exon	0	15	3	21	BOWTIE	1:153003855:+	9:73157654:+	ENSG00000163209
	SPRR3	ANXA1	gtex,cancer,exon-exon	0	15	3	20	BOWTIE	1:153003856:+	9:73157654:+	ENSG00000163209
	NPEPPS	TBC1D3	banned,known,oncogene,bodymap2,hpa,gtex,18cancers,tumor,m0,multi,exon-exon	0	11	6	28	BOWTIE	17:47592545:+	17:38191030:-	ENSG00000141279
	KRT6A	SPRR1B	gtex,m3,oesophagus	0	7	3	28	BOWTIE+STAR	12:52487128:-	1:153032327:+	ENSG00000205420
	KRT6C	PPL	m2	0	7	2	21	BOWTIE+STAR	12:52468518:-	16:4950247:-	ENSG00000170465
	PRIM1	NACA	banned,known,adjacent,conjoing,healthy,bodymap2,hpa,1000genomes,cancer,gap<1K,readthrough	0	5	3	37	BOWTIE+STAR	12:56731608:-	12:56731607:-	ENSG00000198056
	AC024597.1	ZNF365	no_protein,lincrna,10K<gap<100K,exon-exon	0	4	4	26	BOWTIE	10:62289647:+	10:62376181:+	ENSG00000234756
	KRT13	HEBP2	exon-exon	0	4	3	22	BOWTIE	17:41500982:-	6:138403531:+	ENSG00000171401
	KRT13	HEBP2	exon-exon	0	4	2	21	BOWTIE	17:41500981:-	6:138403531:+	ENSG00000171401
	SPINK5	SPRR3	gtex	0	4	4	27	BOWTIE+STAR	5:148137336:+	1:153003002:+	ENSG00000133710
ID1_NDB	KANSL1	ARL17A	banned,known,healthy,bodymap2,hpa,gtex,18cancers,chimerdb3seq,m0,multi,exon-exon	0	3	3	28	BOWTIE	17:46094560:-	17:46570869:-	ENSG00000120071

	KANSL1	ARL17B	banned,known,healthy,bodymap2,hpa,chimerdb3seq,m0,multi,10K<gap<100K,exon-exon	0	3	3	28	BOWTIE	17:46094560:-	17:46352930:-	ENSG00000120071
	RMND5A	ANAPC1	banned,known,bodymap2,hpa,m0,multi,exon-exon	0	3	2	29	BOWTIE	2:86741069:+	2:111822600:-	ENSG00000153561
	S100A2	ANXA1	cancer,exon-exon	0	3	2	22	BOWTIE	1:153561343:-	9:73157654:+	ENSG00000196754
	ANXA2	S100A9	cancer,exon-exon	0	2	2	21	BOWTIE	15:60394574:-	1:153360644:+	ENSG00000182718
	KRT13	GTF2I	exon-exon	0	2	2	21	BOWTIE	17:41500981:-	7:74657667:+	ENSG00000171401
	KRT13	GTF2I	exon-exon	0	2	2	18	BOWTIE	17:41500982:-	7:74657667:+	ENSG00000171401
	LPIN1	ANXA1	cancer,exon-exon	0	2	2	18	BOWTIE	2:11764289:+	9:73158522:+	ENSG00000134324
	PITX1	ANXA1	cancer,exon-exon	0	2	5	22	BOWTIE	5:135028553:-	9:73157654:+	ENSG00000069011
	KANSL1	LRRC37A3	banned,known,hpa,non_cancer_tissues,m0,multi,exon-exon	0	1	2	20	BOWTIE	17:46152904:-	17:64869166:-	ENSG00000120071
	NSF	LRRC37A3	banned,known,hpa,m0,multi,exon-exon	0	1	3	23	BOWTIE	17:46704854:+	17:64898459:-	ENSG00000073969
	SMG1	NPIPB5	banned,known,bodymap2,hpa,m0,multi,exon-exon	0	1	4	26	BOWTIE	16:18858170:-	16:22513522:+	ENSG00000157106
**ID18_NDB**	AC087386.1	IGK@	no_protein,lincrna,m14	0	15	6	35	BOWTIE+STAR	15:20139242:-	2:90254788:+	ENSG00000258410
	IGK@	AC087386.1	no_protein,lincrna,m14	0	15	2	34	BOWTIE+STAR	2:90258285:+	15:20135655:-	ENSG09000001012
	NPEPPS	TBC1D3	banned,known,oncogene,bodymap2,hpa,gtex,18cancers,tumor,m0,multi,exon-exon	0	10	11	30	BOWTIE	17:47592545:+	17:38191030:-	ENSG00000141279
	AGAP13P	AGAP4	no_protein,pseudogene,m2,exon-exon	24	6	3	29	BOWTIE	10:46819536:+	10:45841687:-	ENSG00000243289
	AGAP13P	AGAP4	no_protein,pseudogene,m2,exon-exon	24	6	3	22	BOWTIE	10:46832476:+	10:45828080:-	ENSG00000243289
	AGAP13P	AGAP4	no_protein,pseudogene,m2,exon-exon	24	6	2	22	BOWTIE	10:46822212:+	10:45831429:-	ENSG00000243289
	AGAP4	AGAP13P	no_protein,pseudogene,m2,exon-exon	24	6	4	29	BOWTIE	10:45846687:-	10:46822178:+	ENSG00000188234
	AP005212.3	AL445670.1	no_protein,pseudogene	2	4	6	36	BOWTIE+STAR	18:14253624:+	9:97152578:-	ENSG00000274214
	SPINK5	SPRR3	gtex	0	4	2	27	BOWTIE+STAR	5:148137336:+	1:153003002:+	ENSG00000133710
	SCIN	AC024592.2	no_protein,antisense,m2	4	3	3	27	BOWTIE+STAR	7:12660106:+	19:5847512:+	ENSG00000006747
	PPIP5K1	CATSPER2	banned,known,bodymap2,hpa,1000genomes,10K<gap<100K,exon-exon	6	2	2	26	BOWTIE	15:43564867:-	15:43640496:-	ENSG00000168781
	S100A8	ANXA1	oncogene,gtex,non_cancer_tissues,cancer,exon-exon	0	2	2	19	BOWTIE	1:153390032:-	9:73157654:+	ENSG00000143546
**ID19_NDB**	KRT13	ANXA1	tcga,gtex,non_cancer_tissues,cancer,m38,exon-exon	0	39	3	22	BOWTIE	17:41500982:-	9:73157654:+	ENSG00000171401
	KRT13	ANXA1	tcga,gtex,non_cancer_tissues,cancer,m38,exon-exon	0	39	3	21	BOWTIE	17:41500981:-	9:73157654:+	ENSG00000171401
	SPRR3	ANXA1	gtex,cancer,exon-exon	0	25	2	22	BOWTIE	1:153003855:+	9:73157654:+	ENSG00000163209
	SPRR3	ANXA1	gtex,cancer,exon-exon	0	25	2	21	BOWTIE	1:153003856:+	9:73157654:+	ENSG00000163209
	AC087386.1	IGK@	no_protein,lincrna,m8	0	12	2	20	BOWTIE+STAR	15:20137975:-	2:90257337:+	ENSG00000258410
	IGK@	AC087386.1	no_protein,lincrna,m8	0	12	4	37	BOWTIE+STAR	2:90256432:+	15:20137594:-	ENSG09000001012
	NPEPPS	TBC1D3	banned,known,oncogene,bodymap2,hpa,gtex,18cancers,tumor,m0,multi,exon-exon	0	12	9	30	BOWTIE;BOWTIE+STAR	17:47592545:+	17:38191030:-	ENSG00000141279
	RMND5A	ANAPC1	banned,known,bodymap2,hpa,m0,multi,exon-exon	0	10	8	30	BOWTIE	2:86741069:+	2:111822600:-	ENSG00000153561
	ANXA2	S100A9	cancer,exon-exon	0	7	2	22	BOWTIE	15:60394574:-	1:153360644:+	ENSG00000182718
	IL1RN	ANXA1	cancer	0	4	2	20	BOWTIE+STAR	2:113133361:+	9:73165527:+	ENSG00000136689
	SMG1	NPIPB5	banned,known,bodymap2,hpa,m0,multi,exon-exon	0	4	3	28	BOWTIE	16:18858170:-	16:22513522:+	ENSG00000157106
	SMG1	NPIPB5	banned,known,bodymap2,hpa,m0,multi	0	4	2	38	BOWTIE+STAR	16:18858211:-	16:22513522:+	ENSG00000157106
	CAP1	S100A9	cancer	0	3	2	33	BOWTIE+STAR	1:40072677:+	1:153358269:+	ENSG00000131236
	KRT13	GTF2I	exon-exon	0	3	2	22	BOWTIE	17:41500982:-	7:74657667:+	ENSG00000171401
	ARPC2	ANXA1	cancer,exon-exon	0	2	6	26	BOWTIE	2:218254110:+	9:73157654:+	ENSG00000163466
	ARPC2	ANXA1	cancer,exon-exon	0	2	6	24	BOWTIE	2:218254108:+	9:73157654:+	ENSG00000163466
	ARPC2	ANXA1	cancer,exon-exon	0	2	2	21	BOWTIE	2:218254123:+	9:73157654:+	ENSG00000163466
	SPRR3	DDX24	exon-exon	0	2	2	22	BOWTIE	1:153003855:+	14:94053378:-	ENSG00000163209
	SPRR3	DDX24	exon-exon	0	2	2	21	BOWTIE	1:153003856:+	14:94053378:-	ENSG00000163209
	KANSL1	ARL17A	banned,known,healthy,bodymap2,hpa,gtex,18cancers,chimerdb3seq,m0,multi,exon-exon	0	1	4	28	BOWTIE	17:46094560:-	17:46570869:-	ENSG00000120071

	KANSL1	ARL17B	banned,known,healthy,bodymap2,hpa,chimerdb3seq,m0,multi,10K<gap<100K,exon-exon	0	1	4	28	BOWTIE	17:46094560:-	17:46352930:-	ENSG00000120071
	AC087386.1	IGK@	no_protein,lincrna,m21	0	22	2	32	BOWTIE+STAR	15:20139242:-	2:90254788:+	ENSG00000258410
	IGK@	AC087386.1	no_protein,lincrna,m21	0	22	3	38	BOWTIE+STAR	2:90255789:+	15:20138256:-	ENSG09000001012
	IGK@	AC087386.1	no_protein,lincrna,m21	0	22	3	30	BOWTIE+STAR	2:90258524:+	15:20135407:-	ENSG09000001012
	NPEPPS	TBC1D3	banned,known,oncogene,bodymap2,hpa,gtex,18cancers,tumor,m0,multi,exon-exon	0	14	14	30	BOWTIE	17:47592545:+	17:38191030:-	ENSG00000141279
	RMND5A	ANAPC1	banned,known,bodymap2,hpa,m0,multi,exon-exon	0	7	4	27	BOWTIE	2:86741069:+	2:111822600:-	ENSG00000153561
	ATXN1L	AL136982.5	no_protein,pseudogene,m2	5	5	7	35	BOWTIE+STAR	16:71849747:+	10:87027189:+	ENSG00000224470
	SPRR3	ANXA1	gtex,cancer,exon-exon	0	5	2	22	BOWTIE	1:153003856:+	9:73157654:+	ENSG00000163209
	SPRR3	ANXA1	gtex,cancer,exon-exon	0	5	2	21	BOWTIE	1:153003855:+	9:73157654:+	ENSG00000163209
	ACER3	TMPRSS11B	m2	0	4	4	37	BOWTIE+STAR	11:77009811:+	4:68227424:-	ENSG00000078124
	ACER3	TMPRSS11B	m2	0	4	2	22	BOWTIE+STAR	11:77024273:+	4:68227424:-	ENSG00000078124
	DSC3	AC245884.4	pseudogene,m3	4	4	5	37	BOWTIE+STAR	18:30992180:-	19:54386454:+	ENSG00000134762
	TMPRSS11B	ACER3	m2	0	4	6	32	BOWTIE+STAR	4:68227591:-	11:77012257:+	ENSG00000185873
ID22_NDB	AC145350.2	AC137761.1	no_protein,lincrna,m2	24	3	2	25	BOWTIE+STAR	16:33046591:-	16:32824586:+	ENSG00000260141
	ANXA1	EMP1	cancer	0	3	2	38	BOWTIE+STAR	9:73168880:+	12:13206927:+	ENSG00000135046
	DUS4L	BCAP29	banned,known,adjacent,healthy,bodymap2,hpa,chimerdb3pub,1K<gap<10K,readthrough,exon-exon	0	2	2	30	BOWTIE	7:107576592:+	7:107580759:+	ENSG00000105865
	KANSL1	ARL17A	banned,known,healthy,bodymap2,hpa,gtex,18cancers,chimerdb3seq,m0,multi,exon-exon	0	2	5	30	BOWTIE	17:46094560:-	17:46570869:-	ENSG00000120071
	KANSL1	ARL17B	banned,known,healthy,bodymap2,hpa,chimerdb3seq,m0,multi,10K<gap<100K,exon-exon	0	2	5	30	BOWTIE	17:46094560:-	17:46352930:-	ENSG00000120071
	SLC44A3-AS1	IL20RB	no_protein,pseudogene,exon-exon	0	2	3	23	BOWTIE	1:94638437:-	3:136980466:+	ENSG00000224081
	RRM2	C2ORF48	banned,known,no_protein,adjacent,lincrna,conjoing,cacg,non_tumor_cells,hpa,1000genomes,1K<gap<10K,readthrough,exon-exon	0	1	3	27	BOWTIE	2:10129154:+	2:10141854:+	ENSG00000171848
	KRT13	ANXA1	tcga,gtex,non_cancer_tissues,cancer,m29,exon-exon	0	30	2	20	BOWTIE	17:41500982:-	9:73157654:+	ENSG00000171401
	KRT13	ANXA1	tcga,gtex,non_cancer_tissues,cancer,m29,exon-exon	0	30	2	19	BOWTIE	17:41500981:-	9:73157654:+	ENSG00000171401
	NPEPPS	TBC1D3	banned,known,oncogene,bodymap2,hpa,gtex,18cancers,tumor,m0,multi,exon-exon	0	18	13	30	BOWTIE	17:47592545:+	17:38191030:-	ENSG00000141279
	S100A8	ANXA1	oncogene,gtex,non_cancer_tissues,cancer,exon-exon	0	13	2	19	BOWTIE	1:153390032:-	9:73157654:+	ENSG00000143546
	SPRR3	ANXA1	gtex,cancer,exon-exon	0	13	3	21	BOWTIE	1:153003855:+	9:73157654:+	ENSG00000163209
	SPRR3	ANXA1	gtex,cancer,exon-exon	0	13	3	20	BOWTIE	1:153003856:+	9:73157654:+	ENSG00000163209
	AC087386.1	IGK@	no_protein,lincrna,m8	0	9	2	33	BOWTIE+STAR	15:20137504:-	2:90256525:+	ENSG00000258410
	IGK@	AC087386.1	no_protein,lincrna,m8	0	9	2	37	BOWTIE+STAR	2:90256969:+	15:20137080:-	ENSG09000001012
	SMG1	NPIPB5	banned,known,bodymap2,hpa,m0,multi,exon-exon	0	7	4	30	BOWTIE	16:18858170:-	16:22513522:+	ENSG00000157106
	SCNN1A	TNFRSF1A	banned,known,adjacent,conjoing,healthy,bodymap2,non_tumor_cells,hpa,cancer,1K<gap<10K,readthrough,exon-exon	0	5	3	29	BOWTIE	12:6348727:-	12:6334244:-	ENSG00000111319
	NAIP	OCLN	banned,known,bodymap2,hpa,1000genomes,m0,multi,exon-exon	0	4	5	25	BOWTIE	5:70979869:-	5:69534694:+	ENSG00000249437
ID33_NDB	RMND5A	ANAPC1	banned,known,bodymap2,hpa,m0,multi,exon-exon	0	4	6	30	BOWTIE	2:86741069:+	2:111822600:-	ENSG00000153561
	ABBA01031661.1	ABBA01031663.1	no_protein,pseudogene,m2,10K<gap<100K	3	3	3	37	BOWTIE+STAR	20:29410456:-	20:29448626:-	ENSG00000282935
	DSC3	VSNL1	m2	21	3	5	35	BOWTIE+STAR	18:30992179:-	2:17579125:+	ENSG00000134762
	KRT6A	SPRR1B	gtex,m2,oesophagus	0	3	3	27	BOWTIE+STAR	12:52487128:-	1:153032327:+	ENSG00000205420
	RHCG	ACTB	cancer,m2	0	3	3	27	BOWTIE+STAR	15:89479109:-	7:5527639:-	ENSG00000140519
	SOD2	ACTB	18cancers,cancer	0	3	6	36	BOWTIE+BOWTIE2	6:159712800:-	7:5527622:-	ENSG00000112096
	SOD2	ACTB	18cancers,cancer	0	3	3	33	BOWTIE+STAR	6:159713954:-	7:5527639:-	ENSG00000112096
	SOD2	ACTB	18cancers,cancer	0	3	2	28	BOWTIE+STAR	6:159702851:-	7:5527645:-	ENSG00000112096

	SPSB1	H6PD	banned,known,adjacent,healthy,bodymap2,hpa,chimerdb3seq,10K<gap<100K,exon-exon	0	3	2	28	BOWTIE	1:9293071:+	1:9262059:+	ENSG00000171621
	CAP1	ANXA1	cancer,exon-exon	0	2	2	21	BOWTIE	1:40072271:+	9:73157654:+	ENSG00000131236
	KRT13	CD63	exon-exon	0	2	2	20	BOWTIE	17:41500982:-	12:55726356:-	ENSG00000171401
	KRT13	CD63	exon-exon	0	2	2	19	BOWTIE	17:41500981:-	12:55726356:-	ENSG00000171401
	KRT13	PDIA3	exon-exon	0	2	2	20	BOWTIE	17:41500982:-	15:43746392:+	ENSG00000171401
	KRT13	PDIA3	exon-exon	0	2	2	19	BOWTIE	17:41500981:-	15:43746392:+	ENSG00000171401
	OGT	ANXA1	cancer,exon-exon	0	2	4	20	BOWTIE	X:71537110:+	9:73158522:+	ENSG00000147162
	TMBIM1	ANXA1	18cancers,cancer,exon-exon	0	2	4	20	BOWTIE	2:218284039:-	9:73158522:+	ENSG00000135926
	TRADD	B3GNT9	adjacent,18cancers,1K<gap<10K,readthrough,exon-exon	0	2	2	30	BOWTIE	16:67159838:-	16:67150671:-	ENSG00000102871
	WSB1	ANXA1	cancer,exon-exon	0	2	2	18	BOWTIE	17:27297308:+	9:73158522:+	ENSG00000109046
	NSF	LRRC37A3	banned,known,hpa,m0,multi,exon-exon	0	1	3	30	BOWTIE	17:46704854:+	17:64898459:-	ENSG00000073969
ID35_NDB	KRT13	ANXA1	tcga,gtex,non_cancer_tissues,cancer,m45,exon-exon	0	46	3	19	BOWTIE	17:41500982:-	9:73157654:+	ENSG00000171401
	KRT13	ANXA1	tcga,gtex,non_cancer_tissues,cancer,m45,exon-exon	0	46	2	18	BOWTIE	17:41500981:-	9:73157654:+	ENSG00000171401
	SPRR3	ANXA1	gtex,cancer,exon-exon	0	21	2	19	BOWTIE	1:153003855:+	9:73157654:+	ENSG00000163209
	ACER3	TMPRSS11B	m3	0	7	3	35	BOWTIE+STAR	11:77009811:+	4:68227424:-	ENSG00000078124
	ACER3	TMPRSS11B	m3	0	7	3	29	BOWTIE+STAR	11:77024273:+	4:68227424:-	ENSG00000078124
	NPEPPS	TBC1D3	banned,known,oncogene,bodymap2,hpa,gtex,18cancers,tumor,m0,multi,exon-exon	0	6	6	29	BOWTIE	17:47592545:+	17:38191030:-	ENSG00000141279
	AC004951.3	RASA4	no_protein,pseudogene,m4,exon-exon	61	5	3	22	BOWTIE	7:43965867:-	7:102595784:-	ENSG00000241057
	KRT4	LMO7	cancer	0	4	2	18	BOWTIE+STAR	12:52813800:-	13:75636519:+	ENSG00000170477
	RMND5A	ANAPC1	banned,known,bodymap2,hpa,m0,multi,exon-exon	0	4	7	29	BOWTIE	2:86741069:+	2:111822600:-	ENSG00000153561
	SMG1	NPIPB5	banned,known,bodymap2,hpa,m0,multi	0	4	3	38	BOWTIE+STAR	16:18858211:-	16:22513522:+	ENSG00000157106
	SMG1	NPIPB5	banned,known,bodymap2,hpa,m0,multi	0	4	2	37	BOWTIE+BOWTIE2	16:18858209:-	16:22513426:+	ENSG00000157106
	SMG1	NPIPB5	banned,known,bodymap2,hpa,m0,multi,exon-exon	0	4	2	27	BOWTIE	16:18858170:-	16:22513522:+	ENSG00000157106
	KRT13	CD63	exon-exon	0	2	3	19	BOWTIE	17:41500982:-	12:55726356:-	ENSG00000171401
	KRT13	CD63	exon-exon	0	2	2	18	BOWTIE	17:41500981:-	12:55726356:-	ENSG00000171401
	KRT13	HEBP2	exon-exon	0	2	3	19	BOWTIE	17:41500982:-	6:138403531:+	ENSG00000171401
	KRT13	HEBP2	exon-exon	0	2	2	18	BOWTIE	17:41500981:-	6:138403531:+	ENSG00000171401
	KRT13	LINC00969	no_protein,lincrna,exon-exon	0	2	3	19	BOWTIE	17:41500982:-	3:195681603:+	ENSG00000171401
	KRT13	LINC00969	no_protein,lincrna,exon-exon	0	2	2	18	BOWTIE	17:41500981:-	3:195681603:+	ENSG00000171401
	PITX1	ANXA1	cancer,exon-exon	0	2	3	20	BOWTIE	5:135028553:-	9:73157654:+	ENSG00000069011
	KANSL1	ARL17A	banned,known,healthy,bodymap2,hpa,gtex,18cancers,chimerdb3seq,m0,multi,exon-exon	0	1	4	30	BOWTIE	17:46094560:-	17:46570869:-	ENSG00000120071
	KANSL1	ARL17B	banned,known,healthy,bodymap2,hpa,chimerdb3seq,m0,multi,10K<gap<100K,exon-exon	0	1	4	30	BOWTIE	17:46094560:-	17:46352930:-	ENSG00000120071
ID37_NDB	KRT13	ANXA1	tcga,gtex,non_cancer_tissues,cancer,exon-exon	0	12	2	22	BOWTIE	17:41500981:-	9:73157654:+	ENSG00000171401
	KRT13	ANXA1	tcga,gtex,non_cancer_tissues,cancer,exon-exon	0	12	2	21	BOWTIE	17:41500982:-	9:73157654:+	ENSG00000171401
	SPRR3	ANXA1	gtex,cancer,exon-exon	0	10	2	19	BOWTIE	1:153003855:+	9:73157654:+	ENSG00000163209
	SPRR3	ANXA1	gtex,cancer,exon-exon	0	10	2	18	BOWTIE	1:153003856:+	9:73157654:+	ENSG00000163209
	NPEPPS	TBC1D3	banned,known,oncogene,bodymap2,hpa,gtex,18cancers,tumor,m0,multi,exon-exon	0	9	11	30	BOWTIE	17:47592545:+	17:38191030:-	ENSG00000141279
	RMND5A	ANAPC1	banned,known,bodymap2,hpa,m0,multi,exon-exon	0	7	8	28	BOWTIE	2:86741069:+	2:111822600:-	ENSG00000153561
	AC087386.1	IGK@	no_protein,lincrna	0	5	2	34	BOWTIE+STAR	15:20139242:-	2:90254788:+	ENSG00000258410
	IGK@	AC087386.1	no_protein,lincrna	0	5	4	38	BOWTIE+STAR	2:90255789:+	15:20138256:-	ENSG09000001012
	IGK@	AC087386.1	no_protein,lincrna	0	5	2	34	BOWTIE+STAR	2:90260781:+	15:20133151:-	ENSG09000001012
	IGK@	AC087386.1	no_protein,lincrna	0	5	2	32	BOWTIE+STAR	2:90256208:+	15:20137834:-	ENSG09000001012
	IGK@	AC087386.1	no_protein,lincrna	0	5	2	28	BOWTIE+STAR	2:90257295:+	15:20136698:-	ENSG09000001012
	SPINK5	SPRR3	gtex	0	5	4	27	BOWTIE+STAR	5:148137336:+	1:153003002:+	ENSG00000133710
	TRIM56	TMPRSS11B		1	4	2	26	BOWTIE+STAR	7:101096190:+	4:68227424:-	ENSG00000169871

	CRLF2	GBP6	oncogene,cancer,m2	1	3	3	36	BOWTIE+STAR	X:1224767:-	1:89385616:+	ENSG00000205755
	IGK@	TMEM128		0	3	3	19	BOWTIE+STAR	2:90241463:-	4:4237935:-	ENSG09000000012
	NAIP	OCLN	banned,known,bodymap2,hpa,1000 genomes,m0,multi,exon-exon	0	2	2	29	BOWTIE	5:70983775:-	5:69534694:+	ENSG00000249437
	NAIP	OCLN	banned,known,bodymap2,hpa,1000 genomes,m0,multi,exon-exon	0	2	2	28	BOWTIE	5:70979869:-	5:69534694:+	ENSG00000249437
**ID40_NDB**	*excluded from fusion gene analysis (see 'Methods' section)*										

gene_2_id(3' end_fusion_partner)	exon_1_id(5' end_fusion_partner)	exon_2_id(3' end_fusion_partner)	fusion_sequence	predicted_effect
ENSG00000135046	ENSE00001841099	ENSE00001472097	AGAAAAGATTATTCAATAAAGTTTCCTGCCTTTCTGCAAACAT*AAAAAAAAAAAAAAAAAAAAAAAGCAATCATTAAGCTTTTCATC	UTR/UTR
ENSG00000135046	ENSE00003650505	ENSE00001472097	GAAAAGATTATTCAATAAAGTTTCCTGCCTTTCTGCAAACATA*AAAAAAAAAAAAAAAAAAAAAAAGCAATCATTAAGCTTTTCATC	UTR/UTR
ENSG00000135046	ENSE00001548454	ENSE00001472097	TGCATGTTTCCTGCTCTTCCCTCATTAAATTGCTTTTAATTCC*AAAAAAAAAAAAAAAAAAAAAAAGCAATCATTAAGCTTTTCATC	UTR/UTR
ENSG00000135046	ENSE00001901937	ENSE00001472097	GCATGTTTCCTGCTCTTCCCTCATTAAATTGCTTTTAATTCCA*AAAAAAAAAAAAAAAAAAAAAAAGCAATCATTAAGCTTTTCATC	UTR/UTR
ENSG00000153107	ENSE00002531730	ENSE00002441208	CATCCACAGCAGTGTTTCTCGGGTTGGAAAAGCCATTGATAAG*GATTCACTTTAAGAGATTTGGAAACTCTTCCCTTTGGAATTGC	out-of-frame
ENSG00000118898			TTCCCTCTTATCCATCTGCAGGTGAATCTTCAATAAAATGCTTTTGTCAT*TCAAAAAAAAAAATAAAAAAAAAAAGAGTGCCATCCAGCCGGTTTTGGTA	UTR/intronic
ENSG00000163220	ENSE00002555095	ENSE00001512631	AGTGATTCTCCAGCCTCACCCTCCCGAGTAGCTGGGATTACAG*AAGGAGAATAAGAATGAAAAGGTCATAGAACACATCATGGAGG	UTR/CDS(truncated)
ENSG00000067225			CTTCTCTACCCCAAAGAAAAGATTATTCAATAAAGTTTCCTGCCTTTC*CAAACATAAAAAAAAAAACAAAAAAATCGGTAGAGAGTGATTTCTCTCCC	UTR/intronic
ENSG00000137710			GGTTTGCGTGAGGTCTGGTTTTTTTGGGCTGCAGTATGTAGACAGCAAAG*GTTATTCTACATGGCTTAAACTAAATAAAAAGGTAAAATATGTACGATAA	exonic(no-known-CDS)/CDS(truncated)
ENSG00000274611	ENSE00003785912	ENSE00003731868	AACATGTATTTAACCAAGTTCCAACAAAAGAATGCTGCCACAG*GATGGACGTGGTAGAGGTCGCGGGCAGTTGGTGGGCACAAGAG	CDS(complete)/UTR
ENSG00000228335			CATTTGGTTTTAGGAAGAAGAAACAAAGCCCATTGAGCTCCCTGTCAAAG*AGGAAGAACCCCCTGAAAAAAACTGTTGATGTGGCAGCAGAGAAGAAAGT	CDS(truncated)/exonic(no-known-CDS)
ENSG00000075790	ENSE00003520906	ENSE00003577399	TTAAAGGAAGCAGAAAATGTGTGGCGGATTACTGGGACAGATG*GTGTGAAGAAAAAAATGACACTCCAATGGGCTGCAGTGGCAAC	CDS(truncated)/UTR
ENSG00000238083	ENSE00002689718	ENSE00002373231	GAAAGCAGCTTCAGGAGCCATAGTACCTACAGCAGCACTCCAG*AAATTTCCAAGGAAACTATATTTCTTACATTGATGGAAATGTA	out-of-frame
ENSG00000135046	ENSE00001841099	ENSE00001472097	AGAAAAGATTATTCAATAAAGTTTCCTGCCTTTCTGCAAACAT*AAAAAAAAAAAAAAAAAAAAAAAGCAATCATTAAGCTTTTCATC	UTR/UTR
ENSG00000135046	ENSE00003650505	ENSE00001472097	GAAAAGATTATTCAATAAAGTTTCCTGCCTTTCTGCAAACATA*AAAAAAAAAAAAAAAAAAAAAAAGCAATCATTAAGCTTTTCATC	UTR/UTR
ENSG00000274611	ENSE00003785912	ENSE00003731868	AACATGTATTTAACCAAGTTCCAACAAAAGAATGCTGCCACAG*GATGGACGTGGTAGAGGTCGCGGGCAGTTGGTGGGCACAAGAG	CDS(complete)/UTR
ENSG00000153107	ENSE00002531730	ENSE00002441208	CATCCACAGCAGTGTTTCTCGGGTTGGAAAAGCCATTGATAAG*GATTCACTTTAAGAGATTTGGAAACTCTTCCCTTTGGAATTGC	out-of-frame
ENSG00000124942			TCGTACCTCTGCACTCCAGCCTGGGTGGGTGACAGAGTGAGATCCTATCT*CAAAAAAAAAACCAAAAAAAAAAAAACCAAAAAAAGAAAAGAGAAGCCCA	UTR/intronic
ENSG00000196531	ENSE00003575614	ENSE00003607207	AATCTGGATAAATCCCGAAAAGGAGAACTTCTTAAGAAGAGTG*GGTCTGGAACAGAATCTGACAGTGATGAATCAGTACCAGAGCT	in-frame
ENSG00000067182			TCTTCAAGGAGCTGAACTACAAAACCAATTCTGAGTCTCCCTCTGTCACG*AATTCTCTGGACTGAGGCTCCAGTTCTGGCCTTTGGGGTTCAAGATCACT	CDS(truncated)/UTR
ENSG09000001012			CTGTGAGTTGAATGTTGTGTTCATAGTATCTGCCAAAACAGAAAGAAAAA*AAAACAAAATATTTTGATAAGAAGTTAAAGCTTTGTATATAATATACCTT	intronic/---
ENSG00000183347			CTCTGTCACCCAGGCTGGAGTGCAGTGGTGCAATCTCGGCTCGCTGCAAC*CTCTGCCTCCTGGGTTCAAGAGATTCACCTGCCTCAGCCCCCTAGTAGCT	intergenic/UTR
ENSG00000205755			GATGGGGTTTCACTATGTTGGCCAGGCTGGTCTCGAACTCTTGACCTCAA*GATCCACCCGCCTCGGCCTCCCAAAGTGCTGGGATGACAGGCGTGAGCCA	UTR/intergenic
ENSG00000205755			GATGGGGTTTCACTATGTTGGCCAGGCTGGTCTCGAACTCTTGACCTCAA*GATCCACCCGCCTCGGCCTCCCAAAGTGCTGGGATCACAGGTGTGACTCA	UTR/intergenic
ENSG00000205755			GATGGGGTTTCACTATGTTGGCCAGGCTGGTCTCGAACTCTTGACCTCAA*GATCCACCCGCCTCGGCCTCCCAAAGTGCTGGGATGACAGGCCTGAGCCA	UTR/intergenic
ENSG00000205755			GATGGGGTTTCACTATGTTGGCCAGGCTGGTCTCGAACTCTTGACCTCAA*GATCCACCCGCCTCGGCCTCCCAAAGTGCTGGGATGACAGGCGTGAGCCA	UTR/intergenic
ENSG00000132406			ATGGAATGAAGCTGGCCTCCCGTCTCCTTGATTTGAAGCAGCTTCTCCAC*CTTCAACATTGCTTTATGGCATGTGTGGTCGTTTTTCACTCCATTGTTGT	---/CDS(truncated)
ENSG00000258410			TGATAAGATGACTTGAGATGAGATGAAATGATGAGATGAAATGACAAAAT*GATGAGATGATGAGATGAAATTTTGAGATGAAATGGTGAGTAGAAATGAT	---/intronic
ENSG00000197822	ENSE00003590701	ENSE00003560198	TTATTGATCCAAATTTCAGCTGAGTATGATCCTTCCAAACTAG*GTTAAAAATGTGTCTGCAGGCACACAGGACGTGCCTTCACCCC	out-of-frame
ENSG00000185829	ENSE00003662350	ENSE00003806456	TCGTCAGCAAACAGACATTTACAAACAGATACGTGCTAATAAG*GTTTCTGTGTGGAGACAGTAGAATATAAAAATAACACCTTCGC	out-of-frame
ENSG00000228696	ENSE00003662350	ENSE00002370389	TCGTCAGCAAACAGACATTTACAAACAGATACGTGCTAATAAG*GTTTCTGTGTGGAGACAGTAGAATATAAAAATAACACCTTCGC	out-of-frame
ENSG00000122705	ENSE00001841099	ENSE00001839548	AGAAAAGATTATTCAATAAAGTTTCCTGCCTTTCTGCAAACAT*AAAAAAAAAAAAAAAAAAAAAAAAAAAAAAAAAAAAAAAAAAA	UTR/UTR
ENSG00000122705	ENSE00003650505	ENSE00001839548	GAAAAGATTATTCAATAAAGTTTCCTGCCTTTCTGCAAACATA*AAAAAAAAAAAAAAAAAAAAAAAAAAAAAAAAAAAAAAAAAAA	UTR/UTR
ENSG00000135046	ENSE00001889023	ENSE00003569681	ATTGATCCTCCTGCCTCAGCCTTCTGAGTAGCTGGGACTACAG*ACACTTTTTCAAAAATGGCAATGGTATCAGAATTCCTCAAGCA	UTR/CDS(no-known-start-or-end)
ENSG00000238083	ENSE00002689718	ENSE00002373231	GAAAGCAGCTTCAGGAGCCATAGTACCTACAGCAGCACTCCAG*AAATTTCCAAGGAAACTATATTTCTTACATTGATGGAAATGTA	out-of-frame

ENSG00000135046	ENSE00001841099	ENSE00001472097	AGAAAAGATTATTCAATAAAGTTTCCTGCCTTTCTGCAAACAT*AAAAAAAAAAAAAAAAAAAAAAAGCAATCATTAAGCTTTTCATC	UTR/UTR
ENSG00000135046	ENSE00001548454	ENSE00001472097	TGCATGTTTCCTGCTCTTCCCTCATTAAATTGCTTTTAATTCC*AAAAAAAAAAAAAAAAAAAAAAAGCAATCATTAAGCTTTTCATC	UTR/UTR
ENSG00000135046	ENSE00001901937	ENSE00001472097	GCATGTTTCCTGCTCTTCCCTCATTAAATTGCTTTTAATTCCA*AAAAAAAAAAAAAAAAAAAAAAAGCAATCATTAAGCTTTTCATC	UTR/UTR
ENSG00000274611	ENSE00003785912	ENSE00003731868	AACATGTATTTAACCAAGTTCCAACAAAAGAATGCTGCCACAG*GATGGACGTGGTAGAGGTCGCGGGCAGTTGGTGGGCACAAGAG	CDS(complete)/UTR
ENSG00000153107	ENSE00002531730	ENSE00002441208	CATCCACAGCAGTGTTTCTCGGGTTGGAAAAGCCATTGATAAG*GATTCACTTTAAGAGATTTGGAAACTCTTCCCTTTGGAATTGC	out-of-frame
ENSG00000243716			TTACGTTCATGTAAACAGCATGACGTGAGGCCATGGATGCAGGCATTAAG*GTATGATCTCGTGAAATCTTGAGAGAAACTGAATGACGAATGAAACTATT	CDS(truncated)/UTR
ENSG00000243716	ENSE00001505919	ENSE00002138288	AGGTATACTATGTACCAGAATCAGTTGTTGGAGAAAATTAAAG*GTATGATCTCGTGAAATCTTGAGAGAAACTGAATGACGAATGA	CDS(truncated)/UTR
ENSG00000263001	ENSE00001841099	ENSE00003722138	AGAAAAGATTATTCAATAAAGTTTCCTGCCTTTCTGCAAACAT*AAAAAAAAAAAAGAAAAAAAAGAAAAAAAAAAAGGAGGAGGAGG	UTR/UTR
ENSG00000105245			CTCCAACAGCTGCCTTCTCCCGGGTTCCAGCCTCTCAGTGTGTTGGAGAG*GGCGGACCCCGGAGGCCTGAGCGGCACCTGCCCCCAGCCCCCTGTGGGGC	UTR/CDS(truncated)
ENSG00000167004	ENSE00001841099	ENSE00001489318	AGAAAAGATTATTCAATAAAGTTTCCTGCCTTTCTGCAAACAT*AAAAAAAAAAAAAAAAAAAAAAAAAAAAAAAAAAAAAAAAAAAA	UTR/UTR
ENSG00000094755			CAGGGGGAACAAAAGCATCTCCATGAGTGTGGCTGGGTCACGACAAGGTG*TCAGCTTGGGGGTGCTGAAGGCCCTCCTCCTAAATGCAGGGCAAAGAAAA	CDS(truncated)/intronic
ENSG00000124942			TGATGATTTCTGTGCTCTTCGCTGTTTGCAATTGCTAAATAAAGCAGATT*TAAAATACAAAAAAAAAAAAAAAAAAAAAAATTAGCCAGGCCTGGTGGCAG	UTR/intronic
ENSG00000135046	ENSE00002064325	ENSE00001472097	CCACTCTGCCCTCCAAAAAGACAAAAAAAAAAAAAAAAAAAAAA*AAAAAAAAAAAAAAAAAAAAAAAGCAATCATTAAGCTTTTCATC	UTR/UTR
ENSG00000156482	ENSE00001739980	ENSE00002110148	GAAGAATTTCTCTTTAGTTCTTTGCAAGAAGGTAGAGATAAAG*AGTCTTGCTGTGTCGCCCAGGCTGGAGTACAATGGCGCGATCT	UTR/UTR
ENSG00000233013	ENSE00003682552	ENSE00002337959	AAGTGCAATTCAAGATATCCGAAGGTTCAATTATGTGGTCAAA*ATGATGATTATTCCCCACCTTCTAAGAGACAAAGACCAACGAG	CDS(truncated)/exonic(no-known-CDS)
ENSG00000092148	ENSE00001841099	ENSE00002483533	AGAAAAGATTATTCAATAAAGTTTCCTGCCTTTCTGCAAACAT*GAAAAAAAGAAAAAAAAAAAAGAATCTTTGATAGAAAACAGACA	UTR/UTR
ENSG00000135046	ENSE00001935261	ENSE00001472097	GAAACGAGCTGTGCTTGCAAAGACTTCATAGTTCCCAAGAATT*AAAAAAAAAAAAAAAAAAAAAAAGCAATCATTAAGCTTTTCATC	UTR/UTR
ENSG00000135046	ENSE00001834474	ENSE00001472097	AACGAGCTGTGCTTGCAAAGACTTCATAGTTCCCAAGAATTAA*AAAAAAAAAAAAAAAAAAAAAAAGCAATCATTAAGCTTTTCATC	UTR/UTR
ENSG00000163220	ENSE00003785048	ENSE00001512631	AGCAATTCTCCTGCCTCAGCCTCCCGAGTAGCTGGGATTACAG*AAGGAGAATAAGAATGAAAAGGTCATAGAACACATCATGGAGG	exonic(no-known-CDS)/CDS(truncated)
ENSG00000228335			CATTTGGTTTTAGGAAGAAGAAACAAAGCCCATTGAGCTCCCTGTCAAAG*AGGAAGAACCCCCTGAAAAAAACTGTTGATGTGGCAGCAGAGAAGAAAGT	CDS(truncated)/exonic(no-known-CDS)
ENSG00000135046	ENSE00001616512	ENSE00003569681	AGTGATTCTCCTGCCTCAGCCTCCTGAGTAGCTGGGACTACAG*ACACTTTTTCAAAAATGGCAATGGTATCAGAATTCCTCAAGCA	UTR/CDS(no-known-start-or-end)
ENSG00000135046	ENSE00002064325	ENSE00001472097	CCACTCTGCCCTCCAAAAAGACAAAAAAAAAAAAAAAAAAAAAA*AAAAAAAAAAAAAAAAAAAAAAAGCAATCATTAAGCTTTTCATC	UTR/UTR
ENSG00000163141	ENSE00001548454	ENSE00001851689	TGCATGTTTCCTGCTCTTCCCTCATTAAATTGCTTTTAATTCC*AAAAAAAAAAAAAAAAAAAAAATCTTGAGAGTTTTTTTAAAAAT	UTR/UTR
ENSG00000163141	ENSE00001901937	ENSE00001851689	GCATGTTTCCTGCTCTTCCCTCATTAAATTGCTTTTAATTCCA*AAAAAAAAAAAAAAAAAAAAAATCTTGAGAGTTTTTTTAAAAAT	UTR/UTR
ENSG00000263001	ENSE00001901937	ENSE00003722138	GCATGTTTCCTGCTCTTCCCTCATTAAATTGCTTTTAATTCCA*AAAAAAAAAAAAGAAAAAAAAGAAAAAAAAAAAGGAGGAGGAGG	UTR/UTR
ENSG00000263001	ENSE00001548454	ENSE00003722138	TGCATGTTTCCTGCTCTTCCCTCATTAAATTGCTTTTAATTCC*AAAAAAAAAAAAGAAAAAAAAGAAAAAAAAAAAGGAGGAGGAGG	UTR/UTR
ENSG00000121716	ENSE00001548454	ENSE00003705742	TGCATGTTTCCTGCTCTTCCCTCATTAAATTGCTTTTAATTCC*TAAAAAAAAAAAAAAAAAAAAGGATTTGAAGTCCTGGCCGGAGC	UTR/UTR
ENSG00000272752	ENSE00001548454	ENSE00002319092	TGCATGTTTCCTGCTCTTCCCTCATTAAATTGCTTTTAATTCC*AAAAAAAAAAAAAAAAAAAAAAAAAAAAAAAAAAAAAAAAAAA	UTR/exonic(no-known-CDS)
ENSG00000272752	ENSE00001901937	ENSE00002319092	GCATGTTTCCTGCTCTTCCCTCATTAAATTGCTTTTAATTCCA*AAAAAAAAAAAAAAAAAAAAAAAAAAAAAAAAAAAAAAAAAAA	UTR/exonic(no-known-CDS)
ENSG00000125107	ENSE00003629864	ENSE00002583055	ATGGCATCCTGGAATGCAGTTCCTGCTTCCATTAAACTCATGG*ATGGAGTCTTGCTCTGTTGCCCAGGCTTGAGAGCAGTGGTGTG	CDS(truncated)/UTR
ENSG00000075790	ENSE00003520906	ENSE00003577399	TTAAAGGAAGCAGAAAATGTGTGGCGGATTACTGGGACAGATG*GTGTGAAGAAAAAAATGACACTCCAATGGGCTGCAGTGGCAAC	CDS(truncated)/UTR
ENSG00000185829	ENSE00003662350	ENSE00003806456	TCGTCAGCAAACAGACATTTACAAACAGATACGTGCTAATAAG*GTTTCTGTGTGGAGACAGTAGAATATAAAAATAACACCTTCGC	out-of-frame
ENSG00000228696	ENSE00003662350	ENSE00002370389	TCGTCAGCAAACAGACATTTACAAACAGATACGTGCTAATAAG*GTTTCTGTGTGGAGACAGTAGAATATAAAAATAACACCTTCGC	out-of-frame
ENSG00000143452	ENSE00002175592	ENSE00003622633	GAAGTGAGGAGCCTCTCCGCCCGGCAGCCACCCCATCTGGGAA*GTTGAATTAAAGAAAATACTTTATCAGAAGAAGATGGCCACTG	exonic(no-known-CDS)/UTR
ENSG00000135046	ENSE00001841099	ENSE00001472097	AGAAAAGATTATTCAATAAAGTTTCCTGCCTTTCTGCAAACAT*AAAAAAAAAAAAAAAAAAAAAAAGCAATCATTAAGCTTTTCATC	UTR/UTR
ENSG00000135046	ENSE00003650505	ENSE00001472097	GAAAAGATTATTCAATAAAGTTTCCTGCCTTTCTGCAAACATA*AAAAAAAAAAAAAAAAAAAAAAAGCAATCATTAAGCTTTTCATC	UTR/UTR
ENSG00000135046	ENSE00001548454	ENSE00001472097	TGCATGTTTCCTGCTCTTCCCTCATTAAATTGCTTTTAATTCC*AAAAAAAAAAAAAAAAAAAAAAAGCAATCATTAAGCTTTTCATC	UTR/UTR
ENSG00000135046	ENSE00001901937	ENSE00001472097	GCATGTTTCCTGCTCTTCCCTCATTAAATTGCTTTTAATTCCA*AAAAAAAAAAAAAAAAAAAAAAAGCAATCATTAAGCTTTTCATC	UTR/UTR
ENSG00000274611	ENSE00003785912	ENSE00003731868	AACATGTATTTAACCAAGTTCCAACAAAAGAATGCTGCCACAG*GATGGACGTGGTAGAGGTCGCGGGCAGTTGGTGGGCACAAGAG	CDS(complete)/UTR
ENSG09000001012			AAAAATGAGATGAAATGAAATGAAATAATGAAATGAAATAATGAAATGAG*ATGAAATGAAATAATGAAATGATGAAATAATGAAATGAAAATGAAATGGA	intronic/---
ENSG00000258410			TGATAAGATGACTTGAGATGAGATGAAATGATGAGATGAAATGACAAAAT*GATGAGATGATGAGATGAAATTTTGAGATGAAATGGTGAGTAGAAATGAT	---/intronic
ENSG00000258410			TAAATGAGATGATGAAATTAAATGATGAAATGAAATAATGAAATGGAAAT*GATGAGATGAAATGATGAGATGAATGATGAGATGAAATGAGATGAAATGA	---/intronic
ENSG00000243716			TTACGTTCATGTAAACAGCATGACGTGAGGCCATGGATGCAGGCATTAAG*GTATGATCTCGTGAAATCTTGAGAGAAACTGAATGACGAATGAAACTATT	CDS(truncated)/UTR

ENSG00000243716	ENSE00001505919	ENSE00002138288	AGGTATACTATGTACCAGAATCAGTTGTTGGAGAAAATTAAAG*GTATGATCTCGTGAAATCTTGAGAGAAACTGAATGACGAATGA	CDS(truncated)/UTR
ENSG00000255248			ATATGTGCCAAATTTTCTTAATCCAGTCTATCGTTGCTGGACATTTAGGT*NNNNNNNNNNNNNNNNNNNNNGTCAGTGTGGCGATTCCTCAGGGATCTAGAACTAGAAATACCATTTGACC	---/intergenic
ENSG00000153107	ENSE00002531730	ENSE00002441208	CATCCACAGCAGTGTTTCTCGGGTTGGAAAAGCCATTGATAAG*GATTCACTTTAAGAGATTTGGAAACTCTTCCCTTTGGAATTGC	out-of-frame
ENSG00000197930			TTCAAGCAATTCTCCCACCTCAGCCTCCCGAGTAGCTGGGATTACAGACA*TTCTACAAGTGTGAAAGAATTAGAAAACTTCAGGAACTTGTTACAGAATA	intronic/CDS(truncated)
ENSG00000143536			ACTCCTCACACCACTTAACAGCCACTTGTTTCATCCCACCTGGGCATTAG*GTTGACTTCAAAGATGCCTCAGTTACTGCAAAACATTAATGGGATCATCG	intergenic/UTR
ENSG00000197930			CTCAAGCAATTCTCCTGTCTCAGCCTCCCAAGTAGCTGGGATTACAGGTT*TTCTACAAGTGTGAAAGAATTAGAAAACTTCAGGAACTTGTTACAGAATA	intronic/CDS(truncated)
ENSG00000230825			ACAAAAGTGAAGTTAGGAAATCCTTGAAAGAAAATCATGAATGAACAAAG*ACAATGCATAGGAGTGAATGAGACAGAAAGCAAGACCATGAGGATGACTA	UTR/exonic(no-known-CDS)
ENSG00000237955			CCCTAAAATCTATGTGTTTTAGACTTAGACTTTTTATTGCCCCCCCCC*TTTTTTTTTTGAGACGGAGTCTCGCTCTGTCGCCCAGGCTGGAGTGCAG	UTR/intergenic
ENSG00000143452			CCCCGTCTGAGAAGTGAGGAGCCTCTCCGCCCGGCAGCCACCCCATCTGG*TTGAATTAAAGAAAATACTTTATCAGAAGAAGATGGCCACTGCCCAGTTG	exonic(no-known-CDS)/UTR
ENSG00000197930	ENSE00001775033	ENSE00003581639	AGTGATCCACCCACCATGGCCTCCCAAAGTGCTGGGATTACAG*GGACAAGTGAAGAGAACACTTTTTACAGTTGGCTAGAAGGTCT	UTR/CDS(truncated)
ENSG00000197930	ENSE00001775033	ENSE00003468165	AGTGATCCACCCACCATGGCCTCCCAAAGTGCTGGGATTACAG*AGAACACTTTTTACAGTTGGCTAGAAGGTCTCTGTGTAGAAAA	UTR/CDS(truncated)
ENSG00000135046	ENSE00003788481	ENSE00003569681	AGCAATTCTCCTGCCTCAGCCTTCGGAGTAGCTGGGATTACAG*ACACTTTTTCAAAAATGGCAATGGTATCAGAATTCCTCAAGCA	UTR/CDS(no-known-start-or-end)
ENSG00000122705	ENSE00001841099	ENSE00001839548	AGAAAAGATTATTCAATAAAGTTTCCTGCCTTTCTGCAAACAT*AAAAAAAAAAAAAAAAAAAAAAAAAAAAAAAAAAAAAAAAAA	UTR/UTR
ENSG00000122705	ENSE00003650505	ENSE00001839548	GAAAAGATTATTCAATAAAGTTTCCTGCCTTTCTGCAAACATA*AAAAAAAAAAAAAAAAAAAAAAAAAAAAAAAAAAAAAAAAAA	UTR/UTR
ENSG00000121310	ENSE00003650505	ENSE00001821702	GAAAAGATTATTCAATAAAGTTTCCTGCCTTTCTGCAAACATA*AAAAAAAAAAAAAAAACAGCAAAACTCTCCCCCGCCAAAAATA	UTR/UTR
ENSG00000051620	ENSE00001841099	ENSE00001630655	AGAAAAGATTATTCAATAAAGTTTCCTGCCTTTCTGCAAACAT*AAAAAAAAAAAAAAAAAAAAAAAAAAAAAAAACGATGGGAGTCC	UTR/UTR
ENSG00000051620	ENSE00003650505	ENSE00001630655	GAAAAGATTATTCAATAAAGTTTCCTGCCTTTCTGCAAACATA*AAAAAAAAAAAAAAAAAAAAAAAAAAAAAAAACGATGGGAGTCC	UTR/UTR
ENSG00000095066	ENSE00001841099	ENSE00002811225	AGAAAAGATTATTCAATAAAGTTTCCTGCCTTTCTGCAAACAT*AAAAAAAAAAAAAAAAAAAAAAAGAAACAAAGATGGTCAGGCAG	UTR/UTR
ENSG00000095066	ENSE00003650505	ENSE00002811225	GAAAAGATTATTCAATAAAGTTTCCTGCCTTTCTGCAAACATA*AAAAAAAAAAAAAAAAAAAAAAAGAAACAAAGATGGTCAGGCAG	UTR/UTR
ENSG00000167004	ENSE00001841099	ENSE00001489318	AGAAAAGATTATTCAATAAAGTTTCCTGCCTTTCTGCAAACAT*AAAAAAAAAAAAAAAAAAAAAAAAAAAAAAAAAAAAAAAAAA	UTR/UTR
ENSG00000167004	ENSE00003650505	ENSE00001489318	GAAAAGATTATTCAATAAAGTTTCCTGCCTTTCTGCAAACATA*AAAAAAAAAAAAAAAAAAAAAAAAAAAAAAAAAAAAAAAAAA	UTR/UTR
ENSG00000135046	ENSE00002064325	ENSE00001472097	CCACTCTGCCCTCCAAAAAGACAAAAAAAAAAAAAAAAAAAAAA*AAAAAAAAAAAAAAAAAAAAAAAGCAATCATTAAGCTTTTCATC	UTR/UTR
ENSG00000135046	ENSE00001545781	ENSE00003569681	AGCAATCCTCCCACTTCAGCCTCCAGAGTAGCTGGGACCACAG*ACACTTTTTCAAAAATGGCAATGGTATCAGAATTCCTCAAGCA	UTR/CDS(no-known-start-or-end)
ENSG00000182718	ENSE00002727312	ENSE00002536879	AGCAGTTCTCCTGCCTCACCCTCCCGAGTAGCTGGGATTACAG*GCGGCCAGGCCCTGTTGAAAATACAATATGTGGGCCATAAAAC	UTR/UTR
ENSG00000135046	ENSE00003096626	ENSE00003569681	AGTGATTCTCGTGCCTCAGCCTCCTGAGTTGCTGGGACTACAG*ACACTTTTTCAAAAATGGCAATGGTATCAGAATTCCTCAAGCA	CDS(truncated)/CDS(no-known-start-or-end)
ENSG00000176809	ENSE00002635959	ENSE00003526242	ATAAAGAAAGAAAGGGATAGATGGAATGAGTTTCATCGTGATT*AATTCTCAATCACAATCCTCTGACAACTGTTGAAGATCCATAT	UTR/CDS(truncated)
ENSG00000135046	ENSE00003650505	ENSE00001472097	GAAAAGATTATTCAATAAAGTTTCCTGCCTTTCTGCAAACATA*AAAAAAAAAAAAAAAAAAAAAAAGCAATCATTAAGCTTTTCATC	UTR/UTR
ENSG00000135046	ENSE00001841099	ENSE00001472097	AGAAAAGATTATTCAATAAAGTTTCCTGCCTTTCTGCAAACAT*AAAAAAAAAAAAAAAAAAAAAAAGCAATCATTAAGCTTTTCATC	UTR/UTR
ENSG00000124942			TTCCCTCTTATCCATCTGCAGGTGAATCTTCAATAAAATGCTTTTGTCAT*TCAAAAAAAAAAAAAAAAATAAATAAATAAAAAGAAATGCAAATCTGAGCC	UTR/intronic
ENSG00000274611	ENSE00003785912	ENSE00003731868	AACATGTATTTAACCAAGTTCCAACAAAAGAATGCTGCCACAG*GATGGACGTGGTAGAGGTCGCGGGCAGTTGGTGGGCACAAGAG	CDS(complete)/UTR
ENSG00000163220	ENSE00002555095	ENSE00001512631	AGTGATTCTCCAGCCTCACCCTCCCGAGTAGCTGGGATTACAG*AAGGAGAATAAGAATGAAAAGGTCATAGAACACATCATGGAGG	UTR/CDS(truncated)
ENSG00000170423	ENSE00002555095	ENSE00001659005	AGTGATTCTCCAGCCTCACCCTCCCGAGTAGCTGGGATTACAG*AACGCCAGCCTGCAGGCCGCCATCACTGATGCTGAGCAGCGTG	UTR/CDS(truncated)
ENSG00000163220	ENSE00003746579	ENSE00001512631	AGTGATTCTCCTGCCTCAGCCTCCTGAGTAGCTGGGATTACAG*AAGGAGAATAAGAATGAAAAGGTCATAGAACACATCATGGAGG	UTR/CDS(truncated)
ENSG00000263001	ENSE00001841099	ENSE00003722138	AGAAAAGATTATTCAATAAAGTTTCCTGCCTTTCTGCAAACAT*AAAAAAAAAAAAGAAAAAAAAGAAAAAAAAAAAGGAGGAGGAGG	UTR/UTR
ENSG00000263001	ENSE00003650505	ENSE00003722138	GAAAAGATTATTCAATAAAGTTTCCTGCCTTTCTGCAAACATA*AAAAAAAAAAAAGAAAAAAAAGAAAAAAAAAAAGGAGGAGGAGG	UTR/UTR
ENSG00000163220	ENSE00002766591	ENSE00001512631	AGTGATTCTTCTGCCTCAGCCTCCTGAGTAGCTGGGATTACAG*AAGGAGAATAAGAATGAAAAGGTCATAGAACACATCATGGAGG	UTR/CDS(truncated)
ENSG00000135046	ENSE00001548454	ENSE00001472097	TGCATGTTTCCTGCTCTTCCCTCATTAAATTGCTTTTAATTCC*AAAAAAAAAAAAAAAAAAAAAAAGCAATCATTAAGCTTTTCATC	UTR/UTR
ENSG00000153107	ENSE00002531730	ENSE00002441208	CATCCACAGCAGTGTTTCTCGGGTTGGAAAAGCCATTGATAAG*GATTCACTTTAAGAGATTTGGAAACTCTTCCCTTTGGAATTGC	out-of-frame
ENSG00000136153			TCTCAACTTCTCTACCCCAAAGAAAAGATTATTCAATAAAGTTTCCTGCC*ATTCTGCAAAAAAAAAAAAAAAATCTTCTAGTTTCTGAGTAACTTTCATCC	UTR/intronic
ENSG00000274611	ENSE00003785912	ENSE00003731868	AACATGTATTTAACCAAGTTCCAACAAAAGAATGCTGCCACAG*GATGGACGTGGTAGAGGTCGCGGGCAGTTGGTGGGCACAAGAG	CDS(complete)/UTR
ENSG00000112297			CTTCTCTACCCCAAAGAAAAGATTATTCAATAAAGTTTCCTGCCTTTCTG*CAAAATTAAAAAAAAAAAAAAAAAGAAAGTATATTCTGTTATTACAGTAAA	UTR/intronic
ENSG00000243716			TTACGTTCATGTAAACAGCATGACGTGAGGCCATGGATGCAGGCATTAAG*GTATGATCTCGTGAAATCTTGAGAGAAACTGAATGACGAATGAAACTATT	CDS(truncated)/UTR
ENSG00000243716	ENSE00001505919	ENSE00002138288	AGGTATACTATGTACCAGAATCAGTTGTTGGAGAAAATTAAAG*GTATGATCTCGTGAAATCTTGAGAGAAACTGAATGACGAATGA	CDS(truncated)/UTR
ENSG00000197930	ENSE00002555095	ENSE00001376586	AGTGATTCTCCAGCCTCACCCTCCCGAGTAGCTGGGATTACAG*AATTTCTACAAGTGTGAAAGAATTAGAAAACTTCAGGAACTTG	UTR/CDS(truncated)
ENSG00000135046	ENSE00002583055	ENSE00003569681	AGTGATTCTCCTCCCTCAGCCTCCCGAGTAGCTGGGATTACAG*ACACTTTTTCAAAAATGGCAATGGTATCAGAATTCCTCAAGCA	UTR/CDS(no-known-start-or-end)
ENSG00000135046	ENSE00003724507	ENSE00001472097	TACAGTTTAGAATATTTATTTGTTAATTTTATTAAAATGCTTT*AAAAAAAAAAAAAAAAAAAAAAAGCAATCATTAAGCTTTTCATC	UTR/UTR
ENSG00000182718	ENSE00000854747	ENSE00002555095	CAGACAATTAAAAGACCTTTAAATCCTTTGGCTTCTGGTCAAG*ATGGAGCCTGGCTCTGTCACCCAGCCTGGAGTGTAGTCGCACG	CDS(truncated)/UTR
ENSG00000259827	ENSE00001841099	ENSE00002589916	AGAAAAGATTATTCAATAAAGTTTCCTGCCTTTCTGCAAACAT*AAAAAAAAAAAAAAAAAAAAAAAAAAAAAAAAAAAAAAAAAA	UTR/exonic(no-known-CDS)
ENSG00000167004	ENSE00001841099	ENSE00001489318	AGAAAAGATTATTCAATAAAGTTTCCTGCCTTTCTGCAAACAT*AAAAAAAAAAAAAAAAAAAAAAAAAAAAAAAAAAAAAAAAAA	UTR/UTR
ENSG00000197822	ENSE00003590701	ENSE00003560198	TTATTGATCCAAATTTCAGCTGAGTATGATCCTTCCAAACTAG*GTTAAAAATGTGTCTGCAGGCACACAGGACGTGCCTTCACCCC	out-of-frame

ENSG00000166762	ENSE00003788646	ENSE00003500886	CATGGCAAAAAGCTACCACCTGCCAGTCTGAAGCACCGAGATG*AATTGCTGGAATCCACAAATACCAAACTATGGCCATTGAAGCT	in-frame
ENSG00000232774	ENSE00003584360	ENSE00003756066	CCCTACCTGGCTCCATGAAGATGCCACAGGGATCCTAAAATCT*GAACCTGATGAAATGCCTGGTCTAACAGGAGGATGCAGAGAAA	CDS(truncated)/exonic(no-known-CDS)
ENSG09000001012			ATGAGATGAAATGAAATGAAATAATGAAATGAAATTGAAATAAATAAATA*AAATTGAAATGAGATGAGATGAAATGATGAGATGATGAAATAAAATGATA	intronic/---
ENSG00000258410			TGATAAGATGACTTGAGATGAGATGAAATGATGAGATGAAATGACAAAAT*GATGAGATGATGAGATGAAATTTTGAGATGAAATGGTGAGTAGAAATGAT	---/intronic
ENSG00000258410			TAAATGAGATGATGAAATTAAATGATGAAATGAAATAATGAAATGGAAAT*GATGAGATGAAATGATGAGATGAATGATGAGATGAAATGAGATGAAATGA	---/intronic
ENSG00000271573			GCAAGTCGACTCCTTCCAGGCTCCAGGAACACCACAAAGCAATATGAAAC*CTGTTCATGAGAGGAGTCAGGAATGCCTTCCACCAAAGAAACGAGACCTC	CDS(truncated)/exonic(no-known-CDS)
ENSG00000274611	ENSE00003785912	ENSE00003731868	AACATGTATTTAACCAAGTTCCAACAAAAGAATGCTGCCACAG*GATGGACGTGGTAGAGGTCGCGGGCAGTTGGTGGGCACAAGAG	CDS(complete)/UTR
ENSG00000153107	ENSE00002531730	ENSE00002441208	CATCCACAGCAGTGTTTCTCGGGTTGGAAAAGCCATTGATAAG*GATTCACTTTAAGAGATTTGGAAACTCTTCCCTTTGGAATTGC	out-of-frame
ENSG00000185873			ATCACACCACTGCACTCCAGCCTGGGCAACAGAGTGAGACCCTGTCTCA*AAAAAAAAAAAAAAAAAAAGGAGAGAAGAAAATAAAGAAGGCTAATATAT	intronic/UTR
ENSG00000185873			CTGTTGTACTCCAGCCAGCCTGGGCAACAGAGTGAGACCCTGTCTCAA*AAAAAAAAAAAAAAAAAAAGGAGAGAAGAAAATAAAGAAGGCTAATATAT	UTR/UTR
ENSG00000135046	ENSE00001447864	ENSE00001472097	CCTGGACATGTACCTGCAGAATAATAAAGTCATCAATACCTCA*AAAAAAAAAAAAAAAAAAAAAAGCAATCATTAAGCTTTTCATC	UTR/UTR
ENSG00000055044			ACCATGTACCAGCACAACAATTCCTAGTCCAGAGAAAAAGAAGAAAAAGA*AAAAAAAAGAGAGAGAACGAGGATTAACAGAAAGGAATTACGATTATATC	exonic(no-known-CDS)/CDS(truncated)
ENSG00000137710			GGTTTGCGTGAGGTCTGGTTTTTTTGGGCTGCAGTATGTAGACAGCAAAG*GTTATTCTACATGGCTTAAACTAAATAAAAAGGTAAAATATGTACGATAA	exonic(no-known-CDS)/CDS(truncated)
ENSG00000137710			TAAAATTGGTTTTCCCTGGAGTGAAATCAGAAATATTTCATTTAATGACA*AAAAAATTTGTTATAAAGCCAATCGACAAAAAGGCACCTGTAAGTAATTT	exonic(no-known-CDS)/CDS(truncated)
ENSG00000143536			ACTCCTCACACCACTTAACAGCCACTTGTTTCATCCCACCTGGGCATTAG*GTTGACTTCAAAGATGCCTCAGTTACTGCAAAACATTAATGGGATCATCG	intergenic/UTR
ENSG00000166762	ENSE00001753134	ENSE00001581568	TCATCTTCACGGCCACCCACAGAAATGAAGCAGAGTGGCCTAG*AGGTATCTGCCGGTACTGAGATGTAAGTGATCAGCTGAAGGGA	CDS(truncated)/UTR
ENSG00000143452	ENSE00002175592	ENSE00003622633	GAAGTGAGGAGCCTCTCCGCCCGGCAGCCACCCCATCTGGGAA*GTTGAATTAAAGAAAATACTTTATCAGAAGAAGATGGCCACTG	exonic(no-known-CDS)/UTR
ENSG00000243716	ENSE00001505919	ENSE00002138288	AGGTATACTATGTACCAGAATCAGTTGTTGGAGAAAATTAAAG*GTATGATCTCGTGAAATCTTGAGAGAAACTGAATGACGAATGA	CDS(truncated)/UTR
ENSG00000278931	ENSE00001649566	ENSE00003759425	ATTGTCTGCATTGGATGACGAACTCTTGAGTGTTGCTGCTAAG*TGTGTCCCCGAGAAAGTGTCAGAGCCTTTACGTGGACCTTCTC	exonic(no-known-CDS)/exonic(no-known-CDS)
ENSG00000091073	ENSE00001841099	ENSE00001385035	AGAAAAGATTATTCAATAAAGTTTCCTGCCTTTCTGCAAACAT*AAAAAAAAAAAAAAAAAAAAAAAAAAAAAAAAAAAAAAAAAAA	UTR/UTR
ENSG00000091073	ENSE00003650505	ENSE00001385035	GAAAAGATTATTCAATAAAGTTTCCTGCCTTTCTGCAAACATA*AAAAAAAAAAAAAAAAAAAAAAAAAAAAAAAAAAAAAAAAAAA	UTR/UTR
ENSG00000055208	ENSE00001447878	ENSE00002529799	ATTCAAAGAGCTGGTGCGAAAAGATCTGCAAAATTTTCTCAAG*AGTTTCGCTCTTGTTGCCCAGGCTGGAGTGCAATGGTGTGATC	CDS(truncated)/UTR
ENSG00000135046	ENSE00001545781	ENSE00003569681	AGCAATCCTCCCACTTCAGCCTCCAGAGTAGCTGGGACCACAG*ACACTTTTTCAAAAATGGCAATGGTATCAGAATTCCTCAAGCA	UTR/CDS(no-known-start-or-end)
ENSG00000206561	ENSE00003693947	ENSE00001498676	TATCAACATCATGATTGAGCCACAAGCCACACGGAAGGCCCAG*CCCTTCCCAGCCTGGATCAGAAGAAGCGTGGTGGCCACAAAGC	out-of-frame
ENSG00000107789	ENSE00001782026	ENSE00000909243	AATGATCCGCCTGCCTCAGCCTCCAAAGTAGCTGAGACTACAG*ATATGGAGTTTGGACCTCCAACAGTTAATGATAAACTAATGAG	exonic(no-known-CDS)/CDS(truncated)
ENSG00000135046	ENSE00001841099	ENSE00001472097	AGAAAAGATTATTCAATAAAGTTTCCTGCCTTTCTGCAAACAT*AAAAAAAAAAAAAAAAAAAAAAGCAATCATTAAGCTTTTCATC	UTR/UTR
ENSG00000135046	ENSE00003650505	ENSE00001472097	GAAAAGATTATTCAATAAAGTTTCCTGCCTTTCTGCAAACATA*AAAAAAAAAAAAAAAAAAAAAAGCAATCATTAAGCTTTTCATC	UTR/UTR
ENSG00000135046	ENSE00001548454	ENSE00001472097	TGCATGTTTCCTGCTCTTCCCTCATTAAATTGCTTTTAATTCC*AAAAAAAAAAAAAAAAAAAAAAAGCAATCATTAAGCTTTTCATC	UTR/UTR
ENSG00000135046	ENSE00001901937	ENSE00001472097	GCATGTTTCCTGCTCTTCCCTCATTAAATTGCTTTTAATTCCA*AAAAAAAAAAAAAAAAAAAAAAAGCAATCATTAAGCTTTTCATC	UTR/UTR
ENSG00000274611	ENSE00003785912	ENSE00003731868	AACATGTATTTAACCAAGTTCCAACAAAAGAATGCTGCCACAG*GATGGACGTGGTAGAGGTCGCGGGCAGTTGGTGGGCACAAGAG	CDS(complete)/UTR
ENSG00000169469			AATACACCAGTTCTAAGGGACCATACAGAGTATTCCTCTCTTCACACCAG*GACCAGTCACTGTTGCAGCATGAGTTCCCAGCAGCAGAAGCAGCCTTGCA	intergenic/UTR
ENSG00000118898			TTCTCTGCTCTTTGCTCTTTGTAATTTCTAAATAAAGCAGGTTTTAGAAT*AAAAAAAAATAAAAAAAAAAAGAGTGCCATCCAGCCGGTTTTGGTAGTTT	UTR/intronic
ENSG00000196531			CTTCAAGAGCCATTTAATAAATATGGCAGAACTATATATGTGTCTTAAAC*CTCAAAGTAAATTTTCCTTGAGAAATAATTTATGTTGAAAAGATTTCCTG	UTR/UTR
ENSG00000138311	ENSE00001752289	ENSE00001312443	CAAAGATTGAAAACAAGATAAAGATGAAGGACTCTACAAAATA*GACTTTTAGAGTAATGCAACAGAAGGCTTTTGAGGAAAGCAGA	exonic(no-known-CDS)/UTR
ENSG00000051620	ENSE00001841099	ENSE00001630655	AGAAAAGATTATTCAATAAAGTTTCCTGCCTTTCTGCAAACAT*AAAAAAAAAAAAAAAAAAAAAAAAAAAAAAAACGATGGGAGTCC	UTR/UTR
ENSG00000051620	ENSE00003650505	ENSE00001630655	GAAAAGATTATTCAATAAAGTTTCCTGCCTTTCTGCAAACATA*AAAAAAAAAAAAAAAAAAAAAAAAAAAAAAAACGATGGGAGTCC	UTR/UTR
ENSG00000163209			CCTTCACCAGATCCCAGAGGCTGAACACCTCGACCTTCTCTGCACAGCAG*GTCCAGCATCCTTTGAAGCATGAGTTCTTACCAGCAGAAGCAGACCTTTA	intergenic/UTR
ENSG00000185829	ENSE00003662350	ENSE00003806456	TCGTCAGCAAACAGACATTTACAAACAGATACGTGCTAATAAG*GTTTCTGTGTGGAGACAGTAGAATATAAAAATAACACCTTCGC	out-of-frame

ENSG00000228696	ENSE00003662350	ENSE00002370389	TCGTCAGCAAACAGACATTTACAAACAGATACGTGCTAATAAG*GTTTCTGTGTGGAGACAGTAGAATATAAAAATAACACCTTCGC	out-of-frame
ENSG00000153107	ENSE00002531730	ENSE00002441208	CATCCACAGCAGTGTTTCTCGGGTTGGAAAAGCCATTGATAAG*GATTCACTTTAAGAGATTTGGAAACTCTTCCCTTTGGAATTGC	out-of-frame
ENSG00000135046	ENSE00001447817	ENSE00001472097	CCTTTGAGTTTTGTATTCAATAAACTTTTTTTGTCTGTTGATA*AAAAAAAAAAAAAAAAAAAAAAGCAATCATTAAGCTTTTCATC	UTR/UTR
ENSG00000163220	ENSE00002555095	ENSE00001512631	AGTGATTCTCCAGCCTCACCCTCCCGAGTAGCTGGGATTACAG*AAGGAGAATAAGAATGAAAAGGTCATAGAACACATCATGGAGG	UTR/CDS(truncated)
ENSG00000263001	ENSE00003650505	ENSE00003722138	GAAAAGATTATTCAATAAAGTTTCCTGCCTTTCTGCAAACATA*AAAAAAAAAAAAGAAAAAAAAGAAAAAAAAAAAGGAGGAGGAGG	UTR/UTR
ENSG00000263001	ENSE00001841099	ENSE00003722138	AGAAAAGATTATTCAATAAAGTTTCCTGCCTTTCTGCAAACAT*AAAAAAAAAAAAGAAAAAAAAGAAAAAAAAAAAGGAGGAGGAGG	UTR/UTR
ENSG00000135046	ENSE00001704505	ENSE00003569681	AGTGATCTTCCCACCTCAGCCTCCCAAGTAGCTGGGACTACAG*ACACTTTTTCAAAAATGGCAATGGTATCAGAATTCCTCAAGCA	UTR/CDS(no-known-start-or-end)
ENSG00000135046	ENSE00002064325	ENSE00001472097	CCACTCTGCCCTCCAAAAAGACAAAAAAAAAAAAAAAAAAAAA*AAAAAAAAAAAAAAAAAAAAAAGCAATCATTAAGCTTTTCATC	UTR/UTR
ENSG00000176809	ENSE00002635959	ENSE00003526242	ATAAAGAAAGAAAGGGATAGATGGAATGAGTTTCATCGTGATT*AATTCTCAATCACAATCCTCTGACAACTGTTGAAGATCCATAT	UTR/CDS(truncated)
ENSG00000176809	ENSE00003258850	ENSE00001540364	GAGAGGAGACTTCCTTGCTTCTTTGGAGAATGATATCAAACCA*GCTGAAGTGCAATGTTGTGATCTCGGCTCACTGCAACCTCTGC	CDS(truncated)/UTR
ENSG00000243716	ENSE00001505919	ENSE00002138288	AGGTATACTATGTACCAGAATCAGTTGTTGGAGAAAATTAAAG*GTATGATCTCGTGAAATCTTGAGAGAAACTGAATGACGAATGA	CDS(truncated)/UTR
ENSG09000001012			CTGTGAGTTGAATGTTGTGTTCATAGTATCTGCCAAAACAGAAAGAAAAA*AAAACAAAATATTTTGATAAGAAGTTAAAGCTTTGTATATAATATACCTT	intronic/---
ENSG00000258410			GAGGAAATGATGAGATGAGGAGATGAAATGATGAGATGAATTGAGATGAA*ATGAGATGAAAAATGATATGAAAAATGATATCAAAAATATGAGATGAAAC	---/intronic
ENSG00000274611	ENSE00003785912	ENSE00003731868	AACATGTATTTAACCAAGTTCCAACAAAAGAATGCTGCCACAG*GATGGACGTGGTAGAGGTCGCGGGCAGTTGGTGGGCACAAGAG	CDS(complete)/UTR
ENSG00000188234	ENSE00002466657	ENSE00002848560	CCAGAGGCAAGCACAATATTCCAGAGGAACTCTCAAACAGATG*TTGTAGAAATAAGAAGAAGCAACTGTACAAACCATGTATCTAC	exonic(no-known-CDS)/CDS(truncated)
ENSG00000188234	ENSE00002488090	ENSE00003645446	AAACCATTGTGACCTTGGAGATACCTCATCATATCACACAAAG*AGATGCAGATAGATCTTTGAGCATACCTGATGAACAGTTACAC	exonic(no-known-CDS)/CDS(truncated)
ENSG00000188234	ENSE00002516078	ENSE00003786965	AACAGATGTTGTAGAAATAAGAAGAAGCAACTGTACAAACCAT*TGTGACCTTGGAGATACCTCATCATATCACACAAAGAGATGCA	exonic(no-known-CDS)/CDS(truncated)
ENSG00000243289	ENSE00003564276	ENSE00002516078	CCAGAGGCAAGCACAATATTCCAGAGGAACTCTCAAACAGATG*TTGTAGAAATAAGAAGAAGCAACTGTACAAACCATTGTGACCT	CDS(truncated)/exonic(no-known-CDS)
ENSG00000277103			ATGAACCACACGGCCAGCTTCTGGATGCAGGCACAGTGCCACATTTTTTG*TCACTTCCTGATGTGCCACACCAGCACTGAAGAGACAGCCTGGAGACAGG	exonic(no-known-CDS)/exonic(no-known-CDS)
ENSG00000163209			CCTTCACCAGATCCCAGAGGCTGAACACCTCGACCTTCTCTGCACAGCAG*GTCCAGCATCCTTTGAAGCATGAGTTCTTACCAGCAGAAGCAGACCTTTA	intergenic/UTR
ENSG00000267709			TGACTCCACCGCCTATCCCAAAACCTATAAGAACTAATGATAATCCCACC*ACCCTTTGATGACTCTCTTTTTGGACTCAGCCCGCCTGCACCCAGGTGAA	UTR/exonic(no-known-CDS)
ENSG00000166762	ENSE00003788646	ENSE00003500886	CATGGCAAAAAGCTACCACCTGCCAGTCTGAAGCACCGAGATG*AATTGCTGGAATCCACAAATACCAAACTATGGCCATTGAAGCT	in-frame
ENSG00000135046	ENSE00001447864	ENSE00001472097	CCTGGACATGTACCTGCAGAATAATAAAGTCATCAATACCTCA*AAAAAAAAAAAAAAAAAAAAAAGCAATCATTAAGCTTTTCATC	UTR/UTR
ENSG00000135046	ENSE00001841099	ENSE00001472097	AGAAAAGATTATTCAATAAAGTTTCCTGCCTTTCTGCAAACAT*AAAAAAAAAAAAAAAAAAAAAAGCAATCATTAAGCTTTTCATC	UTR/UTR
ENSG00000135046	ENSE00003650505	ENSE00001472097	GAAAAGATTATTCAATAAAGTTTCCTGCCTTTCTGCAAACATA*AAAAAAAAAAAAAAAAAAAAAAGCAATCATTAAGCTTTTCATC	UTR/UTR
ENSG00000135046	ENSE00001548454	ENSE00001472097	TGCATGTTTCCTGCTCTTCCCTCATTAAATTGCTTTTAATTCC*AAAAAAAAAAAAAAAAAAAAAAGCAATCATTAAGCTTTTCATC	UTR/UTR
ENSG00000135046	ENSE00001901937	ENSE00001472097	GCATGTTTCCTGCTCTTCCCTCATTAAATTGCTTTTAATTCCA*AAAAAAAAAAAAAAAAAAAAAAGCAATCATTAAGCTTTTCATC	UTR/UTR
ENSG09000001012			AGATGAGATGAAATAATGAGATAAAATGATGAGATGAAATGAGATGAATG*ATGAGATGAAATGATGAGATGCAATGATGAGATGAAATGATGAGATGAGA	intronic/---
ENSG00000258410			TGAAATGAAATGAAATAATGAAATGAAATAATGAAATGATGAAATAATGA*AAATGGAAATGATGAGATGAGAAGAAATGATGAGATGAAATGATGAAATG	---/intronic
ENSG00000274611	ENSE00003785912	ENSE00003731868	AACATGTATTTAACCAAGTTCCAACAAAAGAATGCTGCCACAG*GATGGACGTGGTAGAGGTCGCGGGCAGTTGGTGGGCACAAGAG	CDS(complete)/UTR
ENSG00000153107	ENSE00002531730	ENSE00002441208	CATCCACAGCAGTGTTTCTCGGGTTGGAAAAGCCATTGATAAG*GATTCACTTTAAGAGATTTGGAAACTCTTCCCTTTGGAATTGC	out-of-frame
ENSG00000163220	ENSE00002555095	ENSE00001512631	AGTGATTCTCCAGCCTCACCCTCCCGAGTAGCTGGGATTACAG*AAGGAGAATAAGAATGAAAAGGTCATAGAACACATCATGGAGG	UTR/CDS(truncated)
ENSG00000135046			TTTAAGGGTTTGTGGAAAATGAAAATTAGGATTTCATGATTTTTTTTTTT*AAAAACAAAAAAAAAAAAACAAAAAACCCTCAGAGCTAAATGAAGAAAGA	UTR/UTR
ENSG00000243716	ENSE00001505919	ENSE00002138288	AGGTATACTATGTACCAGAATCAGTTGTTGGAGAAAATTAAAG*GTATGATCTCGTGAAATCTTGAGAGAAACTGAATGACGAATGA	CDS(truncated)/UTR
ENSG00000243716			TTACGTTCATGTAAACAGCATGACGTGAGGCCATGGATGCAGGCATTAAG*GTATGATCTCGTGAAATCTTGAGAGAAACTGAATGACGAATGAAACTATT	CDS(truncated)/UTR
ENSG00000163220			ATAAAGTTAATACCAAACTTGCAAACACTCTGTGTGGCTCCTCGGCTTTG*ACAGAGTGCAAGACGATGACTTGCAAAATGTCGCAGCTGGAACGCAACAT	intergenic/UTR
ENSG00000263001	ENSE00001841099	ENSE00003722138	AGAAAAGATTATTCAATAAAGTTTCCTGCCTTTCTGCAAACAT*AAAAAAAAAAAAGAAAAAAAAGAAAAAAAAAAAGGAGGAGGAGG	UTR/UTR
ENSG00000135046	ENSE00001834474	ENSE00001472097	AACGAGCTGTGCTTGCAAAGACTTCATAGTTCCCAAGAATTAA*AAAAAAAAAAAAAAAAAAAAAAGCAATCATTAAGCTTTTCATC	UTR/UTR
ENSG00000135046	ENSE00001935261	ENSE00001472097	GAAACGAGCTGTGCTTGCAAAGACTTCATAGTTCCCAAGAATT*AAAAAAAAAAAAAAAAAAAAAAGCAATCATTAAGCTTTTCATC	UTR/UTR
ENSG00000135046	ENSE00001434207	ENSE00001472097	TGCAAAGACTTCATAGTTCCCAAGAATTAAAAAAAAAAAAAAA*AAAAAAAAAAAAAAAAAAAAAAGCAATCATTAAGCTTTTCATC	UTR/UTR
ENSG00000089737	ENSE00001548454	ENSE00002510739	TGCATGTTTCCTGCTCTTCCCTCATTAAATTGCTTTTAATTCC*AAAAAAAAAAAAAAAAAAAAGAAATTACCTAGGCATGGTGGTG	UTR/UTR
ENSG00000089737	ENSE00001901937	ENSE00002510739	GCATGTTTCCTGCTCTTCCCTCATTAAATTGCTTTTAATTCCA*AAAAAAAAAAAAAAAAAAAAGAAATTACCTAGGCATGGTGGTG	UTR/UTR
ENSG00000185829	ENSE00003662350	ENSE00003806456	TCGTCAGCAAACAGACATTTACAAACAGATACGTGCTAATAAG*GTTTCTGTGTGGAGACAGTAGAATATAAAAATAACACCTTCGC	out-of-frame

ENSG00000228696	ENSE00003662350	ENSE00002370389	TCGTCAGCAAACAGACATTTACAAACAGATACGTGCTAATAAG*GTTTCTGTGTGGAGACAGTAGAATATAAAAATAACACCTTCGC	out-of-frame
ENSG09000001012			CTGTGAGTTGAATGTTGTGTTCATAGTATCTGCCAAAACAGAAAGAAAAA*AAAACAAAATATTTTGATAAGAAGTTAAAGCTTTGTATATAATATACCTT	intronic/---
ENSG00000258410			TGATAAGATGACTTGAGATGAGATGAAATGATGAGATGAAATGACAAAAT*GATGAGATGATGAGATGAAATTTTGAGATGAAATGGTGAGTAGAAATGAT	---/intronic
ENSG00000258410			TGATATGAAATGATGAGATGAGATGGGATGAGATGAAATGAGATAAAATG*ATGAGATGAAATGATGAGATGAAATGATGGGGTGAAGTGATGCACTGTCA	---/intronic
ENSG00000274611	ENSE00003785912	ENSE00003731868	AACATGTATTTAACCAAGTTCCAACAAAAGAATGCTGCCACAG*GATGGACGTGGTAGAGGTCGCGGGCAGTTGGTGGGCACAAGAG	CDS(complete)/UTR
ENSG00000153107	ENSE00002531730	ENSE00002441208	CATCCACAGCAGTGTTTCTCGGGTTGGAAAAGCCATTGATAAG*GATTCACTTTAAGAGATTTGGAAACTCTTCCCTTTGGAATTGC	out-of-frame
ENSG00000271573			GCAAGTCGACTCCTTCCAGGCTCCAGGAACACCACAAAGCAATATGAAAC*CTGTTCATGAGAGGAGTCAGGAATGCCTTCCACCAAAGAAACGAGACCTC	CDS(truncated)/exonic(no-known-CDS)
ENSG00000135046	ENSE00001901937	ENSE00001472097	GCATGTTTCCTGCTCTTCCCTCATTAAATTGCTTTTAATTCCA*AAAAAAAAAAAAAAAAAAAAAAAGCAATCATTAAGCTTTTCATC	UTR/UTR
ENSG00000135046	ENSE00001548454	ENSE00001472097	TGCATGTTTCCTGCTCTTCCCTCATTAAATTGCTTTTAATTCC*AAAAAAAAAAAAAAAAAAAAAAAGCAATCATTAAGCTTTTCATC	UTR/UTR
ENSG00000185873			TATCACACCACTGCACTCCAGCCTGGGCAACAGAGTGAGACCCTGTCTCA*AAAAAAAAAAAAAAAAAAAAGGAGAGAAGAAAATAAAGAAGGCTAATATAT	intronic/UTR
ENSG00000185873			TAACTGTTGTACTCCAGCCAGCCTGGGCAACAGAGTGAGACCCTGTCTCA*AAAAAAAAAAAAAAAAAAAAGGAGAGAAGAAAATAAAGAAGGCTAATATAT	UTR/UTR
ENSG00000237955			ACCCTAAAATCTATGTGTTTTAGACTTAGACTTTTTATTGCCCCCCCCCC*TTTTTTTTTTGAGACGGAGTCTCGCTCTGTCGCCCAGGCTGGAGTGCAGT	UTR/intergenic
ENSG00000078124			GCCCAGGAGTTTGAGGGCAGCCTGGGAAACATGGCTAAATCCGTGTTC*AAAATACAAAAATTAGCTAGGCATGGTGGCGCACGCCTGTAGTCCCAGC	UTR/intronic
ENSG00000260158			GAGTTCACAAGCAACCTGGGCAACATAGCAAGACCCAGTCTCTATCAAAA*TTAAAAAAAAAAAAGTGTTCCTATTTCTCCACATCCTCTCCAGCACCTGT	exonic(no-known-CDS)/exonic(no-known-CDS)
ENSG00000134531			CCGCTAATGTAGAGGATTATTTATTTTCATTTCATTACCATTCGTGTAAG*TTGTGTGTGTGTGTGTGTGTGTGTGTGTGTGTGTGTGGTGTGTAAGAGGG	UTR/intronic
ENSG00000075790	ENSE00003520906	ENSE00003577399	TTAAAGGAAGCAGAAAATGTGTGGCGGATTACTGGGACAGATG*GTGTGAAGAAAAAAATGACACTCCAATGGGCTGCAGTGGCAAC	CDS(truncated)/UTR
ENSG00000185829	ENSE00003662350	ENSE00003806456	TCGTCAGCAAACAGACATTTACAAACAGATACGTGCTAATAAG*GTTTCTGTGTGGAGACAGTAGAATATAAAAATAACACCTTCGC	out-of-frame
ENSG00000228696	ENSE00003662350	ENSE00002370389	TCGTCAGCAAACAGACATTTACAAACAGATACGTGCTAATAAG*GTTTCTGTGTGGAGACAGTAGAATATAAAAATAACACCTTCGC	out-of-frame
ENSG00000174564	ENSE00003784606	ENSE00002319932	AGCAATTCTCCTGCCTCAGCCTCCCAAGTAGCTGGGATTACAG*ATGAAGTGGCCATTCTGCCTGCCCCTCAGAACCTCTCTGTACT	exonic(no-known-CDS)/CDS(truncated)
ENSG00000163009	ENSE00003659027	ENSE00001489842	GTTTGTGGCAGACAGACTTATGCTGGAACTGGGTTTTAGCAAG*GTGCTGGGAGACCGTGAAGTGCAAAGCAGATGGAGTCCAGGCC	CDS(truncated)/exonic(no-known-CDS)
ENSG00000135046	ENSE00001841099	ENSE00001472097	AGAAAAGATTATTCAATAAAGTTTCCTGCCTTTCTGCAAACAT*AAAAAAAAAAAAAAAAAAAAAAAGCAATCATTAAGCTTTTCATC	UTR/UTR
ENSG00000135046	ENSE00003650505	ENSE00001472097	GAAAAGATTATTCAATAAAGTTTCCTGCCTTTCTGCAAACATA*AAAAAAAAAAAAAAAAAAAAAAAGCAATCATTAAGCTTTTCATC	UTR/UTR
ENSG00000274611	ENSE00003785912	ENSE00003731868	AACATGTATTTAACCAAGTTCCAACAAAAGAATGCTGCCACAG*GATGGACGTGGTAGAGGTCGCGGGCAGTTGGTGGGCACAAGAG	CDS(complete)/UTR
ENSG00000135046	ENSE00001447864	ENSE00001472097	CCTGGACATGTACCTGCAGAATAATAAAGTCATCAATACCTCA*AAAAAAAAAAAAAAAAAAAAAAAGCAATCATTAAGCTTTTCATC	UTR/UTR
ENSG00000135046	ENSE00001548454	ENSE00001472097	TGCATGTTTCCTGCTCTTCCCTCATTAAATTGCTTTTAATTCC*AAAAAAAAAAAAAAAAAAAAAAAGCAATCATTAAGCTTTTCATC	UTR/UTR
ENSG00000135046	ENSE00001901937	ENSE00001472097	GCATGTTTCCTGCTCTTCCCTCATTAAATTGCTTTTAATTCCA*AAAAAAAAAAAAAAAAAAAAAAAGCAATCATTAAGCTTTTCATC	UTR/UTR
ENSG09000001012			GATGAAATGATGAGATGAGATAAAATGAGATGAAATGATGAGATGAAATG*ATGAGATGAGATGAAATGAGATGAAATATGATGAGATGAAATGACATAAT	intronic/---
ENSG00000258410			AAATGATGAGATGTGATGAGATGAAATGATGAGATGAGATGACATGAAAT*AATAAAATGATAAAATGATGAGATGTGATGAGATGAAATGATGAGATGAG	---/intronic
ENSG00000243716	ENSE00001505919	ENSE00002138288	AGGTATACTATGTACCAGAATCAGTTGTTGGAGAAAATTAAAG*GTATGATCTCGTGAAATCTTGAGAGAAACTGAATGACGAATGA	CDS(truncated)/UTR
ENSG00000067182	ENSE00003589571	ENSE00003524766	GGAGCTGAACTACAAAACCAATTCTGAGTCTCCCTCTGTCACG*GTGCTCCTGGAGCTGTTGGTGGGAATATACCCCTCAGGGGTTA	in-frame
ENSG00000197822	ENSE00003590701	ENSE00003560198	TTATTGATCCAAATTTCAGCTGAGTATGATCCTTCCAAACTAG*GTTAAAAATGTGTCTGCAGGCACACAGGACGTGCCTTCACCCC	out-of-frame
ENSG00000153107	ENSE00002531730	ENSE00002441208	CATCCACAGCAGTGTTTCTCGGGTTGGAAAAGCCATTGATAAG*GATTCACTTTAAGAGATTTGGAAACTCTTCCCTTTGGAATTGC	out-of-frame
ENSG00000282911			CCTGTTAGATGAGCTCCTGTCAACCCCAGAGTTTCAGCAAAAGGCACAAA*CTTTCCTAGATCCGGCGCCACTGGGGGAGCTGAAGGACGTGGAAGAGCCC	exonic(no-known-CDS)/exonic(no-known-CDS)
ENSG00000163032			CCCTAAAATCTATGTGTTTTAGACTTAGACTTTTTATTGCCCCCCCCCCC*TTTTTTTTTGAGACGGAGTCTCGCTCTGTCGCCCAAGCTGGAGTGCAGTG	UTR/intronic
ENSG00000169469			AATACACCAGTTCTAAGGGACCATACAGAGTATTCCTCTCTTCACACCAG*GACCAGTCACTGTTGCAGCATGAGTTCCCAGCAGCAGAAGCAGCCTTGCA	intergenic/UTR
ENSG00000075624			CCAGAGCAAGGATATTCTTTATCTTGTTTTTTTTTGTTTTGTTTTGTTT*TTTTTTTTTTTTTTTTGGCTTGACTCAGGATTTAAAAACTGGAACGGTGA	intronic/UTR
ENSG00000075624			TTTTTTTTTTTTTTTTTTTTTTTTTTTTTTTTTTTTTTTTTTTTTTTTTT*GCTTGACTCAGGATTTAAAAACTGGAACGGTGAAGGTGACAGCAGTCGG	intronic/UTR
ENSG00000075624			AGCTCAACAGTCGCCACGTTTTTTTTTTTTTTTTTTTTTTTTTTTTTTTT*TTTTTTTTTTTTTTTGGCTTGACTCAGGATTTAAAAACTGGAACGGTGA	intronic/UTR
ENSG00000075624			TATAGGCAAATACCACAACACCTGGCTTTTTTTTTTTTTTTTTTTTTTTT*TTTTGGTTTTTTTTTTTTTTTTGGCTTGACTCAGGATTTAAAAACTGGAA	intronic/UTR

ENSG00000049239	ENSE00001405037	ENSE00000386080	CTCGGCCTTGGAGAGCAGCGGCGGCGGCGGCACCCCGGGCGCG*GCCGCACCAGCTTCTATGAGGAGTACGGTGTCATTCGCGACGT	UTR/CDS(truncated)
ENSG00000135046	ENSE00001937537	ENSE00001472097	TCCTATAGAGATGACTTTAAAAGGAAAAAAAAAAAAAAAAAAAA*AAAAAAAAAAAAAAAAAAAAAAGCAATCATTAAGCTTTTCATC	UTR/UTR
ENSG00000135404	ENSE00001841099	ENSE00002402992	AGAAAAGATTATTCAATAAAGTTTCCTGCCTTTCTGCAAACAT*AAAAAAAAAAAAAAAAAAAAAAGAATTCTCATCTCCATATTGA	UTR/UTR
ENSG00000135404	ENSE00003650505	ENSE00002402992	GAAAAGATTATTCAATAAAGTTTCCTGCCTTTCTGCAAACATA*AAAAAAAAAAAAAAAAAAAAAAGAATTCTCATCTCCATATTGA	UTR/UTR
ENSG00000167004	ENSE00001841099	ENSE00001489318	AGAAAAGATTATTCAATAAAGTTTCCTGCCTTTCTGCAAACAT*AAAAAAAAAAAAAAAAAAAAAAAAAAAAAAAAAAAAAAAAAAA	UTR/UTR
ENSG00000167004	ENSE00003650505	ENSE00001489318	GAAAAGATTATTCAATAAAGTTTCCTGCCTTTCTGCAAACATA*AAAAAAAAAAAAAAAAAAAAAAAAAAAAAAAAAAAAAAAAAAA	UTR/UTR
ENSG00000135046	ENSE00001592316	ENSE00003569681	AGCGATTCTTCTGCCTCAGCCTCCTGAGTAGCTGGGATTACAG*ACACTTTTTCAAAAATGGCAATGGTATCAGAATTCCTCAAGCA	UTR/CDS(no-known-start-or-end)
ENSG00000135046	ENSE00002512367	ENSE00003569681	AGTGATTCTCCTGCCTCGGCCTCCTGAGTAACTGGGACTACAG*ACACTTTTTCAAAAATGGCAATGGTATCAGAATTCCTCAAGCA	UTR/CDS(no-known-start-or-end)
ENSG00000237172	ENSE00002591400	ENSE00001593945	AGCCTGGCGGGCGTGGGAACCCAGGCCCCGCCGAGGCGGCCAG*GCTGCCGGTCCCGGCGGTGCGACACGGCCGGGAGGAGGAGAAC	UTR/UTR
ENSG00000135046	ENSE00002727312	ENSE00003569681	AGCAGTTCTCCTGCCTCACCCTCCCGAGTAGCTGGGATTACAG*ACACTTTTTCAAAAATGGCAATGGTATCAGAATTCCTCAAGCA	UTR/CDS(no-known-start-or-end)
ENSG00000176809	ENSE00003258850	ENSE00001540364	GAGAGGAGACTTCCTTGCTTCTTTGGAGAATGATATCAAACCA*GCTGAAGTGCAATGTTGTGATCTCGGCTCACTGCAACCTCTGC	CDS(truncated)/UTR
ENSG00000135046	ENSE00001841099	ENSE00001472097	AGAAAAGATTATTCAATAAAGTTTCCTGCCTTTCTGCAAACAT*AAAAAAAAAAAAAAAAAAAAAAGCAATCATTAAGCTTTTCATC	
ENSG00000135046	ENSE00003650505	ENSE00001472097	GAAAAGATTATTCAATAAAGTTTCCTGCCTTTCTGCAAACATA*AAAAAAAAAAAAAAAAAAAAAAGCAATCATTAAGCTTTTCATC	
ENSG00000135046	ENSE00001548454	ENSE00001472097	TGCATGTTTCCTGCTCTTCCCTCATTAAATTGCTTTTAATTCC*AAAAAAAAAAAAAAAAAAAAAAGCAATCATTAAGCTTTTCATC	
ENSG00000185873			TATCACACCACTGCACTCCAGCCTGGGCAACAGAGTGAGACCCTGTCTCA*AAAAAAAAAAAAAAAAAAAGGAGAGAAGAAAATAAAGAAGGCTAATATAT	
ENSG00000185873			ACTGTTGTACTCCAGCCAGCCTGGGCAACAGAGTGAGACCCTGTCTCA*AAAAAAAAAAAAAAAAAAAGGAGAGAAGAAAATAAAGAAGGCTAATATAT	
ENSG00000274611	ENSE00003785912	ENSE00003731868	AACATGTATTTAACCAAGTTCCAACAAAAGAATGCTGCCACAG*GATGGACGTGGTAGAGGTCGCGGGCAGTTGGTGGGCACAAGAG	
ENSG00000105808	ENSE00001758063	ENSE00003527553	TCCGGCTGCAGCCCGACCAGTCCAAGAGCCGGCAGCATGACGA*GGCCCAGGGCAGCTGATCCCACTCATCGAGGAGACAACCAGCA	
ENSG00000136153			AGGTGCCTGCTTTGGGGGTGCTGGAGGCTTTGGCACTGGTGGCTTTGGTG*CCGGCGCCTTCGCAGCCGGAGCGGAAGCCGGAGTTGTGGGAGGCCCGCGT	
ENSG00000153107	ENSE00002531730	ENSE00002441208	CATCCACAGCAGTGTTTCTCGGGTTGGAAAAGCCATTGATAAG*GATTCACTTTAAGAGATTTGGAAACTCTTCCCTTTGGAATTGC	
ENSG00000243716			TTACGTTCATGTAAACAGCATGACGTGAGGCCATGGATGCAGGCATTAAG*GTATGATCTCGTGAAATCTTGAGAGAAACTGAATGACGAATGAAACTATT	
ENSG00000243716			ACGTTCATGTAAACAGCATGACGTGAGGCCATGGATGCAGGCATTAAGGT*TTCAGGCACAGAACTGTATATCCAATAATAGTGAAATGGATCCCACTAAT	
ENSG00000243716	ENSE00001505919	ENSE00002138288	AGGTATACTATGTACCAGAATCAGTTGTTGGAGAAAATTAAAG*GTATGATCTCGTGAAATCTTGAGAGAAACTGAATGACGAATGA	
ENSG00000135404	ENSE00001841099	ENSE00002402992	AGAAAAGATTATTCAATAAAGTTTCCTGCCTTTCTGCAAACAT*AAAAAAAAAAAAAAAAAAAAAAGAATTCTCATCTCCATATTGA	
ENSG00000135404	ENSE00003650505	ENSE00002402992	GAAAAGATTATTCAATAAAGTTTCCTGCCTTTCTGCAAACATA*AAAAAAAAAAAAAAAAAAAAAAGAATTCTCATCTCCATATTGA	
ENSG00000051620	ENSE00001841099	ENSE00001630655	AGAAAAGATTATTCAATAAAGTTTCCTGCCTTTCTGCAAACAT*AAAAAAAAAAAAAAAAAAAAAAAAAAAAAAAAAACGATGGGAGTCC	
ENSG00000051620	ENSE00003650505	ENSE00001630655	GAAAAGATTATTCAATAAAGTTTCCTGCCTTTCTGCAAACATA*AAAAAAAAAAAAAAAAAAAAAAAAAAAAAAAAAACGATGGGAGTCC	
ENSG00000242086	ENSE00001841099	ENSE00003711611	AGAAAAGATTATTCAATAAAGTTTCCTGCCTTTCTGCAAACAT*AAAAAAAAAAAAAAAAAAAAAAAAAAAAAAAAAAAAAAAAAAA	
ENSG00000242086	ENSE00003650505	ENSE00003711611	GAAAAGATTATTCAATAAAGTTTCCTGCCTTTCTGCAAACATA*AAAAAAAAAAAAAAAAAAAAAAAAAAAAAAAAAAAAAAAAAAA	
ENSG00000135046	ENSE00002064325	ENSE00001472097	CCACTCTGCCCTCCAAAAAGACAAAAAAAAAAAAAAAAAAAAA*AAAAAAAAAAAAAAAAAAAAAAGCAATCATTAAGCTTTTCATC	
ENSG00000185829	ENSE00003662350	ENSE00003806456	TCGTCAGCAAACAGACATTTACAAACAGATACGTGCTAATAAG*GTTTCTGTGTGGAGACAGTAGAATATAAAAATAACACCTTCGC	
ENSG00000228696	ENSE00003662350	ENSE00002370389	TCGTCAGCAAACAGACATTTACAAACAGATACGTGCTAATAAG*GTTTCTGTGTGGAGACAGTAGAATATAAAAATAACACCTTCGC	
ENSG00000135046	ENSE00003650505	ENSE00001472097	GAAAAGATTATTCAATAAAGTTTCCTGCCTTTCTGCAAACATA*AAAAAAAAAAAAAAAAAAAAAAGCAATCATTAAGCTTTTCATC	UTR/UTR
ENSG00000135046	ENSE00001841099	ENSE00001472097	AGAAAAGATTATTCAATAAAGTTTCCTGCCTTTCTGCAAACAT*AAAAAAAAAAAAAAAAAAAAAAGCAATCATTAAGCTTTTCATC	UTR/UTR
ENSG00000135046	ENSE00001548454	ENSE00001472097	TGCATGTTTCCTGCTCTTCCCTCATTAAATTGCTTTTAATTCC*AAAAAAAAAAAAAAAAAAAAAAGCAATCATTAAGCTTTTCATC	UTR/UTR
ENSG00000135046	ENSE00001901937	ENSE00001472097	GCATGTTTCCTGCTCTTCCCTCATTAAATTGCTTTTAATTCCA*AAAAAAAAAAAAAAAAAAAAAAGCAATCATTAAGCTTTTCATC	UTR/UTR
ENSG00000274611	ENSE00003785912	ENSE00003731868	AACATGTATTTAACCAAGTTCCAACAAAAGAATGCTGCCACAG*GATGGACGTGGTAGAGGTCGCGGGCAGTTGGTGGGCACAAGAG	CDS(complete)/UTR
ENSG00000153107	ENSE00002531730	ENSE00002441208	CATCCACAGCAGTGTTTCTCGGGTTGGAAAAGCCATTGATAAG*GATTCACTTTAAGAGATTTGGAAACTCTTCCCTTTGGAATTGC	out-of-frame
ENSG09000001012			CTGTGAGTTGAATGTTGTGTTCATAGTATCTGCCAAAACAGAAAGAAAAA*AAAACAAAATATTTTGATAAGAAGTTAAAGCTTTGTATATAATATACCTT	intronic/---
ENSG00000258410			TGATAAGATGACTTGAGATGAGATGAAATGATGAGATGAAATGACAAAAT*GATGAGATGATGAGATGAAATTTTGAGATGAAATGGTGAGTAGAAATGAT	---/intronic
ENSG00000258410			AAACCCGAGTCAGTACCAAGAAGAGTCAATTTATTAGTTTTCTAAAATAA*AAAAAAAATCAAAATCACCAAAAAAGAGCAATATCCAAGAAAACATTGAA	---/intronic
ENSG00000258410			AAATGAAATAATGAAAGCAAATTATGAAATGTAATGAAATTGAAATGAAA*TTGAAATGATGAGATGTAATGATGAAATGAAATGATGAAATGAGATGAGA	---/intronic
ENSG00000258410			TAAATGAGATGATGAAATTAAATGATGAAATGAAATAATGAAATGGAAAT*GATGAGATGAAATGATGAGATGAATGATGAGATGAAATGAGATGAAATGA	---/intronic
ENSG00000163209			CCTTCACCAGATCCCAGAGGCTGAACACCTCGACCTTCTCTGCACAGCAG*GTCCAGCATCCTTTGAAGCATGAGTTCTTACCAGCAGAAGCAGACCTTTA	intergenic/UTR
ENSG00000185873			ATCGTACCACTGCACTCCAGCCTGGGCAACAGAGTGAGACCCTGTCTCAA*AAAAAAAAAAAAAAAAAAAGGAGAGAAGAAAATAAAGAAGGCTAATATAT	UTR/UTR

ENSG00000183347			CTCTGTCACCCAGGCTGGAGTGCAGTGGTGCAATCTCGGCTCGCTGCAAC*CTCTGCCTCCTGGGTTCAAGAGATTCACCTGCCTCAGCCCCCTAGTAGCT	intergenic/UTR
ENSG00000132406			ATGGAATGAAGCTGGCCTCCCGTCTCCTTGATTTGAAGCAGCTTCTCCAC*CTTCAACATTGCTTTATGGCATGTGTGGTCGTTTTTCACTCCATTGTTGT	---/CDS(truncated)
ENSG00000197822	ENSE00002214653	ENSE00003560198	TCCCTGGAATCTCTTGAAGTCTCAGGGACAATCCAGTCACAAG*GTTAAAAATGTGTCTGCAGGCACACAGGACGTGCCTTCACCCC	out-of-frame
ENSG00000197822	ENSE00003590701	ENSE00003560198	TTATTGATCCAAATTTCAGCTGAGTATGATCCTTCCAAACTAG*GTTAAAAATGTGTCTGCAGGCACACAGGACGTGCCTTCACCCC	out-of-frame

Supplementary Table 5c: all fusion genes identified in the disease tissue samples (EAC, HGD and NDB). Fusion gene pairs indicated in red are likely false positives, based on fusion description (see Supplementary Table 5a) or a high number of common mapping reads (>10).

sample ID	gene 1 symbol (5' end fusion partner)	gene 2 symbol (3' end fusion partner)	fusion_description	counts_of_common_mapping_reads	spanning_pairs	spanning_unique_reads	longest_anchor_found	fusion_finding_method	fusion_point_for_gene_1 (5' end_fusion_partner)	fusion_point_for_gene_2 (3' end_fusion_partner)
ID20_EAC	SPRR3	ANXA1	gtex,cancer,exon-exon	0	8	3	22	BOWTIE	1:153003855:+	9:73157654:+
	SPRR3	ANXA1	gtex,cancer,exon-exon	0	8	3	21	BOWTIE	1:153003856:+	9:73157654:+
	VCL	ADK	known,adjacent,cell_lines,chimerdb3seq,cancer,10K<gap<100K,readthrough,exon-exon	0	6	5	30	BOWTIE	10:73998375:+	10:74394141:+
	MTIF2	AL592494.2	no_protein,pseudogene,m2	10	5	2	32	BOWTIE+STAR	2:55240119:-	1:121503477:+
	NPEPPS	TBC1D3	banned,known,oncogene,bodymap2,hpa,gtex,18cancers,tumor,m0,multi,exon-exon	0	5	9	30	BOWTIE	17:47592545:+	17:38191030:-
	RMND5A	ANAPC1	banned,known,bodymap2,hpa,m0,multi,exon-exon	0	4	3	26	BOWTIE	2:86741069:+	2:111822600:-
	SMG1	NPIPB5	banned,known,bodymap2,hpa,m0,multi,exon-exon	0	4	4	22	BOWTIE	16:18858170:-	16:22513522:+
	SMG1	NPIPB5	banned,known,bodymap2,hpa,m0,multi	0	4	2	35	BOWTIE+STAR	16:18858211:-	16:22513522:+
	KANSL1	ARL17A	banned,known,healthy,bodymap2,hpa,gtex,18cancers,chimerdb3seq,m1,multi,exon-exon	0	3	3	26	BOWTIE	17:46094560:-	17:46570869:-
	KANSL1	ARL17B	banned,known,healthy,bodymap2,hpa,chimerdb3seq,m0,multi,10K<gap<100K,exon-exon	0	3	3	26	BOWTIE	17:46094560:-	17:46352930:-
	SPINK5	SPRR3	gtex	0	3	4	27	BOWTIE+STAR	5:148137336:+	1:153003002:+
	GSN	ANXA1	cancer,exon-exon	0	2	3	20	BOWTIE	9:121248323:+	9:73158522:+
	NAIP	OCLN	banned,known,bodymap2,hpa,1000genomes,m0,multi,exon-exon	0	2	4	30	BOWTIE	5:70974129:-	5:69534694:+
ID29_EAC	NPEPPS	TBC1D3	banned,known,oncogene,bodymap2,hpa,gtex,18cancers,tumor,m0,multi,exon-exon	0	15	15	29	BOWTIE	17:47592545:+	17:38191030:-
	EIF3F	AL356585.1	no_protein,pseudogene,cancer,m3	56	12	2	35	BOWTIE+STAR	11:7992129:+	13:18207169:-
	KRT13	ANXA1	tcga,gtex,non_cancer_tissues,cancer,m8,exon-exon	0	10	2	19	BOWTIE	17:41500982:-	9:73157654:+
	KRT13	ANXA1	tcga,gtex,non_cancer_tissues,cancer,m8,exon-exon	0	10	2	18	BOWTIE	17:41500981:-	9:73157654:+
	ATXN1L	AL136982.5	no_protein,pseudogene,m2	6	5	4	27	BOWTIE+STAR	16:71849747:+	10:87027189:+
	SPRR3	ANXA1	gtex,cancer,exon-exon	0	4	2	22	BOWTIE	1:153003855:+	9:73157654:+
	IGK@	AC087386.1	no_protein,lincrna	0	3	6	38	BOWTIE+STAR	2:90255789:+	15:20138256:-
	KRT13	PKM	gtex,cancer	0	3	4	23	BOWTIE+STAR	17:41500991:-	15:72205126:-
	NAIP	OCLN	banned,known,bodymap2,hpa,1000genomes,m0,multi,exon-exon	0	3	2	30	BOWTIE	5:70979869:-	5:69534694:+
	NAIP	OCLN	banned,known,bodymap2,hpa,1000genomes,m0,multi,exon-exon	0	3	2	29	BOWTIE	5:70983775:-	5:69534694:+
	RMND5A	ANAPC1	banned,known,bodymap2,hpa,m0,multi,exon-exon	0	3	7	29	BOWTIE	2:86741069:+	2:111822600:-
	SH3RF2	JMJD7-PLA2G4B	m2	1	3	3	31	BOWTIE+STAR	5:146080960:+	15:41837750:+
	MKRN1	ANXA1	cancer,exon-exon	0	2	2	18	BOWTIE	7:140474381:-	9:73158522:+
	KANSL1	ARL17A	banned,known,healthy,bodymap2,hpa,gtex,18cancers,chimerdb3seq,m0,multi,exon-exon	0	1	5	29	BOWTIE	17:46094560:-	17:46570869:-
	KANSL1	ARL17B	banned,known,healthy,bodymap2,hpa,chimerdb3seq,m0,multi,10K<gap<100K,exon-exon	0	1	4	29	BOWTIE	17:46094560:-	17:46352930:-
	POLA2	CDC42EP2	banned,known,adjacent,healthy,bodymap2,hpa,1000genomes,chimerdb3pub,1K<gap<10K,readthrough,exon-exon	0	1	2	25	BOWTIE	11:65295990:+	11:65320544:+
ID30_EAC	NPEPPS	TBC1D3	banned,known,oncogene,bodymap2,hpa,gtex,18cancers,tumor,m0,multi,exon-exon	0	6	9	29	BOWTIE	17:47592545:+	17:38191030:-
	LRPPRC	ACTB	cancer,m2	0	3	10	28	BOWTIE+STAR	2:43904701:-	7:5527638:-
	SMG1	NPIPB5	banned,known,bodymap2,hpa,m0,multi	0	3	2	35	BOWTIE+BOWTIE2	16:18908242:-	16:22507338:+
	SOD2	AHNAK		0	3	4	32	BOWTIE+STAR	6:159680959:-	11:62468372:-
	AC027117.2	PSD3	no_protein,antisense,exon-exon	0	2	2	30	BOWTIE	8:17801742:+	8:18556352:-
	ANXA2	KRT78	exon-exon	0	2	5	27	BOWTIE	15:60394574:-	12:52839984:-
	BPTF	LRRC37A2	banned,known,hpa,m0,multi,exon-exon	0	1	3	28	BOWTIE	17:67826337:+	17:46517362:+
	RMND5A	ANAPC1	banned,known,bodymap2,hpa,m0,multi,exon-exon	0	1	7	29	BOWTIE	2:86741069:+	2:111822600:-
ID43_EAC	*excluded from fusion gene analysis (see 'Methods' section)*									
ID2_HGD	AC020656.1	FRY	no_protein,antisense,m2	0	12	4	36	BOWTIE+STAR	12:69353766:-	13:32263749:+
	AC020656.1	FRY	no_protein,antisense,m2,exon-exon	0	12	4	22	BOWTIE	12:69353743:-	13:32275142:+
	NPEPPS	TBC1D3	banned,known,oncogene,bodymap2,hpa,gtex,18cancers,tumor,m0,multi,exon-exon	0	9	8	30	BOWTIE	17:47592545:+	17:38191030:-
	AC020656.1	ZNF7	no_protein,antisense,m2	0	7	2	37	BOWTIE+STAR	12:69353721:-	8:144838728:+
	KANSL1	ARL17A	banned,known,healthy,bodymap2,hpa,gtex,18cancers,chimerdb3seq,m0,multi,exon-exon	0	5	2	18	BOWTIE	17:46094560:-	17:46570869:-
	KANSL1	ARL17B	banned,known,healthy,bodymap2,hpa,chimerdb3seq,m0,multi,10K<gap<100K,exon-exon	0	5	2	18	BOWTIE	17:46094560:-	17:46352930:-
	LYZ	TRA@	m2	0	5	25	31	BOWTIE+STAR	12:69353497:+	14:21731216:+
	LYZ	TRA@	m2	0	5	2	32	BOWTIE+STAR	12:69353657:+	14:21550403:+
	LYZ	TRA@	m2	0	5	2	24	BOWTIE+STAR	12:69353659:+	14:21715808:+
	LYZ	TRA@	m2	0	5	2	19	BOWTIE+STAR	12:69353500:+	14:21778986:+
	AC068647.2	AADAC	no_protein,adjacent,pseudogene,1K<gap<10K,readthrough	0	4	3	37	BOWTIE+BOWTIE2;BOWTI	3:151803925:+	3:151814139:+

	NDUFB5	TRIM72	m2	3	4	3	38	BOWTIE+STAR	3:179627288:+	16:31230170:+
	KRT8	AGR2	tumor	0	3	2	20	BOWTIE+STAR	12:52897241:-	7:16830427:-
	SARNP	AC073063.1	no_protein,pseudogene,cancer	41	3	3	34	BOWTIE+STAR	12:55800593:-	7:99443293:-
	NAIP	OCLN	banned,known,bodymap2,hpa,1000genomes,m0,multi,exon-exon	0	2	2	28	BOWTIE	5:70979869:-	5:69534694:+
	SMG1	NPIPB5	banned,known,bodymap2,hpa,m0,multi,exon-exon	0	2	2	30	BOWTIE	16:18858170:-	16:22513522:+
	BPTF	LRRC37A2	banned,known,hpa,m0,multi,exon-exon	0	1	2	29	BOWTIE	17:67826337:+	17:46517362:+
	LYZ	TRA@	m7	0	19	2	35	BOWTIE+STAR	12:69353586:+	14:21642435:+
	LYZ	TRA@	m7	0	19	2	26	BOWTIE+STAR	12:69353658:+	14:21715808:+
	TRA@	LYZ	m7	0	19	2	29	BOWTIE+STAR	14:22015358:+	12:69353745:+
	TRA@	AC020656.1	no_protein,antisense,m7	0	13	2	26	BOWTIE+STAR	14:21715810:-	12:69353658:-
	LYZ	SLC2A4	m7	0	12	68	38	BOWTIE+BOWTIE2;BOWTI	12:69353497:+	17:7287133:+
	CCND3	CEACAM5	cancer,m5	5	9	3	33	BOWTIE+STAR	6:41992465:-	19:41729500:+
	CCND3	CEACAM5	cancer,m5	5	9	2	36	BOWTIE+BOWTIE2	6:41993419:-	19:41729495:+
**ID5_HGD**	CCND3	CEACAM5	cancer,m5	5	9	2	23	BOWTIE+STAR	6:41948333:-	19:41729500:+
	CCND3	CEACAM5	cancer,m5	5	9	2	20	BOWTIE+STAR	6:42036939:-	19:41729500:+
	MOG	DDOST	m6	5	7	4	23	BOWTIE+STAR	6:29672337:+	1:20651804:-
	MOG	DDOST	m6	5	7	3	22	BOWTIE+STAR	6:29672295:+	1:20651838:-
	TMEM231	AC020656.1	no_protein,antisense,m2	2	7	2	33	BOWTIE+STAR	16:75537728:-	12:69353729:-
	AC239800.2	AL137802.1	no_protein,lincrna,m3,exon-exon	35	6	4	29	BOWTIE	1:143736123:+	1:16521564:+
	LYZ	ANXA2		0	5	2	29	BOWTIE+STAR	12:69353497:+	15:60363051:-
	CD24	TSPAN8	oncogene,cancer	0	4	6	22	BOWTIE+STAR	6:106969972:-	12:71237157:-
	LYZ	SLC2A4	m26	0	55	48	38	BOWTIE+BOWTIE2;BOWTI	12:69353494:+	17:7287133:+
	AC020656.1	FRY	no_protein,antisense,m2,exon-exon	0	15	6	25	BOWTIE	12:69353743:-	13:32275142:+
	AC020656.1	ZNF7	no_protein,antisense,m2	0	12	3	38	BOWTIE+STAR	12:69353721:-	8:144838728:+
	NPEPPS	TBC1D3	banned,known,oncogene,bodymap2,hpa,gtex,18cancers,tumor,m0,multi,exon-exon	0	4	5	24	BOWTIE	17:47592545:+	17:38191030:-
	PIF1	DDOST	m3	10	4	2	27	BOWTIE+STAR	15:64821914:-	1:20651996:-
	CATSPER2	PPIP5K1	banned,known,bodymap2,hpa,1000genomes,m0,multi,10K<gap<100K,exon-exon	8	3	2	28	BOWTIE	15:43664418:-	15:43564165:-
	JCHAIN	ACTB	cancer,m2	0	3	4	33	BOWTIE+STAR	4:70659551:-	7:5527639:-
	JCHAIN	ACTB	cancer,m2	0	3	2	20	BOWTIE+STAR	4:70655959:-	7:5527641:-
**ID25_HGD**	LYZ	RPS10-NUDT3	m2	0	3	43	38	BOWTIE+STAR	12:69353502:+	6:34421010:-
	LYZ	RPS10-NUDT3	m2	0	3	2	22	BOWTIE+STAR	12:69353504:+	6:34345680:-
	LYZ	RPS10-NUDT3	m2	0	3	2	18	BOWTIE+STAR	12:69353495:+	6:34407055:-
	PPIP5K1	CATSPER2	banned,known,bodymap2,hpa,1000genomes,m0,multi,10K<gap<100K,exon-exon	8	3	2	30	BOWTIE	15:43564103:-	15:43649879:-
	PPIP5K1	CATSPER2	banned,known,bodymap2,hpa,1000genomes,m0,multi,10K<gap<100K,exon-exon	8	3	2	25	BOWTIE	15:43564867:-	15:43640496:-
	PPIP5K1	CATSPER2	banned,known,bodymap2,hpa,1000genomes,m0,multi,10K<gap<100K,exon-exon	8	3	2	24	BOWTIE	15:43564867:-	15:43648063:-
	RMND5A	ANAPC1	banned,known,bodymap2,hpa,m0,multi,exon-exon	0	3	2	29	BOWTIE	2:86741069:+	2:111822600:-
	SMG1	NPIPB5	banned,known,bodymap2,hpa,m0,multi,exon-exon	0	1	4	29	BOWTIE	16:18858170:-	16:22513522:+
	LYZ	SLC2A4	m25	0	47	108	38	BOWTIE+STAR	12:69353497:+	17:7287135:+
	LYZ	TRA@	m5	0	21	3	29	BOWTIE+STAR	12:69353658:+	14:21753608:+
	AC020656.1	ZNF7	no_protein,antisense,m3	0	19	2	33	BOWTIE+STAR	12:69353721:-	8:144838728:+
	ZNF7	AC020656.1	no_protein,antisense,m3	0	19	2	33	BOWTIE+STAR	8:144838787:+	12:69353661:-
	LYZ	TRA@	m3	0	15	2	38	BOWTIE+STAR	12:69353657:+	14:21804444:-
	KCNE3	ALKBH4	m5	19	12	2	19	BOWTIE+STAR	11:74456348:-	7:102460337:-
	TRA@	AC020656.1	no_protein,antisense,m5	0	12	2	18	BOWTIE+STAR	14:21788391:-	12:69354225:-
	TRA@	AC020656.1	no_protein,antisense,m2	0	10	2	34	BOWTIE+STAR	14:21804440:+	12:69353661:-
**ID26_HGD**	AC020656.1	YIPF5	no_protein,antisense,m2	0	9	3	31	BOWTIE+STAR	12:69353737:-	5:144159956:-
	RNF41	AC020656.1	no_protein,antisense,m2	0	8	2	34	BOWTIE+STAR	12:56209624:-	12:69353654:-
	COQ8B	NUMBL	banned,known,adjacent,healthy,bodymap2,hpa,gap<1K,readthrough	0	5	2	21	BOWTIE+STAR	19:40691592:-	19:40686995:-
	SARNP	AC073063.1	no_protein,pseudogene,cancer,m2	32	4	2	31	BOWTIE+STAR	12:55800593:-	7:99443293:-
	IGK@	GPRC5A	tumor,m2	0	3	2	23	BOWTIE+STAR	2:89989628:+	12:12917260:+
	LYZ	CALR	m2	4	3	10	37	BOWTIE+STAR	12:69353649:+	19:12943246:+
	LYZ	RPS24	ribosomal,m2	0	3	2	34	BOWTIE+BOWTIE2	12:69353513:+	10:78036328:+
	NPEPPS	TBC1D3	banned,known,oncogene,bodymap2,hpa,gtex,18cancers,tumor,m0,multi,exon-exon	0	3	9	29	BOWTIE	17:47592545:+	17:38191030:-
	RPS24	LYZ	ribosomal,m2	0	3	4	32	BOWTIE+STAR	10:78055958:+	12:69353696:+
	LYZ	SLC2A4	m28	0	42	10	34	BOWTIE+STAR	12:69353497:+	17:7287125:+
	PRIM1	NACA	banned,known,adjacent,conjoing,healthy,bodymap2,hpa,1000genomes,cancer,m7,gap<1K,readthrough	0	8	4	34	BOWTIE+STAR	12:56731608:-	12:56731607:-
	LYZ	TRA@	m5	0	6	2	36	BOWTIE+STAR	12:69353646:+	14:21568870:+
	SMG1	NPIPB5	banned,known,bodymap2,hpa,m0,multi,exon-exon	0	4	2	26	BOWTIE	16:18858170:-	16:22513522:+
**ID39_HGD**	EIF3E	RSPO2	known,oncogene,cosmic,cgp,chimerdb3kb,chimerdb3pub,cancer,100K<gap<200K,exon-exon	0	3	2	30	BOWTIE	8:108248613:-	8:108082807:-

[illegible]	NPEPPS	TBC1D3	banned,known,oncogene,bodymap2,hpa,gtex,18cancers,tumor,m0,multi,exon-exon	0	3	3	29	BOWTIE	17:47592545:+	17:38191030:-
	RMND5A	ANAPC1	banned,known,bodymap2,hpa,m0,multi,exon-exon	0	2	4	26	BOWTIE	2:86741069:+	2:111822600:-
	RRM2	C2ORF48	banned,known,no_protein,adjacent,lincrna,conjoing,cacg,non_tumor_cells,hpa,1000genomes,1K<gap<10K,readthrough,exon-exon	0	2	3	28	BOWTIE	2:10129154:+	2:10141854:+
	COQ8B	NUMBL	banned,known,adjacent,healthy,bodymap2,hpa,gap<1K,readthrough,exon-exon	0	1	2	30	BOWTIE	19:40692951:-	19:40686995:-
ID18_NDB	LYZ	SLC2A4	m7	0	16	86	38	BOWTIE+STAR	12:69353497:+	17:7287135:+
	AC004923.1	AC068587.6	no_protein,lincrna,m5,exon-exon	31	7	2	27	BOWTIE	11:67955482:-	8:12581044:-
	AC068587.6	AC004923.1	no_protein,lincrna,m5,exon-exon	31	7	2	27	BOWTIE	8:12659130:-	11:67936991:-
	RPL22	MECOM	banned,known,ribosomal,oncogene,chimerdb2,cgp,bodymap2,hpa,18cancers,chimerdb3kb,chimerdb3seq,cancer,tumor,m0,multi	0	4	15	37	BOWTIE+STAR	1:6186507:-	3:169483506:-
	RPL22	MECOM	banned,known,ribosomal,oncogene,chimerdb2,cgp,bodymap2,hpa,18cancers,chimerdb3kb,chimerdb3seq,cancer,tumor,m0,multi	0	4	13	29	BOWTIE+STAR	1:6197732:-	3:169484014:-
	RPL22	MECOM	banned,known,ribosomal,oncogene,chimerdb2,cgp,bodymap2,hpa,18cancers,chimerdb3kb,chimerdb3seq,cancer,tumor,m0,multi	0	4	4	22	BOWTIE+STAR	1:6186797:-	3:169483796:-
	RPL22	MECOM	banned,known,ribosomal,oncogene,chimerdb2,cgp,bodymap2,hpa,18cancers,chimerdb3kb,chimerdb3seq,cancer,tumor,m0,multi	0	4	2	24	BOWTIE+STAR	1:6197660:-	3:169483945:-
	AC009094.1	NOP58	no_protein,pseudogene	25	3	4	38	BOWTIE+STAR	16:60024605:-	2:202303410:+
	CBX3	C15ORF57	banned,known,healthy,tcga,hpa,gtex,gliomas,chimerdb3pub,chimerdb3seq,oesophagus	0	3	2	26	BOWTIE+STAR	7:26212581:+	15:40563710:-
	KRT8	EPCAM	oncogene,cancer	0	3	2	18	BOWTIE+STAR	12:52897239:-	2:47382993:+
	NOP58	AC009094.1	no_protein,pseudogene	25	3	3	37	BOWTIE+STAR	2:202300342:+	16:60024793:-
	AZGP1	GJC3	banned,known,adjacent,healthy,cacg,bodymap2,hpa,10K<gap<100K,readthrough,exon-exon	0	1	2	19	BOWTIE	7:99971746:-	7:99923603:-
	NPEPPS	TBC1D3	banned,known,oncogene,bodymap2,hpa,gtex,18cancers,tumor,m0,multi,exon-exon	0	1	2	29	BOWTIE	17:47592545:+	17:38191030:-
ID1_NDB	DNAJC25	LHX2	exon-exon	0	18	10	30	BOWTIE	9:111631743:+	9:124013961:+
	LYZ	SLC2A4	m9	0	17	6	32	BOWTIE+STAR	12:69353497:+	17:7287125:+
	DNAJC25-GNG10	LHX2	exon-exon	0	16	3	19	BOWTIE+STAR	9:111631743:+	9:124013961:+
	SMG1	NPIPB5	banned,known,bodymap2,hpa,m0,multi,exon-exon	0	12	4	29	BOWTIE	16:18858170:-	16:22513522:+
	SMG1	NPIPB5	banned,known,bodymap2,hpa,m0,multi	0	12	2	35	BOWTIE+STAR	16:18858211:-	16:22513522:+
	SMG1	NPIPB5	banned,known,bodymap2,hpa,m0,multi	0	12	2	27	BOWTIE+STAR	16:18858171:-	16:22507337:+
	NPEPPS	TBC1D3	banned,known,oncogene,bodymap2,hpa,gtex,18cancers,tumor,m0,multi,exon-exon	0	11	9	30	BOWTIE	17:47592545:+	17:38191030:-
	AC068647.2	AADAC	no_protein,adjacent,pseudogene,1K<gap<10K,readthrough	0	3	2	22	BOWTIE+STAR	3:151808240:+	3:151814139:+
	GPRC5A	IGK@	tumor,m2	0	3	2	37	BOWTIE+STAR	12:12914761:+	2:90277750:+
	KRT8	ACTB	cancer,m2	0	3	3	37	BOWTIE+BOWTIE2	12:52922185:-	7:5527623:-
	KRT8	ACTB	cancer,m2	0	3	2	21	BOWTIE+STAR	12:52932810:-	7:5527639:-
	KRT8	ACTB	cancer,m2	0	3	2	19	BOWTIE+STAR	12:52914234:-	7:5527639:-
	GOLT1A	KISS1	banned,known,adjacent,healthy,hpa,18cancers,1K<gap<10K,readthrough,exon-exon	0	2	2	29	BOWTIE	1:204213882:-	1:204192914:-
	KANSL1	ARL17A	banned,known,healthy,bodymap2,hpa,gtex,18cancers,chimerdb3seq,m0,multi,exon-exon	0	2	3	30	BOWTIE	17:46094560:-	17:46570869:-
	KANSL1	ARL17B	banned,known,healthy,bodymap2,hpa,chimerdb3seq,m0,multi,10K<gap<100K,exon-exon	0	2	3	30	BOWTIE	17:46094560:-	17:46352930:-
	NAIP	OCLN	banned,known,bodymap2,hpa,1000genomes,m0,multi,exon-exon	0	2	2	21	BOWTIE	5:70979869:-	5:69534694:+
	RMND5A	ANAPC1	banned,known,bodymap2,hpa,m0,multi,exon-exon	0	2	3	28	BOWTIE	2:86741069:+	2:111822600:-
ID19_NDB	LYZ	SLC2A4	m6	0	12	70	38	BOWTIE+STAR	12:69353497:+	17:7287133:+
	NPEPPS	TBC1D3	banned,known,oncogene,bodymap2,hpa,gtex,18cancers,tumor,m0,multi,exon-exon	0	7	10	30	BOWTIE	17:47592545:+	17:38191030:-
	KANSL1	ARL17A	banned,known,healthy,bodymap2,hpa,gtex,18cancers,chimerdb3seq,m0,multi,exon-exon	0	5	2	19	BOWTIE	17:46094560:-	17:46570869:-
	KANSL1	ARL17B	banned,known,healthy,bodymap2,hpa,chimerdb3seq,m0,multi,10K<gap<100K,exon-exon	0	5	2	19	BOWTIE	17:46094560:-	17:46352930:-
	KMT5A	SETD8P1	no_protein,pseudogene	43	4	2	37	BOWTIE+STAR	12:123395252:+	13:18298174:+
	KMT5A	SETD8P1	no_protein,pseudogene	43	4	2	35	BOWTIE+BOWTIE2	12:123389134:+	13:18297592:+
	KMT5A	SETD8P1	no_protein,pseudogene	43	4	2	24	BOWTIE+STAR	12:123389109:+	13:18297564:+
	AC068587.6	CNN3	no_protein,lincrna,m2	0	3	2	37	BOWTIE+STAR	8:12595234:-	1:94906213:-
	CDH17	CEACAM5	oncogene	0	3	2	20	BOWTIE+STAR	8:94216558:-	19:41729500:+
	KRT8	IFI6		0	3	3	28	BOWTIE+STAR	12:52922197:-	1:27666312:-
	KRT8	IFI6		0	3	2	27	BOWTIE+STAR	12:52935385:-	1:27666312:-
	PIGR	TPD52	oncogene,cancer	1	3	5	31	BOWTIE+STAR	1:206929559:-	8:80152793:-
	SMG1	NPIPB5	banned,known,bodymap2,hpa,m0,multi,exon-exon	0	3	3	30	BOWTIE	16:18858170:-	16:22513522:+
	CFL1	PDIA3	exon-exon	0	2	2	24	BOWTIE	11:65854837:-	15:43746392:+
	GOLM1	SI	exon-exon	0	2	2	30	BOWTIE	9:86084920:-	3:164987226:-
	RMND5A	ANAPC1	banned,known,bodymap2,hpa,m0,multi,exon-exon	0	1	3	29	BOWTIE	2:86741069:+	2:111822600:-

ID22_NDB	LYZ	SLC2A4	m16	0	25	17	37	BOWTIE+STAR	12:69353497:+	17:7287126:+
	CCND3	CEACAM5	cancer,m8	18	17	3	38	BOWTIE+BOWTIE2	6:42034487:-	19:41729495:+
	CCND3	CEACAM5	cancer,m8	18	17	2	35	BOWTIE+BOWTIE2	6:41992465:-	19:41729499:+
	SMG1	NPIPB5	banned,known,bodymap2,hpa,m0,multi	0	5	2	38	BOWTIE+STAR	16:18858211:-	16:22513522:+
	AL022322.2	GALNT4	no_protein,antisense,m2	19	4	4	37	BOWTIE+STAR	22:38134420:+	12:89521262:-
	PIGR	MUC13	cancer	0	3	4	30	BOWTIE+STAR	1:206929571:-	3:124944363:-
	SAR1B	ARL17B	m2	0	3	4	36	BOWTIE+STAR	5:134602888:-	17:46311147:-
	ANXA2	FCGBP	exon-exon	0	2	2	26	BOWTIE	15:60394574:-	19:39886585:-
	FAM171A1	DCLRE1C	exon-exon	0	2	4	26	BOWTIE	10:15370956:-	10:14939869:-
	KANSL1	ARL17A	banned,known,healthy,bodymap2,hpa,gtex,18cancers,chimerdb3seq,m0,multi,exon-exon	0	2	5	28	BOWTIE	17:46094560:-	17:46570869:-
	KANSL1	ARL17B	banned,known,healthy,bodymap2,hpa,chimerdb3seq,m0,multi,10K<gap<100K,exon-exon	0	2	5	28	BOWTIE	17:46094560:-	17:46352930:-
	NPEPPS	TBC1D3	banned,known,oncogene,bodymap2,hpa,gtex,18cancers,tumor,m0,multi,exon-exon	0	2	12	30	BOWTIE	17:47592545:+	17:38191030:-
	SCNN1A	TNFRSF1A	banned,known,adjacent,conjoing,healthy,bodymap2,non_tumor_cells,hpa,cancer,1K<gap<10K,readthrough,exon-exon	0	2	2	22	BOWTIE	12:6348727:-	12:6334244:-
	PPIP5K1	CATSPER2	banned,known,bodymap2,ambiguous,hpa,1000genomes,m0,multi,10K<gap<100K,exon-exon	22	1	2	27	BOWTIE	15:43564867:-	15:43648063:-
	RMND5A	ANAPC1	banned,known,bodymap2,hpa,m0,multi,exon-exon	0	1	2	26	BOWTIE	2:86741069:+	2:111822600:-
ID33_NDB	LYZ	SLC2A4	m43	1	72	12	35	BOWTIE+STAR	12:69353497:+	17:7287125:+
	LYZ	TRA@	m2	0	8	2	24	BOWTIE+STAR	12:69353502:+	14:21719847:+
	LYZ	TRA@	m2	0	8	2	20	BOWTIE+STAR	12:69353506:+	14:21660632:+
	TRA@	LYZ	m2	0	8	2	35	BOWTIE+STAR	14:21575298:+	12:69353708:+
	PRIM1	NACA	banned,known,adjacent,conjoing,healthy,bodymap2,hpa,1000genomes,cancer,gap<1K,readthrough	0	5	4	32	BOWTIE+STAR	12:56731608:-	12:56731607:-
	SMG1	NPIPB5	banned,known,bodymap2,hpa,m0,multi,exon-exon	0	5	2	23	BOWTIE	16:18858170:-	16:22513522:+
	AC020656.1	NUPR1	no_protein,antisense	0	4	2	34	BOWTIE+STAR	12:69353766:-	16:28533648:-
	NAIP	OCLN	banned,known,bodymap2,hpa,1000genomes,m0,multi,exon-exon	0	4	3	23	BOWTIE	5:70979869:-	5:69534694:+
	NPEPPS	TBC1D3	banned,known,oncogene,bodymap2,hpa,gtex,18cancers,tumor,m0,multi,exon-exon	0	3	8	30	BOWTIE	17:47592545:+	17:38191030:-
	RMND5A	ANAPC1	banned,known,bodymap2,hpa,m0,multi,exon-exon	0	2	2	26	BOWTIE	2:86741069:+	2:111822600:-
	NSF	LRRC37A3	banned,known,hpa,m0,multi,exon-exon	0	1	3	26	BOWTIE	17:46704854:+	17:64892600:-
ID35_NDB	LYZ	SLC2A4	m21	0	36	7	33	BOWTIE+STAR	12:69353497:+	17:7287125:+
	KIF13B	EXTL3-AS1	no_protein,antisense	0	9	5	33	BOWTIE+BOWTIE2;BOWTI	8:29070319:-	8:28700043:-
	KIF13B	EXTL3-AS1	no_protein,antisense	0	9	3	36	BOWTIE+STAR	8:29068800:-	8:28700525:-
	RMND5A	ANAPC1	banned,known,bodymap2,hpa,m0,multi,exon-exon	0	9	7	30	BOWTIE;BOWTIE+STAR	2:86741069:+	2:111822600:-
	LINC01972	AC020656.1	no_protein,lincrna,antisense,m5	0	7	2	30	BOWTIE+STAR	3:194765471:-	12:69353707:-
	AC074051.2	AC068587.6	no_protein,lincrna,m3,exon-exon	35	4	3	29	BOWTIE	16:5240105:+	8:12595296:-
	NPEPPS	TBC1D3	banned,known,oncogene,bodymap2,hpa,gtex,18cancers,tumor,m0,multi,exon-exon	0	4	8	30	BOWTIE	17:47592545:+	17:38191030:-
	SMG1	NPIPB5	banned,known,bodymap2,hpa,m1,multi	0	4	3	38	BOWTIE+STAR	16:18858211:-	16:22513522:+
	SMG1	NPIPB5	banned,known,bodymap2,hpa,m1,multi,exon-exon	0	4	2	29	BOWTIE	16:18858170:-	16:22513522:+
	ZNF160	ZNF468	m3	4	4	2	32	BOWTIE+STAR	19:53086054:-	19:52854052:-
	ZNF468	ZNF160	m3	4	4	3	28	BOWTIE+STAR	19:52854084:-	19:53086085:-
	GOLT1A	KISS1	banned,known,adjacent,healthy,hpa,18cancers,1K<gap<10K,readthrough,exon-exon	0	1	2	22	BOWTIE	1:204213882:-	1:204192914:-
	KANSL1	ARL17A	banned,known,healthy,bodymap2,hpa,gtex,18cancers,chimerdb3seq,m0,multi,exon-exon	0	1	5	27	BOWTIE	17:46094560:-	17:46570869:-
	KANSL1	ARL17B	banned,known,healthy,bodymap2,hpa,chimerdb3seq,m0,multi,10K<gap<100K,exon-exon	0	1	5	27	BOWTIE	17:46094560:-	17:46352930:-
	KANSL1	LRRC37A3	banned,known,hpa,non_cancer_tissues,m0,multi,exon-exon	0	1	2	27	BOWTIE	17:46152904:-	17:64869166:-
	NAIP	OCLN	banned,known,bodymap2,hpa,1000genomes,m0,multi,exon-exon	0	1	2	30	BOWTIE	5:70979869:-	5:69534694:+
ID37_NDB	LYZ	SLC2A4	m35	0	59	93	38	BOWTIE+BOWTIE2;BOWTI	12:69353497:+	17:7287133:+
	LYZ	TRA@	m7	0	18	8	37	BOWTIE+STAR	12:69353656:+	14:21575249:+
	AC020656.1	TRA@	no_protein,antisense,m7	0	12	2	36	BOWTIE+STAR	12:69353766:-	14:21559346:-
	TRA@	AC020656.1	no_protein,antisense,m7	0	12	8	37	BOWTIE+STAR	14:21575254:-	12:69353661:-
	AC020656.1	ZNF7	no_protein,antisense,m3	0	10	7	37	BOWTIE+STAR	12:69353721:-	8:144838728:+
	LYZ	MALT1	oncogene,cancer,m3	41	10	2	37	BOWTIE+STAR	12:69353723:+	18:58684682:+
	NPEPPS	TBC1D3	banned,known,oncogene,bodymap2,hpa,gtex,18cancers,tumor,m0,multi,exon-exon	0	7	9	30	BOWTIE	17:47592545:+	17:38191030:-
	KANSL1	ARL17A	banned,known,healthy,bodymap2,hpa,gtex,18cancers,chimerdb3seq,m0,multi,exon-exon	0	3	2	26	BOWTIE	17:46094560:-	17:46570869:-
	KANSL1	ARL17B	banned,known,healthy,bodymap2,hpa,chimerdb3seq,m0,multi,10K<gap<100K,exon-exon	0	3	2	26	BOWTIE	17:46094560:-	17:46352930:-
	SARNP	AC073063.1	no_protein,pseudogene,cancer	23	3	3	30	BOWTIE+STAR	12:55800593:-	7:99443293:-
	WDR70	AL021368.5	no_protein,lincrna,exon-exon	7	3	2	26	BOWTIE	5:37379392:+	6:57961224:-

	AC025279.1	BANP	no_protein,lincrna,oncogene,tumor,exon-exon	7	2	2	21	BOWTIE	16:29326483:+	16:88035323:+
	DUS4L	BCAP29	banned,known,adjacent,healthy,bodymap2,hpa,chimerdb3pub,1K<gap<10K,readthrough,exon-exon	0	2	2	19	BOWTIE	7:107576592:+	7:107580759:+
	NAIP	OCLN	banned,known,bodymap2,hpa,1000genomes,m0,multi,exon-exon	0	2	6	24	BOWTIE	5:70979869:-	5:69534694:+
**ID40_NDB**	*excluded from fusion gene analysis (see 'Methods' section)*									

gene_1_id(5' end_fusion_partner)	gene_2_id(3' end_fusion_partner)	exon_1_id(5' end_fusion_partner)	exon_2_id(3' end_fusion_partner)	fusion_sequence	predicted_effect
ENSG00000163209	ENSG00000135046	ENSE00001548454	ENSE00001472097	TGCATGTTTCCTGCTCTTCCCTCATTAAATTGCTTTTAATTCC*AAAAAAAAAAAAAAAAAAAAAAAGCAATCATTAAGCTTTTCATC	UTR/UTR
ENSG00000163209	ENSG00000135046	ENSE00001901937	ENSE00001472097	GCATGTTTCCTGCTCTTCCCTCATTAAATTGCTTTTAATTCCA*AAAAAAAAAAAAAAAAAAAAAAAGCAATCATTAAGCTTTTCATC	UTR/UTR
ENSG00000035403	ENSG00000156110	ENSE00002288595	ENSE00001024568	GCCCGTGGCCGCCGTGCAGGCGGCCGTCAGCAACCTCGTCCGG*TGGATGATTCAACAGCCACACAAAGCAGCAACATTTTTTGGAT	in-frame
ENSG00000085760	ENSG00000233432			ATATGGCTTTAATGTGAATGCAGGCAATGTTATCCAACAGTCAGCTGCAA*AAAAAAGGAGTAAAAATTAAACTTCACAAAATAATTTACCGTCTTGCTGG	CDS(truncated)/exonic(no-known-CDS)
ENSG00000141279	ENSG00000274611	ENSE00003785912	ENSE00003731868	AACATGTATTTAACCAAGTTCCAACAAAAGAATGCTGCCACAG*GATGGACGTGGTAGAGGTCGCGGGCAGTTGGTGGGCACAAGAG	CDS(complete)/UTR
ENSG00000153561	ENSG00000153107	ENSE00002531730	ENSE00002441208	CATCCACAGCAGTGTTTCTCGGGTTGGAAAAGCCATTGATAAG*GATTCACTTTAAGAGATTTGGAAACTCTTCCCTTTGGAATTGC	out-of-frame
ENSG00000157106	ENSG00000243716	ENSE00001505919	ENSE00002138288	AGGTATACTATGTACCAGAATCAGTTGTTGGAGAAAATTAAAG*GTATGATCTCGTGAAATCTTGAGAGAAACTGAATGACGAATGA	CDS(truncated)/UTR
ENSG00000157106	ENSG00000243716			TTACGTTCATGTAAACAGCATGACGTGAGGCCATGGATGCAGGCATTAAG*GTATGATCTCGTGAAATCTTGAGAGAAACTGAATGACGAATGAAACTATT	CDS(truncated)/UTR
ENSG00000120071	ENSG00000185829	ENSE00003662350	ENSE00003806456	TCGTCAGCAAACAGACATTTACAAACAGATACGTGCTAATAAG*GTTTCTGTGTGGAGACAGTAGAATATAAAAATAACACCTTCGC	out-of-frame
ENSG00000120071	ENSG00000228696	ENSE00003662350	ENSE00002370389	TCGTCAGCAAACAGACATTTACAAACAGATACGTGCTAATAAG*GTTTCTGTGTGGAGACAGTAGAATATAAAAATAACACCTTCGC	out-of-frame
ENSG00000133710	ENSG00000163209			CCTTCACCAGATCCCAGAGGCTGAACACCTCGACCTTCTCTGCACAGCAG*GTCCAGCATCCTTTGAAGCATGAGTTCTTACCAGCAGAAGCAGACCTTTA	intergenic/UTR
ENSG00000148180	ENSG00000135046	ENSE00001518181	ENSE00003569681	AGTGATTCTCCTGCCTCAGCCTCCTGAGTAGCTGGGATTACAG*ACACTTTTTCAAAAATGGCAATGGTATCAGAATTCCTCAAGCA	UTR/CDS(no-known-start-or-end)
ENSG00000249437	ENSG00000197822	ENSE00003606270	ENSE00003560198	TCATTTTTCAAGACTTTGAATGATGACAGCGTGGTGGAAATTG*GTTAAAAATGTGTCTGCAGGCACACAGGACGTGCCTTCACCCC	in-frame
ENSG00000141279	ENSG00000274611	ENSE00003785912	ENSE00003731868	AACATGTATTTAACCAAGTTCCAACAAAAGAATGCTGCCACAG*GATGGACGTGGTAGAGGTCGCGGGCAGTTGGTGGGCACAAGAG	CDS(complete)/UTR
ENSG00000175390	ENSG00000279081			TTAGGTGGCTGTTGACATGGAATTTGCTAAGAATATGTATGAACTGCATA*AAAAAAGTTTCTCCAAATGAGCTCATCTTGGGGTGGTACGCTGCCGGCCA	CDS(truncated)/exonic(no-known-CDS)
ENSG00000171401	ENSG00000135046	ENSE00001841099	ENSE00001472097	AGAAAAGATTATTCAATAAAGTTTCCTGCCTTTCTGCAAACAT*AAAAAAAAAAAAAAAAAAAAAAAGCAATCATTAAGCTTTTCATC	UTR/UTR
ENSG00000171401	ENSG00000135046	ENSE00003650505	ENSE00001472097	GAAAAGATTATTCAATAAAGTTTCCTGCCTTTCTGCAAACATA*AAAAAAAAAAAAAAAAAAAAAAAGCAATCATTAAGCTTTTCATC	UTR/UTR
ENSG00000224470	ENSG00000271573			GCAAGTCGACTCCTTCCAGGCTCCAGGAACACCACAAAGCAATATGAAAC*CTGTTCATGAGAGGAGTCAGGAATGCCTTCCACCAAAGAAACGAGACCTC	CDS(truncated)/exonic(no-known-CDS)
ENSG00000163209	ENSG00000135046	ENSE00001548454	ENSE00001472097	TGCATGTTTCCTGCTCTTCCCTCATTAAATTGCTTTTAATTCC*AAAAAAAAAAAAAAAAAAAAAAAGCAATCATTAAGCTTTTCATC	UTR/UTR
ENSG09000001012	ENSG00000258410			TGATAAGATGACTTGAGATGAGATGAAATGATGAGATGAAATGACAAAAT*GATGAGATGATGAGATGAAATTTTGAGATGAAATGGTGAGTAGAAATGAT	---/intronic
ENSG00000171401	ENSG00000067225			CTTCTCTACCCCAAAGAAAAGATTATTCAATAAAGTTTCCTGCCTTTC*CAAACATAAAAAAAAAAACAAAAAAATCGGTAGAGAGTGATTTCTCTCCC	UTR/intronic
ENSG00000249437	ENSG00000197822	ENSE00003590701	ENSE00003560198	TTATTGATCCAAATTTCAGCTGAGTATGATCCTTCCAAACTAG*GTTAAAAATGTGTCTGCAGGCACACAGGACGTGCCTTCACCCC	out-of-frame
ENSG00000249437	ENSG00000197822	ENSE00002214653	ENSE00003560198	TCCCTGGAATCTCTTGAAGTCTCAGGGACAATCCAGTCACAAG*GTTAAAAATGTGTCTGCAGGCACACAGGACGTGCCTTCACCCC	out-of-frame
ENSG00000153561	ENSG00000153107	ENSE00002531730	ENSE00002441208	CATCCACAGCAGTGTTTCTCGGGTTGGAAAAGCCATTGATAAG*GATTCACTTTAAGAGATTTGGAAACTCTTCCCTTTGGAATTGC	out-of-frame
ENSG00000156463	ENSG00000168970			GCCTCAGCCTCCCGAGTAGCTGGGACTACAGGTGCGTGCCACCTTGCCCT*GCTAATTTTTTTGTATTTTTAGTAGAGACGGGGTTTCACCGTGTTAGCCA	UTR/UTR
ENSG00000133606	ENSG00000135046	ENSE00003470911	ENSE00003569681	TCAAGCAATTCTGCCTCAGCCTCCCAAGTAGCTGGGACTACAG*ACACTTTTTCAAAAATGGCAATGGTATCAGAATTCCTCAAGCA	UTR/CDS(no-known-start-or-end)
ENSG00000120071	ENSG00000185829	ENSE00003662350	ENSE00003806456	TCGTCAGCAAACAGACATTTACAAACAGATACGTGCTAATAAG*GTTTCTGTGTGGAGACAGTAGAATATAAAAATAACACCTTCGC	out-of-frame
ENSG00000120071	ENSG00000228696	ENSE00003662350	ENSE00002370389	TCGTCAGCAAACAGACATTTACAAACAGATACGTGCTAATAAG*GTTTCTGTGTGGAGACAGTAGAATATAAAAATAACACCTTCGC	out-of-frame
ENSG00000014138	ENSG00000149798	ENSE00003465841	ENSE00000992560	AGATGTCCTCATCATCCCGTCAGAGCTGAGGTACTTCGTGAAG*CTCCTCAGCCCTGGACCGGGGACAAGTAACCCTCGGTGACAAG	CDS(truncated)/UTR
ENSG00000141279	ENSG00000274611	ENSE00003785912	ENSE00003731868	AACATGTATTTAACCAAGTTCCAACAAAAGAATGCTGCCACAG*GATGGACGTGGTAGAGGTCGCGGGCAGTTGGTGGGCACAAGAG	CDS(complete)/UTR
ENSG00000138095	ENSG00000075624			GGTTTTGTTTTGGGTTTTTTTTTGTTTTGTTTTTTTTTTTTGTTTTTG*TTTTTTTTTTTTTTTGGCTTGACTCAGGATTTAAAAACTGGAACGGTGA	intronic/UTR
ENSG00000157106	ENSG00000243716			TGGGTAAGCGATTCTCCTGCCTCAGCCTCCCGAGTAGCTGGGATTACAGA*GCTTGAAATGAATTTTAAAGGATGACTGATGGTCCTTGGAAGAGAAACAG	intronic/UTR
ENSG00000112096	ENSG00000124942			CTGTGTACATAGGTTTATCTCCTATTGGAATATTTTCTTTATATAGGCG*TTTTTTTTTTTTTTCTTTTTTTTGGGAGACAGAGTCTCGCTTTGTCACCC	UTR/intronic
ENSG00000253944	ENSG00000156011	ENSE00002111832	ENSE00003584316	GGGGCTCCGCCGCACCCAGCGCTAGGGCCCAGGCCGGGCCGCG*GAGGAGCAACTGAAGTCACATGAAAGTAAGCTGAAGCAGATCA	exonic(no-known-CDS)/CDS(truncated)
ENSG00000182718	ENSG00000170423	ENSE00002555095	ENSE00001659005	AGTGATTCTCCAGCCTCACCCTCCCGAGTAGCTGGGATTACAG*AACGCCAGCCTGCAGGCCGCCATCACTGATGCTGAGCAGCGTG	UTR/CDS(truncated)
ENSG00000171634	ENSG00000238083	ENSE00002689718	ENSE00002373231	GAAAGCAGCTTCAGGAGCCATAGTACCTACAGCAGCACTCCAG*AAATTTCCAAGGAAACTATATTTCTTACATTGATGGAAATGTA	out-of-frame
ENSG00000153561	ENSG00000153107	ENSE00002531730	ENSE00002441208	CATCCACAGCAGTGTTTCTCGGGTTGGAAAAGCCATTGATAAG*GATTCACTTTAAGAGATTTGGAAACTCTTCCCTTTGGAATTGC	out-of-frame
ENSG00000257764	ENSG00000073910			GGTTATTTCTTTGATAAGAACTGAATGTGGCCGGGCGCAGTGGCTCACGC*CTGTAATCCCAGCACTTCGGGAGGCCGAGGCGGGTGGATCACGAGGTCAG	exonic(no-known-CDS)/intronic
ENSG00000257764	ENSG00000073910	ENSE00002336888	ENSE00003270899	CCGGGCGCAGTGGCTCACGCCTGTAATCCCAGCACTTTGGGAG*GCCGAGGTGGGCGGATCACAAGGTCAGGAGATCGAGACCATCC	exonic(no-known-CDS)/UTR
ENSG00000141279	ENSG00000274611	ENSE00003785912	ENSE00003731868	AACATGTATTTAACCAAGTTCCAACAAAAGAATGCTGCCACAG*GATGGACGTGGTAGAGGTCGCGGGCAGTTGGTGGGCACAAGAG	CDS(complete)/UTR
ENSG00000257764	ENSG00000147789			CACGCCTGTAATCCCAGCACTTTGGGAGGCCGAGGTGGGTGGATCACAAG*GTCAGGAGATCGAGACCATCCTGGCCAACACGGTGAAACCCTGTGTCTAC	intronic/UTR
ENSG00000120071	ENSG00000185829	ENSE00003662350	ENSE00003806456	TCGTCAGCAAACAGACATTTACAAACAGATACGTGCTAATAAG*GTTTCTGTGTGGAGACAGTAGAATATAAAAATAACACCTTCGC	out-of-frame
ENSG00000120071	ENSG00000228696	ENSE00003662350	ENSE00002370389	TCGTCAGCAAACAGACATTTACAAACAGATACGTGCTAATAAG*GTTTCTGTGTGGAGACAGTAGAATATAAAAATAACACCTTCGC	out-of-frame
ENSG00000090382	ENSG09000001070			AATTTACCTAAAACCTTGGTTATCAAATACATCTCCAGTACATTCCGTTC*TTTTTTTTTTTGAGACAGTCTCGCTCTTGTCGCCCAGGCTGGAGTGCAG	UTR/---
ENSG00000090382	ENSG09000001070			CCTCCCGAGTAGCTGGGATTACGGGCGCCCGCCACCACGCCCGGCTAATT*TTTTTGTATTTTTAGTAGAGACGGTGTTTCACCATGTTGGTCAGGCTGAT	UTR/---
ENSG00000090382	ENSG09000001070			TCCCGAGTAGCTGGGATTACGGGCGCCCGCCACCACGCCCGGCTAATTTT*TTTGTATTTTTAGTAGAGACAGGGTTTAACCACGTTGGCCAGGCTGGTCT	UTR/---
ENSG00000090382	ENSG09000001070			TTACCTAAAACCTTGGTTATCAAATACATCTCCAGTACATTCCGTTCTTT*TTTTTTTGAGACAGACTCTCACTCTGTCACCCAGGCTCGAGTGCAGTGGC	UTR/---
ENSG00000250271	ENSG00000114771			AAGAAGCTTATTTTATAACCATGGTGGTGGCTCATGTTTGAGTAGTAATG*AGACCAAGAAGCGGGACGTTCACCATGGGAAGAAAATCGCTGTACCTTCT	exonic(no-known-CDS)/UTR

ENSG00000136521	ENSG00000177238			CAGTTGTACGTAAGAAAACCCAATTCCCCCTGAGAAAGAGAAAGAGCTGG*AGTCCTTTAAAAATTAACTGCCTGTTTTTCTGTGGATAGTGAGCCTTATC	UTR/UTR
ENSG00000170421	ENSG00000106541			TTACTACCTGGGGACCCCCCTTGCCCATGCCTCCAGCTACAAAACAATTC*AATTTCTTTTTTTTTTTTTGAGACGGAGTCTCACTCTGTCGCCCAGGCTG	UTR/intronic
ENSG00000205323	ENSG00000228335			CATTTGGTTTTAGGAAGAAGAAACAAAGCCCATTGAGCTCCCTGTCAAAG*AGGAAGAACCCCCTGAAAAAAACTGTTGATGTGGCAGCAGAGAAGAAAGT	CDS(truncated)/exonic(no-known-CDS)
ENSG00000249437	ENSG00000197822	ENSE00003590701	ENSE00003560198	TTATTGATCCAAATTTCAGCTGAGTATGATCCTTCCAAACTAG*GTTAAAAATGTGTCTGCAGGCACACAGGACGTGCCTTCACCCC	out-of-frame
ENSG00000157106	ENSG00000243716	ENSE00001505919	ENSE00002138288	AGGTATACTATGTACCAGAATCAGTTGTTGGAGAAAATTAAAG*GTATGATCTCGTGAAATCTTGAGAGAAACTGAATGACGAATGA	CDS(truncated)/UTR
ENSG00000171634	ENSG00000238083	ENSE00002689718	ENSE00002373231	GAAAGCAGCTTCAGGAGCCATAGTACCTACAGCAGCACTCCAG*AAATTTCCAAGGAAACTATATTTCTTACATTGATGGAAATGTA	out-of-frame
ENSG00000090382	ENSG09000001070			GGAGTGCAGTGGCGCAATCTCGGCTCACTGCAACCTCCACCTCCCGGGTT*CCGCCATTCTCCTGCCTCAGCCTCTCGAGTAGCTGGGACTACAGGCGCCC	UTR/---
ENSG00000090382	ENSG09000001070			CTCCCGAGTAGCTGGGATTACGGGCGCCCGCCACCACGCCCGGCTAATTT*TTTGTATTTTTAGTAGAGACAGGGTTTAACCACGTTGGCCAGGCTGGTC	UTR/---
ENSG09000001070	ENSG00000090382			GTTGGCCAGGCTGATCTCGAATTCCTGACCTTGTGATCCACCCACCTCGG*CCCAAAGTGCTGGGATTACAGGCGTGAGCCACTGCGCCCGGCCACATTCA	---/UTR
ENSG09000001072	ENSG00000257764			GACCAGCCTGGCCAACGTGGTTAAACCCTGTCTCTACTAAAAATACA*AAATTAGCCGGGCGTGGTGGCGGGCGCCCGTAATCCCAGCTACTCGGGAG	---/intronic
ENSG00000090382	ENSG00000181856			AATTTACCTAAAACCTTGGTTATCAAATACATCTCCAGTACATTCCGTTC*TTTTTTTTTTTTGAGACAGTCTCGCTCTGTCGCCCAGGCTCGAGTGCAG	UTR/UTR
ENSG00000112576	ENSG00000105388			CTCCAGCCTGGCACAGAGCAAGACTCCATCTAAAAAAAAAAAAAAAA*AAGACTCTGACCTGTACTCTTGAATACAAGTTTCTGATACCACTGCACTG	intronic/UTR
ENSG00000112576	ENSG00000105388			TGCACTCCAGCCTGGGCAACAGAGCAAGACTCCATCTCAAAAAAAAAAA*AAAAGAAGACTCTGACCTGTACTCTTGAATACAAGTTTCTGATACCACTG	intronic/UTR
ENSG00000112576	ENSG00000105388			CCTGGGTGACAGAGTGAGACCCTGTCTCAAAAAAAAAAACAAAAAAAAAC*AAGACTCTGACCTGTACTCTTGAATACAAGTTTCTGATACCACTGCACTG	intronic/UTR
ENSG00000112576	ENSG00000105388			CCAGCCTGGGTGACAGAGCAAGAGTCTGTCTCAAAAAAACAAAACAAAAC*AAGACTCTGACCTGTACTCTTGAATACAAGTTTCTGATACCACTGCACTG	intronic/UTR
ENSG00000204655	ENSG00000244038			AAAAATAAATAAATAAATAAATAAATAAATAAATAAATAAATAAATAAAA*AAAAATAAAGCGAGATGTTGCCCTCAAACTTCACCTGG	UTR/UTR
ENSG00000204655	ENSG00000244038			CCACTGCACTCTAGCCGGAGTGACAGAGTAAGACTCTGTCTCAAAAATAA*ATAAATAAATAAATAAATAAATAAATAAATAAATAAAAATAAAGCGAGAT	UTR/UTR
ENSG00000205084	ENSG00000257764			AGCGGTGGCTCACGCCTGTAATCCCAGCACTTTGGGAGGCCGAGGTGGGT*GATCACAAGGTCAGGAGATCGAGACCATCCTGGCTAACACGGTGAAACCC	UTR/intronic
ENSG00000232721	ENSG00000224174	ENSE00001605525	ENSE00001677405	GGACGACTGAGCAAGGCTTTGGAAAACCAGAGAGATTAGAGCG*AATGGGGAAATGGAGAGAGAACCTGAAAGAGCCCCAAACTCGA	exonic(no-known-CDS)/exonic(no-known-CDS)
ENSG00000090382	ENSG00000182718			AATTTACCTAAAACCTTGGTTATCAAATACATCTCCAGTACATTCCGTTC*TTTTTTTTTTTTTTTTTTTTTTGTGACAGGGTCTTGCTCTGTCACCC	UTR/intronic
ENSG00000272398	ENSG00000127324			AAACAGATCGAACAGTTTTGAAGCTACTGTGTGTGTGAATGAACACTCTT*TTGATTTATTCTAGAATGCTGGCCAGTCTGTTTCTGGAGAGTAGGGACCT	UTR/intronic
ENSG00000090382	ENSG00000181856			AATTTACCTAAAACCTTGGTTATCAAATACATCTCCAGTACATTCCG*TTTTTTTTTTTTGAGACAGTCTCGCTCTGTCGCCCAGGCTCGAGTGCAGT	UTR/UTR
ENSG00000257764	ENSG00000073910	ENSE00002336888	ENSE00003270899	CCGGGCGCAGTGGCTCACGCCTGTAATCCCAGCACTTTGGGAG*GCCGAGGTGGGCGGATCACAAGGTCAGGAGATCGAGACCATCC	exonic(no-known-CDS)/UTR
ENSG00000257764	ENSG00000147789			CACGCCTGTAATCCCAGCACTTTGGGAGGCCGAGGTGGGTGGATCACAAG*GTCAGGAGATCGAGACCATCCTGGCCAACACGGTGAAACCCTGTGTCTAC	intronic/UTR
ENSG00000141279	ENSG00000274611	ENSE00003785912	ENSE00003731868	AACATGTATTTAACCAAGTTCCAACAAAAGAATGCTGCCACAG*GATGGACGTGGTAGAGGTCGCGGGCAGTTGGTGGGCACAAGAG	CDS(complete)/UTR
ENSG00000140451	ENSG00000244038			TCAAGACTATCCTGGCTAACACGGTAAAACCCTGTCTCTACTAAAAATAC*AAAATTAGCTGGGCGTGGTGGTGGGCACCTGTAGTCCCAGCTACTCGGGA	UTR/UTR
ENSG00000166762	ENSG00000168781	ENSE00003653606	ENSE00001774689	CATGGCAAAAAGCTACCACCTGCCAGTCTGAAGCACCGAGATG*AGCTCTTGTTTGTCCCGGCCGTAAAACGATTTTCTGTGTCGTT	UTR/CDS(truncated)
ENSG00000132465	ENSG00000075624			CATGAGCCACCGTGCCTGGCTTTTTTTTTTTTTTTTTTTTTTTTTTTTTTT*TTTTTTTTTTTTTTTTGGCTTGACTCAGGATTTAAAAACTGGAACGGTGA	intronic/UTR
ENSG00000132465	ENSG00000075624			GGACTGAAAATCAGGAGGTGTAATAAAATGTTCCTCGCATTCCCCCCCGC*GGTTTTTTTTTTTTTTTTGGCTTGACTCAGGATTTAAAAACTGGAACGGT	UTR/UTR
ENSG00000090382	ENSG00000270800			ACCTAAAACCTTGGTTATCAAATACATCTCCAGTACATTCCGTTCTTTTT*TTTTTGAGACAGTCTCGCTCTGTCACCCAGGCTGGAGTGCAGTGGCAAGA	UTR/intronic
ENSG00000090382	ENSG00000270800			TAAAACCTTGGTTATCAAATACATCTCCAGTACATTCCGTTCTTTTTTT*TTTTTTTTTTTTTTTTTTGGGACGGAGTCTCGCTCTGTCGCCCATGCTAG	UTR/intronic
ENSG00000090382	ENSG00000270800			AAAATTTACCTAAAACCTTGGTTATCAAATACATCTCCAGTACATTCCGT*TCTTTTTTTTTTTTTGAGACAGGATCTTGCTCTGTCACCCAGGCTGGAGT	UTR/intronic
ENSG00000168781	ENSG00000166762	ENSE00001774689	ENSE00001581568	GTAAAACGATTTTCTGTGTCGTTTGCAAAGCATCCGACTAACG*AGGTATCTGCCGGTACTGAGATGTAAGTGATCAGCTGAAGGGA	CDS(truncated)/UTR
ENSG00000168781	ENSG00000166762	ENSE00003788646	ENSE00003500886	CATGGCAAAAAGCTACCACCTGCCAGTCTGAAGCACCGAGATG*AATTGCTGGAATCCACAAATACCAAACTATGGCCATTGAAGCT	in-frame
ENSG00000168781	ENSG00000166762	ENSE00003788646	ENSE00003527303	CATGGCAAAAAGCTACCACCTGCCAGTCTGAAGCACCGAGATG*ACATGGCCGCTTACCAACAAGAAGAGCAGATGCAGCTTCCCCG	in-frame
ENSG00000153561	ENSG00000153107	ENSE00002531730	ENSE00002441208	CATCCACAGCAGTGTTTCTCGGGTTGGAAAAGCCATTGATAAG*GATTCACTTTAAGAGATTTGGAAACTCTTCCCTTTGGAATTGC	out-of-frame
ENSG00000157106	ENSG00000243716	ENSE00001505919	ENSE00002138288	AGGTATACTATGTACCAGAATCAGTTGTTGGAGAAAATTAAAG*GTATGATCTCGTGAAATCTTGAGAGAAACTGAATGACGAATGA	CDS(truncated)/UTR
ENSG00000090382	ENSG00000181856			AATTTACCTAAAACCTTGGTTATCAAATACATCTCCAGTACATTCCGTTC*TTTTTTTTTTGAGACAGTCTCGCTCTGTCGCCCAGGCTCGAGTGCAGTGG	UTR/UTR
ENSG00000090382	ENSG09000001070			CTCCCGAGTAGCTGGGATTACGGGCGCCCGCCACCACGCCCGGCTAATTT*TTTGTATTTTTGGTAGAGACAGGGTTTCTCCATGTTGGCCAGGCTGGTC	UTR/---
ENSG00000257764	ENSG00000147789			CACGCCTGTAATCCCAGCACTTTGGGAGGCCGAGGTGGGTGGATCACAAG*GTCAGGAGATCGAGACCATCCTGGCCAACACGGTGAAACCCTGTGTCTAC	intronic/UTR
ENSG00000147789	ENSG00000257764			CGAGACCATCCTGGCCAACACGGTGAAACCCTGTGTCTACTAAAAATACA*AAAAAATTAGCCGGGCGTGGTGGCGGGCGCCCGTAATCCCAGCTACTCGG	UTR/intronic
ENSG00000090382	ENSG09000001072			CCTCCCGAGTAGCTGGGATTACGGGCGCCCGCCACCACGCCCGGCTAATT*TTTTTGTATTTTTAGTGGAGACGGGGTTTCACCGTGTTAGCCAGGATGGT	UTR/---
ENSG00000175538	ENSG00000160993			GCAGTCTTTTCAGTTTTGACTGAGTGATGTGAGAAACTTCTTTTCTTTTC*TTTTTTTTTTTTTGAGACAGGGTCTTGCTTTGTTGTCCAGGCTGGAGTG	UTR/intronic
ENSG09000001072	ENSG00000257764			GCTCTTTAGTTTAATTAGATCCATTTTGTCAATTTTTTGTTTTTGTTGCA*AAGCCTTTAATTAGAATGTTTGTATTTTTTACATCATGCATAACTTCACA	---/exonic(no-known-CDS)
ENSG09000001070	ENSG00000257764			TGAGACCATCCTGGCTAACACGGTGAAACCCCGTCTCCACTAAAAATACA*AAAAAATTAGCCGGGCGTGGTGGCGGGCGCCCGTAATCCCAGCTACTCGG	---/intronic
ENSG00000257764	ENSG00000145817			GCCGGGCGCAGTGGCTCACGCCTGTAATCCCAGCACTTTGGGAGGCCGAG*GCGGGCGGATCACAAGGGCAGGAGATCGAGACCATCCTGGTTAACACGGT	intronic/UTR
ENSG00000181852	ENSG00000257764			CATCCTGGCTAACACATTGAAACCCCGTCTCTACTAAAAATACAAAAAAC*TAGCCGGGCGTGGTGGCGGGCGCCCGTAATCCCAGCTACTCGGGAGGCTG	intronic/intronic
ENSG00000123815	ENSG00000105245			CTCCAACAGCTGCCTTCTCCCGGGTTCCAGCCTCTCAGTGTGTTGGAGAG*GGCGGACCCCGGAGGCCTGAGCGGCACCTGCCCCCAGCCCCCTGTGGGGC	UTR/CDS(truncated)
ENSG00000205323	ENSG00000228335			CATTTGGTTTTAGGAAGAAGAAACAAAGCCCATTGAGCTCCCTGTCAAAG*AGGAAGAACCCCCTGAAAAAAACTGTTGATGTGGCAGCAGAGAAGAAAGT	CDS(truncated)/exonic(no-known-CDS)
ENSG09000001012	ENSG00000013588			CTTGTTTAAGGCTGAATTATATTCCATTGTGTGTGTGTGTGTGTGTGTGC*GCGTGTATGTGCGCCTGACCCTGATTTCTGCAACCTCCAGATTTCTTTCT	---/UTR
ENSG00000090382	ENSG00000179218			TGCCTCAGCCTCCCGAGTAGCTGGGATTACGGGCGCCCGCCACCACGCCC*GGCTGATTTTTTGTATTTTTAGTAGAGACGGGGTTTCACCGTGTTAGCCA	UTR/UTR
ENSG00000090382	ENSG00000138326			TGGTTATCAAATACATCTCCAGTACATTCCGTTCTTTTTTTTTTTGAGAC*AGTTTAGCTCTGTCGCCCAGGCTGGAGTGCAGTGGTACAATCTCCTTGGC	UTR/intronic
ENSG00000141279	ENSG00000274611	ENSE00003785912	ENSE00003731868	AACATGTATTTAACCAAGTTCCAACAAAAGAATGCTGCCACAG*GATGGACGTGGTAGAGGTCGCGGGCAGTTGGTGGGCACAAGAG	CDS(complete)/UTR
ENSG00000138326	ENSG00000090382			CGCCTGGCTAATTTTTGTATTTTTAGTAGAGACGGGGTTTCACCGTGTTG*GCCAGGATGGTCTCGATCTCCTGACCTTGTGATCCACCCACCTCGGCCTC	UTR/UTR
ENSG00000090382	ENSG00000181856			AATTTACCTAAAACCTTGGTTATCAAATACATCTCCAGTACATTCCGTTC*TTTTTTTTTTTTTTTTTTTTGAGACAGTCTCGCTCTGTCGCCCAGGCTC	UTR/UTR
ENSG00000198056	ENSG00000196531			CTTCAAGAGCCATTTAATAAATATGGCAGAACTATATATGTGTCTTAAAC*CTCAAAGTAAATTTTCCTTGAGAAATAATTTATGTTGAAAAGATTTCCTG	UTR/UTR
ENSG00000090382	ENSG09000001070			TCCTGCCTCAGCCTCCCGAGTAGCTGGGATTACGGGCGCCCGCCACCACG*CCCGGCTAATTTTTTTGTATTTTTAGTAGAGACGGGGTTTCACCGTGGTC	UTR/---
ENSG00000157106	ENSG00000243716	ENSE00001505919	ENSE00002138288	AGGTATACTATGTACCAGAATCAGTTGTTGGAGAAAATTAAAG*GTATGATCTCGTGAAATCTTGAGAGAAACTGAATGACGAATGA	CDS(truncated)/UTR
ENSG00000104408	ENSG00000147655	ENSE00002112612	ENSE00001378581	GCATCTAGTCTTTCCGCTTCTTGAATTTCTCTCTGTAAAGGAG*GTTCGTGGCGGAGAGATGCTGATCGCGCTGAACTGACCGGTGC	CDS(truncated)/UTR

ENSG00000141279	ENSG00000274611	ENSE00003785912	ENSE00003731868	AACATGTATTTAACCAAGTTCCAACAAAAGAATGCTGCCACAG*GATGGACGTGGTAGAGGTCGCGGGCAGTTGGTGGGCACAAGAG	CDS(complete)/UTR
ENSG00000153561	ENSG00000153107	ENSE00002531730	ENSE00002441208	CATCCACAGCAGTGTTTCTCGGGTTGGAAAAGCCATTGATAAG*GATTCACTTTAAGAGATTTGGAAACTCTTCCCTTTGGAATTGC	out-of-frame
ENSG00000171848	ENSG00000163009	ENSE00003659027	ENSE00001489842	GTTTGTGGCAGACAGACTTATGCTGGAACTGGGTTTTAGCAAG*GTGCTGGGAGACCGTGAAGTGCAAAGCAGATGGAGTCCAGGCC	CDS(truncated)/exonic(no-known-CDS)
ENSG00000123815	ENSG00000105245	ENSE00003529650	ENSE00003482506	GAAGTCCAGGGACCTCAAATTCCTCACAGGCTTTGAAACCAAG*GGCGGACCCCGGAGGCCTGAGCGGCACCTGCCCCCAGCCCCCT	in-frame
ENSG00000090382	ENSG00000181856			AATTTACCTAAAACCTTGGTTATCAAATACATCTCCAGTACATTCCGTTC*TTTTTTTTTTGAGACAGTCTCGCTCTGTCGCCCAGGCTCGAGTGCAGT	UTR/UTR
ENSG00000254610	ENSG00000283674	ENSE00003779619	ENSE00003799182	CAGGAAGGAGACCGGCACCCAGACAGGTGGCGACGGCAGAGGA*GAAGGAAAATACATGTACAGCCAACTTCCTTGAGGGTTCGTTC	exonic(no-known-CDS)/exonic(no-known-CDS)
ENSG00000283674	ENSG00000254610	ENSE00003792578	ENSE00002156751	CAAGGAGGACACCGGCACCCAGACAGGTGGCGACGGCAGAGGA*GAAGGAAAATACATGTACAGCCAACTTCCTTGAGGATTCGTTC	exonic(no-known-CDS)/exonic(no-known-CDS)
ENSG00000116251	ENSG00000085276			TCAAAGGGCTTCCCTTGGTTCGCCACTCATTTATTTGTAACTTGACTTCT*TTTTTTTTTCTGCTTAAAAATTTCAATTCTCGTGGTAATACCAGAGTAGA	UTR/intronic
ENSG00000116251	ENSG00000085276			TGTTATCATTTGTGTATTTTCTTAGAAAAAGCTTGTGGTGAAGGGGGGCA*AAAAAAAAGAAGCAAGTTCTGAAGTTCACTCTTGATTGCACCCACCCTGT	CDS(truncated)/intronic
ENSG00000116251	ENSG00000085276			TAGTGGAGTTCTTGTGTCCTTTCTCTGCAGGTATTTGAAATATCTCACCA*AAAAAATATTTGAAGAAGAATAATCTACGTGACTGGTTGCGCGTAGTTGC	CDS(truncated)/intronic
ENSG00000116251	ENSG00000085276			TCACTCTTGATTGCACCCACCCTGTAGAAGATGGAATCATGGATGCTG*ATTTTGAGCAGTTTTTGCAAGAAAGGATCAAAGTGAACGGAAAAGCTGGG	CDS(truncated)/intronic
ENSG00000261278	ENSG00000055044			CCATGTACCAGCACAACAATTCCTAGTCCAGAGAAAAAGAAGAAAAAGA*AAAAAAAAGAGAGAGAACGAGGATTAACAGAAAGGAATTACGATTATATC	exonic(no-known-CDS)/CDS(truncated)
ENSG00000122565	ENSG00000128891			ACTTGTGTTCTCATGAGCCTAAGGACCATTCTAGATTTATTACGTGTTTT*TTTGTGTGTGTGTGTGTGTGTGTGTGTGTGTGTTAAAATATATGTAGCAT	UTR/intronic
ENSG00000170421	ENSG00000119888			ACTACCTGGGGACCCCCCTTGCCCATGCCTCCAGCTACAAAACAATTCAA*TTTCTTTTTTTTTTTTGGAATGGAGTCTCGCTGGCCGGACGTGGTGGCTC	UTR/intronic
ENSG00000055044	ENSG00000261278			GCAAAATAGAACAGGTAGATAAAGAGGATGAAATTACTGAAAAGAAAGCC*AAAAAAAGCCAAGATTAAAGTTAAAGCTGAAGAAGAGGAATAAGAAGAAA	CDS(truncated)/exonic(no-known-CDS)
ENSG00000160862	ENSG00000176402	ENSE00001055051	ENSE00001231504	GAGACCCTGAAAGACATCGTGGAGTATTACAACGACAGTAACG*TTCCAGGAAGAAGCTTAGCCCAGGAAAAACAAAGACCAGTTGG	in-frame
ENSG00000141279	ENSG00000274611	ENSE00003785912	ENSE00003731868	AACATGTATTTAACCAAGTTCCAACAAAAGAATGCTGCCACAG*GATGGACGTGGTAGAGGTCGCGGGCAGTTGGTGGGCACAAGAG	CDS(complete)/UTR
ENSG00000059769	ENSG00000106689	ENSE00001519553	ENSE00003715988	GGAGGCTTTCCTGCTGGTGGCAACCGCCTACGAGACACTCAAG*ACCATGCCGTCCATCAGCAGTGACCGCGCCGCGCTGTGCGCCG	in-frame
ENSG00000090382	ENSG00000181856			AATTTACCTAAAACCTTGGTTATCAAATACATCTCCAGTACATTCCGTTC*TTTTTTTTTTTTTTTTTTTTGAGACAGTCTCGCTCTGTCGCCCAGGCTC	UTR/UTR
ENSG00000244115	ENSG00000106689			GCGCCGAGGAGGCTTTCCTGCTGGTGGCAACCGCCTACGAGACACTCAAG*ACCATGCCGTCCATCAGCAGTGACCGCGCCGCGCTGTGCGCCGGCTGCGG	in-frame
ENSG00000157106	ENSG00000243716	ENSE00001505919	ENSE00002138288	AGGTATACTATGTACCAGAATCAGTTGTTGGAGAAAATTAAAG*GTATGATCTCGTGAAATCTTGAGAGAAACTGAATGACGAATGA	CDS(truncated)/UTR
ENSG00000157106	ENSG00000243716			TTACGTTCATGTAAACAGCATGACGTGAGGCCATGGATGCAGGCATTAAG*GTATGATCTCGTGAAATCTTGAGAGAAACTGAATGACGAATGAAACTATT	CDS(truncated)/UTR
ENSG00000157106	ENSG00000243716			AGGCATTAAGGTATACTATGTACCAGAATCAGTTGTTGGAGAAAATTAAA*AGCTTGAAATGAATTTTAAAGGATGACTGATGGTCCTTGGAAGAGAAACA	CDS(truncated)/UTR
ENSG00000141279	ENSG00000274611	ENSE00003785912	ENSE00003731868	AACATGTATTTAACCAAGTTCCAACAAAAGAATGCTGCCACAG*GATGGACGTGGTAGAGGTCGCGGGCAGTTGGTGGGCACAAGAG	CDS(complete)/UTR
ENSG00000250271	ENSG00000114771			CCCATCTGCCATCACTGCTTTGGAGCCCAGGGTAACTCTGCCAGTAACAG*AGACCAAGAAGCGGGACGTTCACCATGGGAAGAAAATCGCTGTACCTTCT	exonic(no-known-CDS)/UTR
ENSG00000013588	ENSG09000001012			TTCAAGCGATTTTCGTGCCTCGGCCTTCTGAGTAGCTGGGATTACAGGCG*TGCGCCACCATGCCTGGCTAATTTTTGTATTTTTAGTAGAGACAGGGTTT	UTR/---
ENSG00000170421	ENSG00000075624			CCCATTTGCTACATTTTTTTTTTTTTTTTTTTTTTTTTTTTTTTTTTTTT*GGCTTGACTCAGGATTTAAAAACTGGAACGGTGAAGGTGACAGCAGTCGG	intronic/UTR
ENSG00000170421	ENSG00000075624			TAGCTTTTTTGTAGGTAGGTTCTTCAGGATTTTTTTGTTTTTGTTTTTT*TTTTTTTTTTTTTTTTGGCTTGACTCAGGATTTAAAAACTGGAACGGTGA	intronic/UTR
ENSG00000170421	ENSG00000075624			ATTACAGGCGTGAGCCACTGCACCCGGTGGATTTTTTTTTTGTTTTGTTT*TTTTTTTTTTTTTTTTGGCTTGACTCAGGATTTAAAAACTGGAACGGTGA	intronic/UTR
ENSG00000174567	ENSG00000170498	ENSE00001377126	ENSE00001125940	CCCCAGGCTGAGTGCGGCATGATCTCCATCACCGAATGGCAGA*CCTCAAGGCACTTCTAGGACCTGCCTCTTCTCACCAAGATGAA	CDS(truncated)/UTR
ENSG00000120071	ENSG00000185829	ENSE00003662350	ENSE00003806456	TCGTCAGCAAACAGACATTTACAAACAGATACGTGCTAATAAG*GTTTCTGTGTGGAGACAGTAGAATATAAAAATAACACCTTCGC	out-of-frame
ENSG00000120071	ENSG00000228696	ENSE00003662350	ENSE00002370389	TCGTCAGCAAACAGACATTTACAAACAGATACGTGCTAATAAG*GTTTCTGTGTGGAGACAGTAGAATATAAAAATAACACCTTCGC	out-of-frame
ENSG00000249437	ENSG00000197822	ENSE00003590701	ENSE00003560198	TTATTGATCCAAATTTCAGCTGAGTATGATCCTTCCAAACTAG*GTTAAAAATGTGTCTGCAGGCACACAGGACGTGCCTTCACCCC	out-of-frame
ENSG00000153561	ENSG00000153107	ENSE00002531730	ENSE00002441208	CATCCACAGCAGTGTTTCTCGGGTTGGAAAAGCCATTGATAAG*GATTCACTTTAAGAGATTTGGAAACTCTTCCCTTTGGAATTGC	out-of-frame
ENSG00000090382	ENSG00000181856			AATTTACCTAAAACCTTGGTTATCAAATACATCTCCAGTACATTCCGTTC*TTTTTTTTTTTTGAGACAGTCTCGCTCTGTCGCCCAGGCTCGAGTGCAGT	UTR/UTR
ENSG00000141279	ENSG00000274611	ENSE00003785912	ENSE00003731868	AACATGTATTTAACCAAGTTCCAACAAAAGAATGCTGCCACAG*GATGGACGTGGTAGAGGTCGCGGGCAGTTGGTGGGCACAAGAG	CDS(complete)/UTR
ENSG00000120071	ENSG00000185829	ENSE00003662350	ENSE00003806456	TCGTCAGCAAACAGACATTTACAAACAGATACGTGCTAATAAG*GTTTCTGTGTGGAGACAGTAGAATATAAAAATAACACCTTCGC	out-of-frame
ENSG00000120071	ENSG00000228696	ENSE00003662350	ENSE00002370389	TCGTCAGCAAACAGACATTTACAAACAGATACGTGCTAATAAG*GTTTCTGTGTGGAGACAGTAGAATATAAAAATAACACCTTCGC	out-of-frame
ENSG00000183955	ENSG00000279730			CAGCCATCGCCAAGCAAGCCCTGAAAAAGCCCATCAAGGGCAAACAG*CCCCCGAAAAAAAGCTCAAGGAAAAACGCAACAGAATCGCAAACACGGAT	CDS(truncated)/exonic(no-known-CDS)
ENSG00000183955	ENSG00000279730			GGGGCGCCGCCGGCCCTTCCCCGGGGCGCCGCGGCGGCGCTGGTGGC*GGCAGCAGCAGCAGCAGCGGCAGCGGCAGCGGTGGTGGCCGGGCAGCGGC	UTR/exonic(no-known-CDS)
ENSG00000183955	ENSG00000279730			CGCCGGCCATGGGGGAAGGGGGCGCCGTGGGGCGCCGCCGGCCCTTCCCC*GGGGCGCCGCGGCGGCGCTGGTGGCGGCGGCAGCAGCAGCAGCAGCGG(	UTR/exonic(no-known-CDS)
ENSG00000283674	ENSG00000117519			CACCTGTAATCCCAGCACTTTGGGAGGCCGAGGCTGGCGGATCACCTAAG*GTCAGGAGTTCGAGACCAGGCTGGCCAACATGGTGAAACCCCGTCTCTGC	exonic(no-known-CDS)/intronic
ENSG00000079112	ENSG00000105388			AGAGCAGACTTGGGAAGATCTGAGAATTAAAAAAAAAAAAAAAACAAACC*AAGACTCTGACCTGTACTCTTGAATACAAGTTTCTGATACCACTGCACTG	intronic/UTR
ENSG00000170421	ENSG00000126709			AGTTCTGGCTCTCCCATTTGCTACATTTTTTTTTTTTTTTTTTTTTTTTT*TTTTTTTTTTTTTTTTTTTTTTTGAGATGGGTTCTCACTATATTGTCCAG	intronic/UTR
ENSG00000170421	ENSG00000126709			AGGTGTGAGCCACTGTGCCCGGCCTTTTTTTTTTTTTTTTTTTTTTTTTT*TTTTTTTTTTTTTTTTTTTTTTTTGAGATGGGTTCTCACTATATTGTCCAG	intronic/UTR
ENSG00000162896	ENSG00000076554			TCACACATAAGAAAATTGAGGTTTGGAAGAGTGAAGCGTTTTTCTTTTTC*TTTTTTTTTTTTTGAGACGGAGTCTCACACTTTCGCCCAGGCTGGAGTGA	UTR/intronic
ENSG00000157106	ENSG00000243716	ENSE00001505919	ENSE00002138288	AGGTATACTATGTACCAGAATCAGTTGTTGGAGAAAATTAAAG*GTATGATCTCGTGAAATCTTGAGAGAAACTGAATGACGAATGA	CDS(truncated)/UTR
ENSG00000172757	ENSG00000167004	ENSE00001344370	ENSE00001489318	CACGGCTACTCATGGAAGCAGGACCAGTAAGGGACCTTCGATT*AAAAAAAAAAAAAAAAAAAAAAAAAAAAAAAAAAAAAAAAAAA	UTR/UTR
ENSG00000135052	ENSG00000090402	ENSE00001858995	ENSE00000999825	AGTGATTCTTCTGCCTCAGCCTCCCGAGTAGCTAGGACTACAG*TCGACAAAAACACATGAAGCTCATTGTTGCTGCAGATGATAAT	UTR/CDS(truncated)
ENSG00000153561	ENSG00000153107	ENSE00002531730	ENSE00002441208	CATCCACAGCAGTGTTTCTCGGGTTGGAAAAGCCATTGATAAG*GATTCACTTTAAGAGATTTGGAAACTCTTCCCTTTGGAATTGC	out-of-frame

ENSG00000090382	ENSG00000181856			AATTTACCTAAAACCTTGGTTATCAAATACATCTCCAGTACATTCCGTTC*TTTTTTTTTTTTTTTTTTTGAGACAGTCTCGCTCTGTCGCCCAGGCT	UTR/UTR
ENSG00000112576	ENSG00000105388			ACTGCACTCCAGCCTGGCAACAGAGCAAGACTCCATCTCAAAAAAAAAAA*AAAAGAAGACTCTGACCTGTACTCTTGAATACAAGTTTCTGATACCACT	intronic/UTR
ENSG00000112576	ENSG00000105388			GCACTCCAGCCTGGCACAGAGCAAGACTCCATCTAAAAAAAAAAAAAAAA*GAAGACTCTGACCTGTACTCTTGAATACAAGTTTCTGATACCACTGCACT	intronic/UTR
ENSG00000157106	ENSG00000243716			TTACGTTCATGTAAACAGCATGACGTGAGGCCATGGATGCAGGCATTAAG*GTATGATCTCGTGAAATCTTGAGAGAAACTGAATGACGAATGAAACTATT	CDS(truncated)/UTR
ENSG00000279080	ENSG00000257594			CTAACACGGTGAAACCTCGTCTCTACTAAAAATACAAAAAAATTAGCCGG*CTGTGGTGGCGGGCGCCTGTAGTCCCAGCTACTCAGGAGGCTGAGGCAGG	exonic(no-known-CDS)/UTR
ENSG00000162896	ENSG00000173702			ATTCTTACCATTTCACACATAAGAAAATTGAGGTTTGGAAGAGTGAAGCG*TTTTTCTTTTTCTTTTTTTTTTTTTGAGACATTGTCTGGCTCTGTCACCT	UTR/intronic
ENSG00000152700	ENSG00000228696			ATTAAGGACTAAATTATCAAAACTGCATGTTTGTTTTTTCTTTTCTTTTC*TTTTTTTTTTTGAGATGAAGTCTCACTCTGTTGCCCAGGCTGGAGTGCAG	UTR/UTR
ENSG00000182718	ENSG00000275395	ENSE00002555095	ENSE00003219075	AGTGATTCTCCAGCCTCACCCTCCCGAGTAGCTGGGATTACAG*GTGAACGGTGTGGACATGAAGCTGCCCGTGGTGCTGGCCAACG	UTR/CDS(truncated)
ENSG00000148468	ENSG00000152457	ENSE00001923221	ENSE00003475855	GCGGTGACCAAGACGCTGCGGGAGCCCGGCGCCGGAGCCCAAG*ATATCTATTGAAATCGAGACTCCTACCCAGATATCTTTAGTGG	out-of-frame
ENSG00000120071	ENSG00000185829	ENSE00003662350	ENSE00003806456	TCGTCAGCAAACAGACATTTACAAACAGATACGTGCTAATAAG*GTTTCTGTGTGGAGACAGTAGAATATAAAAATAACACCTTCGC	out-of-frame
ENSG00000120071	ENSG00000228696	ENSE00003662350	ENSE00002370389	TCGTCAGCAAACAGACATTTACAAACAGATACGTGCTAATAAG*GTTTCTGTGTGGAGACAGTAGAATATAAAAATAACACCTTCGC	out-of-frame
ENSG00000141279	ENSG00000274611	ENSE00003785912	ENSE00003731868	AACATGTATTTAACCAAGTTCCAACAAAAGAATGCTGCCACAG*GATGGACGTGGTAGAGGTCGCGGGCAGTTGGTGGGCACAAGAG	CDS(complete)/UTR
ENSG00000111319	ENSG00000067182	ENSE00003589571	ENSE00003524766	GGAGCTGAACTACAAAACCAATTCTGAGTCTCCCTCTGTCACG*GTGCTCCTGGAGCTGTTGGTGGGAATATACCCCTCAGGGGTTA	in-frame
ENSG00000168781	ENSG00000166762	ENSE00003788646	ENSE00003527303	CATGGCAAAAAGCTACCACCTGCCAGTCTGAAGCACCGAGATG*ACATGGCCGCTTACCAACAAGAAGAGCAGATGCAGCTTCCCCG	in-frame
ENSG00000153561	ENSG00000153107	ENSE00002531730	ENSE00002441208	CATCCACAGCAGTGTTTCTCGGGTTGGAAAAGCCATTGATAAG*GATTCACTTTAAGAGATTTGGAAACTCTTCCCTTTGGAATTGC	out-of-frame
ENSG00000090382	ENSG00000181856			AATTTACCTAAAACCTTGGTTATCAAATACATCTCCAGTACATTCCGTTC*TTTTTTTTTTTTTTTTTTTTGAGACAGTCTCGCTCTGTCGCCCAGGC	UTR/UTR
ENSG00000090382	ENSG09000001070			ACCTAAAACCTTGGTTATCAAATACATCTCCAGTACATTCCGTTCTTTTT*TTTTTTTTTTTTTTTTTTTTTAAAGAAACAAGGTCTTGTTATGTTGCTCA	UTR/---
ENSG00000090382	ENSG09000001070			AAAACCTTGGTTATCAAATACATCTCCAGTACATTCCGTTCTTTTTTTTT*TTTTTTTTTTTTTTTTTAAAGATGGCATCTCGCTGTGCTGCCTAGGCAGA	UTR/---
ENSG09000001070	ENSG00000090382			TTTTTGTATTTTTAGTAGAGACAGGGTTTCACCGTGTTAGCCAGGATGG*TCGATCTCCTGACCTTGTGATCCACCCACCTCGGCCTCCCAAAGTGCTGG	---/UTR
ENSG00000198056	ENSG00000196531			CTTCAAGAGCCATTTAATAAATATGGCAGAACTATATATGTGTCTTAAAC*CTCAAAGTAAATTTTCCTTGAGAAATAATTTATGTTGAAAAGATTTCCTG	UTR/UTR
ENSG00000157106	ENSG00000243716	ENSE00001505919	ENSE00002138288	AGGTATACTATGTACCAGAATCAGTTGTTGGAGAAAATTAAAG*GTATGATCTCGTGAAATCTTGAGAGAAACTGAATGACGAATGA	CDS(truncated)/UTR
ENSG00000257764	ENSG00000176046			GGTTATTTCTTTGATAAGAACTGAATGTGGCCGGGCGCAGTGGCTCACGC*CTGCAATCCCAGCACTTTGGGAGGCCGAGGTGGGAGGATTGCTTGGGCCC	exonic(no-known-CDS)/UTR
ENSG00000249437	ENSG00000197822	ENSE00003590701	ENSE00003560198	TTATTGATCCAAATTTCAGCTGAGTATGATCCTTCCAAACTAG*GTTAAAAATGTGTCTGCAGGCACACAGGACGTGCCTTCACCCC	out-of-frame
ENSG00000141279	ENSG00000274611	ENSE00003785912	ENSE00003731868	AACATGTATTTAACCAAGTTCCAACAAAAGAATGCTGCCACAG*GATGGACGTGGTAGAGGTCGCGGGCAGTTGGTGGGCACAAGAG	CDS(complete)/UTR
ENSG00000153561	ENSG00000153107	ENSE00002531730	ENSE00002441208	CATCCACAGCAGTGTTTCTCGGGTTGGAAAAGCCATTGATAAG*GATTCACTTTAAGAGATTTGGAAACTCTTCCCTTTGGAATTGC	out-of-frame
ENSG00000073969	ENSG00000176809	ENSE00003258850	ENSE00003547821	GAGAGGAGACTTCCTTGCTTCTTTGGAGAATGATATCAAACCA*AAATTTCCAAGGAAACTATATTTCTTACATTGATGGAAATGTA	in-frame
ENSG00000090382	ENSG00000181856			AATTTACCTAAAACCTTGGTTATCAAATACATCTCCAGTACATTCCGTTC*TTTTTTTTTTTTTTTTTTTTGAGACAGTCTCGCTCTGTCGCCCAGGCT	UTR/UTR
ENSG00000197892	ENSG00000246339			GGCTGGTGACCTCCCTGGGCCTCTGGAAGCTTTGTTTCTTCTGTACCCGG*TTCCTCTACTTGGCTCTCCTCCAAAGGTACGCGGCCACAGCAGGCAGGGG	UTR/exonic(no-known-CDS)
ENSG00000197892	ENSG00000246339			CCCTGGATGAAGAGCTGTCACCTCTGCCTTGCCCTTTAACTCAACCCGCT*CCCAGAGACAGGCTCGCAGCTGCCGACCTTATCCACTCGCCCTTTCTGCT	UTR/exonic(no-known-CDS)
ENSG00000153561	ENSG00000153107	ENSE00002531730	ENSE00002441208	CATCCACAGCAGTGTTTCTCGGGTTGGAAAAGCCATTGATAAG*GATTCACTTTAAGAGATTTGGAAACTCTTCCCTTTGGAATTGC	out-of-frame
ENSG00000230401	ENSG00000257764			TCCCAGCACTTGGGGAGGCTGAGGTGGGTGGATCACAAGGTCAGGAGATC*GACCATCCTGGCTAACACGGTGAAACCCTGTCTCTACTAAAAATACAAAA	exonic(no-known-CDS)/intronic
ENSG00000260411	ENSG00000283674	ENSE00002950429	ENSE00003796822	CAGGGAGGAGACCGGCACCCAGACAGGTGGCGACGGCAGAGGA*GTGCAGTGGTTCACACCTGTAATCCCAGCACTTTGGGAGGCCG	exonic(no-known-CDS)/exonic(no-known-CDS)
ENSG00000141279	ENSG00000274611	ENSE00003785912	ENSE00003731868	AACATGTATTTAACCAAGTTCCAACAAAAGAATGCTGCCACAG*GATGGACGTGGTAGAGGTCGCGGGCAGTTGGTGGGCACAAGAG	CDS(complete)/UTR
ENSG00000157106	ENSG00000243716			TTACGTTCATGTAAACAGCATGACGTGAGGCCATGGATGCAGGCATTAAG*GTATGATCTCGTGAAATCTTGAGAGAAACTGAATGACGAATGAAACTATT	CDS(truncated)/UTR
ENSG00000157106	ENSG00000243716	ENSE00001505919	ENSE00002138288	AGGTATACTATGTACCAGAATCAGTTGTTGGAGAAAATTAAAG*GTATGATCTCGTGAAATCTTGAGAGAAACTGAATGACGAATGA	CDS(truncated)/UTR
ENSG00000170949	ENSG00000204604			TCTCCCGTGATCCAGTGACATGAACTTGGGAAGAGGCTGCACTGGGCATG*GTCCTGGGAAGGGCTCACACCCCGACATGGATGGACACGGGGTGAGGGTC	UTR/CDS(truncated)
ENSG00000204604	ENSG00000170949			CCTTCAGTCCCTCTCATCTCGCTTAGGTTCCGTCTCTCGTGACCCAGTGT*CATGAACTTGGGAAGAGGCTGCACTGGGCATGGTCCTGGGAAGGGCTCAC	CDS(truncated)/UTR
ENSG00000174567	ENSG00000170498	ENSE00001377126	ENSE00001125940	CCCCAGGCTGAGTGCGGCATGATCTCCATCACCGAATGGCAGA*CCTCAAGGCACTTCTAGGACCTGCCTCTTCTCACCAAGATGAA	CDS(truncated)/UTR
ENSG00000120071	ENSG00000185829	ENSE00003662350	ENSE00003806456	TCGTCAGCAAACAGACATTTACAAACAGATACGTGCTAATAAG*GTTTCTGTGTGGAGACAGTAGAATATAAAAATAACACCTTCGC	out-of-frame
ENSG00000120071	ENSG00000228696	ENSE00003662350	ENSE00002370389	TCGTCAGCAAACAGACATTTACAAACAGATACGTGCTAATAAG*GTTTCTGTGTGGAGACAGTAGAATATAAAAATAACACCTTCGC	out-of-frame
ENSG00000120071	ENSG00000176809	ENSE00002635959	ENSE00003526242	ATAAAGAAAGAAAGGGATAGATGGAATGAGTTTCATCGTGATT*AATTCTCAATCACAATCCTCTGACAACTGTTGAAGATCCATAT	UTR/CDS(truncated)
ENSG00000249437	ENSG00000197822	ENSE00003590701	ENSE00003560198	TTATTGATCCAAATTTCAGCTGAGTATGATCCTTCCAAACTAG*GTTAAAAATGTGTCTGCAGGCACACAGGACGTGCCTTCACCCC	out-of-frame
ENSG00000090382	ENSG00000181856			AATTTACCTAAAACCTTGGTTATCAAATACATCTCCAGTACATTCCGTTC*TTTTTTTTTTTGAGACAGTCTCGCTCTGTCGCCCAGGCTCGAGTGCAG	UTR/UTR
ENSG00000090382	ENSG09000001070			GCCTCCCGAGTAGCTGGGATTACGGGCGCCCGCCACCACGCCCGGCTAAT*TTTTTGTATTTTTAGTAGAGACAGGGTTTCACCGTGTTAGCCAGGATGG	UTR/---
ENSG00000257764	ENSG09000001072			GGTTATTTCTTTGATAAGAACTGAATGTGGCCGGGCGCAGTGGCTCACGC*CTGTAATCCCAACACCTTGGGAGGCCGAGGCGGGTGGATCACATGACGTC	exonic(no-known-CDS)/---
ENSG09000001072	ENSG00000257764			CGAGACCATCCTGGCTAACACGGTGAAACCCTGTCTCTACTAAAAATACA*AAAAAATTAGCCGGGCGTGGTGGCGGGCGCCCGTAATCCCAGCTACTCGG	---/intronic
ENSG00000257764	ENSG00000147789			CACGCCTGTAATCCCAGCACTTTGGGAGGCCGAGGTGGGTGGATCACAAG*GTCAGGAGATCGAGACCATCCTGGCCAACACGGTGAAACCCTGTGTCTAC	intronic/UTR
ENSG00000090382	ENSG00000172175			GAGACAGGGTTTCACCGTGTTAGCCAGGATGGTCTCGATCTCCTGACCTT*GTCATCCACCCACCTCGGCCTCCCAAAGTGCTGGGATTACAGGCGTGAGC	UTR/intronic
ENSG00000141279	ENSG00000274611	ENSE00003785912	ENSE00003731868	AACATGTATTTAACCAAGTTCCAACAAAAGAATGCTGCCACAG*GATGGACGTGGTAGAGGTCGCGGGCAGTTGGTGGGCACAAGAG	CDS(complete)/UTR
ENSG00000120071	ENSG00000185829	ENSE00003662350	ENSE00003806456	TCGTCAGCAAACAGACATTTACAAACAGATACGTGCTAATAAG*GTTTCTGTGTGGAGACAGTAGAATATAAAAATAACACCTTCGC	out-of-frame
ENSG00000120071	ENSG00000228696	ENSE00003662350	ENSE00002370389	TCGTCAGCAAACAGACATTTACAAACAGATACGTGCTAATAAG*GTTTCTGTGTGGAGACAGTAGAATATAAAAATAACACCTTCGC	out-of-frame
ENSG00000205323	ENSG00000228335			CATTTGGTTTTAGGAAGAAGAAACAAAGCCCATTGAGCTCCCTGTCAAAG*AGGAAGAACCCCCTGAAAAAAACTGTTGATGTGGCAGCAGAGAAGAAAGT	CDS(truncated)/exonic(no-known-CDS)
ENSG00000082068	ENSG00000283352	ENSE00002056284	ENSE00003800756	TGGGGTGTGCGGCCAGCCATGGAGCGCTCTGGGCCCAGCGAAG*TGACAGGCTCAGACGCATCGGGACCGGACCCGCAGCTTGTGGT	CDS(truncated)/exonic(no-known-CDS)

ENSG00000198106	ENSG00000172530	ENSE00002235591	ENSE00002213839	GGTGCAGATCCACCAGATCGGAGAAGACGGACAGGTGCAAGTA*ATCCCACAGGGACACCTCCACATCGCCCAGGTGCCGCAGGGGG	exonic(no-known-CDS)/CDS(truncated)
ENSG00000105865	ENSG00000075790	ENSE00003520906	ENSE00003577399	TTAAAGGAAGCAGAAAATGTGTGGCGGATTACTGGGACAGATG*GTGTGAAGAAAAAAATGACACTCCAATGGGCTGCAGTGGCAAC	CDS(truncated)/UTR
ENSG00000249437	ENSG00000197822	ENSE00003590701	ENSE00003560198	TTATTGATCCAAATTTCAGCTGAGTATGATCCTTCCAAACTAG*GTTAAAAATGTGTCTGCAGGCACACAGGACGTGCCTTCACCCC	out-of-frame

Supplementary Table 5d: fusion genes identified in disease tissue samples (EAC, HGD and NDB), excluding (per sample) the fusion genes that are also detected in healthy tissue. Fusion gene pairs indicated in red are likely false positives, based on fusion description (see Supplementary Table 5a) or a high number of common mapping reads (>10).

sample ID	gene 1 symbol (5' end fusion partner)	gene 2 symbol (3' end fusion partner)	fusion_description	counts_of_common_mapping_reads	spanning_pairs	spanning_unique_reads	longest_anchor_found	fusion_finding_method	fusion_point_for_gene_1(5' end_fusion_partner)	fusion_point_for_gene_2(3' end_fusion_partner)	gene_1_id(5' end_fusion_partner)
ID20_EAC	VCL	ADK	known,adjacent,cell_lines,chimerdb3seq,cancer,10K<gap<100K,readthrough,exon-exon	0	6	5	30	BOWTIE	10:73998375:+	10:74394141:+	ENSG00000035403
	MTIF2	AL592494.2	no_protein,pseudogene,m2	10	5	2	32	BOWTIE+STAR	2:55240119:-	1:121503477:+	ENSG00000085760
	SMG1	NPIPB5	banned,known,bodymap2,hpa,m0,multi,exon-exon	0	4	4	22	BOWTIE	16:18858170:-	16:22513522:+	ENSG00000157106
	SMG1	NPIPB5	banned,known,bodymap2,hpa,m0,multi	0	4	2	35	BOWTIE+STAR	16:18858211:-	16:22513522:+	ENSG00000157106
	KANSL1	ARL17A	banned,known,healthy,bodymap2,hpa,gtex,18cancers,chimerdb3seq,m1,multi,exon-exon	0	3	3	26	BOWTIE	17:46094560:-	17:46570869:-	ENSG00000120071
	KANSL1	ARL17B	banned,known,healthy,bodymap2,hpa,chimerdb3seq,m0,multi,10K<gap<100K,exon-exon	0	3	3	26	BOWTIE	17:46094560:-	17:46352930:-	ENSG00000120071
	SPINK5	SPRR3	gtex	0	3	4	27	BOWTIE+STAR	5:148137336:+	1:153003002:+	ENSG00000133710
	GSN	ANXA1	cancer,exon-exon	0	2	3	20	BOWTIE	9:121248323:+	9:73158522:+	ENSG00000148180
	NAIP	OCLN	banned,known,bodymap2,hpa,1000genomes,m0,multi,exon-exon	0	2	4	30	BOWTIE	5:70974129:-	5:69534694:+	ENSG00000249437
ID29_EAC	EIF3F	AL356585.1	no_protein,pseudogene,cancer,m3	56	12	2	35	BOWTIE+STAR	11:7992129:+	13:18207169:-	ENSG00000175390
	ATXN1L	AL136982.5	no_protein,pseudogene,m2	6	5	4	27	BOWTIE+STAR	16:71849747:+	10:87027189:+	ENSG00000224470
	SPRR3	ANXA1	gtex,cancer,exon-exon	0	4	2	22	BOWTIE	1:153003855:+	9:73157654:+	ENSG00000163209
	KRT13	PKM	gtex,cancer	0	3	4	23	BOWTIE+STAR	17:41500991:-	15:72205126:-	ENSG00000171401
	NAIP	OCLN	banned,known,bodymap2,hpa,1000genomes,m0,multi,exon-exon	0	3	2	29	BOWTIE	5:70983775:-	5:69534694:+	ENSG00000249437
	SH3RF2	JMJD7-PLA2G4B	m2	1	3	3	31	BOWTIE+STAR	5:146080960:+	15:41837750:+	ENSG00000156463
	MKRN1	ANXA1	cancer,exon-exon	0	2	2	18	BOWTIE	7:140474381:-	9:73158522:+	ENSG00000133606
	POLA2	CDC42EP2	banned,known,adjacent,healthy,bodymap2,hpa,1000genomes,chimerdb3pub,1K<gap<10K,readthrough,exon-exon	0	1	2	25	BOWTIE	11:65295990:+	11:65320544:+	ENSG00000014138
ID30_EAC	LRPPRC	ACTB	cancer,m2	0	3	10	28	BOWTIE+STAR	2:43904701:-	7:5527638:-	ENSG00000138095
	SMG1	NPIPB5	banned,known,bodymap2,hpa,m0,multi	0	3	2	35	BOWTIE+BOWTIE2	16:18908242:-	16:22507338:+	ENSG00000157106
	SOD2	AHNAK		0	3	4	32	BOWTIE+STAR	6:159680959:-	11:62468372:-	ENSG00000112096
	AC027117.2	PSD3	no_protein,antisense,exon-exon	0	2	2	30	BOWTIE	8:17801742:+	8:18556352:-	ENSG00000253944
	ANXA2	KRT78	exon-exon	0	2	5	27	BOWTIE	15:60394574:-	12:52839984:-	ENSG00000182718
	BPTF	LRRC37A2	banned,known,hpa,m0,multi,exon-exon	0	1	3	28	BOWTIE	17:67826337:+	17:46517362:+	ENSG00000171634
ID43_EAC	*excluded from fusion gene analysis (see 'Methods' section)*										
ID2_HGD	AC020656.1	FRY	no_protein,antisense,m2	0	12	4	36	BOWTIE+STAR	12:69353766:-	13:32263749:+	ENSG00000257764
	AC020656.1	FRY	no_protein,antisense,m2,exon-exon	0	12	4	22	BOWTIE	12:69353743:-	13:32275142:+	ENSG00000257764
	NPEPPS	TBC1D3	banned,known,oncogene,bodymap2,hpa,gtex,18cancers,tumor,m0,multi,exon-exon	0	9	8	30	BOWTIE	17:47592545:+	17:38191030:-	ENSG00000141279
	AC020656.1	ZNF7	no_protein,antisense,m2	0	7	2	37	BOWTIE+STAR	12:69353721:-	8:144838728:+	ENSG00000257764
	LYZ	TRA@	m2	0	5	25	31	BOWTIE+STAR	12:69353497:+	14:21731216:+	ENSG00000090382
	LYZ	TRA@	m2	0	5	2	32	BOWTIE+STAR	12:69353657:+	14:21550403:+	ENSG00000090382
	LYZ	TRA@	m2	0	5	2	24	BOWTIE+STAR	12:69353659:+	14:21715808:+	ENSG00000090382
	LYZ	TRA@	m2	0	5	2	19	BOWTIE+STAR	12:69353500:+	14:21778986:+	ENSG00000090382
	AC068647.2	AADAC	no_protein,adjacent,pseudogene,1K<gap<10K,readthrough	0	4	3	37	BOWTIE+BOWTIE2;BOWTIE+STAR	3:151803925:+	3:151814139:+	ENSG00000250271
	NDUFB5	TRIM72	m2	3	4	3	38	BOWTIE+STAR	3:179627288:+	16:31230170:+	ENSG00000136521
	KRT8	AGR2	tumor	0	3	2	20	BOWTIE+STAR	12:52897241:-	7:16830427:-	ENSG00000170421
	NAIP	OCLN	banned,known,bodymap2,hpa,1000genomes,m0,multi,exon-exon	0	2	2	28	BOWTIE	5:70979869:-	5:69534694:+	ENSG00000249437
	SMG1	NPIPB5	banned,known,bodymap2,hpa,m0,multi,exon-exon	0	2	2	30	BOWTIE	16:18858170:-	16:22513522:+	ENSG00000157106
	BPTF	LRRC37A2	banned,known,hpa,m0,multi,exon-exon	0	1	2	29	BOWTIE	17:67826337:+	17:46517362:+	ENSG00000171634
ID5_HGD	LYZ	TRA@	m7	0	19	2	35	BOWTIE+STAR	12:69353586:+	14:21642435:+	ENSG00000090382
	LYZ	TRA@	m7	0	19	2	26	BOWTIE+STAR	12:69353658:+	14:21715808:+	ENSG00000090382
	TRA@	LYZ	m7	0	19	2	29	BOWTIE+STAR	14:22015358:+	12:69353745:+	ENSG09000001070
	TRA@	AC020656.1	no_protein,antisense,m7	0	13	2	26	BOWTIE+STAR	14:21715810:-	12:69353658:-	ENSG09000001072
	LYZ	SLC2A4	m7	0	12	68	38	BOWTIE+BOWTIE2;BOWTIE+STAR	12:69353497:+	17:7287133:+	ENSG00000090382
	CCND3	CEACAM5	cancer,m5	5	9	3	33	BOWTIE+STAR	6:41992465:-	19:41729500:+	ENSG00000112576
	CCND3	CEACAM5	cancer,m5	5	9	2	36	BOWTIE+BOWTIE2	6:41993419:-	19:41729495:+	ENSG00000112576
	CCND3	CEACAM5	cancer,m5	5	9	2	23	BOWTIE+STAR	6:41948333:-	19:41729500:+	ENSG00000112576

	CCND3	CEACAM5	cancer,m5	5	9	2	20	BOWTIE+STAR	6:42036939:-	19:41729500:+	ENSG00000112576
	MOG	DDOST	m6	5	7	4	23	BOWTIE+STAR	6:29672337:+	1:20651804:-	ENSG00000204655
	MOG	DDOST	m6	5	7	3	22	BOWTIE+STAR	6:29672295:+	1:20651838:-	ENSG00000204655
	TMEM231	AC020656.1	no_protein,antisense,m2	2	7	2	33	BOWTIE+STAR	16:75537728:-	12:69353729:-	ENSG00000205084
	AC239800.2	AL137802.1	no_protein,lincrna,m3,exon-exon	35	6	4	29	BOWTIE	1:143736123:+	1:16521564:+	ENSG00000232721
	LYZ	ANXA2		0	5	2	29	BOWTIE+STAR	12:69353497:+	15:60363051:-	ENSG00000090382
	CD24	TSPAN8	oncogene,cancer	0	4	6	22	BOWTIE+STAR	6:106969972:-	12:71237157:-	ENSG00000272398
	LYZ	SLC2A4	m26	0	55	48	38	BOWTIE+BOWTIE2;BOWTIE+STAR	12:69353494:+	17:7287133:+	ENSG00000090382
	AC020656.1	FRY	no_protein,antisense,m2,exon-exon	0	15	6	25	BOWTIE	12:69353743:-	13:32275142:+	ENSG00000257764
	AC020656.1	ZNF7	no_protein,antisense,m2	0	12	3	38	BOWTIE+STAR	12:69353721:-	8:144838728:+	ENSG00000257764
	PIF1	DDOST	m3	10	4	2	27	BOWTIE+STAR	15:64821914:-	1:20651996:-	ENSG00000140451
	CATSPER2	PPIP5K1	banned,known,bodymap2,hpa,1000genomes,m0,multi,10K<gap<100K,exon-exon	8	3	2	28	BOWTIE	15:43664418:-	15:43564165:-	ENSG00000166762
	JCHAIN	ACTB	cancer,m2	0	3	4	33	BOWTIE+STAR	4:70659551:-	7:5527639:-	ENSG00000132465
	JCHAIN	ACTB	cancer,m2	0	3	2	20	BOWTIE+STAR	4:70655959:-	7:5527641:-	ENSG00000132465
	LYZ	RPS10-NUDT3	m2	0	3	43	38	BOWTIE+STAR	12:69353502:+	6:34421010:-	ENSG00000090382
	LYZ	RPS10-NUDT3	m2	0	3	2	22	BOWTIE+STAR	12:69353504:+	6:34345680:-	ENSG00000090382
**ID25_HGD**	LYZ	RPS10-NUDT3	m2	0	3	2	18	BOWTIE+STAR	12:69353495:+	6:34407055:-	ENSG00000090382
	PPIP5K1	CATSPER2	banned,known,bodymap2,hpa,1000genomes,m0,multi,10K<gap<100K,exon-exon	8	3	2	30	BOWTIE	15:43564103:-	15:43649879:-	ENSG00000168781
	PPIP5K1	CATSPER2	banned,known,bodymap2,hpa,1000genomes,m0,multi,10K<gap<100K,exon-exon	8	3	2	25	BOWTIE	15:43564867:-	15:43640496:-	ENSG00000168781
	PPIP5K1	CATSPER2	banned,known,bodymap2,hpa,1000genomes,m0,multi,10K<gap<100K,exon-exon	8	3	2	24	BOWTIE	15:43564867:-	15:43648063:-	ENSG00000168781
	RMND5A	ANAPC1	banned,known,bodymap2,hpa,m0,multi,exon-exon	0	3	2	29	BOWTIE	2:86741069:+	2:111822600:-	ENSG00000153561
	SMG1	NPIPB5	banned,known,bodymap2,hpa,m0,multi,exon-exon	0	1	4	29	BOWTIE	16:18858170:-	16:22513522:+	ENSG00000157106
	LYZ	SLC2A4	m25	0	47	108	38	BOWTIE+STAR	12:69353497:+	17:7287135:+	ENSG00000090382
	LYZ	TRA@	m5	0	21	3	29	BOWTIE+STAR	12:69353658:+	14:21753608:+	ENSG00000090382
	AC020656.1	ZNF7	no_protein,antisense,m3	0	19	2	33	BOWTIE+STAR	12:69353721:-	8:144838728:+	ENSG00000257764
	ZNF7	AC020656.1	no_protein,antisense,m3	0	19	2	33	BOWTIE+STAR	8:144838787:+	12:69353661:-	ENSG00000147789
	LYZ	TRA@	m3	0	15	2	38	BOWTIE+STAR	12:69353657:+	14:21804444:-	ENSG00000090382
	KCNE3	ALKBH4	m5	19	12	2	19	BOWTIE+STAR	11:74456348:-	7:102460337:-	ENSG00000175538
	TRA@	AC020656.1	no_protein,antisense,m5	0	12	2	18	BOWTIE+STAR	14:21788391:-	12:69354225:-	ENSG09000001072
	TRA@	AC020656.1	no_protein,antisense,m2	0	10	2	34	BOWTIE+STAR	14:21804440:+	12:69353661:-	ENSG09000001070
**ID26_HGD**	AC020656.1	YIPF5	no_protein,antisense,m2	0	9	3	31	BOWTIE+STAR	12:69353737:-	5:144159956:-	ENSG00000257764
	RNF41	AC020656.1	no_protein,antisense,m2	0	8	2	34	BOWTIE+STAR	12:56209624:-	12:69353654:-	ENSG00000181852
	COQ8B	NUMBL	banned,known,adjacent,healthy,bodymap2,hpa,gap<1K,readthrough	0	5	2	21	BOWTIE+STAR	19:40691592:-	19:40686995:-	ENSG00000123815
	SARNP	AC073063.1	no_protein,pseudogene,cancer,m2	32	4	2	31	BOWTIE+STAR	12:55800593:-	7:99443293:-	ENSG00000205323
	IGK@	GPRC5A	tumor,m2	0	3	2	23	BOWTIE+STAR	2:89989628:+	12:12917260:+	ENSG09000001012
	LYZ	CALR	m2	4	3	10	37	BOWTIE+STAR	12:69353649:+	19:12943246:+	ENSG00000090382
	LYZ	RPS24	ribosomal,m2	0	3	2	34	BOWTIE+BOWTIE2	12:69353513:+	10:78036328:+	ENSG00000090382
	RPS24	LYZ	ribosomal,m2	0	3	4	32	BOWTIE+STAR	10:78055958:+	12:69353696:+	ENSG00000138326
	LYZ	SLC2A4	m28	0	42	10	34	BOWTIE+STAR	12:69353497:+	17:7287125:+	ENSG00000090382
	PRIM1	NACA	banned,known,adjacent,conjoing,healthy,bodymap2,hpa,1000genomes,cancer,m7,gap<1K,readthrough	0	8	4	34	BOWTIE+STAR	12:56731608:-	12:56731607:-	ENSG00000198056
	LYZ	TRA@	m5	0	6	2	36	BOWTIE+STAR	12:69353646:+	14:21568870:+	ENSG00000090382
**ID39_HGD**	EIF3E	RSPO2	known,oncogene,cosmic,cgp,chimerdb3kb,chimerdb3pub,cancer,100K<gap<200K,exon-exon	0	3	2	30	BOWTIE	8:108248613:-	8:108082807:-	ENSG00000104408
	RRM2	C2ORF48	banned,known,no_protein,adjacent,lincrna,conjoing,cacg,non_tumor_cells,hpa,1000genomes,1K<gap<10K,readthrough,exon-exon	0	2	3	28	BOWTIE	2:10129154:+	2:10141854:+	ENSG00000171848
	COQ8B	NUMBL	banned,known,adjacent,healthy,bodymap2,hpa,gap<1K,readthrough,exon-exon	0	1	2	30	BOWTIE	19:40692951:-	19:40686995:-	ENSG00000123815
	LYZ	SLC2A4	m7	0	16	86	38	BOWTIE+STAR	12:69353497:+	17:7287135:+	ENSG00000090382
	AC004923.1	AC068587.6	no_protein,lincrna,m5,exon-exon	31	7	2	27	BOWTIE	11:67955482:-	8:12581044:-	ENSG00000254610
	AC068587.6	AC004923.1	no_protein,lincrna,m5,exon-exon	31	7	2	27	BOWTIE	8:12659130:-	11:67936991:-	ENSG00000283674
	RPL22	MECOM	banned,known,ribosomal,oncogene,chimerdb2,cgp,bodymap2,hpa,18cancers,chimerdb3kb,chimerdb3seq,cancer,tumor,m0,multi	0	4	15	37	BOWTIE+STAR	1:6186507:-	3:169483506:-	ENSG00000116251

ID18_NDB	RPL22	MECOM	banned,known,ribosomal,oncogene,chimerdb2,cgp,bodymap2,hpa,18cancers,chimerdb3kb,chimerdb3seq,cancer,tumor,m0,multi	0	4	13	29	BOWTIE+STAR	1:6197732:-	3:169484014:-	ENSG00000116251
	RPL22	MECOM	banned,known,ribosomal,oncogene,chimerdb2,cgp,bodymap2,hpa,18cancers,chimerdb3kb,chimerdb3seq,cancer,tumor,m0,multi	0	4	4	22	BOWTIE+STAR	1:6186797:-	3:169483796:-	ENSG00000116251
	RPL22	MECOM	banned,known,ribosomal,oncogene,chimerdb2,cgp,bodymap2,hpa,18cancers,chimerdb3kb,chimerdb3seq,cancer,tumor,m0,multi	0	4	2	24	BOWTIE+STAR	1:6197660:-	3:169483945:-	ENSG00000116251
	AC009094.1	NOP58	no_protein,pseudogene	25	3	4	38	BOWTIE+STAR	16:60024605:-	2:202303410:+	ENSG00000261278
	CBX3	C15ORF57	banned,known,healthy,tcga,hpa,gtex,gliomas,chimerdb3pub,chimerdb3seq,oesophagus	0	3	2	26	BOWTIE+STAR	7:26212581:+	15:40563710:-	ENSG00000122565
	KRT8	EPCAM	oncogene,cancer	0	3	2	18	BOWTIE+STAR	12:52897239:-	2:47382993:+	ENSG00000170421
	NOP58	AC009094.1	no_protein,pseudogene	25	3	3	37	BOWTIE+STAR	2:202300342:+	16:60024793:-	ENSG00000055044
	AZGP1	GJC3	banned,known,adjacent,healthy,cacg,bodymap2,hpa,10K<gap<100K,readthrough,exon-exon	0	1	2	19	BOWTIE	7:99971746:-	7:99923603:-	ENSG00000160862
ID1_NDB	DNAJC25	LHX2	exon-exon	0	18	10	30	BOWTIE	9:111631743:+	9:124013961:+	ENSG00000059769
	LYZ	SLC2A4	m9	0	17	6	32	BOWTIE+STAR	12:69353497:+	17:7287125:+	ENSG00000090382
	DNAJC25-GNG10	LHX2	exon-exon	0	16	3	19	BOWTIE+STAR	9:111631743:+	9:124013961:+	ENSG00000244115
	SMG1	NPIPB5	banned,known,bodymap2,hpa,m0,multi	0	12	2	35	BOWTIE+STAR	16:18858211:-	16:22513522:+	ENSG00000157106
	SMG1	NPIPB5	banned,known,bodymap2,hpa,m0,multi	0	12	2	27	BOWTIE+STAR	16:18858171:-	16:22507337:+	ENSG00000157106
	AC068647.2	AADAC	no_protein,adjacent,pseudogene,1K<gap<10K,readthrough	0	3	2	22	BOWTIE+STAR	3:151808240:+	3:151814139:+	ENSG00000250271
	GPRC5A	IGK@	tumor,m2	0	3	2	37	BOWTIE+STAR	12:12914761:+	2:90277750:+	ENSG00000013588
	KRT8	ACTB	cancer,m2	0	3	3	37	BOWTIE+BOWTIE2	12:52922185:-	7:5527623:-	ENSG00000170421
	KRT8	ACTB	cancer,m2	0	3	2	21	BOWTIE+STAR	12:52932810:-	7:5527639:-	ENSG00000170421
	KRT8	ACTB	cancer,m2	0	3	2	19	BOWTIE+STAR	12:52914234:-	7:5527639:-	ENSG00000170421
	GOLT1A	KISS1	banned,known,adjacent,healthy,hpa,18cancers,1K<gap<10K,readthrough,exon-exon	0	2	2	29	BOWTIE	1:204213882:-	1:204192914:-	ENSG00000174567
	NAIP	OCLN	banned,known,bodymap2,hpa,1000genomes,m0,multi,exon-exon	0	2	2	21	BOWTIE	5:70979869:-	5:69534694:+	ENSG00000249437
ID19_NDB	LYZ	SLC2A4	m6	0	12	70	38	BOWTIE+STAR	12:69353497:+	17:7287133:+	ENSG00000090382
	KMT5A	SETD8P1	no_protein,pseudogene	43	4	2	37	BOWTIE+STAR	12:123395252:+	13:18298174:+	ENSG00000183955
	KMT5A	SETD8P1	no_protein,pseudogene	43	4	2	35	BOWTIE+BOWTIE2	12:123389134:+	13:18297592:+	ENSG00000183955
	KMT5A	SETD8P1	no_protein,pseudogene	43	4	2	24	BOWTIE+STAR	12:123389109:+	13:18297564:+	ENSG00000183955
	AC068587.6	CNN3	no_protein,lincrna,m2	0	3	2	37	BOWTIE+STAR	8:12595234:-	1:94906213:-	ENSG00000283674
	CDH17	CEACAM5	oncogene	0	3	2	20	BOWTIE+STAR	8:94216558:-	19:41729500:+	ENSG00000079112
	KRT8	IFI6		0	3	3	28	BOWTIE+STAR	12:52922197:-	1:27666312:-	ENSG00000170421
	KRT8	IFI6		0	3	2	27	BOWTIE+STAR	12:52935385:-	1:27666312:-	ENSG00000170421
	PIGR	TPD52	oncogene,cancer	1	3	5	31	BOWTIE+STAR	1:206929559:-	8:80152793:-	ENSG00000162896
	CFL1	PDIA3	exon-exon	0	2	2	24	BOWTIE	11:65854837:-	15:43746392:+	ENSG00000172757
	GOLM1	SI	exon-exon	0	2	2	30	BOWTIE	9:86084920:-	3:164987226:-	ENSG00000135052
ID22_NDB	LYZ	SLC2A4	m16	0	25	17	37	BOWTIE+STAR	12:69353497:+	17:7287126:+	ENSG00000090382
	CCND3	CEACAM5	cancer,m8	18	17	3	38	BOWTIE+BOWTIE2	6:42034487:-	19:41729495:+	ENSG00000112576
	CCND3	CEACAM5	cancer,m8	18	17	2	35	BOWTIE+BOWTIE2	6:41992465:-	19:41729499:+	ENSG00000112576
	SMG1	NPIPB5	banned,known,bodymap2,hpa,m0,multi	0	5	2	38	BOWTIE+STAR	16:18858211:-	16:22513522:+	ENSG00000157106
	AL022322.2	GALNT4	no_protein,antisense,m2	19	4	4	37	BOWTIE+STAR	22:38134420:+	12:89521262:-	ENSG00000279080
	PIGR	MUC13	cancer	0	3	4	30	BOWTIE+STAR	1:206929571:-	3:124944363:-	ENSG00000162896
	SAR1B	ARL17B	m2	0	3	4	36	BOWTIE+STAR	5:134602888:-	17:46311147:-	ENSG00000152700
	ANXA2	FCGBP	exon-exon	0	2	2	26	BOWTIE	15:60394574:-	19:39886585:-	ENSG00000182718
	FAM171A1	DCLRE1C	exon-exon	0	2	4	26	BOWTIE	10:15370956:-	10:14939869:-	ENSG00000148468
	SCNN1A	TNFRSF1A	banned,known,adjacent,conjoing,healthy,bodymap2,non_tumor_cells,hpa,cancer,1K<gap<10K,readthrough,exon-exon	0	2	2	22	BOWTIE	12:6348727:-	12:6334244:-	ENSG00000111319
	PPIP5K1	CATSPER2	banned,known,bodymap2,ambiguous,hpa,1000genomes,m0,multi,10K<gap<100K,exon-exon	22	1	2	27	BOWTIE	15:43564867:-	15:43648063:-	ENSG00000168781
	LYZ	SLC2A4	m43	1	72	12	35	BOWTIE+STAR	12:69353497:+	17:7287125:+	ENSG00000090382
	LYZ	TRA@	m2	0	8	2	24	BOWTIE+STAR	12:69353502:+	14:21719847:+	ENSG00000090382
	LYZ	TRA@	m2	0	8	2	20	BOWTIE+STAR	12:69353506:+	14:21660632:+	ENSG00000090382
	TRA@	LYZ	m2	0	8	2	35	BOWTIE+STAR	14:21575298:+	12:69353708:+	ENSG09000001070

**ID33_NDB**	PRIM1	NACA	banned,known,adjacent,conjoing,healthy,bodymap2,hpa,1000genomes,cancer,gap<1K,readthrough	0	5	4	32	BOWTIE+STAR	12:56731608:-	12:56731607:-	ENSG00000198056
	AC020656.1	NUPR1	no_protein,antisense	0	4	2	34	BOWTIE+STAR	12:69353766:-	16:28533648:-	ENSG00000257764
	NSF	LRRC37A3	banned,known,hpa,m0,multi,exon-exon	0	1	3	26	BOWTIE	17:46704854:+	17:64892600:-	ENSG00000073969
**ID35_NDB**	LYZ	SLC2A4	m21	0	36	7	33	BOWTIE+STAR	12:69353497:+	17:7287125:+	ENSG00000090382
	KIF13B	EXTL3-AS1	no_protein,antisense	0	9	5	33	BOWTIE+BOWTIE2;BOWTIE+STAR	8:29070319:-	8:28700043:-	ENSG00000197892
	KIF13B	EXTL3-AS1	no_protein,antisense	0	9	3	36	BOWTIE+STAR	8:29068800:-	8:28700525:-	ENSG00000197892
	LINC01972	AC020656.1	no_protein,lincrna,antisense,m5	0	7	2	30	BOWTIE+STAR	3:194765471:-	12:69353707:-	ENSG00000230401
	AC074051.2	AC068587.6	no_protein,lincrna,m3,exon-exon	35	4	3	29	BOWTIE	16:5240105:+	8:12595296:-	ENSG00000260411
	ZNF160	ZNF468	m3	4	4	2	32	BOWTIE+STAR	19:53086054:-	19:52854052:-	ENSG00000170949
	ZNF468	ZNF160	m3	4	4	3	28	BOWTIE+STAR	19:52854084:-	19:53086085:-	ENSG00000204604
	GOLT1A	KISS1	banned,known,adjacent,healthy,hpa,18cancers,1K<gap<10K,readthrough,exon-exon	0	1	2	22	BOWTIE	1:204213882:-	1:204192914:-	ENSG00000174567
	KANSL1	LRRC37A3	banned,known,hpa,non_cancer_tissues,m0,multi,exon-exon	0	1	2	27	BOWTIE	17:46152904:-	17:64869166:-	ENSG00000120071
	NAIP	OCLN	banned,known,bodymap2,hpa,1000genomes,m0,multi,exon-exon	0	1	2	30	BOWTIE	5:70979869:-	5:69534694:+	ENSG00000249437
**ID37_NDB**	LYZ	SLC2A4	m35	0	59	93	38	BOWTIE+BOWTIE2;BOWTIE+STAR	12:69353497:+	17:7287133:+	ENSG00000090382
	LYZ	TRA@	m7	0	18	8	37	BOWTIE+STAR	12:69353656:+	14:21575249:+	ENSG00000090382
	AC020656.1	TRA@	no_protein,antisense,m7	0	12	2	36	BOWTIE+STAR	12:69353766:-	14:21559346:-	ENSG00000257764
	TRA@	AC020656.1	no_protein,antisense,m7	0	12	8	37	BOWTIE+STAR	14:21575254:-	12:69353661:-	ENSG09000001072
	AC020656.1	ZNF7	no_protein,antisense,m3	0	10	7	37	BOWTIE+STAR	12:69353721:-	8:144838728:+	ENSG00000257764
	LYZ	MALT1	oncogene,cancer,m3	41	10	2	37	BOWTIE+STAR	12:69353723:+	18:58684682:+	ENSG00000090382
	KANSL1	ARL17A	banned,known,healthy,bodymap2,hpa,gtex,18cancers,chimerdb3seq,m0,multi,exon-exon	0	3	2	26	BOWTIE	17:46094560:-	17:46570869:-	ENSG00000120071
	KANSL1	ARL17B	banned,known,healthy,bodymap2,hpa,chimerdb3seq,m0,multi,10K<gap<100K,exon-exon	0	3	2	26	BOWTIE	17:46094560:-	17:46352930:-	ENSG00000120071
	SARNP	AC073063.1	no_protein,pseudogene,cancer	23	3	3	30	BOWTIE+STAR	12:55800593:-	7:99443293:-	ENSG00000205323
	WDR70	AL021368.5	no_protein,lincrna,exon-exon	7	3	2	26	BOWTIE	5:37379392:+	6:57961224:-	ENSG00000082068
	AC025279.1	BANP	no_protein,lincrna,oncogene,tumor,exon-exon	7	2	2	21	BOWTIE	16:29326483:+	16:88035323:+	ENSG00000198106
	DUS4L	BCAP29	banned,known,adjacent,healthy,bodymap2,hpa,chimerdb3pub,1K<gap<10K,readthrough,exon-exon	0	2	2	19	BOWTIE	7:107576592:+	7:107580759:+	ENSG00000105865
**ID40_NDB**	*excluded from fusion gene analysis (see 'Methods' section)*										

gene_2_id(3' end_fusion_partner)	exon_1_id(5' end_fusion_partner)	exon_2_id(3' end_fusion_partner)	fusion_sequence	predicted_effect
ENSG00000156110	ENSE00002288595	ENSE00001024568	GCCCGTGGCCGCCGTGCAGGCGGCCGTCAGCAACCTCGTCCGG*TGGATGATTCAACAGCCACACAAAGCAGCAACATTTTTTGGAT	in-frame
ENSG00000233432			ATATGGCTTTAATGTGAATGCAGGCAATGTTATCCAACAGTCAGCTGCAA*AAAAAAGGAGTAAAAATTAAACTTCACAAAATAATTTACCGTCTTGCTGG	CDS(truncated)/exonic(no-known-CDS)
ENSG00000243716	ENSE00001505919	ENSE00002138288	AGGTATACTATGTACCAGAATCAGTTGTTGGAGAAAATTAAAG*GTATGATCTCGTGAAATCTTGAGAGAAACTGAATGACGAATGA	CDS(truncated)/UTR
ENSG00000243716			TTACGTTCATGTAAACAGCATGACGTGAGGCCATGGATGCAGGCATTAAG*GTATGATCTCGTGAAATCTTGAGAGAAACTGAATGACGAATGAAACTATT	CDS(truncated)/UTR
ENSG00000185829	ENSE00003662350	ENSE00003806456	TCGTCAGCAAACAGACATTTACAAACAGATACGTGCTAATAAG*GTTTCTGTGTGGAGACAGTAGAATATAAAAATAACACCTTCGC	out-of-frame
ENSG00000228696	ENSE00003662350	ENSE00002370389	TCGTCAGCAAACAGACATTTACAAACAGATACGTGCTAATAAG*GTTTCTGTGTGGAGACAGTAGAATATAAAAATAACACCTTCGC	out-of-frame
ENSG00000163209			CCTTCACCAGATCCCAGAGGCTGAACACCTCGACCTTCTCTGCACAGCAG*GTCCAGCATCCTTTGAAGCATGAGTTCTTACCAGCAGAAGCAGACCTTTA	intergenic/UTR
ENSG00000135046	ENSE00001518181	ENSE00003569681	AGTGATTCTCCTGCCTCAGCCTCCTGAGTAGCTGGGATTACAG*ACACTTTTTCAAAAATGGCAATGGTATCAGAATTCCTCAAGCA	UTR/CDS(no-known-start-or-end)
ENSG00000197822	ENSE00003606270	ENSE00003560198	TCATTTTTCAAGACTTTGAATGATGACAGCGTGGTGGAAATTG*GTTAAAAATGTGTCTGCAGGCACACAGGACGTGCCTTCACCCC	in-frame
ENSG00000279081			TTAGGTGGCTGTTGACATGGAATTTGCTAAGAATATGTATGAACTGCATA*AAAAAAGTTTCTCCAAATGAGCTCATCTTGGGGTGGTACGCTGCCGGCCA	CDS(truncated)/exonic(no-known-CDS)
ENSG00000271573			GCAAGTCGACTCCTTCCAGGCTCCAGGAACACCACAAAGCAATATGAAAC*CTGTTCATGAGAGGAGTCAGGAATGCCTTCCACCAAAGAAACGAGACCTC	CDS(truncated)/exonic(no-known-CDS)
ENSG00000135046	ENSE00001548454	ENSE00001472097	TGCATGTTTCCTGCTCTTCCCTCATTAAATTGCTTTTAATTCC*AAAAAAAAAAAAAAAAAAAAAAAGCAATCATTAAGCTTTTCATC	UTR/UTR
ENSG00000067225			CTTCTCTACCCCAAAGAAAAGATTATTCAATAAAGTTTCCTGCCTTTC*CAAACATAAAAAAAAAAACAAAAAAATCGGTAGAGAGTGATTTCTCTCCC	UTR/intronic
ENSG00000197822	ENSE00002214653	ENSE00003560198	TCCCTGGAATCTCTTGAAGTCTCAGGGACAATCCAGTCACAAG*GTTAAAAATGTGTCTGCAGGCACACAGGACGTGCCTTCACCCC	out-of-frame
ENSG00000168970			GCCTCAGCCTCCCGAGTAGCTGGGACTACAGGTGCGTGCCACCTTGCCCT*GCTAATTTTTTTGTATTTTTAGTAGAGACGGGGTTTCACCGTGTTAGCCA	UTR/UTR
ENSG00000135046	ENSE00003470911	ENSE00003569681	TCAAGCAATTCTGCCTCAGCCTCCCAAGTAGCTGGGACTACAG*ACACTTTTTCAAAAATGGCAATGGTATCAGAATTCCTCAAGCA	UTR/CDS(no-known-start-or-end)
ENSG00000149798	ENSE00003465841	ENSE00000992560	AGATGTCCTCATCATCCCGTCAGAGCTGAGGTACTTCGTGAAG*CTCCTCAGCCCTGGACCGGGGACAAGTAACCCTCGGTGACAAG	CDS(truncated)/UTR
ENSG00000075624			GGTTTTGTTTTGGGTTTTTTTTTGTTTTGTTTTTTTTTTTTGTTTTTG*TTTTTTTTTTTTTTTGGCTTGACTCAGGATTTAAAAACTGGAACGGTGA	intronic/UTR
ENSG00000243716			TGGGTAAGCGATTCTCCTGCCTCAGCCTCCCGAGTAGCTGGGATTACAGA*GCTTGAAATGAATTTTAAAGGATGACTGATGGTCCTTGGAAGAGAAACAG	intronic/UTR
ENSG00000124942			CTGTGTACATAGGTTTATCTCCTATTGGAATATTTTCTTTATATAGGCG*TTTTTTTTTTTTTTCTTTTTTTTGGGAGACAGAGTCTCGCTTTGTCACCC	UTR/intronic
ENSG00000156011	ENSE00002111832	ENSE00003584316	GGGGCTCCGCCGCACCCAGCGCTAGGGCCCAGGCCGGGCCGCG*GAGGAGCAACTGAAGTCACATGAAAGTAAGCTGAAGCAGATCA	exonic(no-known-CDS)/CDS(truncated)
ENSG00000170423	ENSE00002555095	ENSE00001659005	AGTGATTCTCCAGCCTCACCCTCCCGAGTAGCTGGGATTACAG*AACGCCAGCCTGCAGGCCGCCATCACTGATGCTGAGCAGCGTG	UTR/CDS(truncated)
ENSG00000238083	ENSE00002689718	ENSE00002373231	GAAAGCAGCTTCAGGAGCCATAGTACCTACAGCAGCACTCCAG*AAATTTCCAAGGAAACTATATTTCTTACATTGATGGAAATGTA	out-of-frame
ENSG00000073910			GGTTATTTCTTTGATAAGAACTGAATGTGGCCGGGCGCAGTGGCTCACGC*CTGTAATCCCAGCACTTCGGGAGGCCGAGGCGGGTGGATCACGAGGTCAG	exonic(no-known-CDS)/intronic
ENSG00000073910	ENSE00002336888	ENSE00003270899	CCGGGCGCAGTGGCTCACGCCTGTAATCCCAGCACTTTGGGAG*GCCGAGGTGGGCGGATCACAAGGTCAGGAGATCGAGACCATCC	exonic(no-known-CDS)/UTR
ENSG00000274611	ENSE00003785912	ENSE00003731868	AACATGTATTTAACCAAGTTCCAACAAAAGAATGCTGCCACAG*GATGGACGTGGTAGAGGTCGCGGGCAGTTGGTGGGCACAAGAG	CDS(complete)/UTR
ENSG00000147789			CACGCCTGTAATCCCAGCACTTTGGGAGGCCGAGGTGGGTGGATCACAAG*GTCAGGAGATCGAGACCATCCTGGCCAACACGGTGAAACCCTGTGTCTAC	intronic/UTR
ENSG09000001070			AATTTACCTAAAACCTTGGTTATCAAATACATCTCCAGTACATTCCGTTC*TTTTTTTTTTTTGAGACAGTCTCGCTCTTGTCGCCCAGGCTGGAGTGCAG	UTR/---
ENSG09000001070			CCTCCCGAGTAGCTGGGATTACGGGCGCCCGCCACCACGCCCGGCTAATT*TTTTGTATTTTTAGTAGAGACGGTGTTTCACCATGTTGGTCAGGCTGAT	UTR/---
ENSG09000001070			TCCCGAGTAGCTGGGATTACGGGCGCCCGCCACCACGCCCGGCTAATTTT*TTTGTATTTTTAGTAGAGACAGGGTTTAACCACGTTGGCCAGGCTGGTCT	UTR/---
ENSG09000001070			TTACCTAAAACCTTGGTTATCAAATACATCTCCAGTACATTCCGTTCTTT*TTTTTTGAGACAGACTCTCACTCTGTCACCCAGGCTCGAGTGCAGTGGC	UTR/---
ENSG00000114771			AAGAAGCTTATTTTATAACCATGGTGGTGGCTCATGTTTGAGTAGTAATG*AGACCAAGAAGCGGGACGTTCACCATGGGAAGAAAATCGCTGTACCTTCT	exonic(no-known-CDS)/UTR
ENSG00000177238			CAGTTGTACGTAAGAAAACCCAATTCCCCCTGAGAAAGAGAAAGAGCTGG*AGTCCTTTAAAAATTAACTGCCTGTTTTTCTGTGGATAGTGAGCCTTATC	UTR/UTR
ENSG00000106541			TTACTACCTGGGGACCCCCCTTGCCCATGCCTCCAGCTACAAAACAATTC*AATTTCTTTTTTTTTTTTTGAGACGGAGTCTCACTCTGTCGCCCAGGCTG	UTR/intronic
ENSG00000197822	ENSE00003590701	ENSE00003560198	TTATTGATCCAAATTTCAGCTGAGTATGATCCTTCCAAACTAG*GTTAAAAATGTGTCTGCAGGCACACAGGACGTGCCTTCACCCC	out-of-frame
ENSG00000243716	ENSE00001505919	ENSE00002138288	AGGTATACTATGTACCAGAATCAGTTGTTGGAGAAAATTAAAG*GTATGATCTCGTGAAATCTTGAGAGAAACTGAATGACGAATGA	CDS(truncated)/UTR
ENSG00000238083	ENSE00002689718	ENSE00002373231	GAAAGCAGCTTCAGGAGCCATAGTACCTACAGCAGCACTCCAG*AAATTTCCAAGGAAACTATATTTCTTACATTGATGGAAATGTA	out-of-frame
ENSG09000001070			GGAGTGCAGTGGCGCAATCTCGGCTCACTGCAACCTCCACCTCCCGGGTT*CCGCCATTCTCCTGCCTCAGCCTCTCGAGTAGCTGGGACTACAGGCGCCC	UTR/---
ENSG09000001070			CTCCCGAGTAGCTGGGATTACGGGCGCCCGCCACCACGCCCGGCTAATTT*TTTGTATTTTTAGTAGAGACAGGGTTTAACCACGTTGGCCAGGCTGGTC	UTR/---
ENSG00000090382			GTTGGCCAGGCTGATCTCGAATTCCTGACCTTGTGATCCACCCACCTCGG*CCCAAAGTGCTGGGATTACAGGCGTGAGCCACTGCGCCCGGCCACATTCA	---/UTR
ENSG00000257764			GACCAGCCTGGCCAACGTGGTTAAACCCTGTCTCTACTAAAAATACA*AAATTAGCCGGGCGTGGTGGCGGGCGCCCGTAATCCCAGCTACTCGGGAG	---/intronic
ENSG00000181856			AATTTACCTAAAACCTTGGTTATCAAATACATCTCCAGTACATTCCGTTC*TTTTTTTTTTTTGAGACAGTCTCGCTCTGTCGCCCAGGCTCGAGTGCAG	UTR/UTR
ENSG00000105388			CTCCAGCCTGGCACAGAGCAAGACTCCATCTAAAAAAAAAAAAAAAA*AAGACTCTGACCTGTACTCTTGAATACAAGTTTCTGATACCACTGCACTG	intronic/UTR
ENSG00000105388			TGCACTCCAGCCTGGGCAACAGAGCAAGACTCCATCTCAAAAAAAAAAA*AAAAGAAGACTCTGACCTGTACTCTTGAATACAAGTTTCTGATACCACTG	intronic/UTR
ENSG00000105388			CCTGGGTGACAGAGTGAGACCCTGTCTCAAAAAAAAAAACAAAAAAAAAC*AAGACTCTGACCTGTACTCTTGAATACAAGTTTCTGATACCACTGCACTG	intronic/UTR

ENSG00000105388			CCAGCCTGGGTGACAGAGCAAGAGTCTGTCTCAAAAAAACAAAACAAAAC*AAGACTCTGACCTGTACTCTTGAATACAAGTTTCTGATACCACTGCACTG	intronic/UTR
ENSG00000244038			AAAAATAAATAAATAAATAAATAAATAAATAAATAAATAAATAAATAAAA*AAAAATAAAGCGAGATGTTGCCCTCAAACTTCACCTGG	UTR/UTR
ENSG00000244038			CCACTGCACTCTAGCCGGAGTGACAGAGTAAGACTCTGTCTCAAAAATAA*ATAAATAAATAAATAAATAAATAAATAAATAAATAAAAATAAAGCGAGAT	UTR/UTR
ENSG00000257764			AGCGGTGGCTCACGCCTGTAATCCCAGCACTTTGGGAGGCCGAGGTGGGT*GATCACAAGGTCAGGAGATCGAGACCATCCTGGCTAACACGGTGAAACCC	UTR/intronic
ENSG00000224174	ENSE00001605525	ENSE00001677405	GGACGACTGAGCAAGGCTTTGGAAAACCAGAGAGATTAGAGCG*AATGGGGAAATGGAGAGAGAACCTGAAAGAGCCCCAAACTCGA	exonic(no-known-CDS)/exonic(no-known-CDS)
ENSG00000182718			AATTTACCTAAAACCTTGGTTATCAAATACATCTCCAGTACATTCCGTTC*TTTTTTTTTTTTTTTTTTTTTTGTGACAGGGTCTTGCTCTGTCACCC	UTR/intronic
ENSG00000127324			AAACAGATCGAACAGTTTTGAAGCTACTGTGTGTGTGAATGAACACTCTT*TTGATTTATTCTAGAATGCTGGCCAGTCTGTTTCTGGAGAGTAGGGACCT	UTR/intronic
ENSG00000181856			AATTTACCTAAAACCTTGGTTATCAAATACATCTCCAGTACATTCCG*TTTTTTTTTTTGAGACAGTCTCGCTCTGTCGCCCAGGCTCGAGTGCAGT	UTR/UTR
ENSG00000073910	ENSE00002336888	ENSE00003270899	CCGGGCGCAGTGGCTCACGCCTGTAATCCCAGCACTTTGGGAG*GCCGAGGTGGGCGGATCACAAGGTCAGGAGATCGAGACCATCC	exonic(no-known-CDS)/UTR
ENSG00000147789			CACGCCTGTAATCCCAGCACTTTGGGAGGCCGAGGTGGGTGGATCACAAG*GTCAGGAGATCGAGACCATCCTGGCCAACACGGTGAAACCCTGTGTCTAC	intronic/UTR
ENSG00000244038			TCAAGACTATCCTGGCTAACACGGTAAAACCCTGTCTCTACTAAAAATAC*AAAATTAGCTGGGCGTGGTGGTGGGCACCTGTAGTCCCAGCTACTCGGGA	UTR/UTR
ENSG00000168781	ENSE00003653606	ENSE00001774689	CATGGCAAAAAGCTACCACCTGCCAGTCTGAAGCACCGAGATG*AGCTCTTGTTTGTCCCGGCCGTAAAACGATTTTCTGTGTCGTT	UTR/CDS(truncated)
ENSG00000075624			CATGAGCCACCGTGCCTGGCTTTTTTTTTTTTTTTTTTTTTTTTTTTTTTT*TTTTTTTTTTTTTTTTGGCTTGACTCAGGATTTAAAAACTGGAACGGTGA	intronic/UTR
ENSG00000075624			GGACTGAAAATCAGGAGGTGTAATAAAATGTTCCTCGCATTCCCCCCCGC*GGTTTTTTTTTTTTTTTTGGCTTGACTCAGGATTTAAAAACTGGAACGGT	UTR/UTR
ENSG00000270800			ACCTAAAACCTTGGTTATCAAATACATCTCCAGTACATTCCGTTCTTTTT*TTTTTGAGACAGTCTCGCTCTGTCACCCAGGCTGGAGTGCAGTGGCAAGA	UTR/intronic
ENSG00000270800			TAAAACCTTGGTTATCAAATACATCTCCAGTACATTCCGTTCTTTTTTT*TTTTTTTTTTTTTTTTTTGGGACGGAGTCTCGCTCTGTCGCCCATGCTAG	UTR/intronic
ENSG00000270800			AAAATTTACCTAAAACCTTGGTTATCAAATACATCTCCAGTACATTCCGT*TCTTTTTTTTTTTTTGAGACAGGATCTTGCTCTGTCACCCAGGCTGGAGT	UTR/intronic
ENSG00000166762	ENSE00001774689	ENSE00001581568	GTAAAACGATTTTCTGTGTCGTTTGCAAAGCATCCGACTAACG*AGGTATCTGCCGGTACTGAGATGTAAGTGATCAGCTGAAGGGA	CDS(truncated)/UTR
ENSG00000166762	ENSE00003788646	ENSE00003500886	CATGGCAAAAAGCTACCACCTGCCAGTCTGAAGCACCGAGATG*AATTGCTGGAATCCACAAATACCAAACTATGGCCATTGAAGCT	in-frame
ENSG00000166762	ENSE00003788646	ENSE00003527303	CATGGCAAAAAGCTACCACCTGCCAGTCTGAAGCACCGAGATG*ACATGGCCGCTTACCAACAAGAAGAGCAGATGCAGCTTCCCCG	in-frame
ENSG00000153107	ENSE00002531730	ENSE00002441208	CATCCACAGCAGTGTTTCTCGGGTTGGAAAAGCCATTGATAAG*GATTCACTTTAAGAGATTTGGAAACTCTTCCCTTTGGAATTGC	out-of-frame
ENSG00000243716	ENSE00001505919	ENSE00002138288	AGGTATACTATGTACCAGAATCAGTTGTTGGAGAAAATTAAAG*GTATGATCTCGTGAAATCTTGAGAGAAACTGAATGACGAATGA	CDS(truncated)/UTR
ENSG00000181856			AATTTACCTAAAACCTTGGTTATCAAATACATCTCCAGTACATTCCGTTC*TTTTTTTTTTGAGACAGTCTCGCTCTGTCGCCCAGGCTCGAGTGCAGTGG	UTR/UTR
ENSG09000001070			CTCCCGAGTAGCTGGGATTACGGGCGCCCGCCACCACGCCCGGCTAATTT*TTTTGTATTTTTGGTAGAGACAGGGTTTCTCCATGTTGGCCAGGCTGGTC	UTR/---
ENSG00000147789			CACGCCTGTAATCCCAGCACTTTGGGAGGCCGAGGTGGGTGGATCACAAG*GTCAGGAGATCGAGACCATCCTGGCCAACACGGTGAAACCCTGTGTCTAC	intronic/UTR
ENSG00000257764			CGAGACCATCCTGGCCAACACGGTGAAACCCTGTGTCTACTAAAAATACA*AAAAAATTAGCCGGGCGTGGTGGCGGGCGCCCGTAATCCCAGCTACTCGG	UTR/intronic
ENSG09000001072			CCTCCCGAGTAGCTGGGATTACGGGCGCCCGCCACCACGCCCGGCTAATT*TTTTTGTATTTTTAGTGGAGACGGGGTTTCACCGTGTTAGCCAGGATGGT	UTR/---
ENSG00000160993			GCAGTCTTTTCAGTTTTGACTGAGTGATGTGAGAAACTTCTTTTCTTTTC*TTTTTTTTTTTTTGAGACAGGGTCTTGCTTTGTTGTCCAGGCTGGAGTG	UTR/intronic
ENSG00000257764			GCTCTTTAGTTTAATTAGATCCATTTTGTCAATTTTTTGTTTTTGTTGCA*AAGCCTTTAATTAGAATGTTTGTATTTTTTACATCATGCATAACTTCACA	---/exonic(no-known-CDS)
ENSG00000257764			TGAGACCATCCTGGCTAACACGGTGAAACCCCGTCTCCACTAAAAATACA*AAAAAATTAGCCGGGCGTGGTGGCGGGCGCCCGTAATCCCAGCTACTCGG	---/intronic
ENSG00000145817			GCCGGGCGCAGTGGCTCACGCCTGTAATCCCAGCACTTTGGGAGGCCGAG*GCGGGCGGATCACAAGGGCAGGAGATCGAGACCATCCTGGTTAACACGGT	intronic/UTR
ENSG00000257764			CATCCTGGCTAACACATTGAAACCCCGTCTCTACTAAAAATACAAAAAAC*TAGCCGGGCGTGGTGGCGGGCGCCCGTAATCCCAGCTACTCGGGAGGCTG	intronic/intronic
ENSG00000105245			CTCCAACAGCTGCCTTCTCCCGGGTTCCAGCCTCTCAGTGTGTTGGAGAG*GGCGGACCCCGGAGGCCTGAGCGGCACCTGCCCCCAGCCCCCTGTGGGGC	UTR/CDS(truncated)
ENSG00000228335			CATTTGGTTTTAGGAAGAAGAAACAAAGCCCATTGAGCTCCCTGTCAAAG*AGGAAGAACCCCCTGAAAAAAACTGTTGATGTGGCAGCAGAGAAGAAAGT	CDS(truncated)/exonic(no-known-CDS)
ENSG00000013588			CTTGTTTAAGGCTGAATTATATTCCATTGTGTGTGTGTGTGTGTGTGTGC*GCGTGTATGTGCGCCTGACCCTGATTTCTGCAACCTCCAGATTTCTTTCT	---/UTR
ENSG00000179218			TGCCTCAGCCTCCCGAGTAGCTGGGATTACGGGCGCCCGCCACCACGCCC*GGCTGATTTTTTGTATTTTTAGTAGAGACGGGGTTTCACCGTGTTAGCCA	UTR/UTR
ENSG00000138326			TGGTTATCAAATACATCTCCAGTACATTCCGTTCTTTTTTTTTTGAGAC*AGTTTAGCTCTGTCGCCCAGGCTGGAGTGCAGTGGTACAATCTCCTTGGC	UTR/intronic
ENSG00000090382			CGCCTGGCTAATTTTTGTATTTTTAGTAGAGACGGGGTTTCACCGTGTTG*GCCAGGATGGTCTCGATCTCCTGACCTTGTGATCCACCCACCTCGGCCTC	UTR/UTR
ENSG00000181856			AATTTACCTAAAACCTTGGTTATCAAATACATCTCCAGTACATTCCGTTC*TTTTTTTTTTTTTTTTTTTTGAGACAGTCTCGCTCTGTCGCCCAGGCTC	UTR/UTR
ENSG00000196531			CTTCAAGAGCCATTTAATAAATATGGCAGAACTATATATGTGTCTTAAAC*CTCAAAGTAAATTTTCCTTGAGAAATAATTTATGTTGAAAAGATTTCCTG	UTR/UTR
ENSG09000001070			TCCTGCCTCAGCCTCCCGAGTAGCTGGGATTACGGGCGCCCGCCACCACG*CCCGGCTAATTTTTTTGTATTTTTAGTAGAGACGGGGTTTCACCGTGGTC	UTR/---
ENSG00000147655	ENSE00002112612	ENSE00001378581	GCATCTAGTCTTTCCGCTTCTTGAATTTCTCTCTGTAAAGGAG*GTTCGTGGCGGAGAGATGCTGATCGCGCTGAACTGACCGGTGC	CDS(truncated)/UTR
ENSG00000163009	ENSE00003659027	ENSE00001489842	GTTTGTGGCAGACAGACTTATGCTGGAACTGGGTTTTAGCAAG*GTGCTGGGAGACCGTGAAGTGCAAAGCAGATGGAGTCCAGGCC	CDS(truncated)/exonic(no-known-CDS)
ENSG00000105245	ENSE00003529650	ENSE00003482506	GAAGTCCAGGGACCTCAAATTCCTCACAGGCTTTGAAACCAAG*GGCGGACCCCGGAGGCCTGAGCGGCACCTGCCCCCAGCCCCCT	in-frame
ENSG00000181856			AATTTACCTAAAACCTTGGTTATCAAATACATCTCCAGTACATTCCGTTC*TTTTTTTTTTGAGACAGTCTCGCTCTGTCGCCCAGGCTCGAGTGCAGT	UTR/UTR
ENSG00000283674	ENSE00003779619	ENSE00003799182	CAGGAAGGAGACCGGCACCCAGACAGGTGGCGACGGCAGAGGA*GAAGGAAAATACATGTACAGCCAACTTCCTTGAGGGTTCGTTC	exonic(no-known-CDS)/exonic(no-known-CDS)
ENSG00000254610	ENSE00003792578	ENSE00002156751	CAAGGAGGACACCGGCACCCAGACAGGTGGCGACGGCAGAGGA*GAAGGAAAATACATGTACAGCCAACTTCCTTGAGGATTCGTTC	exonic(no-known-CDS)/exonic(no-known-CDS)
ENSG00000085276			TCAAAGGGCTTCCCTTGGTTCGCCACTCATTTATTTGTAACTTGACTTCT*TTTTTTTTTCTGCTTAAAAATTTCAATTCTCGTGGTAATACCAGAGTAGA	UTR/intronic

ENSG00000085276			TGTTATCATTTGTGTATTTTCTTAGAAAAAGCTTGTGGTGAAGGGGGGCA*AAAAAAAAGAAGCAAGTTCTGAAGTTCACTCTTGATTGCACCCACCCTGT	CDS(truncated)/intronic
ENSG00000085276			TAGTGGAGTTCTTGTGTCCTTTCTCTGCAGGTATTTGAAATATCTCACCA*AAAAAATATTTGAAGAAGAATAATCTACGTGACTGGTTGCGCGTAGTTGC	CDS(truncated)/intronic
ENSG00000085276			TCACTCTTGATTGCACCCACCCTGTAGAAGATGGAATCATGGATGCTG*ATTTTGAGCAGTTTTTGCAAGAAAGGATCAAAGTGAACGGAAAAGCTGGG	CDS(truncated)/intronic
ENSG00000055044			CCATGTACCAGCACAACAATTCCTAGTCCAGAGAAAAAGAAGAAAAAGA*AAAAAAAAGAGAGAGAACGAGGATTAACAGAAAGGAATTACGATTATATC	exonic(no-known-CDS)/CDS(truncated)
ENSG00000128891			ACTTGTGTTCTCATGAGCCTAAGGACCATTCTAGATTTATTACGTGTTTT*TTTGTGTGTGTGTGTGTGTGTGTGTGTGTGTTAAAATATATGTAGCAT	UTR/intronic
ENSG00000119888			ACTACCTGGGGACCCCCCTTGCCCATGCCTCCAGCTACAAAACAATTCAA*TTTCTTTTTTTTTTTTGGAATGGAGTCTCGCTGGCCGGACGTGGTGGCTC	UTR/intronic
ENSG00000261278			GCAAAATAGAACAGGTAGATAAAGAGGATGAAATTACTGAAAAGAAAGCC*AAAAAAAGCCAAGATTAAAGTTAAAGCTGAAGAAGAGGAATAAGAAGAAA	CDS(truncated)/exonic(no-known-CDS)
ENSG00000176402	ENSE00001055051	ENSE00001231504	GAGACCCTGAAAGACATCGTGGAGTATTACAACGACAGTAACG*TTCCAGGAAGAAGCTTAGCCCAGGAAAAACAAAGACCAGTTGG	in-frame
ENSG00000106689	ENSE00001519553	ENSE00003715988	GGAGGCTTTCCTGCTGGTGGCAACCGCCTACGAGACACTCAAG*ACCATGCCGTCCATCAGCAGTGACCGCGCCGCGCTGTGCGCCG	in-frame
ENSG00000181856			AATTTACCTAAAACCTTGGTTATCAAATACATCTCCAGTACATTCCGTTC*TTTTTTTTTTTTTTTTTTTTGAGACAGTCTCGCTCTGTCGCCCAGGCTC	UTR/UTR
ENSG00000106689			GCGCCGAGGAGGCTTTCCTGCTGGTGGCAACCGCCTACGAGACACTCAAG*ACCATGCCGTCCATCAGCAGTGACCGCGCCGCGCTGTGCGCCGGCTGCGG	in-frame
ENSG00000243716			TTACGTTCATGTAAACAGCATGACGTGAGGCCATGGATGCAGGCATTAAG*GTATGATCTCGTGAAATCTTGAGAGAAACTGAATGACGAATGAAACTATT	CDS(truncated)/UTR
ENSG00000243716			AGGCATTAAGGTATACTATGTACCAGAATCAGTTGTTGGAGAAAATTAAA*AGCTTGAAATGAATTTTAAAGGATGACTGATGGTCCTTGGAAGAGAAACA	CDS(truncated)/UTR
ENSG00000114771			CCCATCTGCCATCACTGCTTTGGAGCCCAGGGTAACTCTGCCAGTAACAG*AGACCAAGAAGCGGGACGTTCACCATGGGAAGAAAATCGCTGTACCTTCT	exonic(no-known-CDS)/UTR
ENSG09000001012			TTCAAGCGATTTTCGTGCCTCGGCCTTCTGAGTAGCTGGGATTACAGGCG*TGCGCCACCATGCCTGGCTAATTTTTGTATTTTTAGTAGAGACAGGGTTT	UTR/---
ENSG00000075624			CCCATTTGCTACATTTTTTTTTTTTTTTTTTTTTTTTTTTTTTTTTTTTTTT*GGCTTGACTCAGGATTTAAAAACTGGAACGGTGAAGGTGACAGCAGTCGG	intronic/UTR
ENSG00000075624			TAGCTTTTTTGTAGGTAGGTTCTTCAGGATTTTTTTGTTTTTGTTTTTT*TTTTTTTTTTTTTTTTGGCTTGACTCAGGATTTAAAAACTGGAACGGTGA	intronic/UTR
ENSG00000075624			ATTACAGGCGTGAGCCACTGCACCCGGTGGATTTTTTTTTTGTTTTGTTT*TTTTTTTTTTTTTTTTGGCTTGACTCAGGATTTAAAAACTGGAACGGTGA	intronic/UTR
ENSG00000170498	ENSE00001377126	ENSE00001125940	CCCCAGGCTGAGTGCGGCATGATCTCCATCACCGAATGGCAGA*CCTCAAGGCACTTCTAGGACCTGCCTCTTCTCACCAAGATGAA	CDS(truncated)/UTR
ENSG00000197822	ENSE00003590701	ENSE00003560198	TTATTGATCCAAATTTCAGCTGAGTATGATCCTTCCAAACTAG*GTTAAAAATGTGTCTGCAGGCACACAGGACGTGCCTTCACCCC	out-of-frame
ENSG00000181856			AATTTACCTAAAACCTTGGTTATCAAATACATCTCCAGTACATTCCGTTC*TTTTTTTTTTTTGAGACAGTCTCGCTCTGTCGCCCAGGCTCGAGTGCAGT	UTR/UTR
ENSG00000279730			CAGCCATCGCCAAGCAAGCCCTGAAAAAGCCCATCAAGGGCAAACAG*CCCCCGAAAAAAAGCTCAAGGAAAAACGCAACAGAATCGCAAACACGGAT	CDS(truncated)/exonic(no-known-CDS)
ENSG00000279730			GGGGCGCCGCCGGCCCTTCCCCGGGGCGCCGCGGCGGCGCTGGTGGC*GGCAGCAGCAGCAGCAGCGGCAGCGGCAGCGGTGGTGGCCGGGCAGCGGC	UTR/exonic(no-known-CDS)
ENSG00000279730			CGCCGGCCATGGGGGAAGGGGGCGCCGTGGGGCGCCGCCGGCCCTTCCCC*GGGGCGCCGCGGCGGCGCTGGTGGCGGCGGCAGCAGCAGCAGCAGCGGCA	UTR/exonic(no-known-CDS)
ENSG00000117519			CACCTGTAATCCCAGCACTTTGGGAGGCCGAGGCTGGCGGATCACCTAAG*GTCAGGAGTTCGAGACCAGGCTGGCCAACATGGTGAAACCCCGTCTCTGC	exonic(no-known-CDS)/intronic
ENSG00000105388			AGAGCAGACTTGGGAAGATCTGAGAATTAAAAAAAAAAAAAAAAACAAACC*AAGACTCTGACCTGTACTCTTGAATACAAGTTTCTGATACCACTGCACTG	intronic/UTR
ENSG00000126709			AGTTCTGGCTCTCCCATTTGCTACATTTTTTTTTTTTTTTTTTTTTTTTTT*TTTTTTTTTTTTTTTTTTTTTTTTGAGATGGGTTCTCACTATATTGTCCAG	intronic/UTR
ENSG00000126709			AGGTGTGAGCCACTGTGCCCGGCCTTTTTTTTTTTTTTTTTTTTTTTTTT*TTTTTTTTTTTTTTTTTTTTTTTGAGATGGGTTCTCACTATATTGTCCAG	intronic/UTR
ENSG00000076554			TCACACATAAGAAAATTGAGGTTTGGAAGAGTGAAGCGTTTTTCTTTTTC*TTTTTTTTTTTTTGAGACGGAGTCTCACACTTTCGCCCAGGCTGGAGTGA	UTR/intronic
ENSG00000167004	ENSE00001344370	ENSE00001489318	CACGGCTACTCATGGAAGCAGGACCAGTAAGGGACCTTCGATT*AAAAAAAAAAAAAAAAAAAAAAAAAAAAAAAAAAAAAAAAAAAAA	UTR/UTR
ENSG00000090402	ENSE00001858995	ENSE00000999825	AGTGATTCTTCTGCCTCAGCCTCCCGAGTAGCTAGGACTACAG*TCGACAAAAACACATGAAGCTCATTGTTGCTGCAGATGATAAT	UTR/CDS(truncated)
ENSG00000181856			AATTTACCTAAAACCTTGGTTATCAAATACATCTCCAGTACATTCCGTTC*TTTTTTTTTTTTTTTTTTTTGAGACAGTCTCGCTCTGTCGCCCAGGCT	UTR/UTR
ENSG00000105388			ACTGCACTCCAGCCTGGCAACAGAGCAAGACTCCATCTCAAAAAAAAAAA*AAAAGAAGACTCTGACCTGTACTCTTGAATACAAGTTTCTGATACCACT	intronic/UTR
ENSG00000105388			GCACTCCAGCCTGGCACAGAGCAAGACTCCATCTAAAAAAAAAAAAAAAAA*GAAGACTCTGACCTGTACTCTTGAATACAAGTTTCTGATACCACTGCACT	intronic/UTR
ENSG00000243716			TTACGTTCATGTAAACAGCATGACGTGAGGCCATGGATGCAGGCATTAAG*GTATGATCTCGTGAAATCTTGAGAGAAACTGAATGACGAATGAAACTATT	CDS(truncated)/UTR
ENSG00000257594			CTAACACGGTGAAACCTCGTCTCTACTAAAAATACAAAAAAATTAGCCGG*CTGTGGTGGCGGGCGCCTGTAGTCCCAGCTACTCAGGAGGCTGAGGCAGG	exonic(no-known-CDS)/UTR
ENSG00000173702			ATTCTTACCATTTCACACATAAGAAAATTGAGGTTTGGAAGAGTGAAGCG*TTTTTCTTTTTCTTTTTTTTTTTTTGAGACATTGTCTGGCTCTGTCACCT	UTR/intronic
ENSG00000228696			ATTAAGGACTAAATTATCAAAACTGCATGTTTGTTTTTTCTTTTCTTTTC*TTTTTTTTTTTGAGATGAAGTCTCACTCTGTTGCCCAGGCTGGAGTGCAG	UTR/UTR
ENSG00000275395	ENSE00002555095	ENSE00003219075	AGTGATTCTCCAGCCTCACCCTCCCGAGTAGCTGGGATTACAG*GTGAACGGTGTGGACATGAAGCTGCCCGTGGTGCTGGCCAACG	UTR/CDS(truncated)
ENSG00000152457	ENSE00001923221	ENSE00003475855	GCGGTGACCAAGACGCTGCGGGAGCCCGGCGCCGGAGCCCAAG*ATATCTATTGAAATCGAGACTCCTACCCAGATATCTTTAGTGG	out-of-frame
ENSG00000067182	ENSE00003589571	ENSE00003524766	GGAGCTGAACTACAAAACCAATTCTGAGTCTCCCTCTGTCACG*GTGCTCCTGGAGCTGTTGGTGGGAATATACCCCTCAGGGGTTA	in-frame
ENSG00000166762	ENSE00003788646	ENSE00003527303	CATGGCAAAAAGCTACCACCTGCCAGTCTGAAGCACCGAGATG*ACATGGCCGCTTACCAACAAGAAGAGCAGATGCAGCTTCCCCG	in-frame
ENSG00000181856			AATTTACCTAAAACCTTGGTTATCAAATACATCTCCAGTACATTCCGTTC*TTTTTTTTTTTTTTTTTTTTGAGACAGTCTCGCTCTGTCGCCCAGGC	UTR/UTR
ENSG09000001070			ACCTAAAACCTTGGTTATCAAATACATCTCCAGTACATTCCGTTCTTTTT*TTTTTTTTTTTTTTTTTTTTTTAAAGAAACAAGGTCTTGTTATGTTGCTCA	UTR/---
ENSG09000001070			AAAACCTTGGTTATCAAATACATCTCCAGTACATTCCGTTCTTTTTTTTT*TTTTTTTTTTTTTTTTTTAAAGATGGCATCTCGCTGTGCTGCCTAGGCAGA	UTR/---
ENSG00000090382			TTTTTTGTATTTTTAGTAGAGACAGGGTTTCACCGTGTTAGCCAGGATGG*TCGATCTCCTGACCTTGTGATCCACCCACCTCGGCCTCCCAAAGTGCTGG	---/UTR

ENSG00000196531			CTTCAAGAGCCATTTAATAAATATGGCAGAACTATATATGTGTCTTAAAC*CTCAAAGTAAATTTTCCTTGAGAAATAATTTATGTTGAAAAGATTTCCTG	UTR/UTR
ENSG00000176046			GGTTATTTCTTTGATAAGAACTGAATGTGGCCGGGCGCAGTGGCTCACGC*CTGCAATCCCAGCACTTTGGGAGGCCGAGGTGGGAGGATTGCTTGGGCCC	exonic(no-known-CDS)/UTR
ENSG00000176809	ENSE00003258850	ENSE00003547821	GAGAGGAGACTTCCTTGCTTCTTTGGAGAATGATATCAAACCA*AAATTTCCAAGGAAACTATATTTCTTACATTGATGGAAATGTA	in-frame
ENSG00000181856			AATTTACCTAAAACCTTGGTTATCAAATACATCTCCAGTACATTCCGTTC*TTTTTTTTTTTTTTTTTTTTGAGACAGTCTCGCTCTGTCGCCCAGGCT	UTR/UTR
ENSG00000246339			GGCTGGTGACCTCCCTGGGCCTCTGGAAGCTTTGTTTCTTCTGTACCCGG*TTCCTCTACTTGGCTCTCCTCCAAAGGTACGCGGCCACAGCAGGCAGGGG	UTR/exonic(no-known-CDS)
ENSG00000246339			CCCTGGATGAAGAGCTGTCACCTCTGCCTTGCCCTTTAACTCAACCCGCT*CCCAGAGACAGGCTCGCAGCTGCCGACCTTATCCACTCGCCCTTTCTGCT	UTR/exonic(no-known-CDS)
ENSG00000257764			TCCCAGCACTTGGGGAGGCTGAGGTGGGTGGATCACAAGGTCAGGAGATC*GACCATCCTGGCTAACACGGTGAAACCCTGTCTCTACTAAAAATACAAAA	exonic(no-known-CDS)/intronic
ENSG00000283674	ENSE00002950429	ENSE00003796822	CAGGGAGGAGACCGGCACCCAGACAGGTGGCGACGGCAGAGGA*GTGCAGTGGTTCACACCTGTAATCCCAGCACTTTGGGAGGCCG	exonic(no-known-CDS)/exonic(no-known-CDS)
ENSG00000204604			TCTCCCGTGATCCAGTGACATGAACTTGGGAAGAGGCTGCACTGGGCATG*GTCCTGGGAAGGGCTCACACCCCGACATGGATGGACACGGGGTGAGGGTC	UTR/CDS(truncated)
ENSG00000170949			CCTTCAGTCCCTCTCATCTCGCTTAGGTTCCGTCTCTCGTGACCCAGTGT*CATGAACTTGGGAAGAGGCTGCACTGGGCATGGTCCTGGGAAGGGCTCAC	CDS(truncated)/UTR
ENSG00000170498	ENSE00001377126	ENSE00001125940	CCCCAGGCTGAGTGCGGCATGATCTCCATCACCGAATGGCAGA*CCTCAAGGCACTTCTAGGACCTGCCTCTTCTCACCAAGATGAA	CDS(truncated)/UTR
ENSG00000176809	ENSE00002635959	ENSE00003526242	ATAAAGAAAGAAAGGGATAGATGGAATGAGTTTCATCGTGATT*AATTCTCAATCACAATCCTCTGACAACTGTTGAAGATCCATAT	UTR/CDS(truncated)
ENSG00000197822	ENSE00003590701	ENSE00003560198	TTATTGATCCAAATTTCAGCTGAGTATGATCCTTCCAAACTAG*GTTAAAAATGTGTCTGCAGGCACACAGGACGTGCCTTCACCCC	out-of-frame
ENSG00000181856			AATTTACCTAAAACCTTGGTTATCAAATACATCTCCAGTACATTCCGTTC*TTTTTTTTTTTTGAGACAGTCTCGCTCTGTCGCCCAGGCTCGAGTGCAG	UTR/UTR
ENSG09000001070			GCCTCCCGAGTAGCTGGGATTACGGGCGCCCGCCACCACGCCCGGCTAAT*TTTTTTGTATTTTTAGTAGAGACAGGGTTTCACCGTGTTAGCCAGGATGG	UTR/---
ENSG09000001072			GGTTATTTCTTTGATAAGAACTGAATGTGGCCGGGCGCAGTGGCTCACGC*CTGTAATCCCAACACCTTGGGAGGCCGAGGCGGGTGGATCACATGACGTC	exonic(no-known-CDS)/---
ENSG00000257764			CGAGACCATCCTGGCTAACACGGTGAAACCCTGTCTCTACTAAAAATACA*AAAAAATTAGCCGGGCGTGGTGGCGGGCGCCCGTAATCCCAGCTACTCGG	---/intronic
ENSG00000147789			CACGCCTGTAATCCCAGCACTTTGGGAGGCCGAGGTGGGTGGATCACAAG*GTCAGGAGATCGAGACCATCCTGGCCAACACGGTGAAACCCTGTGTCTAC	intronic/UTR
ENSG00000172175			GAGACAGGGTTTCACCGTGTTAGCCAGGATGGTCTCGATCTCCTGACCTT*GTCATCCACCCACCTCGGCCTCCCAAAGTGCTGGGATTACAGGCGTGAGC	UTR/intronic
ENSG00000185829	ENSE00003662350	ENSE00003806456	TCGTCAGCAAACAGACATTTACAAACAGATACGTGCTAATAAG*GTTTCTGTGTGGAGACAGTAGAATATAAAAATAACACCTTCGC	out-of-frame
ENSG00000228696	ENSE00003662350	ENSE00002370389	TCGTCAGCAAACAGACATTTACAAACAGATACGTGCTAATAAG*GTTTCTGTGTGGAGACAGTAGAATATAAAAATAACACCTTCGC	out-of-frame
ENSG00000228335			CATTTGGTTTTAGGAAGAAGAAACAAAGCCCATTGAGCTCCCTGTCAAAG*AGGAAGAACCCCCTGAAAAAAACTGTTGATGTGGCAGCAGAGAAGAAAGT	CDS(truncated)/exonic(no-known-CDS)
ENSG00000283352	ENSE00002056284	ENSE00003800756	TGGGGTGTGCGGCCAGCCATGGAGCGCTCTGGGCCCAGCGAAG*TGACAGGCTCAGACGCATCGGGACCGGACCCGCAGCTTGTGGT	CDS(truncated)/exonic(no-known-CDS)
ENSG00000172530	ENSE00002235591	ENSE00002213839	GGTGCAGATCCACCAGATCGGAGAAGACGGACAGGTGCAAGTA*ATCCCACAGGGACACCTCCACATCGCCCAGGTGCCGCAGGGGG	exonic(no-known-CDS)/CDS(truncated)
ENSG00000075790	ENSE00003520906	ENSE00003577399	TTAAAGGAAGCAGAAAATGTGTGGCGGATTACTGGGACAGATG*GTGTGAAGAAAAAAATGACACTCCAATGGGCTGCAGTGGCAAC	CDS(truncated)/UTR

Supplementary Table 5d: all fusion genes identified in the plasma samples (EAC, HGD and NDB). Fusion gene pairs indicated in red are likely false positives, based on fusion description (see Supplementary Table 5a) or a high number of common mapping reads (>10).

sample ID	gene 1 symbol (5' end fusion partner)	gene 2 symbol (3' end fusion partner)	fusion_description	counts_of_common_mapping_reads	spanning_pairs	spanning_unique_reads	longest_anchor_found	fusion_finding_method	fusion_point_for_gene_1(5' end_fusion_partner)	fusion_point_for_gene_2(3' end_fusion_partner)	gene_1_id(5' end_fusion_partner)
ID20_EAC	*no fusion genes detected*										
ID29_EAC	ARL17A	KANSL1	banned,known,healthy,bodymap2,hpa,gtex,18cancers,chimerdb3seq,m0,multi,exon-exon	0	2	2	23	BOWTIE	17:46570759:-	17:46094701:-	ENSG00000185829
	ARL17B	KANSL1	banned,known,healthy,bodymap2,hpa,chimerdb3seq,m0,multi,10K<gap<100K,exon-exon	0	2	2	23	BOWTIE	17:46352820:-	17:46094701:-	ENSG00000228696
ID30_EAC	*no fusion genes detected*										
ID43_EAC	*no fusion genes detected*										
ID2_HGD	ARL17A	KANSL1	banned,known,healthy,bodymap2,hpa,gtex,18cancers,chimerdb3seq,m0,multi,exon-exon	0	4	4	26	BOWTIE	17:46570759:-	17:46094701:-	ENSG00000185829
	ARL17B	KANSL1	banned,known,healthy,bodymap2,hpa,chimerdb3seq,m0,multi,10K<gap<100K,exon-exon	0	4	4	26	BOWTIE	17:46352820:-	17:46094701:-	ENSG00000228696
	KANSL1	ARL17A	banned,known,healthy,bodymap2,hpa,gtex,18cancers,chimerdb3seq,m0,multi,exon-exon	0	4	2	29	BOWTIE	17:46094560:-	17:46570869:-	ENSG00000120071
	KANSL1	ARL17B	banned,known,healthy,bodymap2,hpa,chimerdb3seq,m0,multi,10K<gap<100K,exon-exon	0	4	2	29	BOWTIE	17:46094560:-	17:46352930:-	ENSG00000120071
	AL138694.1	TNFSF13B	adjacent,cancer,10K<gap<100K,exon-exon	0	2	2	27	BOWTIE	13:108335572:+	13:108303253:+	ENSG00000283384
ID5_HGD	KMT2C	AC100868.1	cancer,m2	0	7	3	31	BOWTIE+STAR	7:152435626:-	8:132080325:-	ENSG00000055609
	KMT2C	AC100868.1	cancer,m2	0	7	3	31	BOWTIE+STAR	7:152435626:-	8:132085639:-	ENSG00000055609
ID25_HGD	*no fusion genes detected*										
ID26_HGD	*no fusion genes detected*										
ID39_HGD	*no fusion genes detected*										
ID1_NDB	ARL17A	KANSL1	banned,known,healthy,bodymap2,hpa,gtex,18cancers,chimerdb3seq,m0,multi,exon-exon	0	12	6	29	BOWTIE	17:46570759:-	17:46094701:-	ENSG00000185829
	ARL17B	KANSL1	banned,known,healthy,bodymap2,hpa,chimerdb3seq,m0,multi,10K<gap<100K,exon-exon	0	12	6	29	BOWTIE	17:46352820:-	17:46094701:-	ENSG00000228696
	KANSL1	ARL17A	banned,known,healthy,bodymap2,hpa,gtex,18cancers,chimerdb3seq,m0,multi,exon-exon	0	12	3	23	BOWTIE	17:46094560:-	17:46570869:-	ENSG00000120071
	KANSL1	ARL17B	banned,known,healthy,bodymap2,hpa,chimerdb3seq,m0,multi,10K<gap<100K,exon-exon	0	12	3	23	BOWTIE	17:46094560:-	17:46352930:-	ENSG00000120071
	MAML3	IRF2BPL	m2	12	3	2	28	BOWTIE+STAR	4:139889492:-	14:77027450:-	ENSG00000196782
	MAML3	IRF2BPL	m2	12	3	2	28	BOWTIE+STAR	4:139889951:-	14:77027450:-	ENSG00000196782
	NPEPPS	TBC1D3	banned,known,oncogene,bodymap2,hpa,gtex,18cancers,tumor,m0,multi,exon-exon	0	1	3	21	BOWTIE	17:47592545:+	17:38191030:-	ENSG00000141279
ID18_NDB	*no fusion genes detected*										
ID19_NDB	ARL17A	KANSL1	banned,known,healthy,bodymap2,hpa,gtex,18cancers,chimerdb3seq,m0,multi,exon-exon	0	11	8	26	BOWTIE	17:46570759:-	17:46094701:-	ENSG00000185829
	ARL17B	KANSL1	banned,known,healthy,bodymap2,hpa,chimerdb3seq,m0,multi,10K<gap<100K,exon-exon	0	11	8	26	BOWTIE	17:46352820:-	17:46094701:-	ENSG00000228696
	KANSL1	ARL17A	banned,known,healthy,bodymap2,hpa,gtex,18cancers,chimerdb3seq,m0,multi,exon-exon	0	11	5	28	BOWTIE	17:46094560:-	17:46570869:-	ENSG00000120071
	KANSL1	ARL17B	banned,known,healthy,bodymap2,hpa,chimerdb3seq,m0,multi,10K<gap<100K,exon-exon	0	11	5	28	BOWTIE	17:46094560:-	17:46352930:-	ENSG00000120071
	KMT2C	AC100868.1	cancer,m3	0	5	3	29	BOWTIE+STAR	7:152435626:-	8:132080325:-	ENSG00000055609
	KMT2C	AC100868.1	cancer,m3	0	5	3	29	BOWTIE+STAR	7:152435626:-	8:132085639:-	ENSG00000055609
	SMG1	NPIPB5	banned,known,bodymap2,hpa,m0,multi,exon-exon	0	2	2	23	BOWTIE	16:18858170:-	16:22513522:+	ENSG00000157106
	RMND5A	ANAPC1	banned,known,bodymap2,hpa,m0,multi,exon-exon	0	1	3	26	BOWTIE	2:86741069:+	2:111822600:-	ENSG00000153561
ID22_NDB	ARL17A	KANSL1	banned,known,healthy,bodymap2,hpa,gtex,18cancers,chimerdb3seq,m0,multi,exon-exon	0	4	2	29	BOWTIE	17:46570759:-	17:46094701:-	ENSG00000185829
	ARL17B	KANSL1	banned,known,healthy,bodymap2,hpa,chimerdb3seq,m0,multi,10K<gap<100K,exon-exon	0	4	2	29	BOWTIE	17:46352820:-	17:46094701:-	ENSG00000228696
	KANSL1	ARL17A	banned,known,healthy,bodymap2,hpa,gtex,18cancers,chimerdb3seq,m0,multi,exon-exon	0	4	2	30	BOWTIE	17:46094560:-	17:46570869:-	ENSG00000120071
	KANSL1	ARL17B	banned,known,healthy,bodymap2,hpa,chimerdb3seq,m0,multi,10K<gap<100K,exon-exon	0	4	2	30	BOWTIE	17:46094560:-	17:46352930:-	ENSG00000120071
ID33_NDB	*no fusion genes detected*										
	ARL17A	KANSL1	banned,known,healthy,bodymap2,hpa,gtex,18cancers,chimerdb3seq,m0,multi,exon-exon	0	9	6	25	BOWTIE	17:46570759:-	17:46094701:-	ENSG00000185829
	ARL17B	KANSL1	banned,known,healthy,bodymap2,hpa,chimerdb3seq,m0,multi,10K<gap<100K,exon-exon	0	9	6	25	BOWTIE	17:46352820:-	17:46094701:-	ENSG00000228696

**ID35_NDB**	KANSL1	ARL17A	banned,known,healthy,bodymap2,hpa,gtex,18cancers,chimerdb3seq,m0,multi,exon-exon	0	9	6	29	BOWTIE	17:46094560:-	17:46570869:-	ENSG00000120071
	KANSL1	ARL17B	banned,known,healthy,bodymap2,hpa,chimerdb3seq,m0,multi,10K<gap<100K,exon-exon	0	9	6	29	BOWTIE	17:46094560:-	17:46352930:-	ENSG00000120071
	SMG1	NPIPB5	banned,known,bodymap2,hpa,m0,multi,exon-exon	0	6	2	24	BOWTIE	16:18858170:-	16:22523820:+	ENSG00000157106
	NPEPPS	TBC1D3	banned,known,oncogene,bodymap2,hpa,gtex,18cancers,tumor,m0,multi,exon-exon	0	1	2	21	BOWTIE	17:47592545:+	17:38191030:-	ENSG00000141279
**ID37_NDB**	ARL17A	KANSL1	banned,known,healthy,bodymap2,hpa,gtex,18cancers,chimerdb3seq,m0,multi,exon-exon	0	5	4	29	BOWTIE	17:46570759:-	17:46094701:-	ENSG00000185829
	ARL17B	KANSL1	banned,known,healthy,bodymap2,hpa,chimerdb3seq,m0,multi,10K<gap<100K,exon-exon	0	5	4	29	BOWTIE	17:46352820:-	17:46094701:-	ENSG00000228696
	KANSL1	ARL17A	banned,known,healthy,bodymap2,hpa,gtex,18cancers,chimerdb3seq,m0,multi,exon-exon	0	5	5	28	BOWTIE	17:46094560:-	17:46570869:-	ENSG00000120071
	KANSL1	ARL17B	banned,known,healthy,bodymap2,hpa,chimerdb3seq,m0,multi,10K<gap<100K,exon-exon	0	5	5	28	BOWTIE	17:46094560:-	17:46352930:-	ENSG00000120071
	PCNP	ACTB	cancer	0	3	6	28	BOWTIE+STAR	3:101592187:+	7:5527639:-	ENSG00000081154
	PCNP	ACTB	cancer	0	3	6	27	BOWTIE+STAR	3:101591763:+	7:5527637:-	ENSG00000081154
	PCNP	ACTB	cancer	0	3	3	25	BOWTIE+STAR	3:101591788:+	7:5527639:-	ENSG00000081154
	ZNF83	ZNF137P	no_protein,ensembl_partially_overlapping,pseudogene,gencode_partially_overlapping,m2	7	3	6	36	BOWTIE+STAR	19:52655561:-	19:52588532:+	ENSG00000167766
	RMND5A	ANAPC1	banned,known,bodymap2,hpa,m0,multi,exon-exon	0	2	3	29	BOWTIE	2:86741069:+	2:111822600:-	ENSG00000153561
**ID40_NDB**	*no fusion genes detected*										

gene_2_id(3' end_fusion_partner)	exon_1_id(5' end_fusion_partner)	exon_2_id(3' end_fusion_partner)	fusion_sequence	predicted_effect
ENSG00000120071	ENSE00003806456	ENSE00003662350	AAAATCAGACCTCTGTGGCAGCATTTTTTCCAGAACACAAAAG*GAGACGCAGGTCAGAATGGAAATGGGCTGCAGACCGGGCAGCT	in-frame
ENSG00000120071	ENSE00002370389	ENSE00003662350	AAAATCAGACCTCTGTGGCAGCATTTTTTCCAGAACACAAAAG*GAGACGCAGGTCAGAATGGAAATGGGCTGCAGACCGGGCAGCT	in-frame
ENSG00000120071	ENSE00003806456	ENSE00003662350	AAAATCAGACCTCTGTGGCAGCATTTTTTCCAGAACACAAAAG*GAGACGCAGGTCAGAATGGAAATGGGCTGCAGACCGGGCAGCT	in-frame
ENSG00000120071	ENSE00002370389	ENSE00003662350	AAAATCAGACCTCTGTGGCAGCATTTTTTCCAGAACACAAAAG*GAGACGCAGGTCAGAATGGAAATGGGCTGCAGACCGGGCAGCT	in-frame
ENSG00000185829	ENSE00003662350	ENSE00003806456	TCGTCAGCAAACAGACATTTACAAACAGATACGTGCTAATAAG*GTTTCTGTGTGGAGACAGTAGAATATAAAAATAACACCTTCGC	out-of-frame
ENSG00000228696	ENSE00003662350	ENSE00002370389	TCGTCAGCAAACAGACATTTACAAACAGATACGTGCTAATAAG*GTTTCTGTGTGGAGACAGTAGAATATAAAAATAACACCTTCGC	out-of-frame
ENSG00000102524	ENSE00003795801	ENSE00003687225	CTGTGATGAGCTGAGTCATGGGTGAGAGCTGGAGAAAACAGAG*GATCTTACACATTTGTTCCATGGCTTCTCAGCTTTAAAAGGGG	UTR/CDS(truncated)
ENSG00000258417			GGCCGGCCTCGCAAAGATGGCGCTTCCCCTTTCCAGAGAGCCAGAAAGAA*ACTCAGCCCGCCTGCACCCAGGTGAAATAAACAGCTTTATTGCTCACACA	CDS(truncated)/intronic
ENSG00000258417			GGCCGGCCTCGCAAAGATGGCGCTTCCCCTTTCCAGAGAGCCAGAAAGAA*ACTCAGCCCGCCTGCACCCAGGTGAAATAAACAGCTTTATTGCTCACACA	CDS(truncated)/UTR
ENSG00000120071	ENSE00003806456	ENSE00003662350	AAAATCAGACCTCTGTGGCAGCATTTTTTCCAGAACACAAAAG*GAGACGCAGGTCAGAATGGAAATGGGCTGCAGACCGGGCAGCT	in-frame
ENSG00000120071	ENSE00002370389	ENSE00003662350	AAAATCAGACCTCTGTGGCAGCATTTTTTCCAGAACACAAAAG*GAGACGCAGGTCAGAATGGAAATGGGCTGCAGACCGGGCAGCT	in-frame
ENSG00000185829	ENSE00003662350	ENSE00003806456	TCGTCAGCAAACAGACATTTACAAACAGATACGTGCTAATAAG*GTTTCTGTGTGGAGACAGTAGAATATAAAAATAACACCTTCGC	out-of-frame
ENSG00000228696	ENSE00003662350	ENSE00002370389	TCGTCAGCAAACAGACATTTACAAACAGATACGTGCTAATAAG*GTTTCTGTGTGGAGACAGTAGAATATAAAAATAACACCTTCGC	out-of-frame
ENSG00000119669			TCCAGCAGCAGCAACAGCAGCAGCAACAGCAACAGCAGCAGCAGCAGCAG*CAGCAGCAGCAGCAGCAGCAGCAGCAGCAACAACAGCTCAACCACGT	in-frame
ENSG00000119669			CCAAACTCATGCAGCAGAAACAGCAACAGCAACAGCAGCAGCAGCAGCAG*CAGCAGCAGCAGCAGCAGCAGCAGCAGCAACAACAGCTCAACCACGT	in-frame
ENSG00000274611	ENSE00003785912	ENSE00003731868	AACATGTATTTAACCAAGTTCCAACAAAAGAATGCTGCCACAG*GATGGACGTGGTAGAGGTCGCGGGCAGTTGGTGGGCACAAGAG	CDS(complete)/UTR
ENSG00000120071	ENSE00003806456	ENSE00003662350	AAAATCAGACCTCTGTGGCAGCATTTTTTCCAGAACACAAAAG*GAGACGCAGGTCAGAATGGAAATGGGCTGCAGACCGGGCAGCT	in-frame
ENSG00000120071	ENSE00002370389	ENSE00003662350	AAAATCAGACCTCTGTGGCAGCATTTTTTCCAGAACACAAAAG*GAGACGCAGGTCAGAATGGAAATGGGCTGCAGACCGGGCAGCT	in-frame
ENSG00000185829	ENSE00003662350	ENSE00003806456	TCGTCAGCAAACAGACATTTACAAACAGATACGTGCTAATAAG*GTTTCTGTGTGGAGACAGTAGAATATAAAAATAACACCTTCGC	out-of-frame
ENSG00000228696	ENSE00003662350	ENSE00002370389	TCGTCAGCAAACAGACATTTACAAACAGATACGTGCTAATAAG*GTTTCTGTGTGGAGACAGTAGAATATAAAAATAACACCTTCGC	out-of-frame
ENSG00000258417			GGCCGGCCTCGCAAAGATGGCGCTTCCCCTTTCCAGAGAGCCAGAAAGAA*ACTCAGCCCGCCTGCACCCAGGTGAAATAAACAGCTTTATTGCTCACACA	CDS(truncated)/intronic
ENSG00000258417			GGCCGGCCTCGCAAAGATGGCGCTTCCCCTTTCCAGAGAGCCAGAAAGAA*ACTCAGCCCGCCTGCACCCAGGTGAAATAAACAGCTTTATTGCTCACACA	CDS(truncated)/UTR
ENSG00000243716	ENSE00001505919	ENSE00002138288	AGGTATACTATGTACCAGAATCAGTTGTTGGAGAAAATTAAAG*GTATGATCTCGTGAAATCTTGAGAGAAACTGAATGACGAATGA	CDS(truncated)/UTR
ENSG00000153107	ENSE00002531730	ENSE00002441208	CATCCACAGCAGTGTTTCTCGGGTTGGAAAAGCCATTGATAAG*GATTCACTTTAAGAGATTTGGAAACTCTTCCCTTTGGAATTGC	out-of-frame
ENSG00000120071	ENSE00003806456	ENSE00003662350	AAAATCAGACCTCTGTGGCAGCATTTTTTCCAGAACACAAAAG*GAGACGCAGGTCAGAATGGAAATGGGCTGCAGACCGGGCAGCT	in-frame
ENSG00000120071	ENSE00002370389	ENSE00003662350	AAAATCAGACCTCTGTGGCAGCATTTTTTCCAGAACACAAAAG*GAGACGCAGGTCAGAATGGAAATGGGCTGCAGACCGGGCAGCT	in-frame
ENSG00000185829	ENSE00003662350	ENSE00003806456	TCGTCAGCAAACAGACATTTACAAACAGATACGTGCTAATAAG*GTTTCTGTGTGGAGACAGTAGAATATAAAAATAACACCTTCGC	out-of-frame
ENSG00000228696	ENSE00003662350	ENSE00002370389	TCGTCAGCAAACAGACATTTACAAACAGATACGTGCTAATAAG*GTTTCTGTGTGGAGACAGTAGAATATAAAAATAACACCTTCGC	out-of-frame
ENSG00000120071	ENSE00003806456	ENSE00003662350	AAAATCAGACCTCTGTGGCAGCATTTTTTCCAGAACACAAAAG*GAGACGCAGGTCAGAATGGAAATGGGCTGCAGACCGGGCAGCT	in-frame
ENSG00000120071	ENSE00002370389	ENSE00003662350	AAAATCAGACCTCTGTGGCAGCATTTTTTCCAGAACACAAAAG*GAGACGCAGGTCAGAATGGAAATGGGCTGCAGACCGGGCAGCT	in-frame

ENSG00000185829	ENSE00003662350	ENSE00003806456	TCGTCAGCAAACAGACATTTACAAACAGATACGTGCTAATAAG*GTTTCTGTGTGGAGACAGTAGAATATAAAAATAACACCTTCGC	out-of-frame
ENSG00000228696	ENSE00003662350	ENSE00002370389	TCGTCAGCAAACAGACATTTACAAACAGATACGTGCTAATAAG*GTTTCTGTGTGGAGACAGTAGAATATAAAAATAACACCTTCGC	out-of-frame
ENSG00000243716	ENSE00001505919	ENSE00003563471	AGGTATACTATGTACCAGAATCAGTTGTTGGAGAAAATTAAAG*GTTATCAATACTCTGGCTGACCATCATCATCGTGGGACTGACT	in-frame
ENSG00000274611	ENSE00003785912	ENSE00003731868	AACATGTATTTAACCAAGTTCCAACAAAAGAATGCTGCCACAG*GATGGACGTGGTAGAGGTCGCGGGCAGTTGGTGGGCACAAGAG	CDS(complete)/UTR
ENSG00000120071	ENSE00003806456	ENSE00003662350	AAAATCAGACCTCTGTGGCAGCATTTTTTCCAGAACACAAAAG*GAGACGCAGGTCAGAATGGAAATGGGCTGCAGACCGGGCAGCT	in-frame
ENSG00000120071	ENSE00002370389	ENSE00003662350	AAAATCAGACCTCTGTGGCAGCATTTTTTCCAGAACACAAAAG*GAGACGCAGGTCAGAATGGAAATGGGCTGCAGACCGGGCAGCT	in-frame
ENSG00000185829	ENSE00003662350	ENSE00003806456	TCGTCAGCAAACAGACATTTACAAACAGATACGTGCTAATAAG*GTTTCTGTGTGGAGACAGTAGAATATAAAAATAACACCTTCGC	out-of-frame
ENSG00000228696	ENSE00003662350	ENSE00002370389	TCGTCAGCAAACAGACATTTACAAACAGATACGTGCTAATAAG*GTTTCTGTGTGGAGACAGTAGAATATAAAAATAACACCTTCGC	out-of-frame
ENSG00000075624			TGTTTTTTGTTTTTTTGTGTGTTTTTTGTTTTTTGTTTTTTTGTTTGTT*TTTTTTTTTTTTTTTTTGGCTTGACTCAGGATTTAAAAACTGGAACGGTGA	intronic/UTR
ENSG00000075624			TAATGGATTACAGGCAACAGGTTTTTTTGTTTTTTTGTTTTGATTTTG*TTTTTTTTTTTTTTTGGCTTGACTCAGGATTTAAAAACTGGAACGGTGA	intronic/UTR
ENSG00000075624			TTTTTGTTTTTTTGTTTTGATTTTGGTGTTTTTTTTTTTTTTTTTTTTT*TTTTTTTTTTTTTTTTTGGCTTGACTCAGGATTTAAAAACTGGAACGGTGA	intronic/UTR
ENSG00000123870			TTTATACAGGGCCGTGATGTTGGAGAACTACAGGAACCTGGAGTCTGTGG*GATTGATTTCCAAAGACTCATGCTATGTAAGGAAGCCACCAAGAAGGGCA	UTR/exonic(no-known-CDS)
ENSG00000153107	ENSE00002531730	ENSE00002441208	CATCCACAGCAGTGTTTCTCGGGTTGGAAAAGCCATTGATAAG*GATTCACTTTAAGAGATTTGGAAACTCTTCCCTTTGGAATTGC	out-of-frame
