# Supplementary material for: Comprehensive RNA dataset of tissue and plasma from patients with esophageal cancer or precursor lesions
Source: Sci Data. 2022 Mar 14;9:86. doi: 10.1038/s41597-022-01176-x (PMC8921197; doi:10.1038/s41597-022-01176-x)
Supplement: Supplementary file 6 — Supplementary File 1 [file 41597_2022_1176_MOESM6_ESM.pdf]

Supplementary File 1: Templates for a Data Transfer Agreement for requesting raw sequencing data (EGAS0000100493935). For EU institutes/companies, DTA_doc1 has to be signed; for non-EU institutes/companies, both DTA_doc1 and DTA_doc2 have to be signed.

DTA_doc1:

DATA TRANSFER AGREEMENT

GHENT UNIVERSITY

This data transfer agreement (the "Agreement") is entered into on July 14th, 2021 by and between the following parties:

Universiteit Gent (Ghent University), public institution with legal personality, having its administrative offices in Belgium, B-9000 Gent, Sint-Pietersnieuwstraat 25, company registration number 0248.015.142, for whom prof. dr. Rik Van de Walle, rector, for whom mr. Wim Van Camp, general manager UGent TechTransfer, acts by delegation pursuant to the Board of Governors' decision ("**UGent**" or "**Provider**")

AND

[name of institution/company], having its offices in [full address], duly represented by [name of legal representative], [position] ("**Recipient**")

**Start Date:**	[date]	**Term:**	[date or period]

UGent owns the **Data** as described in the **Technical Annex** and controls certain valuable technical and proprietary information relating to this Data;

Recipient wants to obtain a (partial) copy of the Data from UGent for use in the **Research** as described in the Technical Annex and UGent is willing to provide Recipient access to the Data under the General Terms set forth in this Agreement;

The Data contains personal data as defined in European Regulation 2016/679 of 27 April 2016 concerning the protection of natural persons with respect to the processing of personal data and the free movement of data and until the repeal of Regulation 95/46/EC (hereinafter the "**GDPR**"); in addition to the General Terms, the Special Terms relating to the storage, handling and processing of personal data ("Special Terms") and the Standard contractual clauses are applicable to this Agreement.

This Agreement consists of this **Signing Page**, the **General Terms**, the **Technical Annex**, the **Special Terms** and the **Standard contractual clauses** . In case of conflict between these documents, the Special Terms shall take precedence.

This Agreement constitutes the entire agreement between the parties and supersedes all prior arrangements, understandings, representations and communications, oral or written with respect to its subject matter.

**For Recipient [name instution/company]**		**For Universiteit Gent (Ghent University)**	
Signature:		Signature:	
Name:		Name:	Wim Van Camp
Position:		Position:	General manager UGent TechTransfer
Date:		Date:	
**Recipient Lead Scientist (if different from above)**		**UGent Promoter**	
Signature		Signature:	
Name		Name:	[name professor, principal investigator]
Department		Department:	[department]
Date:		Date:	

-- END OF SIGNING PAGE --

GENERAL TERMS – DATA TRANSFER AGREEMENT

Article 1 Definitions

1.1 "**Commercial Purposes**" means any and all (research) activities generating revenues for Recipient or any transfer or license of rights to non-academic third parties, including but not limited to the sale or licence of the Data, the use of the Data in industry sponsored projects, the use of the Date for the purpose of producing or manufacturing products for general sale or the use of the Data in fee-for-service activities conducted to the benefit of a third party.

1.2 "**Confidential Information**" means information disclosed by one party to the other and identified in writing as confidential before or at the time of disclosure, or the confidential nature of which was confirmed by the disclosing party in writing within **ten calendar days** counting from the date of its disclosure. In any case however, the Data, and any information related to the Data disclosed by Provider to the Recipient shall be Confidential Information whether or not labelled as such.

1.3 "**Inventions**" shall mean any and all inventions, know-how, materials, substances and other results conceived or generated by Recipient (whether patentable or not) and related to the Data or its use, or developed using the Data.

1.4 "**Data**" refers to the data that is covered by this Agreement and shall mean the data as described in the Technical Annex.

1.5 "**Research**" shall mean the non-commercial research project and non-commercial research activities as defined in the Technical Annex.

Article 2 Use of the Data

2.1 Except as explicitly provided herein, this Agreement does not imply any direct or indirect license. Nothing in this Agreement shall be deemed to grant Recipient any rights under any proprietary rights, patent or patent application, nor any direct or indirect rights or license to use, or permit the use of, any products or processes containing, using, or derived from the Data for commercial purposes.

2.2 The Data is provided to the Recipient for the sole purpose of **academic internal non-commercial research**. Recipient will use the Data solely for the Research under the direction of Recipient's Lead Scientist as identified on the Signing Page. Recipient is not permitted to use the Date for Commercial Purposes.

2.3 Recipient shall not transmit by any means whatsoever all or part of the Data, to any third party without the express and specific prior written consent of Provider. Recipient shall refer any third-party request for access to the Data to Provider.

2.4 Recipient shall limit access to the Data to those of its employees who have a need to know to execute the Research. Recipient shall ensure that any of its personnel involved in the Research comply with the provisions of this Agreement.

2.5 The Recipient agrees to use the Data in compliance with all applicable statutes and regulations.

Article 3 Intellectual Property

3.1 The Data is and remains the property of Provider, including any form or part of the Data included in Inventions.

3.2 Recipient will promptly and fully disclose in writing to Provider any and all patentable or commercially useful Inventions.

3.3 Ownership of any Inventions will be negotiated and agreed by the Parties in good faith taking into account the Parties' respective contributions to such Inventions. The Provider will in any case be granted a royalty free, non-exclusive license to any Invention for its internal non-commercial research and teaching purposes. Recipient agrees not to apply for any patent or any other industrial property title, which would claim Inventions, without prior written agreement between Provider and Recipient. In any case, if any revenues

result from Recipient’s use of the Inventions, the Provider will be entitled to a fair and reasonable share of any such revenues.

Article 4 Confidentiality

4.1 Confidential Information shall not be distributed, disclosed, or disseminated in any way or form by Recipient, except to the own employees who have a reasonable need to know the Confidential Information for the Research and who shall be bound by confidentiality obligations at least as stringent as the one provided for in this Agreement. The Recipient shall keep the Data confidential for as long as none of the exceptions as listed below apply and shall in any case respect the intellectual property rights in and to the Data, such as but not limited to the database rights, controlled by the Provider. The obligations of confidentiality shall not apply to any information, which the Recipient can prove:

a) is or becomes part of the public domain, through no breach of this Agreement by Recipient;

b) was in Recipient’s possession prior to receipt from Provider;

c) is received by Recipient from a third party free to disclose such information;

d) is independently developed by Recipient, without use of Provider’s Confidential Information; or

e) is approved for release by prior written authorization of the Provider.

The above obligations of confidentiality shall furthermore not apply to information to the extent such information is required to be disclosed by operation of law or by court or administrative order. The Recipient will furnish prompt and prior written notice of such requirement to the Provider and will cooperate with the Provider in contesting a disclosure.

Article 5 Publications

5.1 The Recipient agrees to acknowledge the Provider and the source of the Data in any publications reporting the scientific results of the Research resulting from the use of the Data, as is customary in the scientific community, and agrees to cite in any publication reporting results of the Research using of the Data as specified in the Technical Annex.

Article 6 Warranties and limitation of liability

6.1 Recipient understands that the Data is experimental in nature. The Provider makes no representations and gives no warranties either express or implied in relation to it. For example, no warranties are given about quality or fitness for a particular purpose; or that the use of the Data will not infringe any intellectual property or other rights of third parties. Notwithstanding the above, in supplying the Data, Provider does warrant that the original collection of the Data complied with all legal and ethical requirements and guidelines including the applicable regulations concerning processing and protection of personal data and that it has obtained the express informed consent, if applicable, to the use of such Data for the Research and that such express informed consent permits Recipient to use the Data, in accordance with the provisions of this Agreement.

6.2 Recipient assumes all liability for damages which may arise from its use, storage or disposal of the Data. The Provider will not be liable to Recipient for any loss, claim or demand made by Recipient, or made against Recipient by any other party, due to or arising from the use, storage or disposal of the Data by the Recipient.

6.3 The liability of Provider for any breach of Providers’ obligations under this Agreement will in no event extend to any indirect damages or losses, or to any loss of profits, loss of revenue, loss of data, loss of contracts or opportunity (whether direct or indirect), even if Recipient has advised Provider of the possibility of those losses, or even if they were within the Recipient’s contemplation.

6.4 Notwithstanding the foregoing, a Party’s liability shall not be excluded or limited in the event and to the extent damages are caused by the wilful misconduct of such a Party and any limitations or exclusions of liability under this Agreement shall not apply to the extent such liability cannot be limited or excluded by applicable law.

6.5 Either Party represent that this Agreement, to the best of its knowledge, does not, and will not conflict with any other right or obligation provided under any other agreement or obligation that either Party has with any third party.

Article 7 Term & Termination

7.1 This Agreement shall commence on the Start Date and will (subject to earlier termination pursuant to clause 7.2) continue until the End Date.

7.2 Provider may terminate this Agreement if Recipient is in material breach of any of the terms of this Agreement and, where the breach is capable of remedy, Recipient has failed to remedy the same within **twenty calendar days** of a written notice from Provider specifying the breach and requiring it to be remedied.

7.3 Upon expiration or termination of this Agreement, Recipient will discontinue use of the Data and Confidential Information which shall be returned to Provider or be destroyed within **fifteen calendar days**. In case of destruction of the Data and Confidential Information, a written confirmation shall be sent to Provider within **thirty calendar days** after the termination or expiration of the Agreement. One record copy of documents may be retained for the sole purpose of determining compliance under this Agreement.

7.4 Any provisions of this Agreement which by their nature extend beyond termination shall survive the termination of this Agreement

Article 8 Miscellaneous

8.1 **Human Rights:** The parties ensure that they shall respect human rights. Each of the parties may terminate this agreement with immediate effect if the other party is involved in a serious or systematic violation of human rights.

8.2 **Assignment:** Neither party may assign or transfer this Agreement as a whole, or any of its rights or obligations under it, without first obtaining the written consent of the other party. That consent may not be unreasonably withheld or delayed.

8.3 **Illegal/unenforceable provisions**: If the whole or any part of any provision of this Agreement is void or unenforceable in any jurisdiction, the other provisions of this Agreement, and the rest of the void or unenforceable provision, will continue in force in that jurisdiction, and the validity and enforceability of that provision in any other jurisdiction will not be affected.

8.4 **Waiver of rights**: If a party fails to enforce, or delays in enforcing, an obligation of the other party, or fails to exercise, or delays in exercising, a right under this Agreement, that failure or delay will not affect its right to enforce that obligation or constitute a waiver of that right. Any waiver of any provision of this Agreement will not, unless expressly stated to the contrary, constitute a waiver of that provision on a future occasion.

8.5 **No agency**: Nothing in this Agreement creates, implies or evidences any partnership or joint venture between the parties, or the relationship between them of principal and agent. Neither party has any authority to make any representation or commitment, or to incur any liability, on behalf of the other.

8.6 **Entire agreement**: This Agreement constitutes the entire agreement between the parties and supersedes all prior arrangements, understandings, representation and communications, oral or written with respect to the subject matter.

8.7 **Formalities**: Each party will take any action and execute any document reasonably required by the other party to give effect to any of its rights under this Agreement.

8.8 **Amendments**: No variation or amendment of this Agreement will be effective unless it is made in writing and signed by each party's authorised representatives.

8.9 **Export:** The parties shall adhere to all applicable export control laws and regulations, including Council Regulation (EC) No. 428/2009 of 5 May 2009 setting up a Community regime for the control of exports, transfer, brokering and transit of dual-use items, and inform each other if goods, software or technology are

affected by export control laws and regulations. The export of goods, software or technology outside the European Union may be subject to an export license provided by the relevant authority.

8.10 **Governing law**: This Agreement is governed by, and is to be construed in accordance with Belgian Law. The courts of Ghent will have exclusive jurisdiction to deal with any dispute, which has arisen or may arise out of or in connection with this Agreement, except that either party may bring proceedings for an injunction in any jurisdiction.

8.11 **Escalation**: If the parties are unable to reach agreement regarding a dispute on any issue concerning this Agreement within fourteen (14) days after one party has notified the other of that issue, they will refer the matter to the rector or any person appointed by him in the case of UGent, and to a person appointed by Recipient in an attempt to resolve the issue within ten (10) calendar days after the referral. Either party may apply to the court for an injunction in accordance with clause 8.9 if the matter has not been resolved within that period.

-- END OF GENERAL TERMS --

TECHNICAL ANNEX

THE DATA (description)		
**Code**	**Description**	**Technical Format**
The following aspects and related information of the Data are considered to be confidential by Provider: identity, structure, characteristics, conformation, origin and properties of the Data, the uses to which it is or may be put.		

RESEARCH – PURPOSE – RESTRICTED USE

FINANCIAL ARRANGEMENTS
The Material will be delivered to Recipient at no cost.<br>Recipient shall pay a fee of 0 EUR (costs of preparation and distribution) to UGent.

**Notices to Recipient**		**Notices to UGent**	
**Name**			
**Dept.**		**Dept.**	**Technology Transfer**
**Address**		**Address**	p/a Sint-Pietersnieuwstraat 25<br>9000 Gent<br>Belgium
**Tel.**		**Tel**	0032 9 264 30 30
**E-mail**		**E-mail**	contracten@ugent.be

		**IMPORTANT: a copy of all notices to UGent concerning this Agreement has to be sent to the Promoter via e-mail as indicated below.**	
**Recipient Principal Investigator**		**UGent Promoter**	
**Name**		**Name**	
**Tel**		**Tel**	
**E-mail**		**E-mail**	
**Address**		**Address**	

-- END OF TECHNICAL ANNEX –

SPECIAL TERMS – STORAGE, HANDLING AND PROCESSING OF PERSONAL DATA.

As Between the parties as identified in the Data Transfer Agreement **Axx-TT-xxxx** (the **"DTA"**)

This Addendum is an integral part of the DTA; its provisions take precedence over the General Terms. All terms defined in the DTA shall have the same meaning in this Addendum. For the Purpose of this Addendum, both the Provider and the Recipient as identified in the DTA shall be referred to as the "**Controllers**".

In view of the fact that:

The Parties wish to set out their rights and obligations with respect to the protection of personal data as stipulated in European Regulation 2016/679 of 27 April 2016 concerning the protection of natural persons with respect to the processing of personal data and the free movement of data and until the repeal of Regulation 95/46/EC (hereinafter the "**GDPR**") and in the Act of 30 July 2018 on the protection of natural persons with respect to the processing of personal data (hereinafter the "**Privacy Act**") in the current addendum (hereinafter the '**Addendum'**);

The terms used in this Addendum have the same meaning as defined in the GDPR and the Privacy Act;

Within the framework of the DTA, the Recipient may receive and process specific personal data that has been supplied by the Provider.

The Parties represent to being informed and knowledgeable about UGent's General Data Protection Policy, in particular the "Generic Code of Conduct for the processing of personal data and confidential information at Ghent University" as published on the UGent website; UGent shall provide a copy of these document upon first request.

1. SUBJECT

1.1. Recipient will only process personal data made available by or through the Provider within the performance of the DTA. Recipient will not process personal data for any other purpose, unless subject to deviating legal obligations. Recipient may only process the personal data of data subjects that the Provider has obtained on the basis of legitimate legal grounds and for legitimate purposes.

1.2. The Provider will make the following categories of personal data available to the Recipient (non-exhaustive list):

a) [description of the dataset, format, EGA identification]

1.3. The personal data made available by UGent to the Controller comprise the following categories of data subjects (non-exhaustive list):

a) [describe category]
b) [describe category]
c) ….

2. Rights and Obligations of the Parties

2.1. The Provider must ensure that the personal data of the data subject/s are obtained in a valid manner and that it has a legitimate legal ground and purpose for processing them. The Recipient will not check the validity of the legal ground and purpose and therefore cannot be held liable for any fraudulent acts committed by the Provider.

2.2. Personal data will only be processed with regard to passing on personal data to a third country or an international organisation, unless Recipient is obliged to conduct such processing by virtue of a provision of EU or Member State law to which Recipient is subject: in such case, Recipient will inform the Provider of this legal obligation prior to processing, unless legislation forbids such notification for urgent reasons of public interest.

2.3. Recipient will permit and facilitate audits, including inspections by the Provider or an auditor authorised by the Provider. More specifically, the auditor may access the premises and rooms of Recipient where the personal data is processed. The auditor must inform Recipient of this in a suitable manner and present themselves discretely at Recipient's premises and rooms within regular working hours. The costs of audits requested by the Provider will be borne by the Provider.

3. Technical and organisational measures

3.1. UGent's general data protection policy sets out the basic security level at the generic level. UGent's policy on "Working safely with personal data and confidential information", the Regulation on the correct use of the ICT infrastructure and UGent's Generic Code of Conduct provide general guarantees concerning:

- The protection of personal data against unauthorised access or viewing by third parties (confidentiality)
- The protection of personal data against unauthorised changes (integrity)
- The protection of personal data against destruction, loss or if for any reason, it is impossible to consult the data or there is a physical or technical incident, that availability of and access to the personal data will be reinstated in good time (availability)
- The right of data subjects to view their personal data (transparency).

3.2. Recipient will take all technical and organisational measures to guarantee a level of security that is in line with the risk relating to the storage and processing of personal data. This will take into account the state of technology, the implementation costs, the nature, scope, context and processing objectives and the likelihood and seriousness of the various risks. Upon the Provider's request, Recipient will submit documentation describing the measures that have been taken.

3.3. In view of the risks accompanying the specific processing and nature of the data that must be protected, Recipient will take satisfactory additional safety measures that comply with relevant standards and quality requirements.

4. Article 5: Assistance to the Controller

4.1. Parties will provide each other with all information and assistance that is necessary and/or may reasonably be expected to enable them respectively to fulfil their obligations under the GDPR.

4.2. The Recipient will act in accordance with the instructions issued by the Provider with respect to requests from data subjects with regard to their personal data. If a Data Subject submits a request concerning his or her personal data to the Recipient, such request will be immediately referred to the Provider.

4.3. Taking the nature of the processing into account and insofar as possible, Recipient will assist the Provider in fulfilling its obligation to comply with requests from data subjects to exercise their established rights by taking fitting technological and organisational measures.

5. Transfer

5.1. Personal Data may only be processed outside the European Economic Area or by an international organisation if the Recipient has informed the Provider beforehand in writing and in conformity with the GDPR.

5.2. Any request for transfer or provision of personal data to a third country, based on a court ruling or a decision by an administrative authority may only be complied with if the court ruling or decision is based on an international agreement, such as a treaty on mutual legal assistance between the third country submitting the request and the Union or a Member State. If this situation should arise, the Recipient will inform the Provider of the request immediately and prior to passing on the data.

6. Notification of a breach

6.1. The Recipient must immediately notify the Provider of every data leak they become aware of within the framework of the present collaboration in order to discuss the subsequent actions to be undertaken. All

this must be agreed within the framework of the parties' respective obligations to notify the supervisory authority. Not only must Recipient notify the Provider of any data leaks, but the Provider must also inform the supervisory authority thereof as quickly as possible.

6.2. Recipient must notify the Provider of a data leak within **24 hours** its discovery, and if possible, inform the Provider of any steps they have already undertaken. Recipient does not have an obligation to notify the supervisory authority.

6.3. If it is likely that the breach concerning personal data presents a risk to the rights and freedoms of natural persons, the Provider will, in turn, inform the supervisory authority of a data leak within 72 hours of its notification of discovery, and if possible, inform the supervisory authority of any steps that have already been undertaken. If sensitive data is involved, the Data Subject must also be informed.

6.4. The notification referred to in clause 6.1 will in any case contain the following description or information:

a) the nature of the data leak, if possible stating the categories of Data Subjects and registrations of personal data in question and, if they are to be approached, the number of Data Subjects and registers of personal data in question;

b) the name and contact details of the data protection officer or another contact person who can supply more information if these persons are available;

c) the probable consequences of the data leak insofar as they can be overseen by the Recipient;

d) the measures proposed by Recipient to tackle the data leak, including if applicable, measures to limit any harmful consequences that may arise from it.

7. Processing by third parties

7.1. Recipient will not employ any third-party data processor without first obtaining permission to do so from the Provider. In the case of general written consent, Recipient will inform the Provider of intended changes regarding the addition or replacement of third-party data processors, whereby the Provider will be given the opportunity to object to these changes.

7.2. If the Recipient employs a third-party data processor to perform specific processing activities on account of the Recipient, this third-party data processor will be bound through an agreement or other legal act pursuant to EU legislation or the laws of a Member State by the same obligations concerning data protection as those set out in this Agreement or other legal transaction between the Provider and Recipient, namely the obligation to provide sufficient guarantees with regard to the application of suitable technical and organisational measures to ensure that the processing is in compliance with prevailing legislation.

8. Liability

8.1. Unless explicitly agreed upon to the contrary, Recipient's obligations under this Agreement are on a best effort basis. Without prejudice to deviating mandatory legal provisions, Recipient is only liable for damage caused by non-compliance with these obligations if and insofar as this damage was caused by an intentional act, gross negligence or fraud. Recipient is not liable for any other errors.

8.2. The liability of either party to the other for any breach of this Agreement, any negligence or arising in any other way, whether direct or indirect, out of the subject matter of this Agreement, the Project and the Results, will not extend to any indirect damages or losses, or to any loss of profits, loss of revenue, loss of data, loss of contracts or opportunity even if the party bringing the claim has advised the other of the possibility of those losses, or if they were within the other party's contemplation.

=== END OF ADDENDUM ===

DTA_doc2:

SET II - Standard contractual clauses for the transfer of personal data from the Community to third countries (controller to controller transfers)

Data transfer agreement

between

Universiteit Gent (Ghent University), public institution with legal personality, having its administrative offices in Belgium, B-9000 Gent, Sint-Pietersnieuwstraat 25, company registration number 0248.015.142, for whom prof. dr. Rik Van de Walle, rector, for whom mr. Wim Van Camp, general manager UGent TechTransfer, acts by delegation pursuant to the Board of Governors' decision (hereinafter referred to as "data exporter");

and

[name of institution/company], having its offices in [full address], duly represented by [name of legal representative], [position] ("**Recipient**") (hereinafter "data importer");

The data exporter and the data importer are each referred to as a "party" and together as "the parties".

Definitions

For the purposes of the clauses:

(a) "personal data", "special categories of data/sensitive data", "process/processing", "controller", "processor", "data subject" and "supervisory authority/authority" shall have the same meaning as in Directive 95/46/EC of 24 October 1995 (whereby "the authority" shall mean the competent data protection authority in the territory in which the data exporter is established);
(b) "the data exporter" shall mean the controller who transfers the personal data;
(c) "the data importer" shall mean the controller who agrees to receive from the data exporter personal data for further processing in accordance with the terms of these clauses and who is not subject to a third country's system ensuring adequate protection;
(d) "clauses" shall mean these contractual clauses, which are a free-standing document that does not incorporate commercial business terms established by the parties under separate commercial arrangements.

The details of the transfer (as well as the personal data covered) are specified in Annex B, which forms an integral part of the clauses.

I. Obligations of the data exporter

The data exporter warrants and undertakes that:

(a) The personal data have been collected, processed and transferred in accordance with the laws applicable to the data exporter.
(b) It has used reasonable efforts to determine that the data importer is able to satisfy its legal obligations under these clauses.
(c) It will provide the data importer, when so requested, with copies of relevant data protection laws or references to them (where relevant, and not including legal advice) of the country in which the data exporter is established.
(d) It will respond to enquiries from data subjects and the authority concerning processing of the personal data by the data importer, unless the parties have agreed that the data importer will so respond, in which case the data exporter will still respond to the extent reasonably possible and with the information reasonably available to it if the data importer is unwilling or unable to respond. Responses will be made within a reasonable time.
(e) It will make available, upon request, a copy of the clauses to data subjects who are third party beneficiaries under clause III, unless the clauses contain confidential information, in which case it may remove such information. Where information is removed, the data exporter shall inform data subjects in writing of the reason for removal and of their right to draw the removal to the attention of the authority. However, the data exporter shall abide by a decision of the authority regarding access to the full text of the clauses by data subjects, as long as data subjects have agreed to respect the confidentiality of the confidential information removed. The data exporter shall also provide a copy of the clauses to the authority where required.

II. Obligations of the data importer

The data importer warrants and undertakes that:

(a) It will have in place appropriate technical and organisational measures to protect the personal data against accidental or unlawful destruction or accidental loss, alteration, unauthorised disclosure or access, and which provide a level of security appropriate to the risk represented by the processing and the nature of the data to be protected.
(b) It will have in place procedures so that any third party it authorises to have access to the personal data, including processors, will respect and maintain the confidentiality and security of the personal data. Any person acting under the authority of the data importer, including a data processor, shall be obligated to process the personal data only on instructions from the data importer. This provision does not apply to persons authorised or required by law or regulation to have access to the personal data.
(c) It has no reason to believe, at the time of entering into these clauses, in the existence of any local laws that would have a substantial adverse effect on the guarantees provided for under these clauses, and it will inform the data exporter (which will pass such notification on to the authority where required) if it becomes aware of any such laws.
(d) It will process the personal data for purposes described in Annex B, and has the legal authority to give the warranties and fulfil the undertakings set out in these clauses.

(e) It will identify to the data exporter a contact point within its organisation authorised to respond to enquiries concerning processing of the personal data, and will cooperate in good faith with the data exporter, the data subject and the authority concerning all such enquiries within a reasonable time. In case of legal dissolution of the data exporter, or if the parties have so agreed, the data importer will assume responsibility for compliance with the provisions of clause I(e).

(f) At the request of the data exporter, it will provide the data exporter with evidence of financial resources sufficient to fulfil its responsibilities under clause III (which may include insurance coverage).

(g) Upon reasonable request of the data exporter, it will submit its data processing facilities, data files and documentation needed for processing to reviewing, auditing and/or certifying by the data exporter (or any independent or impartial inspection agents or auditors, selected by the data exporter and not reasonably objected to by the data importer) to ascertain compliance with the warranties and undertakings in these clauses, with reasonable notice and during regular business hours. The request will be subject to any necessary consent or approval from a regulatory or supervisory authority within the country of the data importer, which consent or approval the data importer will attempt to obtain in a timely fashion.

(h) It will process the personal data, at its option, in accordance with:

(i) the data protection laws of the country in which the data exporter is established, or

(ii) the relevant provisions[1] of any Commission decision pursuant to Article 25(6) of Directive 95/46/EC, where the data importer complies with the relevant provisions of such an authorisation or decision and is based in a country to which such an authorisation or decision pertains, but is not covered by such authorisation or decision for the purposes of the transfer(s) of the personal data[2], or

(iii) the data processing principles set forth in Annex A.

Data importer to indicate which option it selects: iii

Initials of data importer: SS;

(i) It will not disclose or transfer the personal data to a third party data controller located outside the European Economic Area (EEA) unless it notifies the data exporter about the transfer and

(i) the third party data controller processes the personal data in accordance with a Commission decision finding that a third country provides adequate protection, or

---

[1] “Relevant provisions” means those provisions of any authorisation or decision except for the enforcement provisions of any authorisation or decision (which shall be governed by these clauses).

[2] However, the provisions of Annex A.5 concerning rights of access, rectification, deletion and objection must be applied when this option is chosen and take precedence over any comparable provisions of the Commission Decision selected.

(ii) the third party data controller becomes a signatory to these clauses or another data transfer agreement approved by a competent authority in the EU, or

(iii) data subjects have been given the opportunity to object, after having been informed of the purposes of the transfer, the categories of recipients and the fact that the countries to which data is exported may have different data protection standards, or

(iv) with regard to onward transfers of sensitive data, data subjects have given their unambiguous consent to the onward transfer

III. Liability and third party rights

(a) Each party shall be liable to the other parties for damages it causes by any breach of these clauses. Liability as between the parties is limited to actual damage suffered. Punitive damages (i.e. damages intended to punish a party for its outrageous conduct) are specifically excluded. Each party shall be liable to data subjects for damages it causes by any breach of third party rights under these clauses. This does not affect the liability of the data exporter under its data protection law.

(b) The parties agree that a data subject shall have the right to enforce as a third party beneficiary this clause and clauses I(b), I(d), I(e), II(a), II(c), II(d), II(e), II(h), II(i), III(a), V, VI(d) and VII against the data importer or the data exporter, for their respective breach of their contractual obligations, with regard to his personal data, and accept jurisdiction for this purpose in the data exporter’s country of establishment. In cases involving allegations of breach by the data importer, the data subject must first request the data exporter to take appropriate action to enforce his rights against the data importer; if the data exporter does not take such action within a reasonable period (which under normal circumstances would be one month), the data subject may then enforce his rights against the data importer directly. A data subject is entitled to proceed directly against a data exporter that has failed to use reasonable efforts to determine that the data importer is able to satisfy its legal obligations under these clauses (the data exporter shall have the burden to prove that it took reasonable efforts).

IV. Law applicable to the clauses

These clauses shall be governed by the law of the country in which the data exporter is established, with the exception of the laws and regulations relating to processing of the personal data by the data importer under clause II(h), which shall apply only if so selected by the data importer under that clause.

V. Resolution of disputes with data subjects or the authority

(a) In the event of a dispute or claim brought by a data subject or the authority concerning the processing of the personal data against either or both of the parties, the parties will inform each other about any such disputes or claims, and will cooperate with a view to settling them amicably in a timely fashion.

(b) The parties agree to respond to any generally available non-binding mediation procedure initiated by a data subject or by the authority. If they do participate in the proceedings, the

parties may elect to do so remotely (such as by telephone or other electronic means). The parties also agree to consider participating in any other arbitration, mediation or other dispute resolution proceedings developed for data protection disputes.

(c) Each party shall abide by a decision of a competent court of the data exporter's country of establishment or of the authority which is final and against which no further appeal is possible.

VI. Termination

(a) In the event that the data importer is in breach of its obligations under these clauses, then the data exporter may temporarily suspend the transfer of personal data to the data importer until the breach is repaired or the contract is terminated.

(b) In the event that:

(i) the transfer of personal data to the data importer has been temporarily suspended by the data exporter for longer than one month pursuant to paragraph (a);

(ii) compliance by the data importer with these clauses would put it in breach of its legal or regulatory obligations in the country of import;

(iii) the data importer is in substantial or persistent breach of any warranties or undertakings given by it under these clauses;

(iv) a final decision against which no further appeal is possible of a competent court of the data exporter's country of establishment or of the authority rules that there has been a breach of the clauses by the data importer or the data exporter; or

(v) a petition is presented for the administration or winding up of the data importer, whether in its personal or business capacity, which petition is not dismissed within the applicable period for such dismissal under applicable law; a winding up order is made; a receiver is appointed over any of its assets; a trustee in bankruptcy is appointed, if the data importer is an individual; a company voluntary arrangement is commenced by it; or any equivalent event in any jurisdiction occurs

then the data exporter, without prejudice to any other rights which it may have against the data importer, shall be entitled to terminate these clauses, in which case the authority shall be informed where required. In cases covered by (i), (ii), or (iv) above the data importer may also terminate these clauses.

(c) Either party may terminate these clauses if (i) any Commission positive adequacy decision under Article 25(6) of Directive 95/46/EC (or any superseding text) is issued in relation to the country (or a sector thereof) to which the data is transferred and processed by the data importer, or (ii) Directive 95/46/EC (or any superseding text) becomes directly applicable in such country.

(d) The parties agree that the termination of these clauses at any time, in any circumstances and for whatever reason (except for termination under clause VI(c)) does not exempt them from the obligations and/or conditions under the clauses as regards the processing of the personal data transferred.

VII. Variation of these clauses

The parties may not modify these clauses except to update any information in Annex B, in which case they will inform the authority where required. This does not preclude the parties from adding additional commercial clauses where required.

VIII. Description of the Transfer

The details of the transfer and of the personal data are specified in Annex B. The parties agree that Annex B may contain confidential business information which they will not disclose to third parties, except as required by law or in response to a competent regulatory or government agency, or as required under clause I(e). The parties may execute additional annexes to cover additional transfers, which will be submitted to the authority where required. Annex B may, in the alternative, be drafted to cover multiple transfers.

Dated: [date]

FOR DATA IMPORTER	FOR DATA EXPORTER
Signature	Signature
[legal representative]	Wim Van Camp
[position]	General manager UGent TechTrasfer
[principal investigator]	[principal investigator]
[position and department, if applicable]	[position and department]

ANNEX A DATA PROCESSING PRINCIPLES

1. Purpose limitation: Personal data may be processed and subsequently used or further communicated only for purposes described in Annex B or subsequently authorised by the data subject.

2. Data quality and proportionality: Personal data must be accurate and, where necessary, kept up to date. The personal data must be adequate, relevant and not excessive in relation to the purposes for which they are transferred and further processed.

3. Transparency: Data subjects must be provided with information necessary to ensure fair processing (such as information about the purposes of processing and about the transfer), unless such information has already been given by the data exporter.

4. Security and confidentiality: Technical and organisational security measures must be taken by the data controller that are appropriate to the risks, such as against accidental or unlawful destruction or accidental loss, alteration, unauthorised disclosure or access, presented by the processing. Any person acting under the authority of the data controller, including a processor, must not process the data except on instructions from the data controller.

5. Rights of access, rectification, deletion and objection: As provided in Article 12 of Directive 95/46/EC, data subjects must, whether directly or via a third party, be provided with the personal information about them that an organisation holds, except for requests which are manifestly abusive, based on unreasonable intervals or their number or repetitive or systematic nature, or for which access need not be granted under the law of the country of the data exporter. Provided that the authority has given its prior approval, access need also not be granted when doing so would be likely to seriously harm the interests of the data importer or other organisations dealing with the data importer and such interests are not overridden by the interests for fundamental rights and freedoms of the data subject. The sources of the personal data need not be identified when this is not possible by reasonable efforts, or where the rights of persons other than the individual would be violated. Data subjects must be able to have the personal information about them rectified, amended, or deleted where it is inaccurate or processed against these principles. If there are compelling grounds to doubt the legitimacy of the request, the organisation may require further justifications before proceeding to rectification, amendment or deletion. Notification of any rectification, amendment or deletion to third parties to whom the data have been disclosed need not be made when this involves a disproportionate effort. A data subject must also be able to object to the processing of the personal data relating to him if there are compelling legitimate grounds relating to his particular situation. The burden of proof for any refusal rests on the data importer, and the data subject may always challenge a refusal before the authority.

6. Sensitive data: The data importer shall take such additional measures (e.g. relating to security) as are necessary to protect such sensitive data in accordance with its obligations under clause II.

7. Data used for marketing purposes: Where data are processed for the purposes of direct marketing, effective procedures should exist allowing the data subject at any time to “opt-out” from having his data used for such purposes.

8. Automated decisions: For purposes hereof “automated decision” shall mean a decision by the data exporter or the data importer which produces legal effects concerning a data subject or significantly affects a data subject and which is based solely on automated processing of personal data intended to evaluate certain personal aspects relating to him, such as his performance at work, creditworthiness, reliability, conduct, etc. The data importer shall not make any automated decisions concerning data subjects, except when:

   (a) (i) such decisions are made by the data importer in entering into or performing a contract with the data subject, and

   (ii) the data subject is given an opportunity to discuss the results of a relevant automated decision with a representative of the parties making such decision or otherwise to make representations to that parties.

   or

   (b) where otherwise provided by the law of the data exporter.

ANNEX B DESCRIPTION OF THE TRANSFER

Data subjects

The personal data transferred concerns the following categories of data subjects:

a) [specify category]
b) [specify category]

Purpose of the transfer(s)

The transfer is made for the following purposes:

[description]

Categories of data

The personal data transferred concern the following categories of data: [format of data, EGA identified code if applicable]:

[description of the dataset]

Recipients

The personal data transferred may be discloses only to the following recipients or categories of recipients: [full name of principal investigator] and members of her/his research group [name of the department] at [name of institution/company]

Sensitive data (if appropriate):

The personal data transferred concern the following categories of sensitive data:

Genetic information

Data protection registration information of data exporter (where applicable):

The personal data transferred concern the following categories of sensitive data: Genetic information

Additional useful information (storage limits and other relevant information): [specify, if applicable]

Contact points for data protection enquiries:

Data importer	Data exporter
[full name principal investigator]	[full name of UGent data exporter(s)]
